# *Epi*-Cyclophellitol Cyclosulfate, a Mechanism-Based Endoplasmic Reticulum α-Glucosidase II Inhibitor, Blocks Replication of SARS-CoV-2 and Other Coronaviruses

**DOI:** 10.1021/acscentsci.4c00506

**Published:** 2024-07-25

**Authors:** Melissa Thaler, Tim P. Ofman, Ken Kok, Jurriaan J. A. Heming, Elisha Moran, Isabelle Pickles, Anouk A. Leijs, Adrianus M. C. H. van den Nieuwendijk, Richard J. B. H. N. van den Berg, Gijs Ruijgrok, Zachary Armstrong, Clarisse Salgado-Benvindo, Dennis K. Ninaber, Eric J. Snijder, Constant A. A. van Boeckel, Marta Artola, Gideon J. Davies, Herman S. Overkleeft, Martijn J. van Hemert

**Affiliations:** † Leiden University Center for Infectious Diseases (LUCID), Leiden University Medical Center, 2333 ZA Leiden, The Netherlands; ‡ Leiden Institute of Chemistry, Leiden University, 2311 EZ Leiden, The Netherlands; § Department of Chemistry, University of York, York YO10 5DD, United Kingdom; ∥ Department of Pulmonology, Leiden University Medical Center, 2333 ZA Leiden, The Netherlands

## Abstract

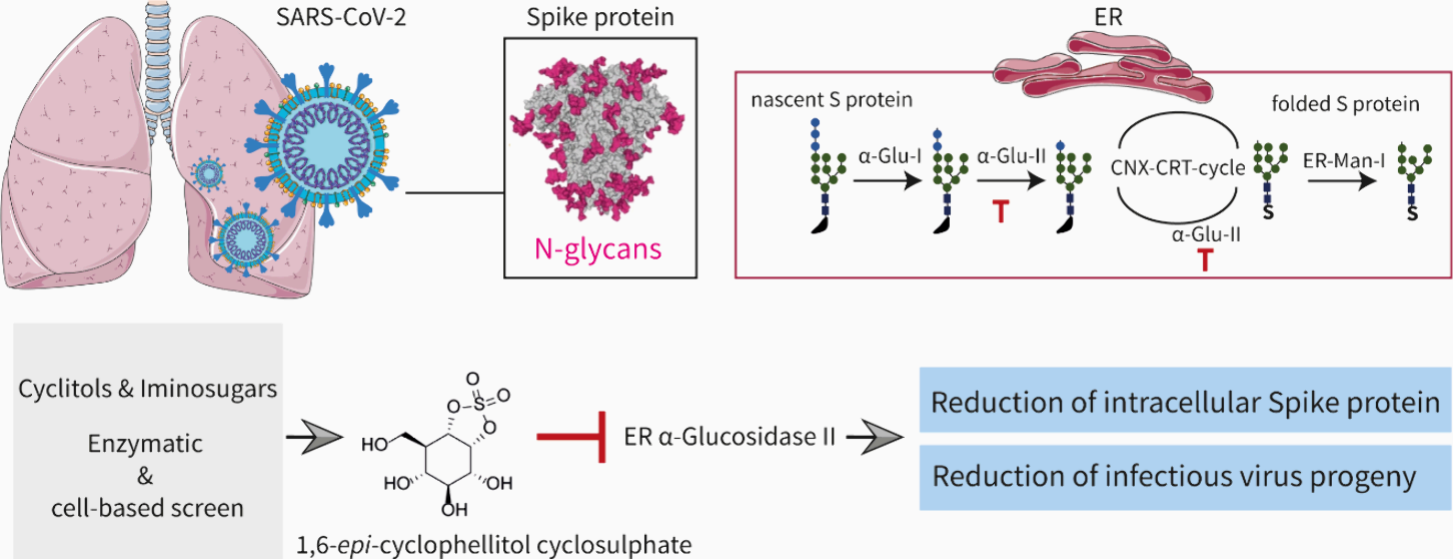

The combined inhibition of endoplasmic reticulum (ER) α-glucosidases I and II has been shown to inhibit replication of a broad range of viruses that rely on ER protein quality control. We found, by screening a panel of deoxynojirimycin and cyclitol glycomimetics, that the mechanism-based ER α-glucosidase II inhibitor, 1,6-*epi*-cyclophellitol cyclosulfate, potently blocks SARS-CoV-2 replication in lung epithelial cells, halting intracellular generation of mature spike protein, reducing production of infectious progeny, and leading to reduced syncytium formation. Through activity-based protein profiling, we confirmed ER α-glucosidase II inhibition in primary airway epithelial cells, grown at the air–liquid interface. 1,6-*epi*-Cyclophellitol cyclosulfate inhibits early pandemic and more recent SARS-CoV-2 variants, as well as SARS-CoV and MERS-CoV. The reported antiviral activity is comparable to the best-in-class described glucosidase inhibitors, all competitive inhibitors also targeting ER α-glucosidase I and other glycoprocessing enzymes not involved in ER protein quality control. We propose selective blocking ER-resident α-glucosidase II in a covalent and irreversible manner as a new strategy in the search for effective antiviral agents targeting SARS-CoV-2 and other viruses that rely on ER protein quality control.

## Abstract 2

Screening of deoxynojirimycin and cyclitol glycomimetics identified mechanism-based ER α-glucosidase II inhibitor 1,6-*epi*-cyclophellitol cyclosulfate as a potent inhibitor of SARS-CoV-2 replication.

## Introduction

**Figure 1:**
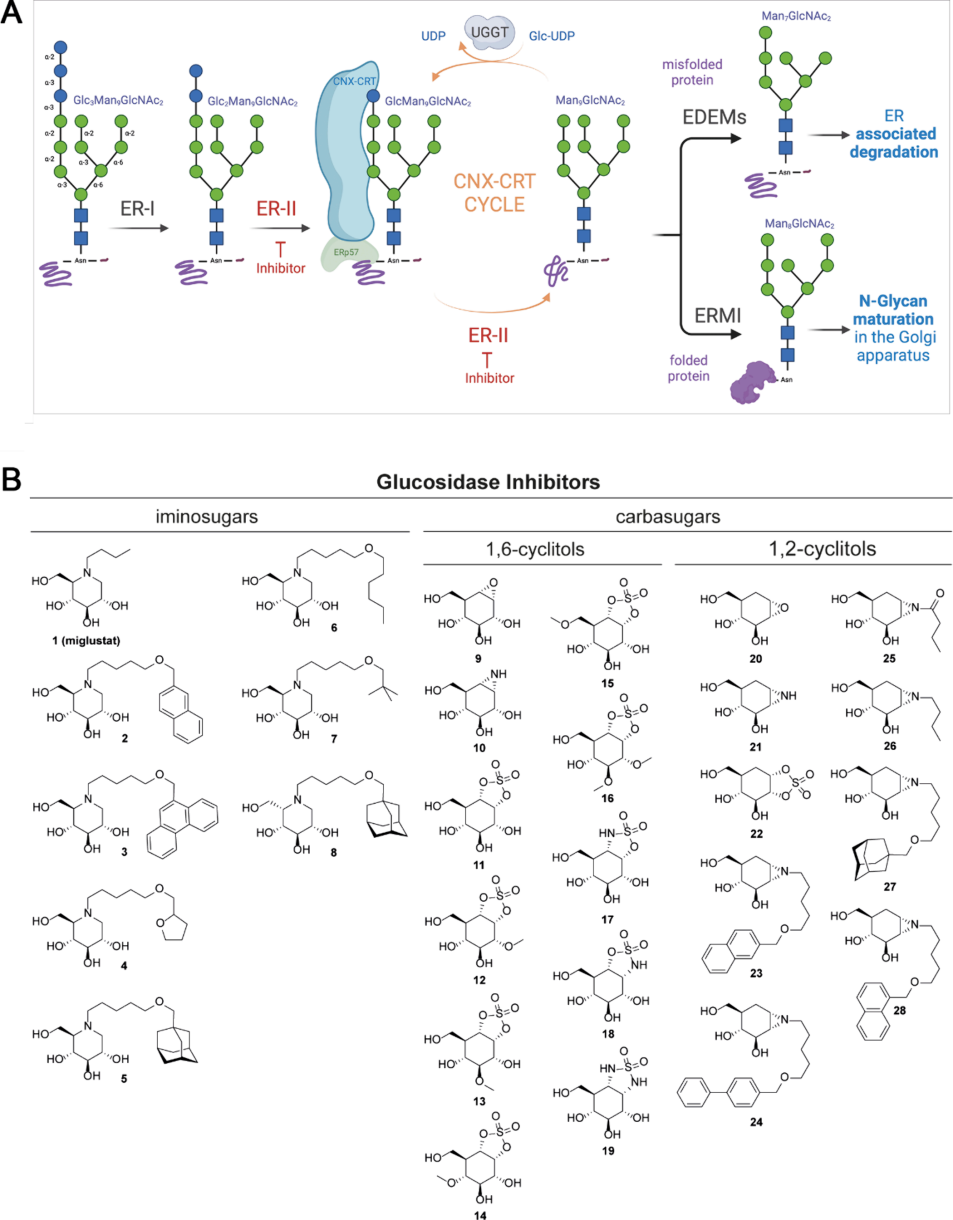
(A) Schematic of N-glycan processing of newly synthesized proteins in the ER lumen. Folding of nascent proteins in the ER is promoted by the calnexin–calreticulin cycle (CNX–CRT cycle), which relies on glycan trimming by ER α-Glu II (ER-II). (B) Focused library of 28 iminosugars and cyclitol subjects of the here-presented studies.

Coronaviruses, like many other virus groups, use the host machinery for co- and post-translational formation and processing of N-linked glycans. N-linked oligosaccharides are crucial for the proper protein folding, stability, and functioning of many proteins that are part of viral envelopes.^1^ In the endoplasmic reticulum (ER), α-glucosidases I and II (α-Glu I and α-Glu II) are responsible for trimming the terminal glucose moieties of nascent N-glycans (Figure 1A), and the resultant monoglucosylated N-glycans are subsequently recognized by the ER chaperones calnexin and calreticulin (CNX–CRT cycle),^2,3^ which prevent protein aggregation and assist in polypeptide folding. When a protein fails to fold correctly, glycoprotein glycosyltransferase (UGGT) reconstructs the monoglucosylated G1M9 *N*-glycan, enabling another round of refolding attempts facilitated by the CNX–CRT chaperones. Upon proper folding of the protein, the final glucose residue in high-mannose-type N-glycans is removed by α-Glu II, leading to further trimming by ER α-mannosidase I (ERMI), after which the *N*-glycoproteins are routed to the Golgi apparatus for N-glycan maturation and further post-translational modification events en route to their final destination. Glycoproteins that fail to attain their proper conformation undergo mannose trimming orchestrated by the ER degradation-enhancing mannosidase-like proteins (EDEMs) and ultimately are routed toward the ER-associated degradation (ERAD) machinery. Inhibition of ER α-Glu I and II has been shown to interfere with proper processing of nascent proteins through the CNX–CRT cycle, leading to their inappropriate folding, eventual dislocation from the ER, and proteasomal degradation.^4^ This holds true for host and viral *N*-glycoproteins alike, and ER α-Glu I/II inhibition has therefore been considered as a viable strategy for antiviral therapeutics development for several decades.^5,6^

Many studies have reported the ability of iminosugars to inhibit replication of various viruses, through the blocking of ER protein quality control via ER α-Glu I/II inhibition.^7^ Iminosugars are polyhydroxylated glycomimetic alkaloids featuring a basic amine, replacing the sugar ring oxygen, that is thought to interact with glycosidase active site residues that partake in enzymatic glycosidic bond hydrolysis.^8,9^ The potential of iminosugars as antivirals was first reported in 1987^5,10,11^ in the context of human immunodeficiency virus (HIV), which relies on the host ER machinery for glycoprotein processing.^12^ These studies revealed that the two iminosugar compounds, deoxynojirimycin and castanospermine, as well as some structural analogues thereof, inhibit ER α-Glu I and II and block the production of HIV infectious progeny *in vitro*. Later studies using a host of structurally diverse iminosugars described blocking replication of a broad range of viruses *in vitro* and *in vivo*, including influenza viruses,^13−15^ severe acute respiratory syndrome coronavirus (SARS-CoV),^16^ dengue virus, and the hemorrhagic fever viruses Marburg and Ebola.^17,18^ One of the studied iminosugars, UV-4B, showed promising results in mice, as a single high dose, which caused hallmarks of ER α-Glu I inhibition *in vivo*, protected the animals from a lethal dose of DENV or influenza virus.^15^ Interestingly, patients that have N-glycosylation defects (defects in ER α-Glu I) due to a congenital disorder have also reduced susceptibility to infection with enveloped viruses that depend on host glycan processing for their replication.^19^ Despite promising *in vitro* studies, phase II clinical trials with the iminosugar Celgosivir (a prodrug form of castanospermine) showed no beneficial outcomes when it was used as monotherapy for dengue and hepatitis C viral infections.^20,21^ Most recently, a range of competitive α-glucosidase inhibitors have been studied during the search for antivirals against SARS-CoV-2.^22−24^ The spike (S) protein of SARS-CoV-2, one of the envelope proteins on the virus surface, is heavily glycosylated with 23 reported N-glycan sites.^25^ Besides shielding of antibody epitopes,^26^ and modulating protein structure, N-glycosylation of S protein and its receptor binding domain (RBD) is crucial for virus infectivity, as the S protein drives virus entry by binding to the host receptor ACE2 and mediates fusion between the virus and host cell membrane.^27^ N-glycans and their modulation through deletion of specific sites on the RBD were reported to be important for conformational stability and accessibility of the RBD for ACE2 binding.^28−31^ Therefore, the incorporation of nonfunctional immaturely glycosylated S proteins can reduce the specific infectivity of progeny virions.^16,32^ Disruption of the CNX–CRT-mediated glycoprotein processing, by iminosugars specifically, was reported to reduce the incorporation of S protein into SARS-CoV pseudovirus particles.^16^ In this study, it was suggested that ER α-Glu I/II inhibition could lead to both the degradation of improperly processed S proteins in the ER and the incorporation of incompletely glycosylated S proteins into virus particles, thus having a two-pronged mode of action.

Despite the decades of research on iminosugars, no small molecules inhibiting ER α-Glu have proceeded beyond phase II clinical trials^33,34^ as antivirals. With the aim of uncovering alternative inhibitor designs for antiviral drug discovery, and building upon our recent studies on mechanism-based, covalent and irreversible glycosidase inhibition,^35−40^ we decided to assess a panel of mechanism-based inhibitors, side by side with a set of classical *N*-alkyl iminosugars, for their ability to inhibit SARS-CoV-2 replication through inhibition of ER α-Glu I and II. While performing the same net transformation (hydrolysis of α-glucosidic linkages), ER α-Glu I and II do so with distinct mechanisms. Both enzymes feature a carboxylic acid and a carboxylate containing amino acid in their active sites and process their substrate by acid catalysis.^8,9^ Both enzymes are therefore amenable to inhibition by a basic, glucose-mimetic iminosugar. In contrast to ER α-Glu I, ER α-Glu II forms a covalent intermediate with its substrate during processing by utilizing one of the carboxylates as a nucleophile. This nucleophile can be trapped by glucomimetic cyclitols endowed with an electrophile (epoxide, aziridine, or cyclic sulfate). We have shown in the past that 1,6-*epi*-cyclophellitol (**9**, Figure 1) as well as its aziridine (**10**) and cyclic sulfate (**11**) analogues potently and selectively blocks ER α-Glu II.^35^ In this study, we screened members of both compound classes, cyclitols and iminosugars, for their inhibition of ER α-Glu II and antiviral activity against SARS-CoV-2. We demonstrate that 1,6-*epi*-cyclophellitol cyclosulfate (**11**) most potently reduces the enzyme activity of α-Glu II and exerts the best antiviral efficacy against SARS-CoV-2. We also show that this compound blocks replication of all SARS-CoV-2 variants tested, as well as the pathogenic SARS-CoV and MERS-CoV, making it an interesting lead for further exploration toward a new class of antiviral drugs.

## Results

### Efficacy of Glucosidase Inhibitors against SARS-CoV-2 Correlates with Their Activity against ER α-Glucosidase II

The panel of iminosugars and cyclitols, subject of the here-presented studies, is depicted in Figure 1B. With respect to the iminosugars, and to keep in line with literature precedents, we selected *N*-alkyl deoxynojirimycins **1**–**8**. Deoxynojirimycin (DNJ) features the glucopyranose configuration, and *N*-alkyl derivatives have been shown to be more effective glucosidase inhibitors compared to nonsubstituted DNJ.^41−43^ This includes the benchmark analogue, *N*-butyl-DNJ **1** (Miglustat, Zavesca) which is part of almost all antiviral studies on iminosugars targeting α-Glu I/II. In fact, Miglustat is a clinical drug for the treatment of Gaucher disease and acts as a glucosylceramidase (GCS) inhibitor.^44^ It also inhibits the human retaining β-glucosidases, GBA1, GBA2, and GBA3, displaying a rather broad activity profile across various glycoprocessing enzymes not involved in ER protein quality control. Besides Miglustat **1**, we included DNJ derivatives **2**–**8** to assess the influence of the hydrophobic *N*-alkyl substituent on antiviral activity. Compound **8** has the l-*ido* configuration and comprises the C6 epimer (glucopyranose numbering) of DNJ derivative **5**. Compared to **5**, l-*ido*-DNJ **8** is a much weaker ER α-Glu inhibitor, which should be reflected in its antiviral potency. With respect to the cyclitols, we previously published 1,6-*epi*-cyclophellitol **9**, 1,6-*epi*-cyclophellitol aziridine **10**, and 1,6-*epi*-cyclophellitol cyclosulfate **11** as potent and selective, mechanism-based, covalent and irreversible retaining α-glucosidase inhibitors.^35,45^ Besides inhibiting ER α-Glu II, the single detected off-target (in the context of pharmacological ER protein quality control interference) is the lysosomal α-glucosidase, human acid α-glucosidase GAA. These 1,6-*epi*-cyclophellitol analogues were designed to inhibit retaining α-glucosidases exclusively (so, not inverting ones like α-Glu I), and while epoxide **9** and aziridine **10** partially inhibit the retaining β-glucosidases, GBA1 and GBA2, cyclosulfate **11** is completely inactive toward these enzymes. We also found that tempering the electrophilicity, as in cyclosulfamidates **17** and **18** and cyclosulfamide **19**, yields competitive retaining α-glucosidase inhibitors, and to investigate the effect of going from covalent to competitive inhihition within the same compound class, we included these compounds in our assays. In addition, we tested a number of structural cyclitol variations. These include 1,2-*epi*-cyclophellitols (**20**–**22**), which may block α-Glu II in a covalent, irreversible manner similar to the 1,6-*epi*-cyclophellitols.^46^ A number of partially O-methylated cyclosulfates (**12**–**16**) were included to assess the effect of polarity, while compounds **23**–**28** were designed to contain alkyl substituents also present in the iminosugar series tested. The synthesis of the iminosugar and cyclitol inhibitors **1**–**11**, **17**–**22**, **25**, and **26** have been published previously.^35,41−43,45,47^ The synthesis of methylated sulfates **12**–**16** and alkyl aziridines **23**, **24**, **27**, and **28** can be found in the Supporting Information (Schemes S1–S5).

**Figure 2:**
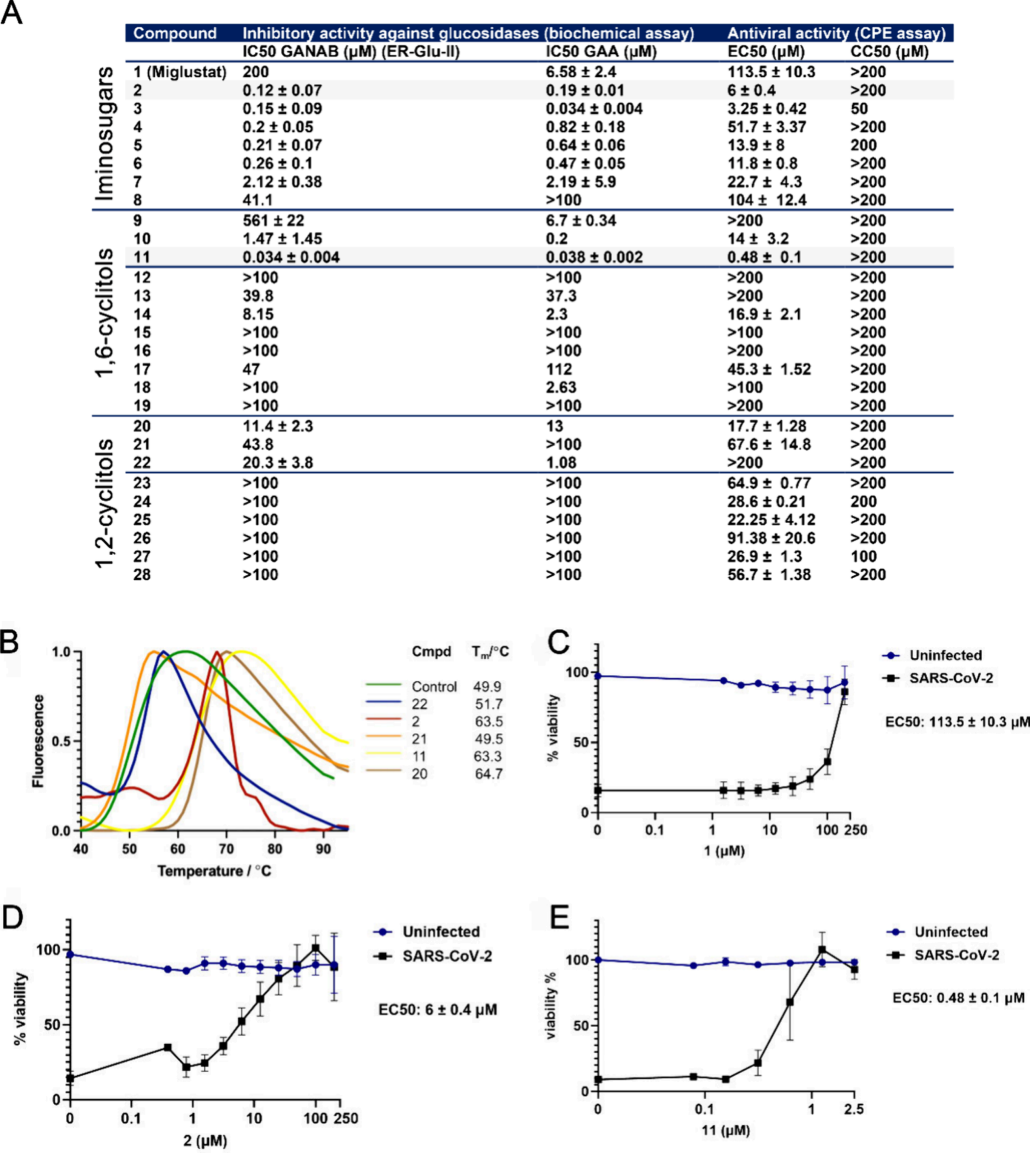
ER α-Glu II inhibitory potency correlates with reduction of SARS-CoV-2 mediated cytopathic effect in cell culture. (A) IC_50_ values of compounds in *in vitro* enzyme activity assays with ER α-Glu II and GAA, and EC_50_ and CC_50_ values of compounds determined by CPE reduction assays with SARS-CoV-2. (B) Thermal shift profile of preincubated ER α-Glu II with inhibitors. (C–E) SARS-CoV-2 CPE reduction assay dose–response curves of (C) Miglustat **1**, (D) naphthyl-deoxynojirimycin **2**, and (E) cyclosulfate **11**. *n* = 3 independent experiments. The viability of uninfected compound-treated cells was established by MTS assay in parallel. Mean ± SEM values are shown. The 50% inhibitory concentration (EC_50_) values were determined by nonlinear regression with GraphPad Prism 6.

The inhibitory effect of all synthesized molecules on the activity of GAA and endoplasmic reticulum α-glucosidase II (ER α-Glu II, GANAB) was determined following *in vitro* enzyme activity methods reported previously,^35^ using 4-methylumbelliferyl-α-d-glucopyranoside (4-MU-α-Glc) as a fluorogenic substrate and measuring the amount of 4-MU-mediated fluorescence (Figure 2A, left panel). *N*-Alkyldeoxynojirimycins **1**–**8** all inhibited both ER α-Glu II and GAA, but with potencies varying from the nanomolar to the micromolar range. *N*-Alkyl-iminosugars **2**–**7**, featuring an extended lipophilic *N*-alkyl moiety relative to *N*-butyl-DNJ **1**, inhibited both enzymes rather more potently than this benchmark iminosugar, with **2** showing high potencies for both ER α-Glu II (IC_50_ = 0.3 ± 0.07 μM) and GAA (IC_50_ = 1.1 ± 0.09 μM). l-*ido*-Deoxynojirimycin **8** is a much weaker ER α-Glu II inhibitor than its d-*gluco* isoster **5** (both compounds containing the same adamantane-modified *N*-alkyl chain), and it showed no activity against GAA at the measured concentrations. These results match the literature trend indicating that large, hydrophobic *N*-alkyl appendages positively influence glucosidase inhibitory potency in this class of compound.^41−43,47^

With respect to the cyclitol class of compounds, 1,6-*epi*-cyclophellitol cyclosulfate **11** proved to be the most potent ER α-Glu II inhibitor of all compounds tested, with an IC_50_ value of 0.03 ± 0.007 μM. Cyclosulfate **11** was also and, together with naphthyl-iminosugar **2**, the most potent of the GAA inhibitors. Methylation of either of the four hydroxyls (or combinations thereof) in **11**, as in 1,6-*epi*-cyclophellitol cyclosulfates **12**–**16**, proved detrimental to inhibitory potency, though 4-*O*-methyl derivative **14** with IC_50_ values of 8.2 ± 0.1 μM for ER α-Glu II and 2.2 ± 0.09 μM for GAA still outperformed Miglustat (**1**) as an inhibitor of both of these enzymes. Moving from covalent (cyclosulfate, **11**) to competitive (**17**–**19**) cyclitol designs proved detrimental for ER α-Glu II inhibition, although compound **18** retains remarkable (IC_50_ = 6.1 ± 1.3 μM) inhibitory activity against GAA. 1,2-*epi*-Cyclitols **20**–**22** turned out to be only moderately active ER α-Glu II inhibitors. In contrast to the 1,6-analogues (**9**–**11**), where the cyclosulfate was more potent compared to the aziridine and epoxide, epoxide **20** was the most potent of this series.^46^ Interestingly, 1,2-cyclosulfate **22** proved to be a rather potent GAA inhibitor, much more so than epoxide **20** and aziridine **21**, suggesting that conformational aspects (the epoxide and aziridine likely enforcing a half-chair conformation with respect to the cyclitol ring where the cyclosulfate will allow a chairlike conformation) are in play for this enzyme. Finally, and in contrast to what was observed for the competitive inhibitor series **1**–**8**, 1,2-cyclophellitol aziridines **23**–**28** bearing an *N*-alkyl chain (and in the case of **25** an *N*-acyl one) are much worse inhibitors for both enzymes tested (no significant inhibition up to 100 μM) when compared to the nonsubstituted aziridine **21**. In all, 1,6-*epi*-cyclophellitol cyclosulfate **11** is the most potent ER α-Glu II inhibitor, with naphthylated deoxynojirimycin **2** as the most effective of the competitive inhibitors almost on a par with **11**.

To confirm the stabilizing effect of these two compounds on the enzyme, we performed a thermal stability assay with these, as well as with the less potent inhibitors **20**–**22**, on recombinant *Mus musculus* α-Glu II, a mouse enzyme with high sequence homology to the human enzyme (Figure 2B). ER α-Glu II denaturation as a consequence of heat exposure, as well as the effect of active site-binding inhibitors on the denaturation temperature, can be monitored by a naturally quenched SYPRO orange dye. Upon denaturation of a protein, hydrophobic regions are exposed to which the dye binds, demonstrating a distinct difference in melting temperature (*T*_m_) for each inhibitor compared to the unliganded ER α-Glu II control. *Mm*α-Glu II preincubated with compound **11** or **2** displayed melting temperatures (*T*_m_) of 63.3 and 63.5 °C, respectively, whereas the unliganded enzyme denatured at approximately 15 °C lower (*T*_m_ = 49.9 °C). In comparison, compounds **21** and **22** gave no (49.5 °C) to marginal (51.7 °C) *T*_m_ increases, while epoxide **20**, which had the best efficacy of all 1,2-*epi*-cyclophellitols in the enzyme activity assay, gave a remarkably high *T*_m_ of 64.7 °C.

To elucidate the structure–activity relationship and predict the binding mode of the compounds before and after the covalent reaction with the nucleophilic aspartate, docking into ER α-Glu II was performed for compounds **11**, **10** and **9**. The top scoring pose of **11**, **10**, and **9** after noncovalent docking using Glide (in the Schrödinger Maestro GUI) was overlaid with the bound d-glucose molecule from the original PDB file (PDB: 5H9O) as a measure of the accuracy of the pose. The compound adopted a near-identical conformation in the binding site (Figure S1A). The ligand was also subjected to covalent docking to mimic a postreaction conformation. The outputted poses made the same hydrogen bonding interactions as the noncovalently docked pose. The top poses were overlaid with a PDB file containing a 5-fluoro-α-d-glucopyranosyl (PDB: 5HJR); the poses overlaid well in a skewed boat confirmation (Figure S1B–D), suggesting confidence in the docking results. These binding pose predictions suggest compounds **11**, **10**, and **9** are orientated correctly in the binding site of ER α-Glu II to facilitate a covalent reaction with Asp564.

All compounds were then analyzed for their antiviral activities against SARS-CoV-2, in cytopathic effect (CPE) reduction assays, in which Vero E6 cells were pretreated and infected with SARS-CoV-2 in the presence of various concentrations of a compound. Three days postinfection cell viability was measured and EC_50_ values (compound concentration at which 50% of cell viability is reached as compared to the nontreated, infected cells) were determined (Figure 2A, right panel). Simultaneously, uninfected cells were treated with the same concentrations of compound to determine the CC_50_ (compound concentration at which cell viability is 50% of that of untreated cells due to cytotoxicity). All iminosugars **1**–**8** protected cells from SARS-CoV-2 infection in this assay, and naphthyl deoxynojirimycin **2**, being the most potent competitive ER α-Glu II inhibitor from the enzyme activity assay, also displayed the highest efficacy of the eight iminosugars assessed in blocking SARS-CoV-2 replication, with an EC_50_ value of 6 ± 0.4 μM (Figure 2D). Similar deoxynojirimycin derivatives were previously reported to have activity against SARS-CoV-2.^24,48^ UV-4, an iminosugar that was previously described to be efficacious in a mouse model,^13^ was tested in parallel, and its activity was compared to those of compounds **11** and **2**. The antiviral efficacy of UV-4 was similar to that of our iminosugar compound **2** (Figure S2A). In contrast, the EC_50_ value in the CPE assay for Miglustat **1** was above 100 μM (Figure 2C), which correlates to other studies which found limited antiviral activity for this compound against SARS-CoV-2.^24,49^ 1,6-*epi*-Cyclophellitol cyclosulfate **11**, our most potent ER α-Glu II inhibitor, also proved to be the most potent SARS-CoV-2 replication inhibitor of all compounds tested, with an EC_50_ value of 0.48 ± 0.1 μM (Figure 2E). This matches our general finding that ER α-Glu II inhibitory potency correlates with anti-SARS-CoV-2 replication efficacy (Figure 2A). Selective ER α-Glu II inhibition thus appears a promising strategy in the discovery of new antiviral agents. To validate the results obtained in the Vero E6 cell based assays, CPE reduction assays on H1299/ACE2 lung epithelial cells were performed with compounds **11**, **2**, and UV-4. With these human lung cells, comparable EC_50_ values were obtained (Figure S2B).

Given that 1,6-*epi*-cyclophellitol cyclosulfate **11** came out as the most potent compound in both the enzyme inhibition and SARS-CoV-2 CPE assays, and that this compound class, in contrast to that of iminosugars, comprises a new design class, we decided to further profile this inhibitor in more advanced virological assays to study its efficacy and mechanism of action.

### 1,6-*epi*-Cyclophellitol Cyclosulfate Reduces SARS-CoV-2 Infectious Progeny in Cell Culture

**Figure 3:**
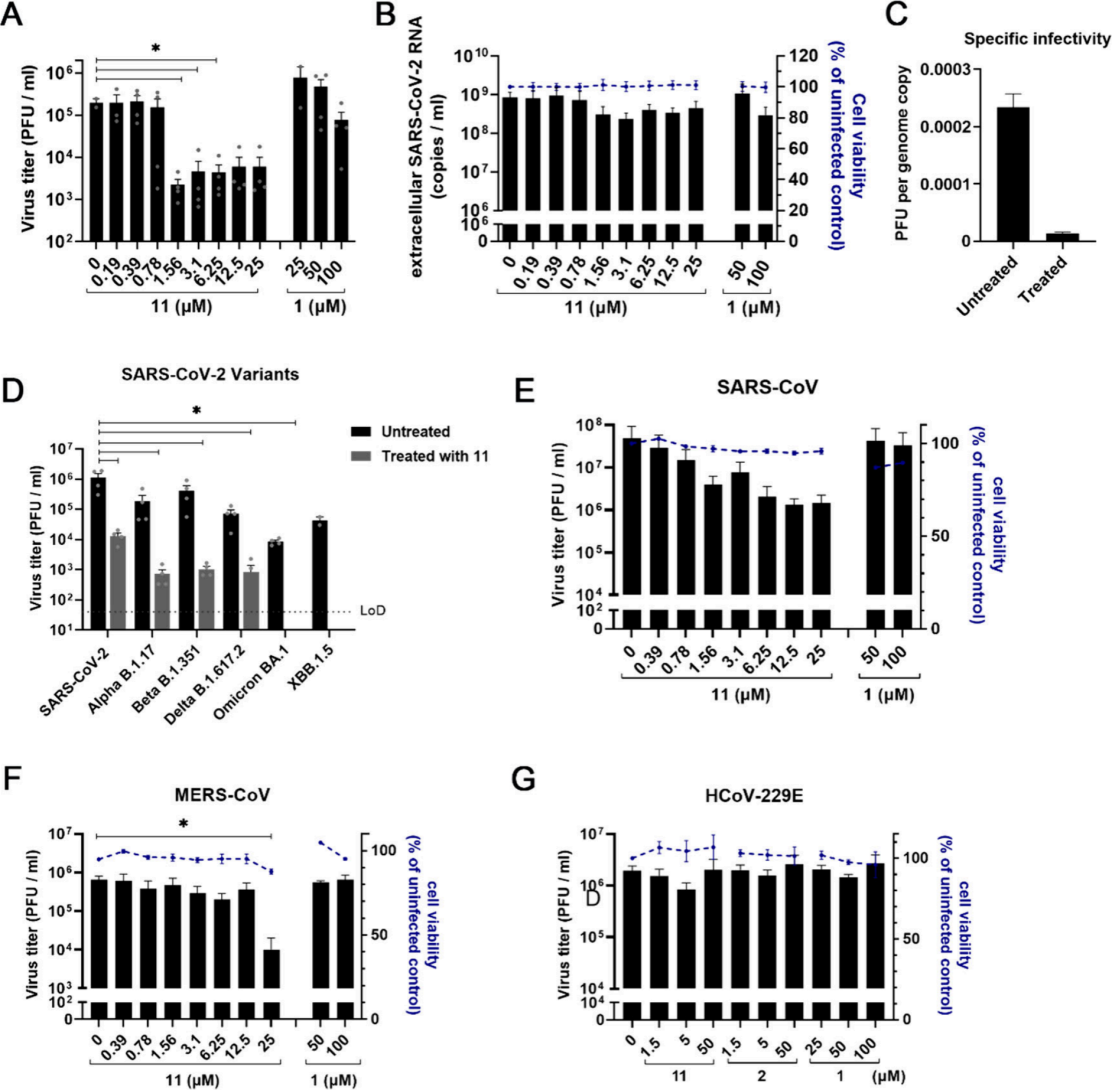
Spectrum of activity of 1,6-*epi*-cyclophellitol cyclosulfate **11** and iminosugars **1** and **2** against various coronaviruses. (A, B) Viral load reduction assay on H1299/ACE2 cells with SARS-CoV-2 (MOI 1) in the presence of compounds **1** or **11**. (A) Infectious virus titer and (B) extracellular viral RNA copy numbers were quantified by plaque assay and RT-qPCR, respectively. Uninfected compound-treated cells were assessed by MTS assay in parallel to measure cytotoxicity of the compounds. *n* = 3 independent experiments. Mean ± SEM values are shown. (C) The specific infectivity of treated (using 1.5 μM of compound **11**) and untreated samples was calculated by dividing the infectious virus titer (PFU/mL) by the viral RNA copy number (copies/mL). Viral load reduction assays with (D) SARS-CoV-2 variants in H1299/ACE2 cells, (E) SARS-CoV in Vero E6 cells, (F) MERS-CoV in HuH-7 cells, and (G) HCoV-229E in H1299/ACE2 cells (all with MOI 1), and treatment with **1**, **2**, or **11**. Supernatant was harvested at 16 hpi to quantify infectious progeny by plaque assay. *n* = 3 independent experiments. Uninfected compound-treated cells were measured by MTS assay in parallel to assess the cytotoxicity of the compounds. Mean ± SEM values are shown. Statistical analysis was conducted using one-way ANOVA, and significant differences are indicated by ∗, *p* < 0.05.

To investigate further the results from the CPE reduction assays, the effect of the most potent glucosidase inhibitor, 1,6-*epi*-cyclophellitol cyclosulfate **11**, was assessed in viral load reduction assays on infected H1299/ACE2 lung epithelial cells. Cells were pretreated with **11** and infected with SARS-CoV-2 at a multiplicity of infection (MOI) of 1. At 16 h postinfection (hpi) supernatant was harvested to quantify the infectious virus titer by plaque assay and extracellular viral RNA copies by RT-qPCR. Treatment of infected H1299/ACE2 lung epithelial cells with **11** resulted in a 100-fold reduction of the infectious progeny virus titer (Figure 3A). The inhibitory effect reached a plateau at 1.6 μM, and higher concentrations of **11** did not lead to more inhibition of virus replication. In contrast, Miglustat **1** reduced infectious progeny production only minimally, even at a concentration as high as 100 μM. Cyclosulfate **11** only slightly reduced extracellular viral RNA copy numbers (Figure 3B), indicating no effect on viral RNA production. This is in line with the expected mechanism of action of the compound that involves viral (structural) protein maturation, likely resulting in reduced infectivity of progeny virus. We then calculated the specific infectivity (defined as the number of infectious particles per viral RNA copy) of treated and untreated samples for the data in Figure 3A,B (Figure 3C). Treatment with compound **11** caused a decrease in specific infectivity, suggesting that the infectivity of released particles is affected. None of the treatments caused noticeable cytotoxicity in uninfected treated cells (Figure 3B). Similarly, treatment of infected Calu-3 lung epithelial cells with **11** reduced infectious progeny virus titers by ∼10-fold, while no reduction in extracellular viral RNA copies was observed (Figure S3).

### 1,6-*epi*-Cyclophellitol Cyclosulfate Inhibits Infectious Progeny of SARS-CoV-2 Variants, SARS-CoV, and MERS-CoV, but Not HCoV-229E

To investigate the spectrum of activity against coronaviruses of 1,6-*epi*-cyclophellitol cyclosulfate **11**, its effect on the replication of SARS-CoV-2 variants Alpha, Beta, Delta, Omicron BA.1, and XBB.1.5 was tested (Figure 3D). As in the above experiments (Figure 3A), viral load reduction assays were performed, during which different cell lines were infected with the respective virus in the presence of compound, and at 16 hpi supernatant was harvested to quantify the infectious virus titer by plaque assay. Similar to the antiviral effect on the early pandemic SARS-CoV-2 isolate, treatment of H1299/ACE2 cells that were infected with other variants showed an ∼100-fold reduction in infectious virus titer (Figure 3D). Viral load reduction assays with SARS-CoV on Vero E6 cells and MERS-CoV on HuH-7 cells showed a significant reduction of infectious progeny upon treatment with increasing concentrations of compound **11** (Figure 3E,F), although the efficacy of the compound was slightly lower against SARS-CoV and clearly lower against MERS-CoV. Interestingly, the viral load reduction assay with HCoV-229E on H1299/ACE2 cells did not show any reduction in virus infectivity, upon treatment with either compound **11** or **2** (Figure 3G).

### 1,6-*epi*-Cyclophellitol Cyclosulfate Strongly Reduces α-Glucosidase Activity and Inhibits SARS-CoV-2 in Primary Human Bronchial Epithelial Cells Cultured at the Air–Liquid Interface

**Figure 4:**
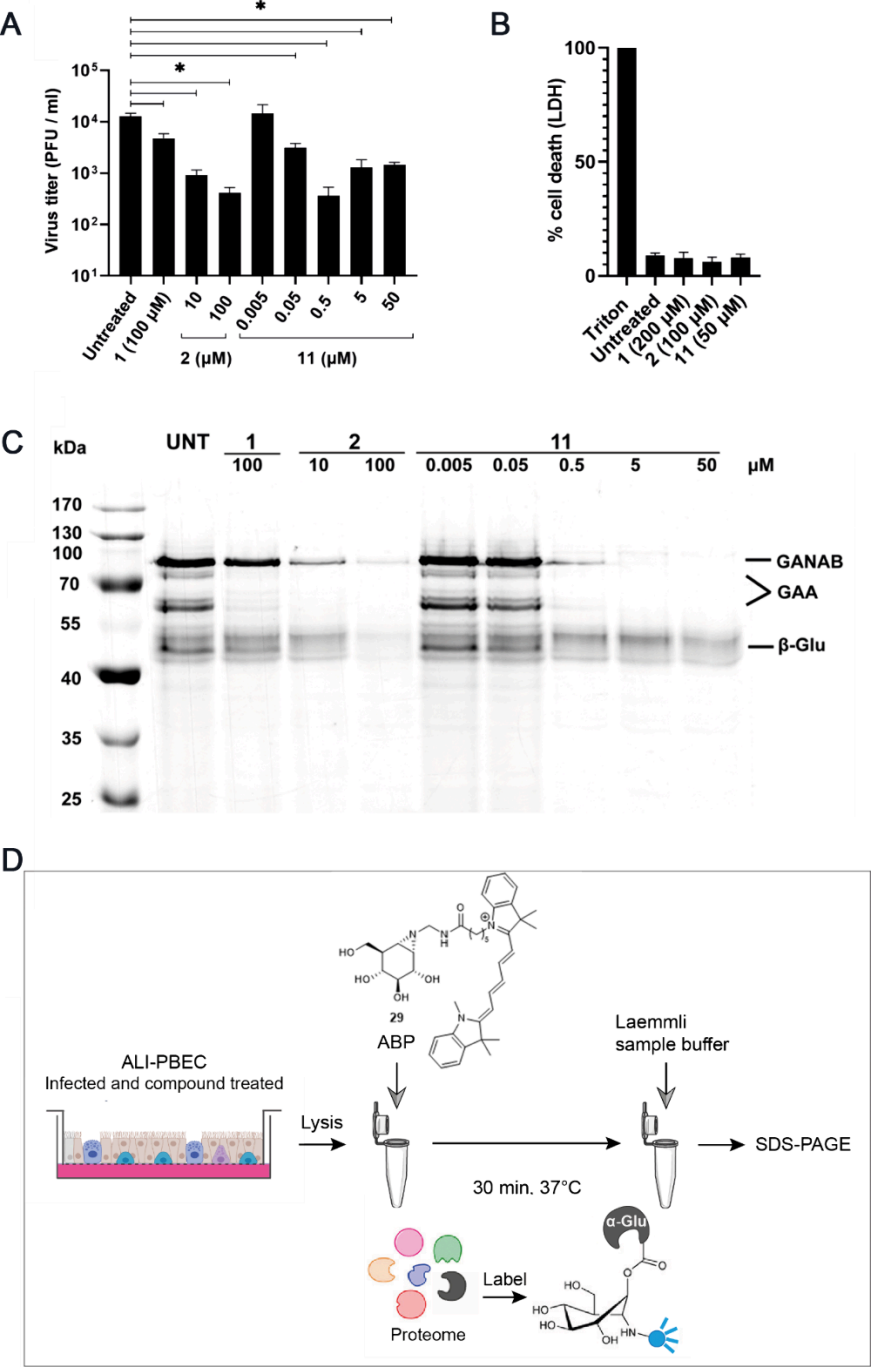
Reduction of SARS-CoV-2 infection in primary bronchial epithelial cells is consistent with inhibition of active ER α-glucosidase II. (A) Viral load reduction assay in ALI-PBEC. Supernatant was harvested at 48 hpi to quantify infectious progeny by plaque assay. *n* = 3 independent experiments. Mean ± SEM values are shown. Statistical analysis was conducted using one-way ANOVA, and significant differences are indicated by ∗, *p* < 0.05. (B) The viability of uninfected compound-treated cells was measured by LDH release assay in parallel, to assess the cytotoxicity of the compounds. Mean ± SEM values are shown. (C) Following compound treatment, cells were lysed and the lysate at pH 7.0 was treated with activity-based probe (ABP) **29** to assess cellular retaining α-glucosidase activities in a competitive activity-based protein profiling experiment. A representative gel of three independent experiments (with two biological replicates/ALI-PBEC inserts each) is shown. (D) Schematic representation of ABP labeling. Part of the figure in (D) was adapted from ref (52), and part was generated using Servier Medical Art, provided by Servier, licensed under a Creative Commons Attribution 3.0 unported license. Figure S4 shows the Gelcode Blue stained gel of (C), which demonstrated that equal amounts of protein were loaded.

We next evaluated the efficacy of 1,6-*epi*-cyclophellitol cyclosulfate **11**, in comparison to our most potent iminosugar, naphthyl-deoxynojirimycin **2**, as well as Miglustat **1** in a more advanced model of primary human bronchial epithelial cells that were cultured at the air–liquid interface (ALI-PBEC), as we described previously.^50,51^ Thus, ALI-PBEC cells were infected with SARS-CoV-2 (10^5^ PFU per insert; estimated MOI ∼0.1) and treated with compounds on the apical side of the cells for 2 h. For uninfected controls, PBS was used instead of virus. The compounds were also present in the basal medium during the whole experiment until 48 hpi, when samples were harvested. Treatment with 0.5 μM compound **11** reduced the viral load significantly by up to 100-fold compared to the untreated control (Figure 4A). Deoxynojiriomycin derivative **2** reduced SARS-CoV-2 to similar titers, but at higher compound concentrations (10 and 100 μM), while Miglustat **1** had only a slight effect at the highest concentration measured (100 μM) (Figure 4A). Measurement of cell death (by LDH release in the supernatant) revealed that none of the compounds tested caused significant cytotoxicity at the highest concentrations (Figure 4B). We also evaluated the reduction of retaining α-glucosidases in the treated ALI-PBEC cell cultures by treatment of the cell lysate at 48 hpi with retaining α-glucosidase activity-based probe **29**, which labels GAA (isoforms at 70 and 76 kDa) and both isoforms of GANAB (∼100 kDa) at pH 7^52^ (Figure 4D). In line with the *in vitro* enzyme activity assay results (Figure 2A), compound **11** was most efficient in inhibiting ER α-Glu II and GAA at low concentrations (Figure 4C and Figure S4), suggesting that *in cellulo* ER α-Glu II inhibition potency correlated well with the efficacy to block SARS-CoV-2 replication.

### 1,6-*epi*-Cyclophellitol Cyclosulfate Inhibits SARS-CoV-2 Replication at a Postentry Step of the Viral Replication Cycle

**Figure 5:**
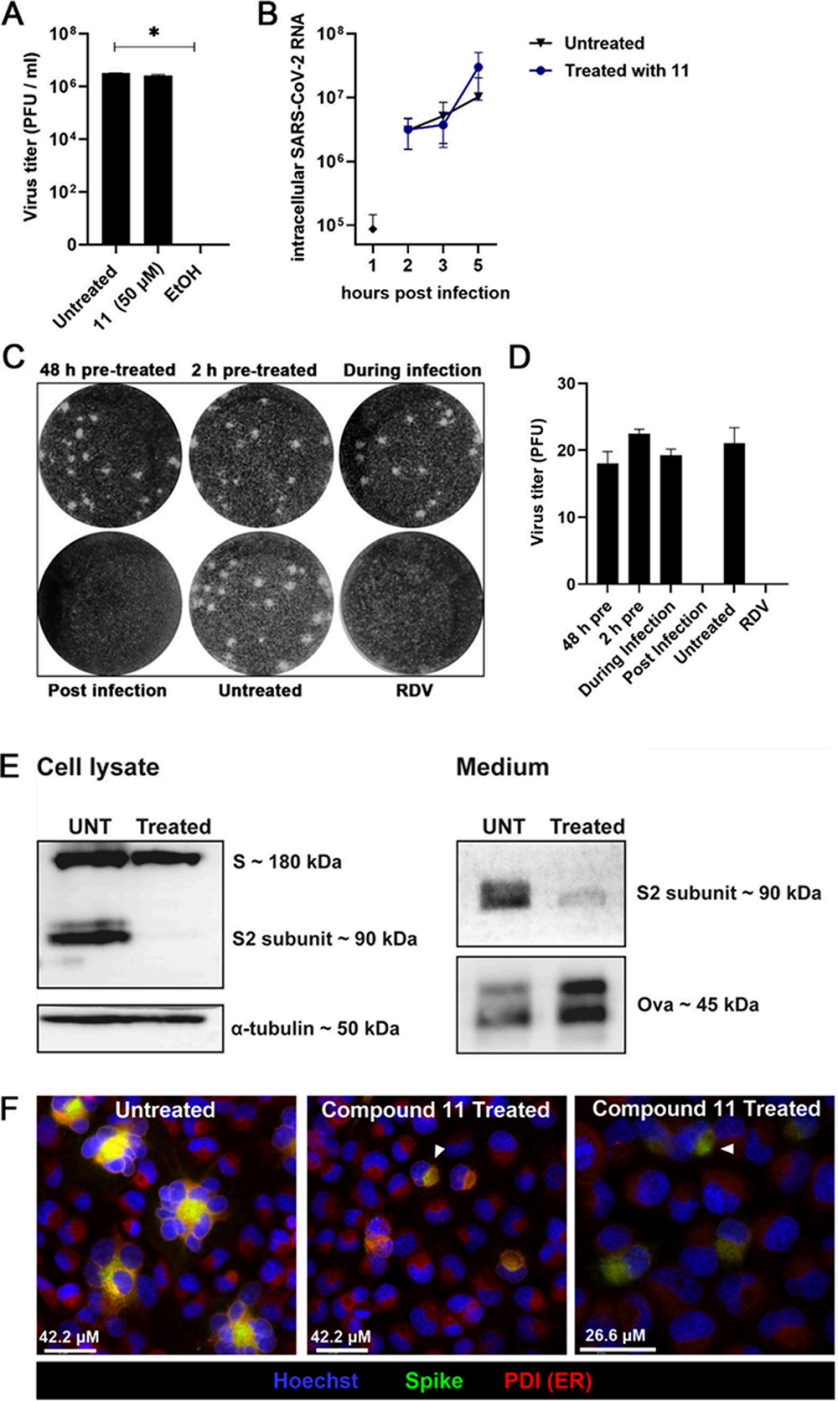
1,6-*epi*-Cyclophellitol cyclosulfate **11** inhibits SARS-CoV-2 replication and syncytium formation by reducing intracellular spike protein levels and processing. (A) Virucidal activity assay in which SARS-CoV-2 was incubated with compound **11** or 70% ethanol (as control) for 1 h at RT, and (remaining) infectious progeny was quantified by plaque assay. *n* = 2 independent experiments. Mean ± SEM values are shown. Statistical analysis was conducted using one-way ANOVA, and significant differences are indicated by ∗, *p* < 0.05. (B) H1299/ACE2 cells were infected with SARS-CoV-2 (MOI 3) and treated with **11** from 1 hpi until harvesting at the indicated time points. Intracellular viral RNA copies were quantified by RT-qPCR. *n* = 3 independent experiments. (C, D) Plaque reduction assay was performed with 1 h infection and incubation for 3 days until cells were fixed and stained with crystal violet. Cells were treated with 5 μM compound **11**, either before infection (pretreatment), during infection, or after infection (postinfection) in the overlay. Treatment with RDV in the overlay was used as a control. *n* = 2 independent experiments. Mean ± SEM values are shown. (E) Western blot analysis of viral S protein in the medium and cell lysates of untreated (UNT) or compound **11** treated (2 μM) H1299/ACE2 cells that were infected with SARS-CoV-2 (MOI 2) and analyzed at 10 hpi using an S2-specific antibody. The medium was spiked with ovalbumin (Ova) as a recovery control and was concentrated before a sample corresponding to ∼250 μL of the original medium volume was analyzed. α-Tubulin was used as a loading control for cell lysates. (F) H1299/ACE2 cells were infected with SARS-CoV-2 (MOI 0.1), fixed at 10 hpi, and the viral S protein and ER marker PDI were visualized by immunofluorescence microscopy. Cells were stained with human anti-SARS-CoV-2 S protein antibody (green), mouse anti-PDI antibody for ER staining (red), and Hoechst for visualizing nuclei (blue). White arrows indicate colocalization of S with PDI. Images are representative of *n* = 2 independent experiments.

We then investigated the mode of action of 1,6-*epi*-cyclophellitol cyclosulfate **11** by assessing which step in the viral replication cycle is inhibited. First, we assessed whether the compound affects the infectivity of virus particles, that is, has virucidal or neutralizing activity. Therefore, SARS-CoV-2 was incubated with a high concentration of compound **11** (50 μM) for 1 h at 37 °C, and subsequently the infectious virus titer was quantified by plaque assay. Control treatment with 70% ethanol led to full inactivation of the virus, while compound **11** had no effect on the infectious titer (Figure 5A). Next, we assessed if treatment early during infection had an effect on virus replication. We infected H1299/ACE2 cells with SARS-CoV-2 at an MOI 3 and started treatment with compound **11** at 1 hpi. At 2, 3, and 5 hpi, cells were harvested and RT-qPCR was performed to quantify the intracellular viral genome copies. The kinetics of intracellular viral RNA accumulation were similar in untreated and compound **11** treated cells, suggesting the compound had no effect on (early) RNA replication (Figure 5B).

To evaluate whether compound **11** has an effect on host proteins (for instance, ACE2) involved in viral entry, we treated monolayers of H1299/ACE2 cells with compound **11** either 48 or 2 h before infection, during infection (0–1 h), or starting from 1 h postinfection (hpi). The cell monolayers were infected with ∼20 PFU of SARS-CoV-2, and after 1 h the inoculum was replaced with an overlay. In one well (Post Infection) the overlay contained compound **11**. Remdesivir, a viral RNA synthesis inhibitor, was added to the overlay of another well, as a positive control for blocking virus replication in the cell. At 3 days postinfection cells were fixed and stained with crystal violet. Pretreatment of the cells with compound **11**, or treatment only during infection, had no effect on the number of plaques that developed or their morphology. Only the presence of compound **11** after infection prevented the formation of plaques, similar to treatment with remdesivir (Figure 5C,D). This result suggests that the antiviral effect of **11** is not through modulating expression or functioning of host proteins (such as the ACE2 receptor) that are essential for viral attachment to, or entry into, the host cell.

### 1,6-*epi*-Cyclophellitol Cyclosulfate Inhibits SARS-CoV-2 Replication through Effects on Intracellular S Protein Maturation and Infectivity of Viral Progeny

From the above-described experiments it became evident that treatment with 1,6-*epi*-cyclophellitol cyclosulfate **11** led to a reduction in virus infectivity, but not to a reduction in the number of viral genome copies (Figure 3), and that inhibition was not through an effect on the receptor or virus binding and entry, but at a postentry step other than RNA replication (Figure 5A–D). Therefore, we suspected an effect on the S protein. As shown in Figure 4, compound **11** effciently inhibited ER α-Glu II, which is crucial for the processing of N-glycosylated viral proteins such as S. To assess the effect of α-Glu II inhibition on S protein production/maturation, we performed viral load reduction assays on H1299/ACE2 cells. Cells were infected with SARS-CoV-2 (MOI 2) and treated with 2 μM compound **11** or cell culture medium. At 10 hpi medium and cell lysate were harvested to analyze S protein levels by Western blotting with an S2-specific antibody. Treatment with compound **11** led to a minor reduction in the amount of full-length S protein in the cell lysate and to the almost complete disappearance of the ∼90 kDa S2 fragment, a product of proteolytic (furin) cleavage of mature S protein in the Golgi apparatus. This indicated that treatment with **11** impaired maturation of the S protein in the ER, leading to reduced trafficking to the Golgi (Figure 5E). The amount of (processed) S2 was also strongly reduced in the medium of compound-treated cells, suggesting the compound impaired biogenesis of particles or their S protein content (Figure 5E).

Next, we set out to analyze the effect of compound **11** treatment on the level and localization of the S protein in infected cells and on the formation of syncytia, which are large multinucleated cells resulting from the interaction of S protein on the surface of infected cells with ACE2 receptors on neighboring cells, which triggers cell fusion. To this end, SARS-CoV-2-infected H1299/ACE2 cells (MOI 0.1) were treated with 5 μM compound **11** or cell culture medium as control, and at 10 hpi cells were fixed and analyzed by immunofluorescence staining for the viral S protein and the ER marker protein disulfide isomerase (Figure 5F). We observed a reduction in the amount of S protein in infected cells that were treated with compound **11** and the colocalization of S protein with the ER marker, which suggests (partial) retention of S proteins in the ER. Treatment also led to reduced syncytium formation compared to untreated infected cells, likely due to impaired maturation, and subsequent impaired trafficking of S protein to the plasma membrane.

## Discussion

In this study we have assessed the ER α-Glu II inhibitory potency and anti-SARS-CoV-2 activity of selected members (28 compounds in total) of two classes of glycomimetics—iminosugars and cyclitol analogues—and to what extent these two effects correlate. Deoxynojirimycin-type iminosugars as competitive inhibitors have been studied for almost four decades as candidate antivirals for pathogenic viruses that rely on ER-protein quality control and in recent years have also been explored as anti-SARS-CoV-2 agents.^14,15,18,24,53,54^ In contrast, cyclophellitol-type mechanism-based inhibitors have not been considered for this purpose. The results described here support the hypothesis that mechanism-based inactivation of ER α-Glu II may lead to effective new antiviral agents to treat infections with the numerous viruses that rely on host protein glycosylation for replication. In particular, 1,6-*epi*-cyclocyclosulfate **11**, the most potent ER α-Glu II inhibitor of the tested compounds, also blocked viral replication most effectively. Although 0.5–1.6 μM doses of compound **11** reduced infectious virus titers up to 2 logs in Calu-3 cells and ALI-PBEC, higher concentrations did not lead to a further reduction and complete inhibition of virus replication was not observed at high doses. In ALI-PBEC the maximum antiviral effect was already reached at 0.5 μM, a concentration at which an almost full inhibition of ER α-Glu II was observed, suggesting that the remaining virus replication was not due to incomplete inhibition of this enzyme. Further investigations revealed that the antiviral effect is not due to effects on (glycosylation or quantity of) host cell factors that play a role in virus binding and entry into the host cell, or replication of the viral genome, suggesting it does not (noticeably) target the SARS-CoV-2 nonstructural proteins. The antiviral effect is on blocking N-glycosylation of the S protein, the most heavily N-glycosylated SARS-CoV-2 protein, which plays crucial roles in virus binding and entry. The absence of cleaved S2 fragment in compound treated cells indicates that impairing processing of S protein at the ER led to reduced trafficking of S to the Golgi and prevention of (furin) cleavage of the S1/S2 site, ultimately leading to less mature S protein for incorporation into infectious virus particles. Thus, cyclosulfate **11** acts on protein N-glycosylation/ER protein quality control, just as the *N*-alkyl deoxynojirimycin derivatives tested by us and others, but in addition compound **11** is much more selective compared to the iminosugars.^35^ Considering the mechanistic mode of action of inverting and retaining glucosidases, compound **11** inhibits retaining α-glucosidases exclusively over inverting α-glucosidases, within the context of this work the lysosomal retaining α-glucosidase, GAA, as the single off-target.

Deoxynojirimycin-type iminosugars in contrast also block inverting α-glucosidases including ER α-Glu I. The finding that blocking ER α-Glu II alone is sufficient (at least in the assays reported here) for halting SARS-CoV-2 replication may therefore be beneficial for situations in which ER inhibiting α-Glu I has adverse effects. Iminosugars have often also other human glycoprocessing enzymes as off-target. *N*-Butyldeoxynojirimycin **1** (Miglustat) is applied in the clinic for the treatment of Gaucher disease, where it acts as a glucosylceramide synthase inhibitor.^44,55^ It also inhibits the three human retaining β-glucosidases, GBA1, GBA2, and GBA3.^56^ None of these enzymes plays a role in SARS-CoV-2 infections, and their inhibition may lead to adverse effects as well. Such adverse effects in contrast are not to be expected from 1,6-*epi*-cyclophellitol cyclosulfate **11**, which does not inhibit any of these enzymes (GCS, GBA1, GBA2, GBA3) as we have shown before.^35^ Arguably, adverse effects such as elicited by **11** may be the result of inhibition of the lysosomal α-glucosidase, GAA; however, this enzyme is also inhibited by the iminosugars.^57^ We therefore conclude that compound **11**, which in contrast to the iminosugars is nonbasic, thus not charged at physiological conditions, may be a good starting point for the development of new antiviral agents for the treatment of infections by SARS-CoV-2 and other (emerging) viruses that require ER-protein quality control for replication.

## Methods

### Compounds and Cell Lines

Inhibitors were synthesized at the bio-organic synthesis group at the Leiden Institute of Chemistry. The synthesis of the cyclitol and iminosugar inhibitors **9**–**11**, **17**, **18**–**22**, **25**, **26**, and **1**–**8** have been published previously.^35,41−43,45,47^ The syntheses of methylated sulfates **12**–**16** and alkyl aziridines **23**, **24**, **27** and **28** can be found in the Supporting Information (Schemes S1–S5). Lyophilized compounds were diluted in DMSO prior to use. Remdesivir, which was used as the compound control in different assays, was purchased from Sigma-Aldrich and dissolved in DMSO. UV-4 (SP187) was purchased from MedChemExpress and dissolved in DMSO.

Vero E6 cells and HuH-7 cells were cultured as previously described.^58^ Human lung cell line H1299/ACE2 is described elsewhere.^59^ These cells were cultured in Dulbecco’s modified Eagle’s medium with 4.5 g/L glucose with l-glutamine (DMEM; Lonza, Basel, Switzerland) supplemented with 10% fetal calf serum (FCS; CapriCorn Scientific, Ebsdorfergrund, Germany), 100 U/mL penicillin/streptomycin (P/S; Sigma-Aldrich, St. Louis, MO, USA), and 1200 μg/mL G418 for selection (InvivoGen, San Diego, CA, USA). Infections of Vero E6 cells, HuH-7 cells, and H1299/ACE2 cells were performed in Eagle’s minimal essential medium with 25 mM HEPES (EMEM; Lonza) supplemented with 2% FCS, 2 mM l-glutamine (Sigma-Aldrich), and 100 U/mL P/S. Primary human bronchial epithelial cells (PBEC) were isolated and cultured as previously described.^60^ All cell cultures were maintained at 37 °C in an atmosphere of 5% CO_2_.

### Virus Stocks

All experiments with infectious SARS-CoV, SARS-CoV-2, or MERS-CoV were performed at the LUMC biosafety level 3 facilities. The clinical isolate SARS-CoV-2/Leiden-0008 (isolated at LUMC during the first wave of the Corona pandemic in March 2020 (GenBank: MT705206.1) was used for H1299/ACE2 and ALI-PBEC infections. This virus stock was not adapted to Vero E6 cells with regard to the spike S1/S2 cleavage site (confirmed by NGS). For CPE assays in Vero E6 cells SARS-CoV-2/Leiden0002 was used (GenBank: MT510999.1). SARS-CoV-2 variant B.1.1.7 (Alpha), variant B.1.351 (Beta), and variant B.1.617 (Delta) were obtained from the University of Leuven. SARS-CoV-2 variant BA.1 (Omicron) was obtained from RIVM (strain hCoV-19/Netherlands/NH-RIVM-72291/2021, lineage B.1.1.529, GenBank: OR427989.1), and variant XBB.1.5 was isolated from a patient sample at LUMC. SARS-CoV-2/Leiden-0008 (Passage 2), SARS-CoV-2/Leiden0002, and SARS-CoV isolate Frankfurt 1^61^ (Passage 4) were grown on Vero E6 cells. Αlpha (Passage 4), Beta (Passage 4), Delta (Passage 4), Omicron BA.1, and XBB.1.5 (P3) variants were grown on Calu-3 cells. MERS-CoV (N3/Jordan) (GenBank: KJ614529.1) (Passage 3) and HCoV-229E were grown on HuH-7 cells. Virus titers were determined by plaque assay on Vero E6 cells, and for MERS-CoV and HCoV-229E on HuH-7 cells, as described before.^62^

### *In Vitro* GAA and GANAB Enzyme Activity Assay

Inhibition of the enzymes GAA and GANAB by the compounds was tested *in vitro* as described previously.^35^ Briefly, enzymes were preincubated with a range of inhibitor concentrations for 30 min at 37 °C. The residual activities of the enzymes were then measured by adding the 4-MU–Glc substrate mixture at their corresponding optimal pHs. Reactions were quenched with 1 M NaOH–glycine (pH 10.3) upon completion, and 4-MU fluorescence was measured with an LS55 fluorescence spectrophotometer (PerkinElmer) (λ_EX_ 366 nm; λ_EM_ 445 nm). IC_50_ values reported are the mean values from three technical replicates.

### Cytopathic Effect (CPE) Reduction Assay

CPE reduction assays were performed as previously described.^58^ Briefly, Vero E6 cells were seeded in 96-well plates at a density of 5 × 10^3^ cells/well. The next day, cells were infected with SARS-CoV-2/Leiden0002 in the presence of 2-fold serial dilutions of compound. Four days postinfection the CellTiter 96 aqueous nonradioactive cell proliferation kit (Promega) was used to measure the cell viability of infected (protection) and noninfected cells (assessment of cytotoxicity). EC_50_ values reported are the mean values from three independent experiments and were calculated using GraphPad Prism 6.

### Expression of *Mm*α-Glu-II

The two subunits of *M. musculus* α-glucosidase II ganab and prkcsh were subcloned into separate vectors (pOPING and pOPINGS for ganab and prkcsh respectively) and codon optimized for mammalian expression by Genscript. Each vector was transformed into DH5α (Thermo Fisher) cells by heat shock. Cultures of each subunit were grown at 37 °C in LB, and the amplified DNA was purified using the PureLink HiPure plasmid filter Maxiprep kit (Invitrogen) obtaining 750 μg of DNA for both constructs. The isolated DNA was cotransfected into a 600 mL suspension of 293-F cells following the Freestyle 293 Expression system protocol (Thermo Fisher) and harvested after 4 days at 37 °C, 8% CO_2_, at 135 rpm.

### Purification of ER α-Glu-II

Cells were pelleted at 200*g*, for 20 min at 4 °C, and the clarified media was then further centrifuged for 20 min, at 5000*g* at 4 °C. The clarified media was loaded onto a pre-equilibrated 5 mL HisTrap excel column (Cytiva) with binding buffer (1× PBS, 20 mM imidazole, 5% glycerol w/v) and eluted using a buffer gradient 0–100% of elution buffer (1× PBS, 500 mM imidazole, 5% glycerol w/v) over 20 CVs. Fractions containing *Mm*α-Glu-II were concentrated and loaded onto a size exclusion S200 column (Cytiva), which was pre-equilibrated with HEPES buffer (20 mM HEPES pH 7.5 and 150 mM NaCl). The *Mm*α-Glu-II containing fractions were pooled, and a trypsinolysis was performed using sequencing grade modified trypsin (Promega), supplemented with 2 mM CaCl_2_ for 4 h at a ratio of 1:100 (trypsin:*Mm*α-Glu-II). The size exclusion was repeated, and the resulting *Mm*α-Glu-II was pooled and concentrated to 8 mg/mL.

### Thermal Shift Assays

Triplicate reactions of 10 μM *Mm*α-Glu-II unliganded control and 10 μM *Mm*α-Glu-II with 50 μM inhibitor were prepared to a final volume of 30 μL with buffer (20 mM HEPES pH 7.5 and 150 mM NaCl). Before the assay, 20× SYPRO orange dye was added to each reaction mixture. The assay was performed using the Stratagene Mx3005P qPCR machine, where the SYPRO orange dye was excited at λ_EX_ 517 nm and monitored at 585 nm with 2 °C min^–1^ increases from 25 to 95 °C. Readings were averaged to produce a thermal stability curve with fluorescence plotted against temperature and the *T*_m_ estimated from the midpoint.

### Viral Load Reduction Assays

For SARS-CoV-2 (variants) and HCoV-229E infections, H1299/ACE2 cells were seeded in 96-well plates at a density of 10^4^ cells/well and the next day infected at MOI 1. Infections with SARS-CoV-2 were incubated at 37 °C, and infections with HCoV-229E were incubated at 33 °C. For SARS-CoV or MERS-CoV infections (MOI 1), Vero E6 or HuH-7 cells were seeded in 96-well plates at a density of 2 × 10^4^ cells/well. Cells were incubated at 37 °C. After removal of the inoculum at 1 hpi, cells were washed three times with warm PBS or medium, after which they were incubated in infection medium (EMEM). Supernatant samples were harvested at 16 hpi, and infectious virus titers were determined by plaque assay and viral RNA copy numbers by RT-qPCR. In parallel, the cytotoxicity of compound treatment was measured on uninfected cells by the CellTiter 96 aqueous nonradioactive cell proliferation kit.

### Immunofluorescence Staining

For immunofluorescence imaging of viral spike protein, H1299/ACE2 cells were seeded onto glass coverslips in 24-well plates at a density of 1.6 × 10^5^ cells/well. The next day they were infected with SARS-CoV-2/Leiden0008 (MOI 0.1) in Opti-MEM reduced serum medium (Thermo Fisher Scientific). At 16 hpi, cells were fixed with 3% warm paraformaldehyde. Immunofluorescent staining of viral spike protein was done using human antispike antibody P52 (gift from King’s College) and goat-α-human IgG Alexa 488 antibody (Thermo Fisher Scientific). Staining of endoplasmic reticulum was done using mouse anti-PDI antibody (Fuller)^63^ and donkey-α-mouse Cy3 antibody (Jackson).

### Western Blot

For Western blot analysis, H1299/ACE2 cells were seeded in 6-well plates at a density of 6.5 × 10^5^ cells/well and the next day infected with SARS-CoV-2/Leiden0008 at an MOI of 2. At 10 hpi supernatant was harvested and 4000 μL of medium was spiked with ovalbumin (internal recovery control) and concentrated to 150 μL using Amicon Ultra-0.5 centrifugal filter units (Merck), according to the manufacturer’s instruction. An equal amount of Laemmli buffer was added, and samples were heated at 95 °C for 5 min. Samples were analyzed by SDS-PAGE (10% gel, 30 min at 90 V, then 50 min at 120 V) and subsequently blotted for 30 min in a semidry blotting system (Bio-Rad). The membrane was blocked with 1% casein in PBST for 1 h at RT, before incubation with primary antibodies overnight at 4 °C. Spike proteins were detected using SARS/SARS-CoV-2 spike protein S2-specific mAb 1A9 (Invitrogen) as the primary antibody. The loading control tubulin was detected with mouse-anti-α-tubulin antibody B-5-1-2 (abcam), and spiked ovalbumin was detected with mouse ovalbumin mAb 1D3D5 (Thermo Fisher). The next day the membrane was washed three times for 5 min with PBST and then incubated in 0.5% casein in PBST with a secondary goat-α-mouse-HRP antibody (P0447, Dako) for 1 h at RT. After washing again three times, the membrane was incubated in Clarity Western ECL Substrate (Bio-Rad) for 2 min and imaged with the Uvitec Alliance Q9 advanced imager.

### RNA Isolation and RT-qPCR

RNA was isolated by magnetic bead isolation, as described in ref (51). Equine arteritis virus (EAV) in AVL lysis buffer (Qiagen) was spiked into the isolation reagent as an internal control for extracellular RNA samples. RT-qPCR was performed using TaqMan Fast Virus 1-step master mix (Thermo Fisher Scientific) and as previously described.^64^ The cellular reference gene PGK1 served as a control for intracellular RNA. Primers and probes for EAV and PGK1 and the normalization procedure were described before.^62^ Primers and probes for SARS-CoV-2, as well as a standard curve, were used as described previously.^64,65^

### Plaque Assay

To quantify infectious virus titers, plaque assays were done on Vero E6 cells (SARS-CoV-2 and variants, SARS-CoV), H1299/ACE2 (HCoV-229E), or HuH-7 (MERS-CoV). For SARS-CoV-2 and variants, 2 × 10^4^ cells/well were seeded in a 96-well plate, and serial dilutions of samples were inoculated for 1 h at 37 °C on a rocking platform. Inoculums were removed and 100 μL of methylcellulose overlay was added. Cells were incubated for 4 days until fixation and crystal violet staining. Alternatively, plaque assays for SARS-CoV-2 (variants) were done in 6-well plates, with Avicel overlay and 3 days of incubation. HCoV-229E samples were quantified in 12-well plates, using Avicel overlay and incubating for 4 days. MERS-CoV samples were quantified in 12-well plates with Avicel overlay or 96-well plates with methylcellulose overlay for 3 days.

### Infection of ALI-PBEC and Activity-Based Probe Labeling

ALI-PBEC were pretreated with compound in the basal medium for 3 h. Cells were infected with 100 000 PFU of SARS-CoV-2/Leiden0008 per insert (estimated MOI 0.1) with compounds present in the inoculum. After 2 h at 37 °C on a rocking platform, the inoculum was removed and cells were washed three times with warm PBS. Compounds stayed present in the basal medium until 48 h postinfection. At 48 hpi the viral load was determined by plaque assay on a 200 μL apical wash (PBS incubated on the apical side of the insets for 10 min at 37 °C). For assessing cytotoxicity with the CyQuant LDH cytotoxicity assay (Thermo Fisher Scientific), 10 μL of apical wash was diluted 5 times with 40 μL of PBS. A 25 μL volume of this dilution was added to 25 μL of assay reagent and incubated for 30 min at RT in the dark. The plate was fixed and measured at a wavelength of 490 nM (Envision reader, PerkinElmer). For the activity-based probe labeling, the insets were washed one more time with PBS and processed as described previously.^52^ Briefly, cells were lysed with 60 μL of potassium phosphate buffer per insert. A fluorescently labeled probe (JJB383) was diluted in MclIvaine buffer (pH 7) to a 10 μM stock and incubated for 5 min on ice. For labeling of the cell lysate, 10 μL of lysate was added to 10 μL of MclIvaine buffer and 5 μL of probe. The lysate was incubated for 30 min at 37 °C before the addition of 10 μL of Laemmli sample buffer (4×). Samples were heated at 95 °C for 5 min and separated in a 10% SDS-PAGE gel. Fluorescence was measured at a wavelength of 625 nm (Cy5) with a Uvitec Alliance Q9 imager (BioSPX). After imaging, the gels were stained with GelCode Blue stain reagent (Thermo Fisher Scientific) and visualized using a Uvitec Essential V6 system to check for equal loading.

### Plaque Reduction Assay

H1299/ACE2 cells were seeded in a 6-well plate at a density of 1.3 × 10^5^ cells/well (20% confluency), 96 h prior to infection. Cells were treated with 5 μM compound **11** either 48 or 2 h before infection, or during the 1 h infection in the inoculum. The monolayers were infected with ∼20 PFU of SARS-CoV-2/Leiden0008. In the postinfection treatment, the compound (or RDV) was added to the Avicel overlay. Cells were incubated for 4 days at 37 °C before fixation and crystal violet staining.

## Supporting Information Available

Additional experimental details including synthesis of the methylated sulfamidates **12**–**16** and the alkyl aziridines **23**, **24**, **27**, and **28**; additional results including SARS-CoV-2 viral load reduction assay on Calu-3 lung epithelial cells, SARS-CoV viral load reduction assay on Vero E6 cells, GelCode Blue staining of SDS-PAGE gel of activity-based protein profiling experiment, SDS-PAGE gel of activity-based protein profiling at pH 4 (PDF)
