# Supplementary material for: Epi-Cyclophellitol Cyclosulfate, a Mechanism-Based Endoplasmic Reticulum α-Glucosidase II Inhibitor, Blocks Replication of SARS-CoV-2 and Other Coronaviruses
Source: ACS Cent Sci. 2024 Jul 25;10(8):1594–608. doi: 10.1021/acscentsci.4c00506 (PMC11363342; doi:10.1021/acscentsci.4c00506)
Supplement: Supplementary file 1 — oc4c00506_si_001.pdf [file oc4c00506_si_001.pdf]

Supplementary information

Epi-cyclophellitol cyclosulfate, a mechanism-based ER α-glucosidase II inhibitor, blocks replication of SARS-CoV-2 and other coronaviruses

Melissa Thaler[a], Tim P. Ofman[b], Ken Kok[b], Jurriaan J.A. Heming[b], Elisha Moran[c], Isabelle Pickles[c], Anouk A. Leijs[a], Adrianus M. C. H. van den Nieuwendijk[b], Richard J. B. H. N. van den Berg[b], Gijs Ruijgrok[b], Zach Armstrong[b], Clarisse Salgado-Benvindo[a], Dennis K. Ninaber[d], Eric J. Snijder[a], Constant A. A. van Boeckel[b], Marta Artola[b], Gideon J. Davies[c], Herman S. Overkleeft[b#], Martijn J. van Hemert[a#]

[a]Leiden University Center for Infectious Diseases (LUCID), Leiden University Medical Center, Leiden, The Netherlands.

[b]Leiden Institute of Chemistry, Leiden University, Leiden, The Netherlands.

[c]Department of Chemistry, University of York, YO10 5DD, York, United Kingdom.

[d]Department of Pulmonology, Leiden University Medical Center, Leiden, The Netherlands.

Running Head: Mechanism-based ER α-Glu-II inhibitor blocks coronavirus infection

#Address correspondence to:

Martijn J. van Hemert, m.j.van_hemert@lumc.nl or

Herman S. Overkleeft, h.s.overkleeft@chem.leidenuniv.nl

Table of Contents

Supplementary Figures **2**

Supporting synthesis schemes **4**

Biochemical methods **7**

General experimental procedures **16**

Synthetic procedures **16**

NMR Data; spectra of new and selected compounds **28**

References **86**

Supplementary Figures

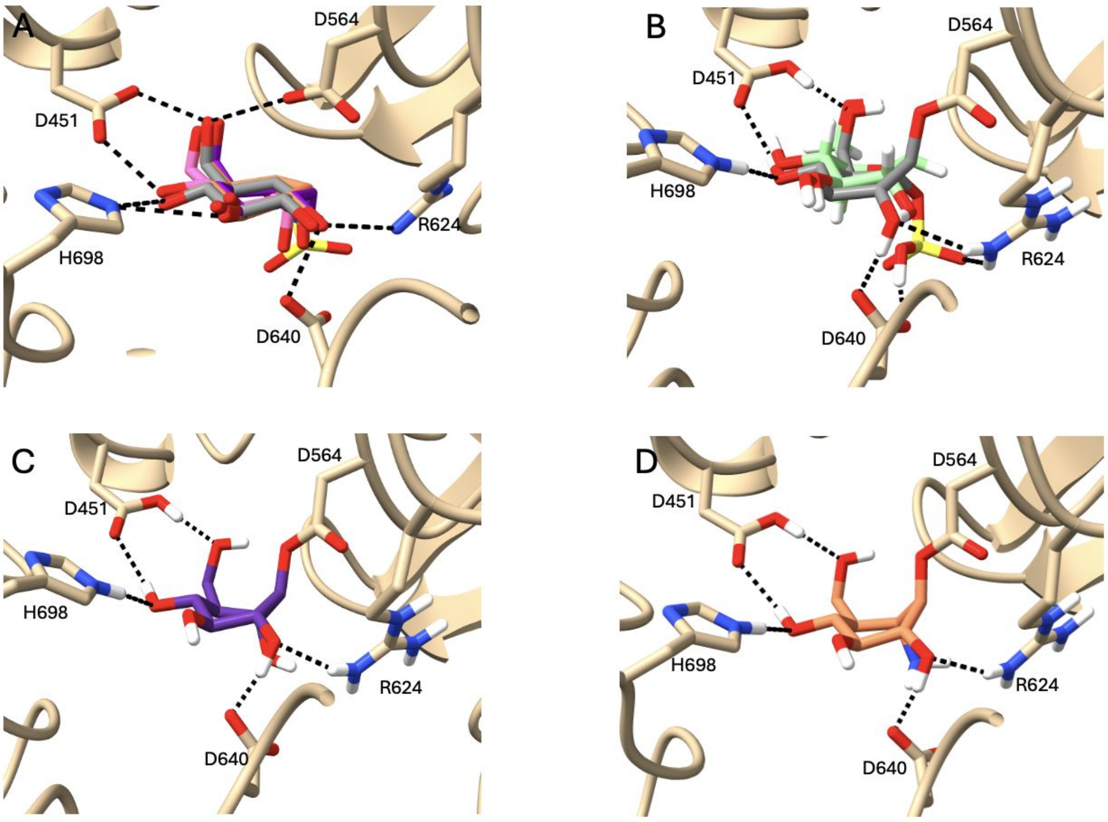

Figure S1: Docking of 1,6-cyclitols compounds into the α-glucosidase II active site (PDB 5H9O).
(A) Overlay of the top non-covalent dockings of compounds 10 (orange), 9 (purple) and 11 (grey) with glucose (pink PDB: 5H9O). Black lines indicating H-bond interactions with active site residues, including catalytic nucleophile D564.
(B) Top covalent docking for compound 11 (grey), the covalent bond formed between nucleophile D564 along with H-bond interactions and overlaid with covalent 5F-Glc (PDB: 5HJR, green).
(C) Top covalent docking of compound 9 covalently bound to catalytic nucleophile D564 along with H-bonds interactions (black dashes).
(D) Top covalent docking of compound 10 covalently bound to catalytic nucleophile D564 along with H-bonds interactions (black dashes).

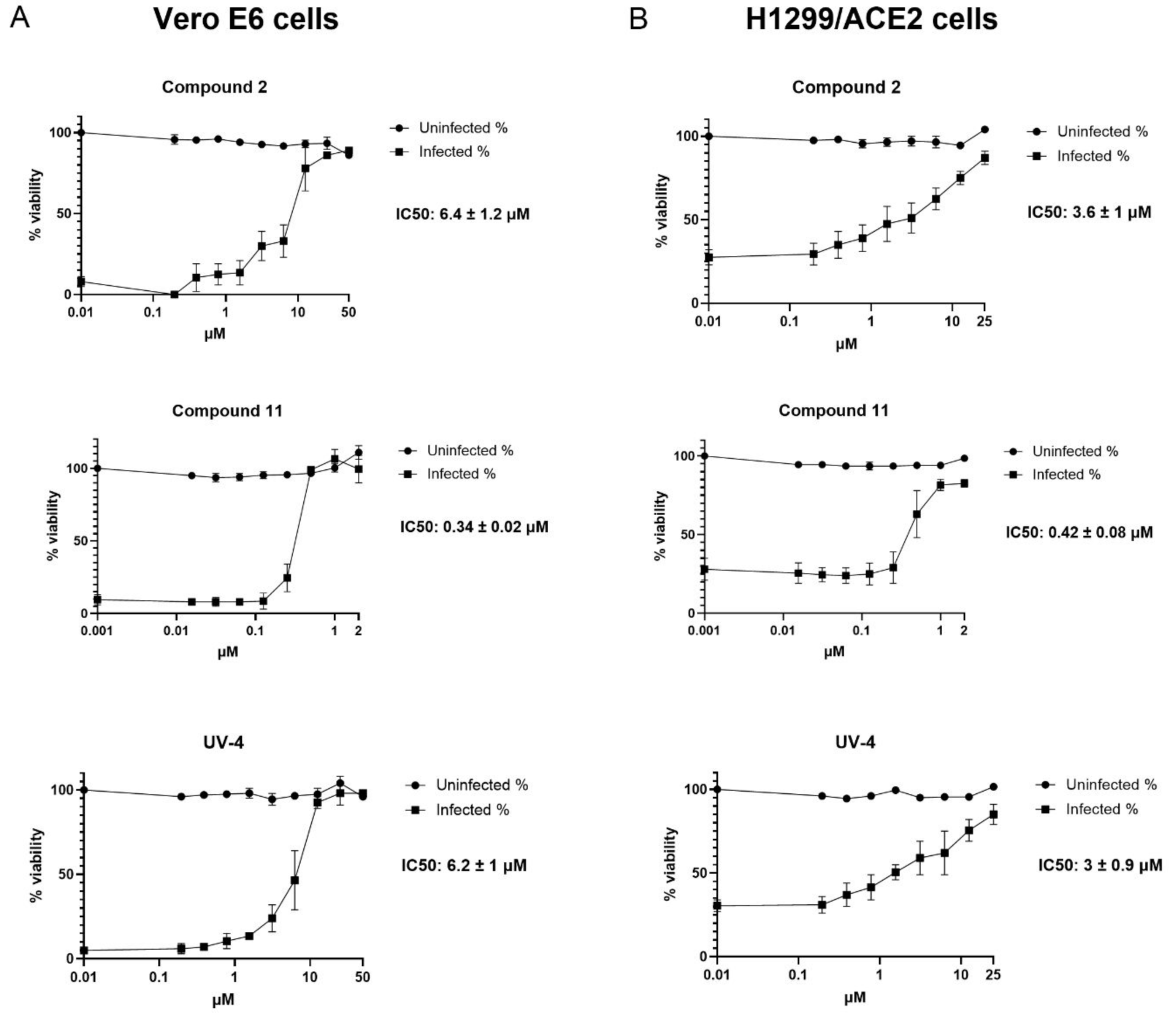

Figure S2: Cytopathic effect reduction assays on Vero E6 monkey kidney cells and H1299/ACE2 human lung epithelial cells. (A) SARS-CoV-2 CPE assay dose-response curves of naphthyl-deoxynojirimycin **2**, cyclosulfate **11**, and iminosugar **UV4** on (A) Vero E6 cells, and (B) H1299/ACE2 cells. n=2 independent experiments. The viability of uninfected compound-treated cells was established by MTS assay in parallel. Means ± SEM are shown. The 50% inhibitory concentration (EC_{50}) values were determined by non-linear regression with GraphPad Prism 6. Methods for the CPE assay on H1299/ACE2 cells can be found in this supplementary material.

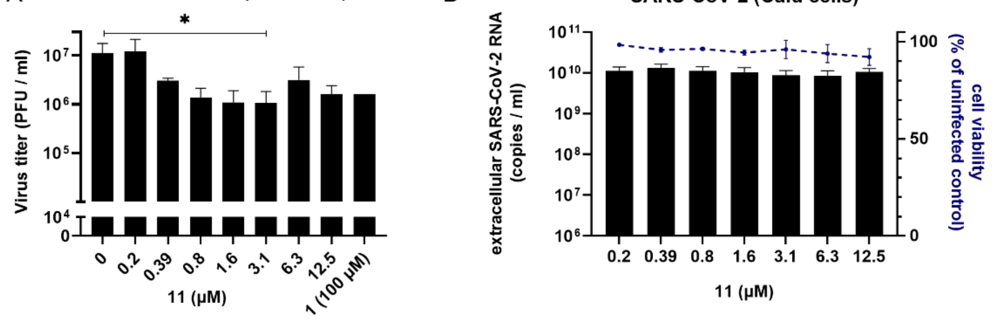

Figure S3: 1,6-*epi*-cyclophellitol cyclosulfate inhibits SARS-CoV-2 infectious progeny production in Calu-3 lung epithelial cells. (A-B) Viral load reduction assay in Calu-3 cells with SARS-CoV-2 (MOI 1) and samples harvested at 16 hpi. (A) Infectious virus titer and (B) extracellular viral RNA copy numbers were quantified by

plaque assay and RT-qPCR, respectively. Uninfected compound-treated cells were assessed by MTS assay in parallel to measure cytotoxicity of the compounds. n=3 independent experiments. Mean ± SEM are shown.

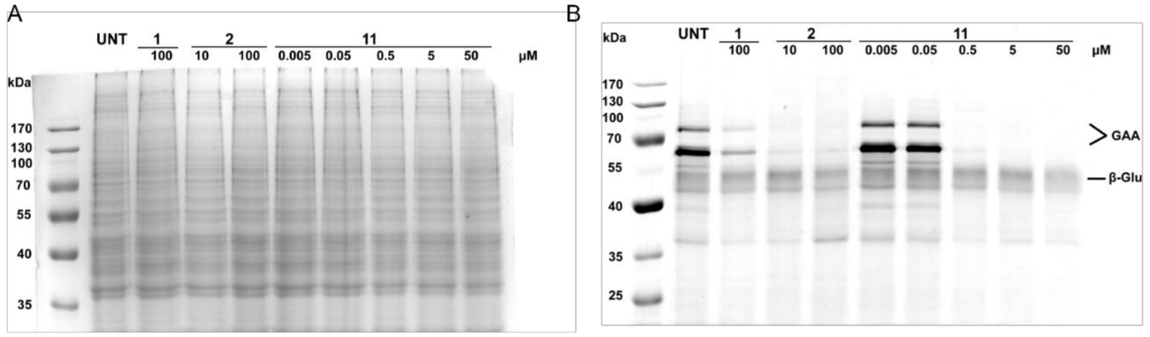

Figure S4: Activity-based probe labelling of cell lysate from SARS-CoV-2 infected ALI-PBEC that were treated with compound **11**, **1** and **2**. (A) performed at pH7. GelCode Blue staining of SDS-PAGE to visualize the total protein amount that was loaded. Gel was washed three times for 5 minutes with deionized water, stained over night with GelCode Blue staining reagent, and washed again three times, before imaging using a Uvitec Essential V6 system. (B) performed at pH4.

Supporting synthesis schemes

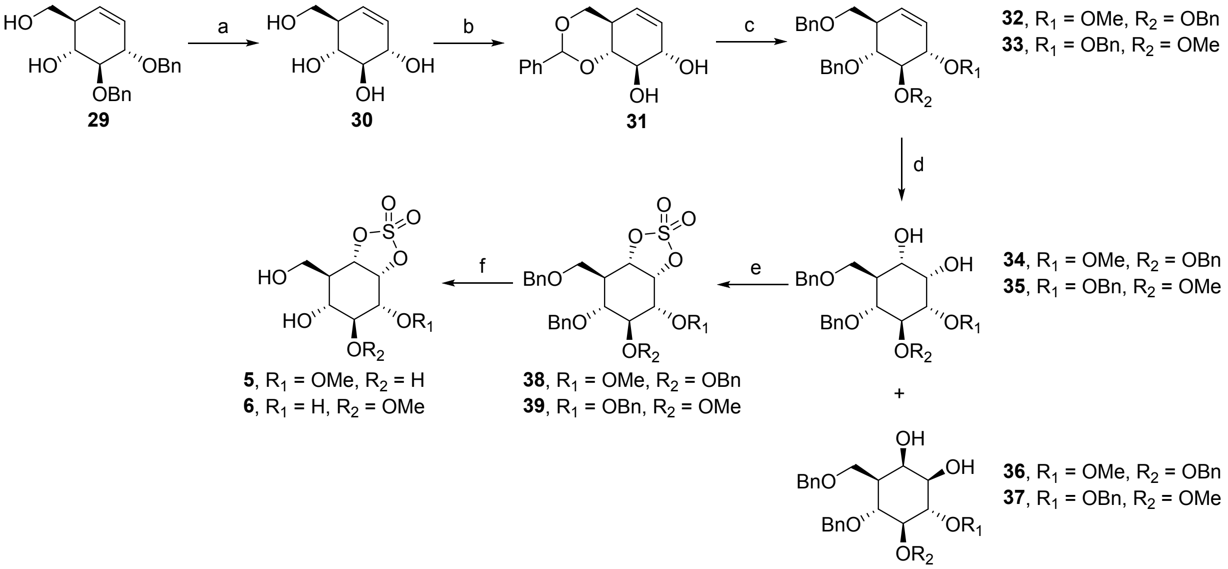

Scheme S1. Preparation of target compounds **5** and **6**. Reagents and conditions: (a) BCl_3, DCM, -78 °C, 4 h, 89% (b) Benzaldehyde dimethyl acetal, *p*-TsOH, DMF/MeCN, 4 h, 73% (c) i: KI, K_2CO_3, 2-aminoethyl diphenylborinate, alkyl halide, MeCN, 18 h ii: TFA, H_2O, DCM, 1.5 h, iii: alkyl halide, TBAI, 60% NaH, DMF, 2 h, 36% (**32**), 31% (**33**) (d) $RuCl_3 \cdot H_2O$, $NaIO_4$, EtOAc/H_2O/MeCN, (e) i: $SOCl_2$, Et_3N, DCM, ii: $RuCl_3 \cdot H_2O$, $NaIO_4$, EtOAc/H_2O/MeCN, 46% (**38**), 40% (**39**) over 3 steps (f) Pd/C, H_2, MeOH/THF, 4 h, 91% (**5**), 93% (**6**).

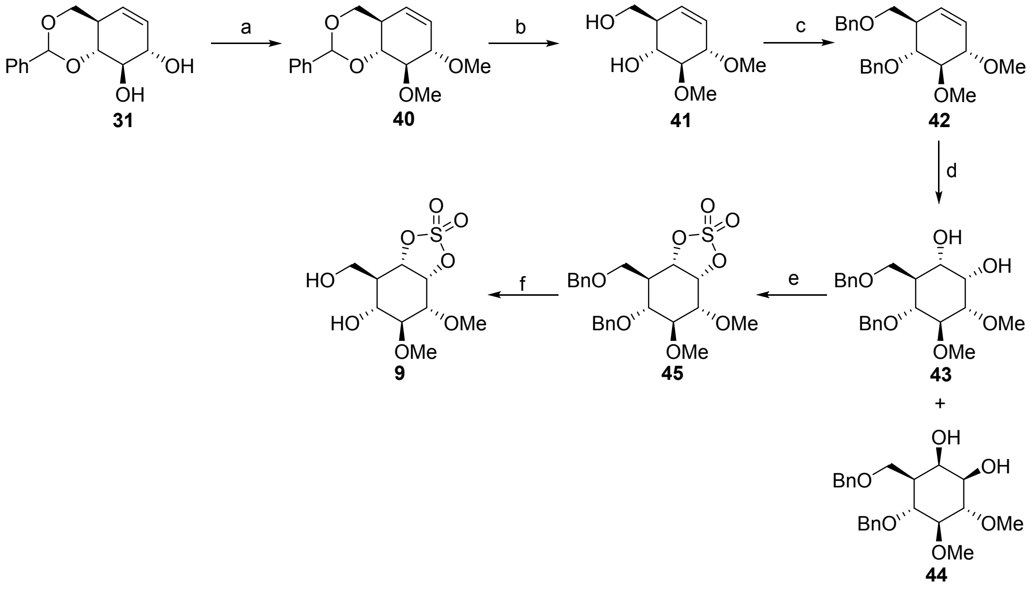

Scheme S2. Preparation of target compound **9**. Reagents and conditions: (a) MeI, 60% NaH, DMF, 4 h, 91%, (b) TFA, H_2O, DCM, 1.5 h, 83%, (c) BnBr, TBAI, 60% NaH, DMF, 5 h, 94%, (d) $RuCl_3 \cdot H_2O$, $NaIO_4$, EtOAc/H_2O/MeCN, 2 h, 39% (**43**), 29% (**44**), (e) i: $SOCl_2$, Et_3N, DCM, 1.5 h, ii: $RuCl_3 \cdot H_2O$, $NaIO_4$, EtOAc/H_2O/MeCN, 2.5 h, 73%, (f) Pd/C, H_2, MeOH/THF, 4 h, 93%.

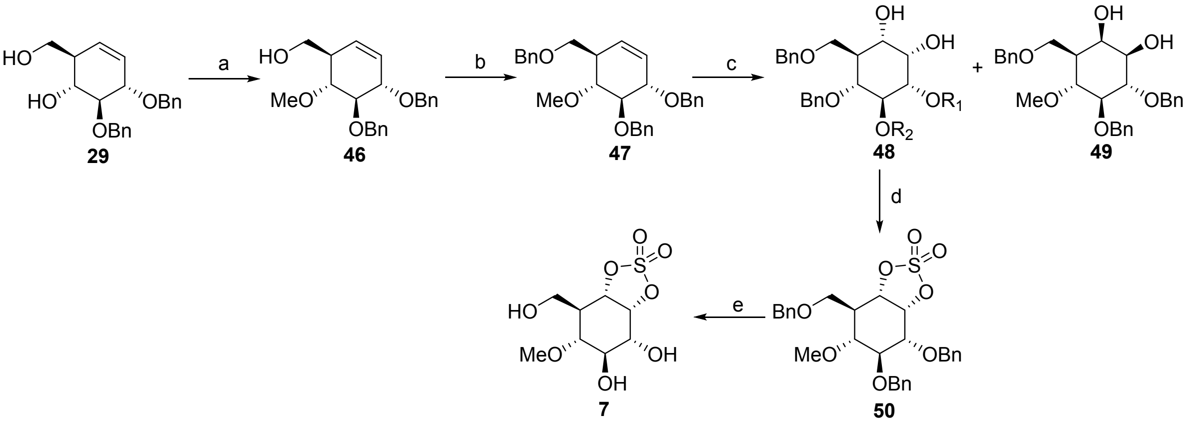

Scheme S3. Preparation of target compound **7**. Reagents and conditions: (a) i: TrtCl, DMAP, Et_3N, DMF, 18 h, ii: MeI, 60% NaH, DMF, 3 h, iii: *p*-TsOH, DCM/MeOH, 4 h, 62% over 3 steps (b) BnBr, TBAI, 60% NaH, DMF, 2 h, 95% (c) $RuCl_3 \cdot H_2O$, $NaIO_4$, EtOAc/H_2O/MeCN, 2 h (d) i: $SOCl_2$, Et_3N, DCM, 1.5, ii: $RuCl_3 \cdot H_2O$, $NaIO_4$, EtOAc/H_2O/MeCN, 2.5 h, 29% over 3 steps, (e) Pd/C, H_2, MeOH/THF, 5 h, 97%.

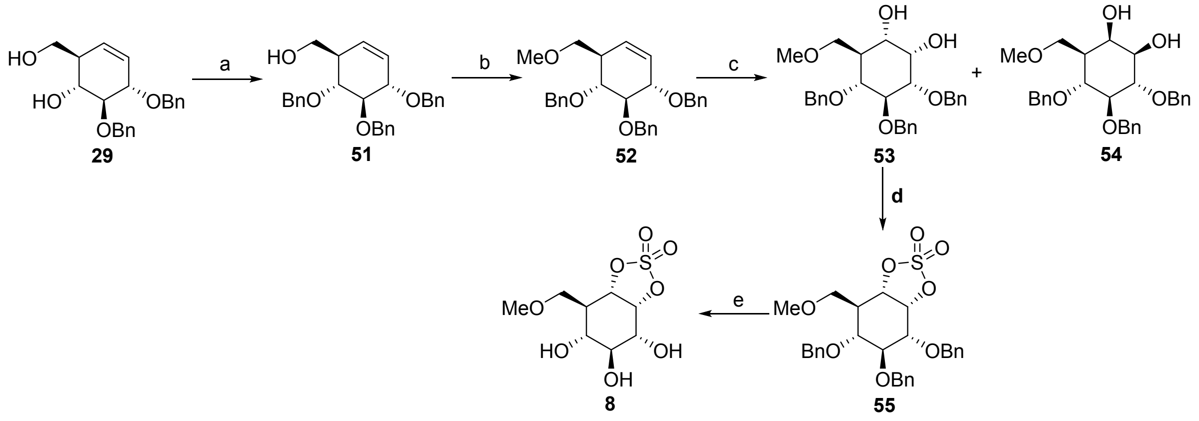

Scheme S4. Preparation of target compound **8**. Reagents and conditions: (a) i: TrtCl, DMAP, Et_3N, DMF, 18 h ii: BnBr, 60% NaH, TBAI, DMF, 6 h, iii: *p*-TsOH, DCM/MeOH, 4 h, 71% over 3 steps (b) MeI, 60% NaH, DMF, 2 h, 92% (c) $RuCl_3 \cdot H_2O$, $NaIO_4$, EtOAc/H_2O/MeCN, 2 h, 39% (**53**), 32% (**54**), (d) i: $SOCl_2$, Et_3N, DCM, 1.5 h, ii: $RuCl_3 \cdot H_2O$, $NaIO_4$, EtOAc/H_2O/MeCN, 2.5 h, 74% (e) Pd/C, H_2, MeOH/THF, 5 h, 93%.

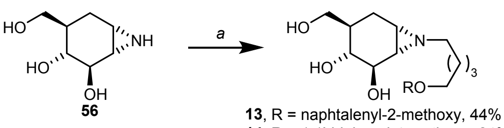

13, R = naphtalenyl-2-methoxy, 44%
14, R = 1,1'-biphenyl-4-methoxy, 24%
17, R = adamantyl-1-methoxy, 41%
18, R = naphtalenyl-1-methoxy, 38%

Scheme S5. Preparation of target compounds **13**, **14**, **17** and **18**. Reagents and conditions: (a) alkyl halogen, K_2CO_3, DMF, 3 h 100 °C, 44% (**13**), 24% (**14**), 41% (**17**), 38% (**18**).

Biochemical methods

Cell culture/lysates

Fibroblast cell lines were cultured in HAMF12-DMEM medium supplied with 10% (v/v) FCS, 0.1% (w/v) penicillin/streptomycin, and 0.5% (w/v) sodium pyruvate, under 7% CO_2 at 37 °C. Confluent fibroblasts were cultured 1:3 each week. Cell pellets were stored at -80 °C until lysates were prepared. Cell lysates were prepared in potassium phosphate (KPi) lysis buffer (25 mM K_2HPO_4/KH_2PO_4, pH 6.5, supplemented with protease inhibitor cocktail (EDTA-free, Roche, Basel, Switzerland) and 0.1 % (v/v) triton X-100) via one Freeze-thaw cycle, followed by sonication on ice. Protein concentration was determined with the BCA Protein Assay Kit (ThermoFisher Pierce™) with 10x lysate dilution in KPi buffer (without protease inhibitor). Lysates were stored in aliquots at -80 °C until use.

IC50

Enzymes used for IC_{50} were obtained as follows: recombinant human GAA (Myozyme) were obtained from Genzyme, USA and fibroblast cell lysates were used for ER-II alpha-glucosidase. Apparent IC_{50} values were determined throughout pre-incubation of 12.5 µL enzyme-mixture with 12.5 µL inhibitor for 30 minutes at 37 °C. GAA activity was measured with 47 nM enzyme (Myozyme) and 100 µL 3 mM 4-MU-α-D-glucopyranoside for 30 minutes at 37 °C. ER-II activity was measured using fibroblast cell lysates containing 10 µg protein (concentration was determined with BCA protein assay kit; Thermo Fisher) and 100 µL, 3 mM 4-MU α-D-glucopyranoside for 1 hour at 37 °C. After incubation with substrate mixture, the enzymatic reactions were quenched with 200 µL 1 M NaOH-Glycine (pH 10.3) and hydrolyzed 4-MU fluorescence is measured with a LS55 fluorescence spectrophotometer (Perkin Elmer: λ_{EX} 366 nm, λ_{EM} 445 nm). Background fluorescence (enzyme-mixture without substrate) is subtracted from the mean value, and normalized with maximal activity (without inhibitor). GAA is diluted in 150 mM McIlvain buffer pH 4.0 supplemented with 0.1% bovine serum albumin (BSA, w/v%) and 0.01% NaN_3 as bacteriostatic. ER-II is diluted in 150 mM McIlvain buffer pH 7.0 supplemented with 0.1% bovine serum albumin (BSA, w/v%) and 0.01% NaN_3 as bacteriostatic. Values plotted for concentration inhibitor are those in the final reaction mixture containing enzyme, inhibitor and substrate (125 µL total). The IC_{50} value is the average of two-/triplicates from technical triplicates.

Time-dependent inhibition

To study the type of inhibition, GAA and fibroblast cell lysates were pre-incubated for 5, 10, 15, 30, and 60 minutes with inhibitor (2x IC_{50} value) at 37 °C. Thereafter, 100 µL of substrate mixture (3 mM 4-MU α-D-glucopyranoside pH 4.0 for GAA, pH 7.0 for ER-II alpha-glucosidase) was added and incubated for 30 minutes (GAA) or 60 minutes (ER-II alpha-glucosidase). Finally, stop buffer (1 M glycine-NaOH pH 10.3) was added to stop the reaction and hydrolyzed 4-MU fluorescence was measured. Background fluorescence (enzyme-mixture without substrate) was subtracted from the mean value, and normalized to maximal activity (without inhibitor). Time was plotted *vs* residual enzyme activity. either a straight line was observed or decreased activity over time, relating to non-covalent or covalent inhibition, respectively.

Docking Method

The crystal structure co-ordinates of murine endoplasmic reticulum α-glucosidase II in complex with D-glucose (PDB: 5H9O) [5] were imported into Maestro software (Release 2024-1, Schrödinger, LLC, New York, NY, 2024) and prepared using the Protein Preparation module (pH = 7.4) [6]. A receptor grid was generated centred around the bound D-glucose molecule. The ligand was imported into the Maestro GUI and prepared using the LigPrep tool. Prepared ligands were docked into the generated receptor grid using the Glide module and the OPLS4 forcefield [7-11]. XP mode (extra precision) and

flexible ligand sampling were utilised, and epik state penalties were added to the docking scores [12]. Conformational, torsional and positional restraints were not used, except for stereochemical definitions in the ligand file. A maximum of 10 outputted poses was requested, and post-docking minimisation was carried out. Covalent docking was also performed using the covalent docking module of Glide [13]. The prepared ligand was docked into a receptor grid generated around the bound D-glucose molecule, and Asp564 was defined as the reactive residue. A custom reaction type for the aziridine and cyclic sulfate was created, allowing for attack of the aspartate residue at position 1 of the ligand definition (cyclic sulfate: [C][O][S]=[O]; aziridine [C][N][C]), and bond breakage between positions 1 and 2 of the ligand definition. A pre-defined reaction type was used for the epoxide. Docking was carried out in thorough 'pose prediction' mode, and MM-GBSA scoring was performed. Conformational, torsional and positional restraints were not used, except for stereochemical definitions in the ligand file.

The docking poses were exported as .pdb files and imported into ChimeraX, where figures were generated [14].

Cytopathic Effect (CPE) reduction assay on H1299/ACE2

CPE reduction assays were performed as previously described [15]. Briefly, H1299/ACE2 cells were seeded in 96-well plates at a density of $1*10^4$ cells per well. The next day, cells were infected with SARS-CoV-2/Leiden0008 in the presence of 2-fold serial dilutions of compound. 2 days post infection the CellTiter 96 aqueous nonradioactive cell proliferation kit (Promega) was used to measure the cell viability of infected (protection) and non-infected cells (assessment of cytotoxicity). EC50 values reported are the mean values from three independent experiments and were calculated using GraphPad Prism 6.

Fluorescent IC_{50} assays on recombinant human GAA (Myozyme) and ER-II for compounds

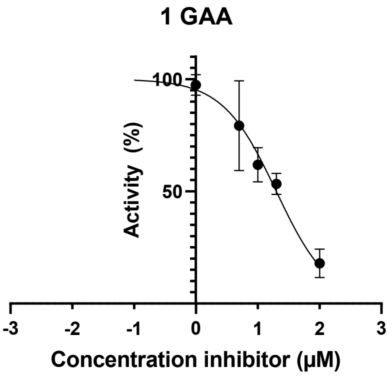

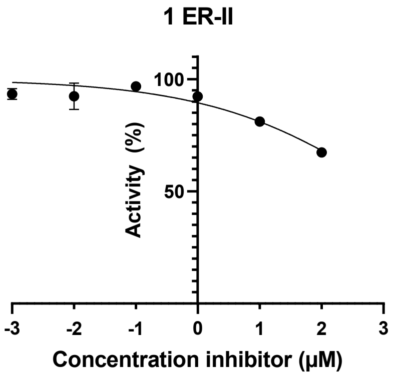

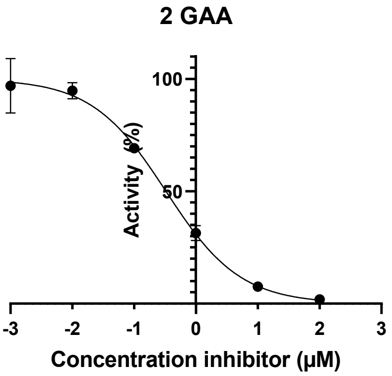

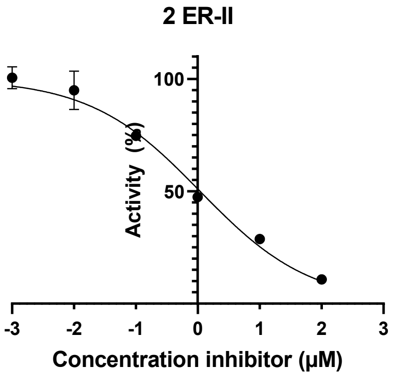

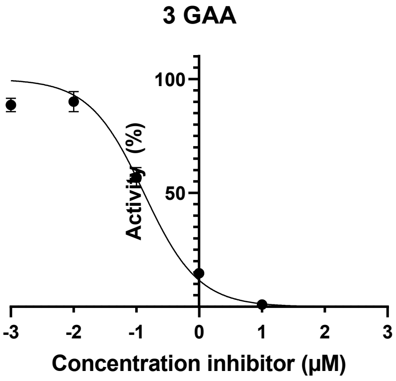

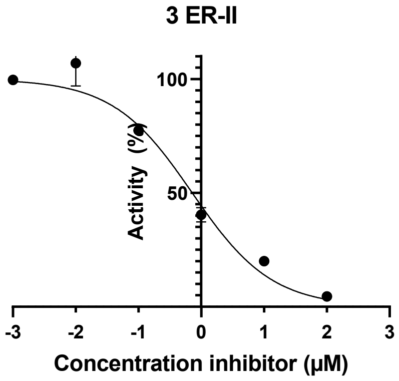

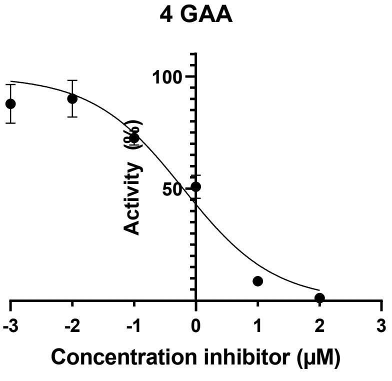

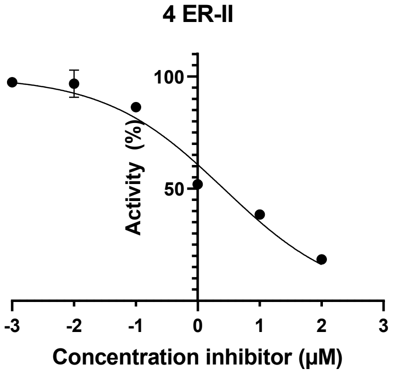

5 GAA

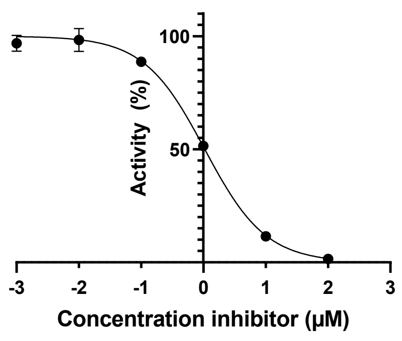

5 ER-II

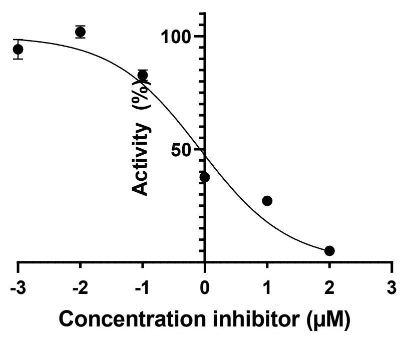

6 GAA

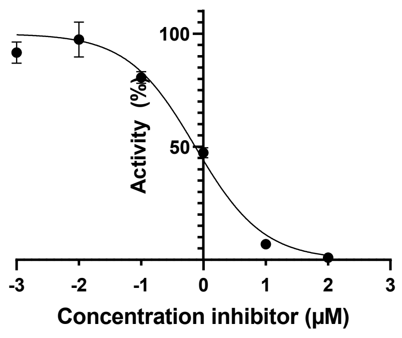

6 ER-II

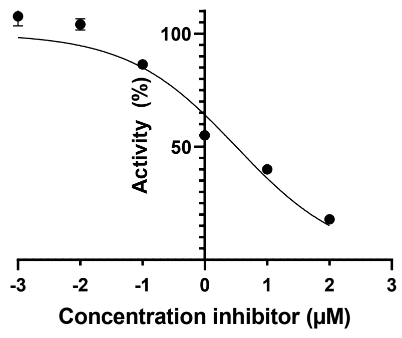

7 GAA

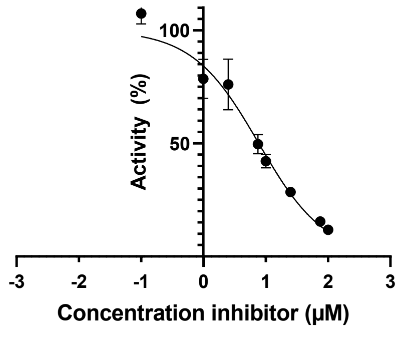

7 ER-II

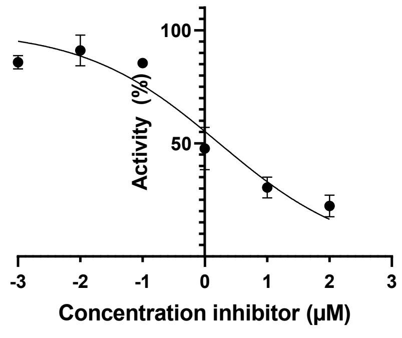

8 GAA

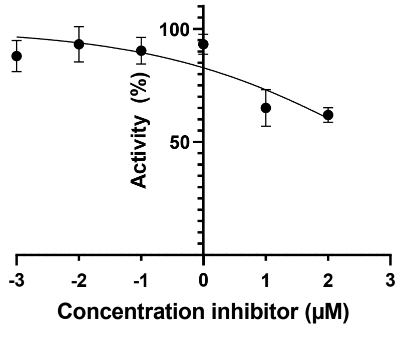

8 ER-II

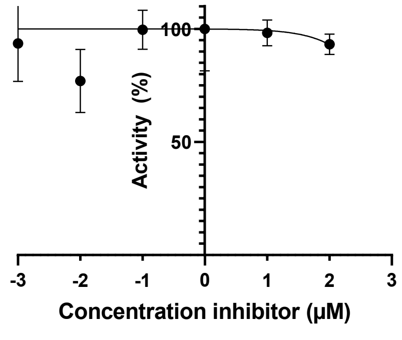

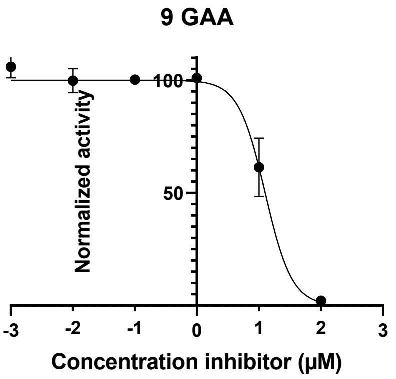
9 GAA
Normalized activity
100
50
-3 -2 -1 0 1 2 3
Concentration inhibitor (µM)

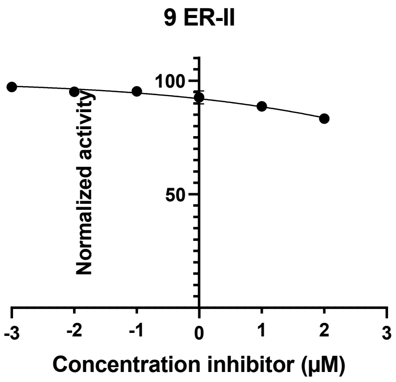
9 ER-II
Normalized activity
100
50
-3 -2 -1 0 1 2 3
Concentration inhibitor (µM)

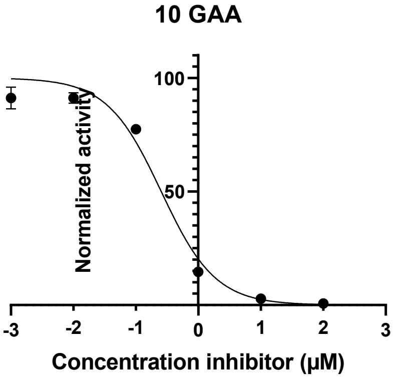
10 GAA
Normalized activity
100
50
-3 -2 -1 0 1 2 3
Concentration inhibitor (µM)

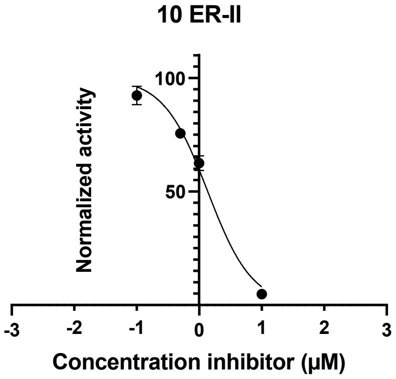
10 ER-II
Normalized activity
100
50
-3 -2 -1 0 1 2 3
Concentration inhibitor (µM)

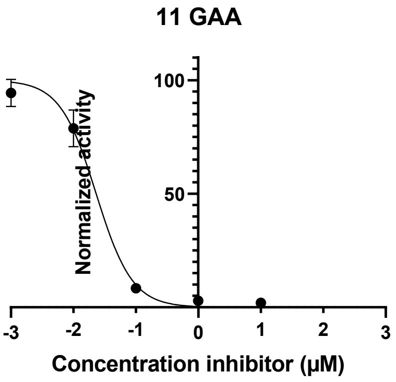
11 GAA
Normalized activity
100
50
-3 -2 -1 0 1 2 3
Concentration inhibitor (µM)

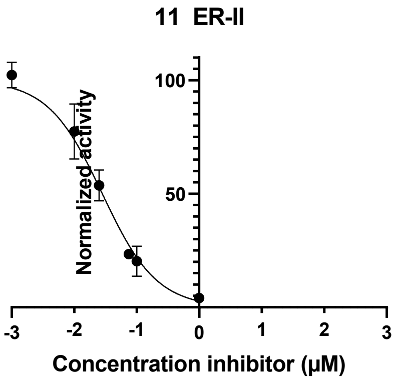
11 ER-II
Normalized activity
100
50
-3 -2 -1 0 1 2 3
Concentration inhibitor (µM)

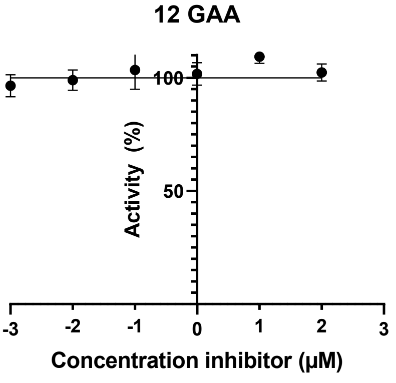
12 GAA
Activity (%)
100
50
-3 -2 -1 0 1 2 3
Concentration inhibitor (µM)

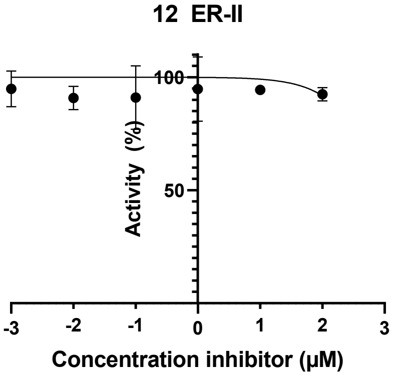
12 ER-II
Activity (%)
100
50
-3 -2 -1 0 1 2 3
Concentration inhibitor (µM)

13 GAA

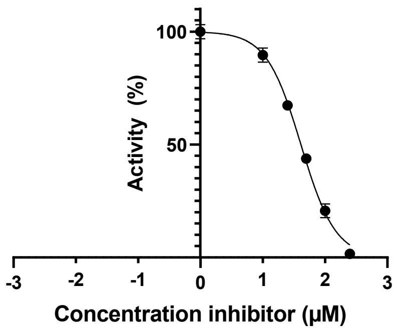

13 ER-II

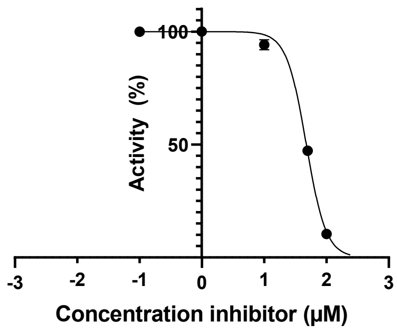

14 GAA

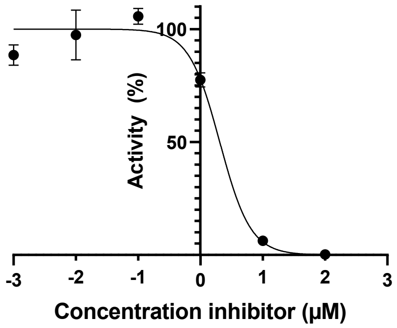

14 ER-II

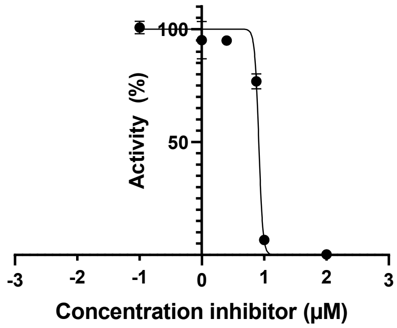

15 GAA

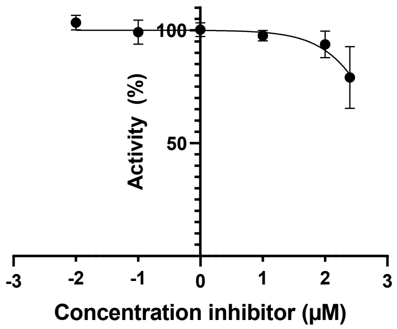

15 ER-II

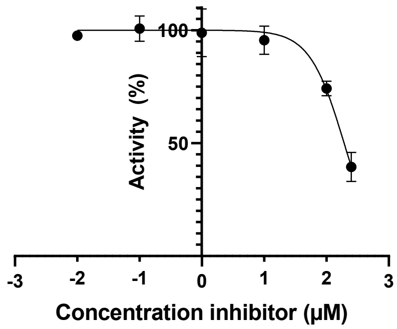

16 GAA

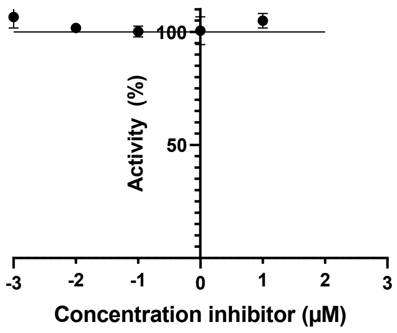

16 ER-II

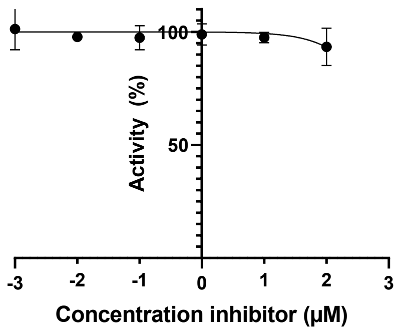

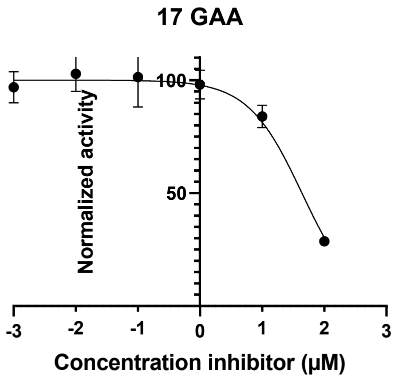
17 GAA
Normalized activity
100
50
-3 -2 -1 0 1 2 3
Concentration inhibitor (μM)

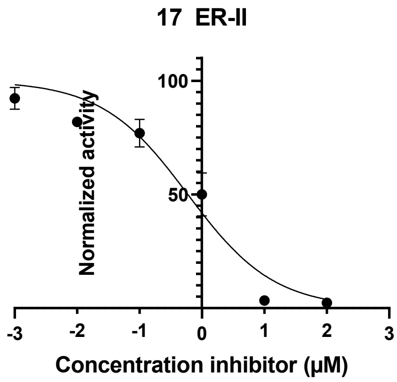
17 ER-II
Normalized activity
100
50
-3 -2 -1 0 1 2 3
Concentration inhibitor (μM)

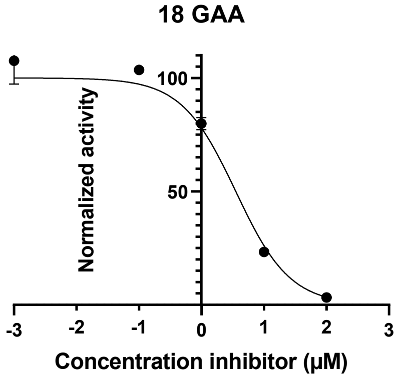
18 GAA
Normalized activity
100
50
-3 -2 -1 0 1 2 3
Concentration inhibitor (μM)

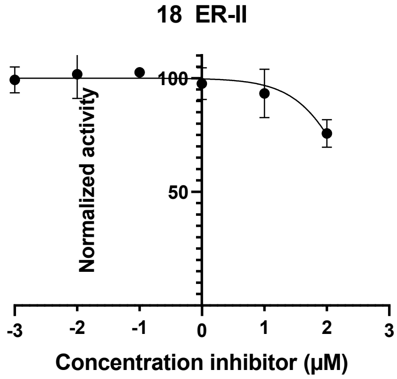
18 ER-II
Normalized activity
100
50
-3 -2 -1 0 1 2 3
Concentration inhibitor (μM)

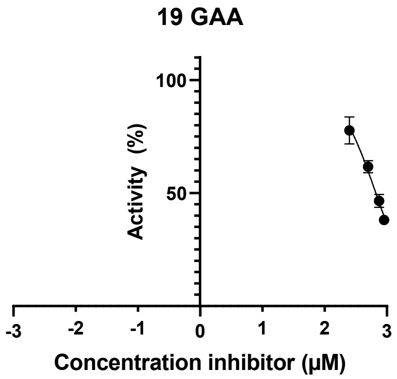
19 GAA
Activity (%)
100
50
-3 -2 -1 0 1 2 3
Concentration inhibitor (μM)

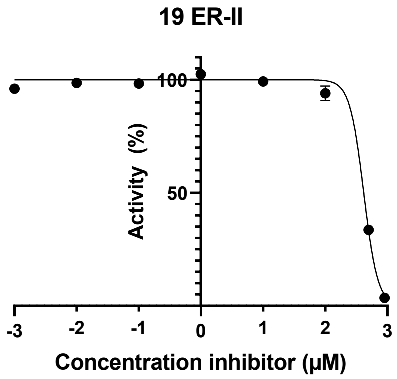
19 ER-II
Activity (%)
100
50
-3 -2 -1 0 1 2 3
Concentration inhibitor (μM)

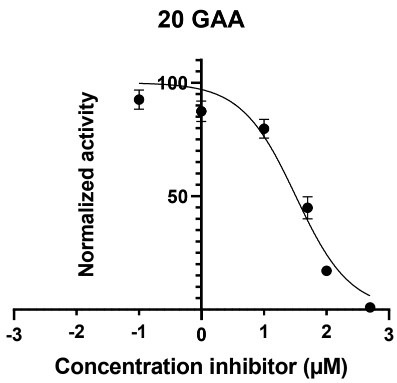
20 GAA
Normalized activity
100
50
-3 -2 -1 0 1 2 3
Concentration inhibitor (μM)

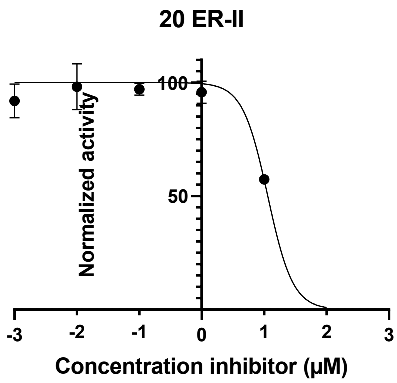
20 ER-II
Normalized activity
100
50
-3 -2 -1 0 1 2 3
Concentration inhibitor (μM)

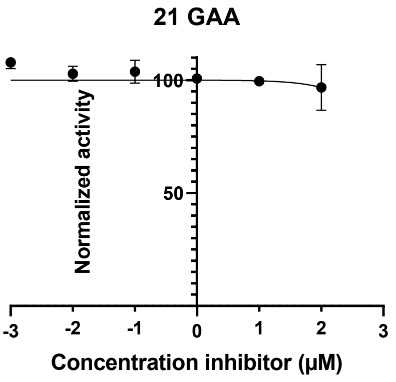
21 GAA
Normalized activity
100
50
-3 -2 -1 0 1 2 3
Concentration inhibitor (μM)

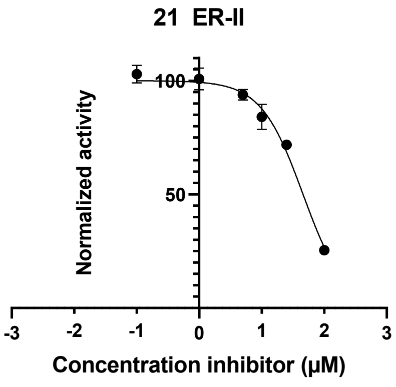
21 ER-II
Normalized activity
100
50
-3 -2 -1 0 1 2 3
Concentration inhibitor (μM)

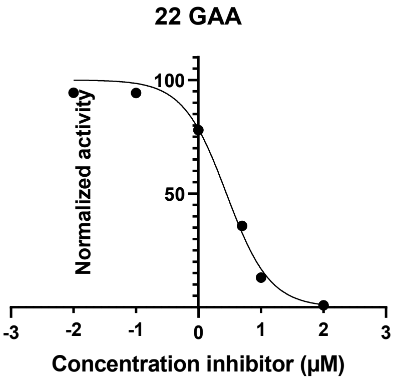
22 GAA
Normalized activity
100
50
-3 -2 -1 0 1 2 3
Concentration inhibitor (μM)

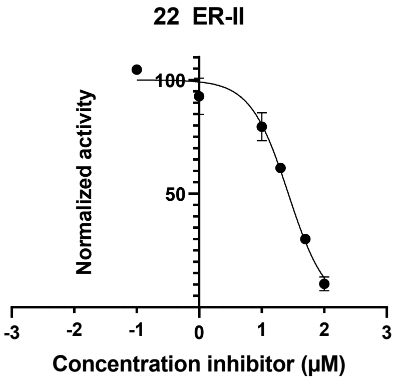
22 ER-II
Normalized activity
100
50
-3 -2 -1 0 1 2 3
Concentration inhibitor (μM)

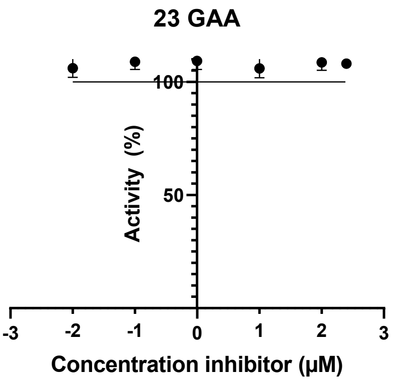
23 GAA
Activity (%)
100
50
-3 -2 -1 0 1 2 3
Concentration inhibitor (μM)

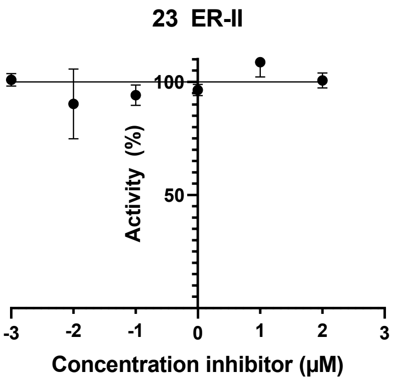
23 ER-II
Activity (%)
100
50
-3 -2 -1 0 1 2 3
Concentration inhibitor (μM)

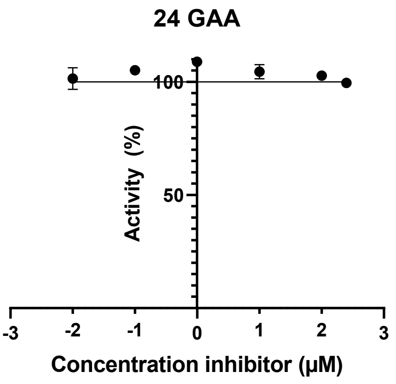
24 GAA
Activity (%)
100
50
-3 -2 -1 0 1 2 3
Concentration inhibitor (μM)

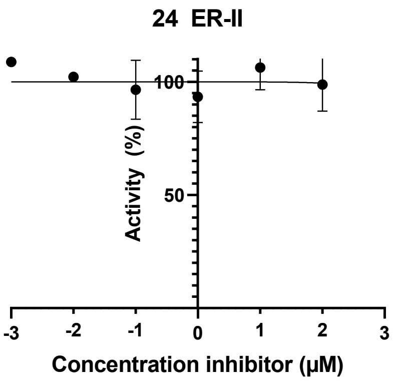
24 ER-II
Activity (%)
100
50
-3 -2 -1 0 1 2 3
Concentration inhibitor (μM)

25 GAA

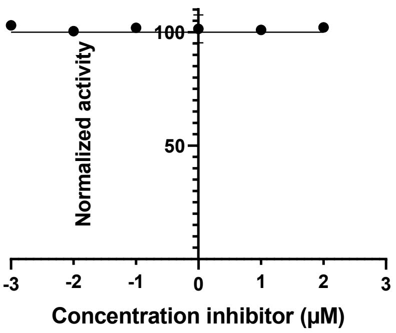

25 ER-II

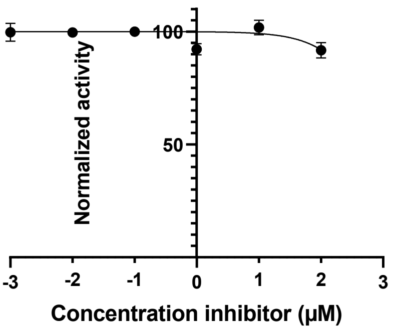

26 GAA

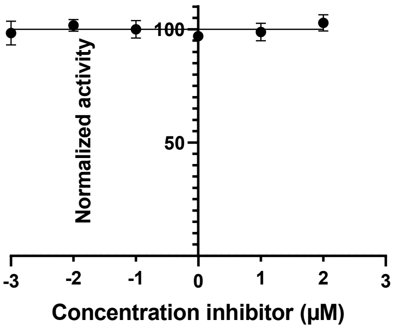

26 ER-II

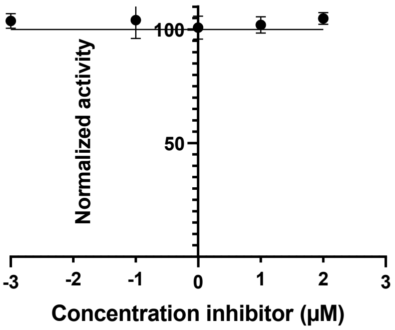

27 GAA

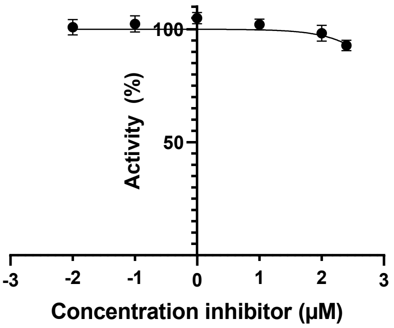

27 ER-II

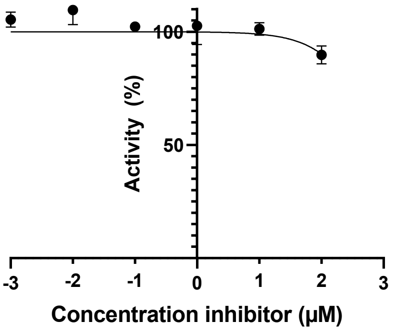

28 GAA

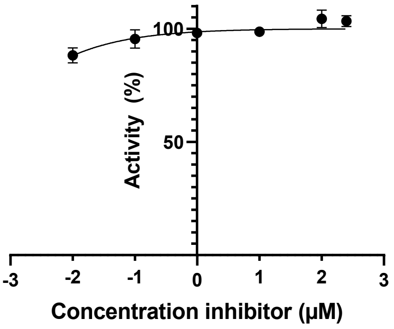

28 ER-II

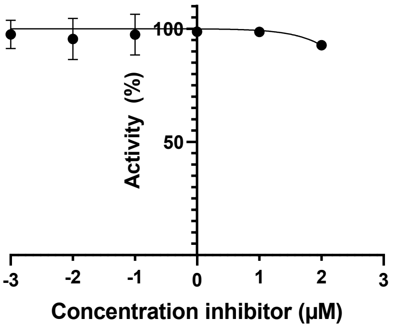

General experimental procedures

All chemicals were of commercial grade and were used as received unless stated otherwise. Solvents used in synthesis were dried and stored over 4Å molecular sieves. Deuterated chloroform was stored over activated 3 Å molecular rods (rods, size 1/16 in., Sigma Aldrich) and potassium carbonate. Flash column chromatography was performed on silica gel 60 Å (0.04 – 0.063 mm, Screening Devices B.V.). TLC analysis was performed on TLC Silica gel 60 (Kieselgel 60 F254, Merck) with UV detection (254 nm) and by spraying with a solution of $(NH_4)_6Mo_7O_{24}\cdot H_2O$ (25 g/L) and $(NH_4)_4Ce(SO_4)_4\cdot 2H_2O$ (10 g/L) in 10% sulfuric acid in water followed by charring at ± 200 °C. TLC-MS analysis was performed on a Camag TLC-MS Interface coupled with an API165 (SCIEX) mass spectrometer (eluted with *tert*-butylmethylether/EtOAc/MeOH, 5/4/1, v:v:v +0.1% formic acid, flow rate 0.12 mL/min). High-resolution mass spectra (HRMS) were recorded on a Waters Synapt G2-Si (TOF) equipped with an electrospray ion source in positive mode (source voltage 3.5 kV) and an internal lock mass LeuEnk ($M+H^+$ = 556.2771). ^{1}H and ^{13}C NMR spectra were recorded on a Bruker AV-400 NMR (400 and 101 MHz respectively) or a Bruker AV-500 NMR (500 and 126 MHz respectively). All samples were measured in $CDCl_3$, unless stated otherwise. Chemical shifts (δ) are given in ppm relative to tetramethyl silane as internal standard or the residual signal of the deuterated solvent. Coupling constants (*J*) are given in Hz. All given ^{13}C APT spectra are proton decoupled. NMR peak assignment was accomplished using COSY, HSQC. Proton and carbon numbering for NMR peak assignment was done as followed: numbering was done similarly to their glucose counterparts and not their respective nomenclature. Numbering starts at the 'anomeric' center and progresses similarly as their glucose counterpart. 'H-7' or 'C-7' is used where the intramolecular oxygen is substituted for carbon.

Synthetic procedures

(1*R*,2*R*,3*S*,6*R*)-6-(hydroxymethyl)cyclohex-4-ene-1,2,3-triol (30)

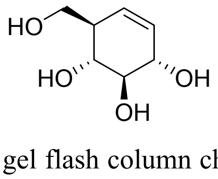

Cyclohexene **29** (0.85 g, 2.5 mmol, 1.0 eq) was dissolved in anhydrous DCM (17 mL), cooled to -78℃ and BCl_3 (1M solution in DCM, 12.5 mmol, 5.0 eq) was added dropwise. The reaction was stirred at -78℃ for 4 h and quenched with MeOH. The reaction mixture was concentrated *in vacuo* and the crude material was purified by silica gel flash column chromatography (0%→30% MeOH in EtOAc, silica prewashed with MeOH) to obtain **30** (0.35 g, 2.2 mmol, 89%) as a white solid. ^{1}H NMR (400 MHz, MeOD) δ 5.63 (dt, *J* = 10.2, 1.7 Hz, 1H, H-1), 5.58 (dt, *J* = 10.1, 2.0 Hz, 1H, H-6), 4.04 (ddd, *J* = 7.7, 3.8, 2.0 Hz, 1H, H-2), 3.79 (dd, *J* = 10.6, 4.1 Hz, 1H, H-7b), 3.60 (dd, *J* = 10.7, 6.1 Hz, 1H, H-7a), 3.49 – 3.39 (m, 2H, H-3, H-4), 2.27 (ddq, *J* = 8.3, 4.3, 2.1 Hz, 1H, H-5). ^{13}C NMR (101 MHz, MeOD) δ 128.9 (C-6), 126.5 (C-1), 76.8 (C-3), 71.5 (C-2), 69.9 (C-4), 61.3 (C-7), 45.6 (C-5). HRMS (ESI) m/z: [$M+Na^+$] calcd for $C_7H_{12}O_4Na$ 183.0633, found 183.0634.

(4a*R*,7*S*,8*R*,8a*R*)-2-phenyl-4a,7,8,8a-tetrahydro-4H-benzo[*d*][1,3]dioxine-7,8-diol (31)

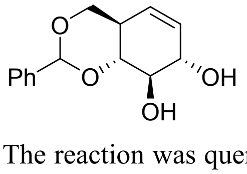

Cyclohexene **30** (80 mg, 0.5 mmol, 1.0 eq) was dissolved in an anhydrous 4:1 mixture of ACN/DMF (2.5 mL) and benzaldehyde dimethylacetal (0.19 mL, 1.25 mmol, 2.5 eq) was added. The pH of the mixture was adjusted to 2 with *p*TsOH and the reaction mixture was stirred on a rotary evaporator (rotavap) for 4 h at 60℃ and 650 mbar. The reaction was quenched with Et_3N and diluted with EtOAc. The organic layer was washed with sat. aq.

$NaHCO_3$ and brine, dried over $MgSO_4$, filtered and concentrated *in vacuo*. The crude product was purified by silica gel flash column chromatography (0%→10% MeOH in DCM) to obtain **31** (92 mg, 0.37 mmol, 73%) as a white solid. ^{1}H NMR (400 MHz, $CDCl_3$) δ 7.56 – 7.43 (m, 2H, CH_{Ar}), 7.43 – 7.31 (m, 3H, CH_{Ar}), 5.62 (dt, *J* = 10.0, 2.8 Hz, 1H, H-6), 5.56 (s, 1H, H-8), 5.36 – 5.31 (m, 1H, H-1), 4.33 – 4.22 (m, 2H, H-2, H-7b), 3.88 (dd, *J* = 10.4, 7.3 Hz, 1H, H-3), 3.63 – 3.55 (m, 2H, H-4, H-7a), 2.62 (ddtd, *J* = 11.4, 6.6, 3.4, 1.6 Hz, 1H, H-5). ^{13}C NMR (101 MHz, $CDCl_3$) δ 137.8 (C_{qAr}), 130.6 (C-6), 129.3, 128.4, 126.4 (CH_{Ar}), 124.3 (C-1), 102.3 (C-8), 80.8 (C-4), 75.7 (C-3), 73.9 (C-2), 70.0 (C-7), 38.6 (C-5). HRMS (ESI) m/z: [M+Na$^+$] calcd for $C_{14}H_{16}O_4Na$ 271.0946, found 271.0948.

((((1*R*,2*R*,3*S*,6*R*)-6-((benzyloxy)methyl)-2-methoxycyclohex-4-ene-1,3-diyl)bis(oxy))bis(methylene))dibenzene (32) Diol **31** (0.12 g, 0.5 mmol, 1.0 eq) was co-evaporated (3x) toluene and dissolved in anhydrous MeCN (2.5 mL). Subsequently, KI (83 mg, 0.5 mmol, 1.0 eq), K_2CO_3 (83 mg, 0.6 mmol, 1.2 eq) and 2-aminoethyl diphenylborinate (38 mg, 0.15 mmol, 0.3 eq) and BnBr (59 µL, 0.5 mmol, 1.0 eq) were added to the solution and the mixture was stirred at 60 °C for 18 h. The reaction was quenched with water and diluted with EtOAc. The organic layer was washed with sat. aq. $NaHCO_3$ and brine, dried over $MgSO_4$, filtered and concentrated *in vacuo*. The crude material was filtered over a silica plug. The filtered material was dissolved in DCM (2.5 mL) and cooled to 0 °C. Water (1 mL) and TFA (0.19 mL, 2.5 mmol, 5.0 eq) were added and the reaction was stirred for 1.5 h at rt after which TLC analysis indicated full conversion of the starting material. The solution was washed with sat. aq. $NaHCO_3$, H_2O and brine, dried over $MgSO_4$, filtered and concentrated *in vacuo*. The crude material was dissolved in anhydrous DMF and cooled to 0°C. NaH (60% dispersion in mineral oil) was added and the reaction was stirred for 15 min at 0°C. Subsequently, MeI was added dropwise. The reaction was stirred for 2 h at rt, diluted with Et_2O and quenched with MeOH at 0°C. The organic layer was washed with H_2O and brine (2x), dried over $MgSO_4$, filtered and concentrated *in vacuo*. The crude material was purified by silica gel flash column chromatography (0%→15% EtOAc in pentane) to obtain **32** (80 mg, 0.18 mmol, 36% over 3 steps) as a colorless oil. ^{1}H NMR (500 MHz, $CDCl_3$) δ 7.43 – 7.33 (m, 2H, CH_{Ar}), 7.31 – 7.23 (m, 13H, CH_{Ar}), 5.74 – 5.69 (m, 1H, H-6), 5.67 (dt, *J* = 10.2, 1.7 Hz, 1H, H-1), 4.91 (s, 1H, CH*H*Ph), 4.89 (s, 1H, CH*H*Ph), 4.71 (s, 1H, CH*H*Ph), 4.68 (s, 1H, CH*H*Ph), 4.56 – 4.52 (m, 1H, CH*H*Ph), 4.51 (d, *J* = 12.2 Hz, 1H, CH*H*Ph), 4.21 (ddd, *J* = 7.8, 3.5, 1.7 Hz, 1H, H-2), 3.74 (dd, *J* = 10.1, 7.8 Hz, 1H, H-3), 3.62 (dd, *J* = 9.0, 3.4 Hz, 1H, H-7b), 3.57 (dd, *J* = 9.0, 5.0 Hz, 1H, H-7a), 3.52 (s, 3H, OCH_3), 3.41 (t, *J* = 9.8 Hz, 1H, H-4), 2.51 – 2.43 (m, 1H, H-5). ^{13}C NMR (126 MHz, $CDCl_3$) δ 139.5, 139.0, 138.6 (3x C_{qAr}), 129.6 (C-1), 128.5, 128.4, 128.1, 128.0, 127.9, 127.8, 127.7, 127.6 (CH_{Ar}), 127.2 (C-6), 85.7 (C-3), 81.2 (C-2), 80.9 (C-4), 75.6, 73.7, 72.8 (3x CH_2Ph), 70.1 (C-7), 59.1 (OCH_3), 45.1 (C-5). HRMS (ESI) m/z: [M+Na$^+$] calcd for $C_{29}H_{32}O_4Na$ 467.2198, found 467.2200.

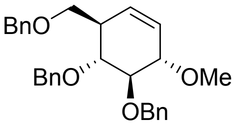

(3a*S*,4*R*,5*R*,6*S*,7*R*,7a*R*)-5-(benzyloxy)-4-((benzyloxy)methyl)-7-methoxy-6-(naphthalen-2-ylmethoxy)hexahydrobenzo[*d*][1,3,2]dioxathiole 2,2-dioxide (38) A solution of $NaIO_4$ (0.13 g, 0.63 mmol, 2.5 eq) and $RuCl_3 \cdot H_2O$ (3.7 mg, 18 µmol, 0.07 eq) in water (2.0 mL) was added dropwise to an ice-cooled and vigorously stirred solution of cyclohexene **32** (0.11 g, 0.25 mmol, 1.0 eq) in EtOAc/MeCN 1:1 (7.5 mL). The reaction was stirred for 2 h at 0°C after which TLC analysis indicated full conversion. The reaction was quenched with sat. aq. $Na_2S_2O_3$ and the aqueous layer was extracted with EtOAc (3x). The combined organic layers were washed with brine, dried over $MgSO_4$, filtered and concentrated *in vacuo*. The crude material was purified by silica gel flash column chromatography (5%→30% acetone in pentane) to obtain **34** as white solid. $SOCl_2$ (22 µL, 0.31 mmol, 3.5 eq) was added dropwise over 5 min to an ice- cooled solution of diol **34** (42 mg, 88 µmol, 1.0 eq) and Et_3N (49 µL, 0.35 mmol, 4.0 eq) in DCM (1.0 mL). The reaction was stirred for 1,5 h at 0°C after which TLC analysis indicated full conversion of the starting material. The reaction mixture was diluted with cold Et_2O and the organic layer was washed with cold water and brine, dried over $MgSO_4$, filtered and concentrated *in vacuo*. Final traces of Et_3N were removed under high vacuum. The crude material was dissolved in EtOAc/ACN and a solution of $NaIO_4$ and $RuCl_3 \cdot H_2O$ in water was added at 0°C. The reaction was stirred for 2,5 h at this temperature and subsequently diluted with EtOAc and quenched with sat. aq. $N_2S_2O_3$. The layers were separated and the aqueous layer was extracted with EtOAc. The combined organic layers were washed with water and brine, dried over $MgSO_4$, filtered and concentrated *in vacuo*. The crude product was purified by silica gel flash column chromatography (0%→15%

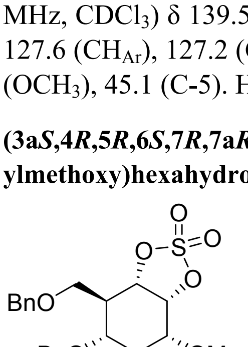

EtOAc in pentane) to obtain cyclosulfate **38** (21 mg, 40 µmol, 46%) as a colorless oil. ^{1}H NMR (500 MHz, $CDCl_3$) δ 7.38 – 7.29 (m, 15H, CH_{Ar}), 5.09 – 5.02 (m, 2H, H-1, H-6), 4.77 – 4.71 (m, 4H, 4x CH*H*Ph), 4.54 (s, 2H, 2x CH*H*Ph), 3.86 – 3.80 (m, 2H, H-3, H-7b), 3.71 – 3.66 (m, 1H, H-2), 3.59 (dd, *J* = 9.5, 2.2 Hz, 1H, H-7a), 3.52 (s, 3H, OCH_3), 3.26 (dd, *J* = 11.7, 8.2 Hz, 1H, H-4), 2.44 (ddt, *J* = 11.6, 9.5, 2.2 Hz, 1H, H-5). ^{13}C NMR (126 MHz, $CDCl_3$) δ 138.4, 137.9, 137.5 (3x C_{qAr}), 128.9, 128.7, 128.6, 128.4, 128.2, 128.12, 128.08, 128.05, 128.0 (CH_{Ar}), 81.9 (C-3), 81.1 (C-1), 79.8 (C-6), 77.5 (C-4), 76.1 (C-2), 75.0, 74.1, 73.7 (3x CH_2Ph), 64.1 (C-7), 61.2 (OCH_3), 43.6 (C-5). HRMS (ESI) m/z: [M+Na$^+$] calcd for $C_{29}H_{32}O_8SNa$ 563.1716, found 563.1717.

(3a*S*,4*R*,5*R*,6*S*,7*R*,7a*R*)-5,6-dihydroxy-4-(hydroxymethyl)-7-methoxyhexahydrobenzo[*d*][1,3,2]dioxathiole 2,2-dioxide (5) Cyclosulfate **38** (15 mg, 28 µmol, 1.0 eq) was dissolved in MeOH/THF (1 mL) and purged with N_2. Pd/C (10 wt%, 12 mg, 11 µmol, 0.4 eq) was added to the solution and the reaction mixture was again purged with N_2. The reaction mixture was flushed for 5 min with H_2 before being left to stir under H_2 atmosphere for 5 h. The reaction mixture was flushed with N_2 and filtered over whatman filter paper. The filtrate was concentrated *in vacuo* and the crude material was purified by silica gel flash column chromatography (0%→20% MeOH in DCM, silica prewashed with MeOH) to obtain **5** (6.8 mg, 25 µmol, 91%) as a white solid. ^{1}H NMR (500 MHz, MeOD) δ 5.11 – 5.05 (m, 1H, H-1), 4.08 (dd, J = 11.1, 2.3 Hz, 1H, H-7B), 3.76 (dd, J = 10.9, 4.0 Hz, 1H, H-6), 3.69 (dd, J = 11.1, 2.8 Hz, 1H, H-7A), 3.63 – 3.54 (m, 2H, CH-2, H-3), 3.39 – 3.34 (m, 1H, H-4), 2.94 (s, 3H, CH_3), 1.98 (tt, J = 11.1, 2.6 Hz, 1H, H-5). ^{13}C NMR (126 MHz, MeOD) δ 85.3 (C-1), 74.6 (C-2/3), 70.9 (C-2/3), 68.8 (C-4), 62.7 (C-6), 57.1 (C-7), 47.1 (C-5), 37.3 (CH_3). HRMS (ESI) m/z: [M+Na$^+$] calcd for $C_8H_{14}O_8SNa$ 293.2418, found 293.2420.

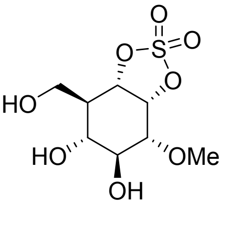

((((1*R*,2*R*,3*S*,6*R*)-6-((benzyloxy)methyl)-2-methoxycyclohex-4-ene-1,3-diyl)bis(oxy))bis(methylene))dibenzene (33) Diol **31** (0.12 g, 0.5 mmol, 1.0 eq) was co-evaporated (3x) toluene and dissolved in anhydrous MeCN (2.5 mL). Subsequently, KI (83 mg, 0.5 mmol, 1.0 eq), K_2CO_3 (83 mg, 0.6 mmol, 1.2 eq) and 2-aminoethyl diphenylborinate (38 mg, 0.15 mmol, 0.3 eq) and MeI (31 µL, 0.5 mmol, 1.0 eq) were added to the solution and the mixture was stirred at 60 °C for 18 h. The reaction was quenched with water and diluted with EtOAc. The organic layer was washed with sat. aq. $NaHCO_3$ and brine, dried over $MgSO_4$, filtered and concentrated *in vacuo*. The crude material was filtered over a silica plug. The filtered material was dissolved in DCM (2.5 mL) and cooled to 0 °C. Water (1 mL) and TFA (0.19 mL, 2.5 mmol, 5.0 eq) were added and the reaction was stirred for 1.5 h at rt after which TLC analysis indicated full conversion of the starting material. The solution was washed with sat. aq. $NaHCO_3$, H_2O and brine, dried over $MgSO_4$, filtered and concentrated *in vacuo*. The crude material was dissolved in anhydrous DMF (10 mL) and cooled to 0°C. NaH (60% dispersion in mineral oil, 92 mg, 2.3 mmol, 4.5 eq) was added and the reaction was stirred for 15 min at 0°C. Subsequently, TBAI (9.2 mg, 25 µmol, 0.05 eq) and BnBr (0.21 mL, 1.8 mmol, 3.6 eq) were added. The reaction was stirred for 2 h at rt, diluted with Et_2O and quenched with MeOH at 0°C. The organic layer was washed with H_2O and brine (2x), dried over $MgSO_4$, filtered and concentrated *in vacuo*. The crude material was purified by silica gel flash column chromatography (0%→15% EtOAc in pentane) to obtain **33** (71 mg, 0.16 mmol, 31%) as a colorless oil. ^{1}H NMR (500 MHz, $CDCl_3$) δ 7.40 – 7.36 (m, 2H, CH_{Ar}), 7.35 – 7.26 (m, 13H, CH_{Ar}), 5.74 – 5.69 (m, 1H, H-6), 5.67 (dt, *J* = 10.2, 1.7 Hz, 1H, H-1), 4.93 (s, 1H, CH*H*Ph), 4.91 (s, 1H, CH*H*Ph), 4.72 (s, 1H, CH*H*Ph), 4.69 (s, 1H, CH*H*Ph), 4.59 – 4.55 (m, 1H, CH*H*Ph), 4.53 (d, *J* = 12.2 Hz, 1H, CH*H*Ph), 4.17 (ddd, *J* = 7.8, 3.5, 1.7 Hz, 1H, H-2), 3.75 (dd, *J* = 10.1, 7.8 Hz, 1H, H-3), 3.62 (dd, *J* = 9.0, 3.4 Hz, 1H, H-7b), 3.58 (dd, *J* = 9.0, 5.0 Hz, 1H, H-7a), 3.53 (s, 3H, OCH_3), 3.41 (t, *J* = 9.8 Hz, 1H, H-4), 2.51 – 2.46 (m, 1H, H-5). ^{13}C NMR (126 MHz, $CDCl_3$) δ 139.0, 138.9, 138.6 (3x C_{qAr}), 129.4 (C-1), 128.3, 128.2, 128.1, 128.0, 127.9, 127.8, 127.7, 127.6 (CH_{Ar}), 127.2 (C-6), 85.8 (C-3), 81.5 (C-2), 80.9 (C-4), 75.6, 73.5, 72.9 (3x CH_2Ph), 70.4 (C-7), 61.5 (OCH_3), 45.1 (C-5). HRMS (ESI) m/z: [M+Na$^+$] calcd for $C_{29}H_{32}O_4Na$ 467.2198, found 467.2200.

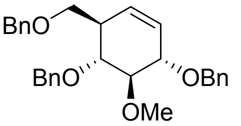

(3a*R*,4*R*,5*S*,6*R*,7*R*,7a*S*)-4,6-bis(benzyloxy)-7-((benzyloxy)methyl)-5-methoxyhexahydrobenzo[*d*][1,3,2]dioxathiole 2,2-dioxide (39) A solution of $NaIO_4$ (64 mg, 0.3 mmol, 2.5 eq)

and $RuCl_3 \cdot H_2O$ (1.7 mg, 8.4 µmol, 0.07 eq) in water was added dropwise to an ice-cooled and vigorously stirred solution of cyclohexene **33** (53 mg, 0.12 mmol, 1.0 eq)in EtOAc/MeCN 1:1 (6 mL). The reaction was stirred for

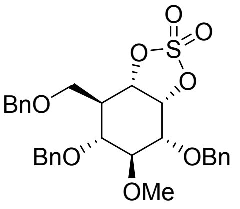

2 h at 0°C after which TLC analysis indicated full conversion. The reaction was quenched with sat. aq. $Na_2S_2O_3$ and the aqueous layer was extracted with EtOAc (3x). The combined organic layers were washed with brine, dried over $MgSO_4$, filtered and concentrated *in vacuo*. The crude material was purified by silica gel flash column chromatography (5%→ 30% acetone in pentane) to obtain diols **35** and **37**. $SOCl_2$ (14 µL, 0.19 mmol, 3.5 eq) was added dropwise over 5 min to an ice- cooled solution of diol **35** (26 mg, 54 µmol, 1.0 eq) and Et_3N (67 µL, 0.48 mmol, 4.0 eq) in DCM (1.2 mL). The reaction was stirred for 1,5 h at 0°C after which TLC analysis indicated full conversion of the starting material. The reaction mixture was diluted with cold Et_2O and the organic layer was washed with cold water and brine, dried over $MgSO_4$, filtered and concentrated *in vacuo*. Final traces of Et_3N were removed under high vacuum. The crude material was dissolved in EtOAc/MeCN 1:1 (4.4 mL) and a solution of $NaIO_4$ (24 mg, 0.11 mmol, 2.0 eq) and $RuCl_3 \cdot H_2O$ (1.1 mg, 5.5 µmol, 0.1 eq) in water (2.2 mL) was added at 0°C. The reaction was stirred for 2,5 h at this temperature and subsequently diluted with EtOAc and quenched with sat. aq. $N_2S_2O_3$. The layers were separated and the aqueous layer was extracted with EtOAc. The combined organic layers were washed with water and brine, dried over $MgSO_4$, filtered and concentrated *in vacuo*. The crude product was purified by silica gel flash column chromatography (0%→15% EtOAc in pentane) to obtain cyclosulfate **39** (26 mg, 48 µmol, 40% over 3 steps) as a colorless oil. ^1H NMR (500 MHz, $CDCl_3$) δ 7.34 – 7.27 (m, 15H, CH_{Ar}), 5.11 – 5.04 (m, 2H, H-1, H-6), 4.78 – 4.72 (m, 4H, 4x CH*H*Ph), 4.53 (s, 2H, 2x CH*H*Ph), 3.85 – 3.79 (m, 2H, H-3, H-7b), 3.71 – 3.66 (m, 1H, H-2), 3.56 (dd, *J* = 9.5, 2.2 Hz, 1H, H-7a), 3.52 (s, 3H, OCH_3), 3.28 (dd, *J* = 11.7, 8.2 Hz, 1H, H-4), 2.48 (ddt, *J* = 11.6, 9.5, 2.2 Hz, 1H, H-5). ^{13}C NMR (126 MHz, $CDCl_3$) δ 138.5, 138.0, 137.6 (3x C_{qAr}), 128.7, 128.6, 128.5, 128.4, 128.3, 128.11, 128.08, 128.06, 128.0 (CH_{Ar}), 82.0 (C-3), 81.2 (C-1), 80.3 (C-6), 78.1 (C-4), 76.1 (C-2), 75.2, 73.9, 73.3 (3x CH_2Ph), 64.0 (C-7), 60.7 (OCH_3), 43.1 (C-5). HRMS (ESI) m/z: $[M+Na^+]$ calcd for $C_{29}H_{32}O_8SNa$ 563.1716, found 563.1717.

(3a*R*,4*R*,5*S*,6*R*,7*R*,7a*S*)-4,6-dihydroxy-7-(hydroxymethyl)-5-methoxyhexahydrobenzo[*d*][1,3,2]dioxathiole 2,2-dioxide (6) Cyclosulfate **39** (21 mg, 39 µmol, 1.0 eq) was dissolved in MeOH/THF (1 mL) and purged with

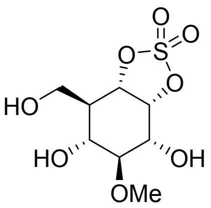

N_2. Pd/C (10 wt%, 17 mg, 16 µmol, 0.4 eq) was added to the solution and the reaction mixture was again purged with N_2. The reaction mixture was flushed for 5 min with H_2 before being left to stir under H_2 atmosphere for 5 h. The reaction mixture was flushed with N_2 and filtered over whatman filter paper. The filtrate was concentrated *in vacuo* and the crude material was purified by silica gel flash column chromatography (0%→20% MeOH in DCM, silica prewashed with MeOH) to obtain **6** (10 mg, 37 µmol, 94%) as a white solid. ^1H NMR (500 MHz, MeOD) δ 4.91 (dd, J = 10.2, 5.3 Hz, 1H, H-6), 4.02 (dd, J = 11.2, 2.3 Hz, 1H, H-7A), 3.96 (dd, J = 5.3, 3.4 Hz, 1H, H-1), 3.69 (dd, J = 8.5, 2.7 Hz, 1H, H-7B), 3.68 – 3.65 (m, 1H, H-3), 3.61 (dd, J = 9.8, 3.4 Hz, 1H, H-2), 3.41 (dd, J = 11.2, 8.7 Hz, 1H, H-4), 2.97 (s, 3H, CH_3), 2.23 (tt, J = 10.3, 2.5 Hz, 1H, H-5). ^{13}C NMR (126 MHz, MeOD) δ 80.1 (C-6), 76.2 (C-3), 72.9 (C-2), 70.1 (C-4), 65.3 (C-1), 57.8 (C-7), 47.9 (C-5), 35.1 (CH_3). HRMS (ESI) m/z: $[M+Na^+]$ calcd for $C_8H_{14}O_8SNa$ 293.2418, found 293.2419.

(4a*R*,7*S*,8*R*,8a*R*)-7,8-dimethoxy-2-phenyl-4a,7,8,8a-tetrahydro-4H-benzo[*d*][1,3]dioxine (40)

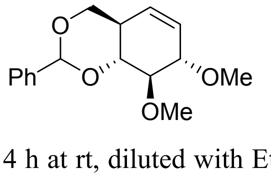

Compound **31** (62 mg, 0.25 mmol, 1.0 eq) was dissolved in anhydrous DMF (5 mL) and cooled to 0°C. NaH (60% dispersion in mineral oil, 30 mg, 0.75 mmol, 3.0 eq) was added and the reaction was stirred for 15 min at 0°C. Subsequently, MeI (39 µL, 0.63 mmol, 2.5 eq) was added dropwise to the solution. The reaction was stirred for 4 h at rt, diluted with Et_2O and quenched with MeOH at 0°C. The organic layer was washed with H_2O and brine (2x), dried over $MgSO_4$, filtered and concentrated *in vacuo*. The crude material was purified by silica gel flash column chromatography (10%→30% EtOAc in pentane) to obtain **40** (64 mg, 0.23 mmol, 91%) as a colorless oil. ^1H NMR (400 MHz, $CDCl_3$) δ 7.55 – 7.49 (m, 2H, CH_{Ar}), 7.40 – 7.33 (m, 3H, CH_{Ar}), 5.75 (dt, *J* = 9.9, 3.0 Hz, 1H, H-1), 5.60 (s, 1H, H-8), 5.40 (dt, *J* = 9.9, 1.8 Hz, 1H, H-6), 4.28 (dd, *J* = 10.8, 4.6 Hz, 1H, H-7b), 3.97 (dtd, *J* = 6.4, 3.1, 1.8 Hz, 1H, H-2), 3.74 – 3.60 (m, 6H, H-3, H-4 , H-7a, OCH_3), 3.49 (s, 3H, OCH_3), 2.71 – 2.61 (m, 1H, H-5). ^{13}C NMR (101 MHz, $CDCl_3$) δ 138.13 (C_{qAr}), 128.8, 128.3 (CH_{Ar}), 128.2 (C-1) 126.1 (CH_{Ar}), 125.4 (C-6), 101.6 (C-8), 83.1 (C-3), 82.2 (C-2/C-4), 82.1 (C-2/C-4), 70.0 (C-7),

60.5 (OCH_3), 57.2 (OCH_3), 38.4 (C-5). HRMS (ESI) m/z: [M+Na$^+$] calcd for $C_{16}H_{20}O_4Na$ 299.1259, found 299.1261.

(1*R*,2*R*,5*S*,6*S*)-2-(hydroxymethyl)-5,6-dimethoxycyclohex-3-en-1-ol (41) Compound **40** (55 mg, 0.2 mmol, 1.0 eq) was dissolved in DCM (1 mL) and cooled to 0 ℃. Water (0.39 mL) and TFA (77 µL, 1.0 mmol, 5.0 eq) were added and the reaction was stirred for 1.5 h at rt after which TLC analysis indicated full conversion of the starting material. The solution was washed with sat. aq. $NaHCO_3$, H_2O and brine, dried over $MgSO_4$, filtered and concentrated *in vacuo*. The crude product was purified by silica gel flash column chromatography (0%→ 10% MeOH in DCM) to obtain **41** (31 mg, 0.17 mmol, 83%) as a colorless oil. ^{1}H NMR (400 MHz, MeOD) δ 5.73 – 5.65 (m, 2H, H-1, H-6), 3.83 – 3.76 (m, 2H, H-2, H-7b), 3.61 (s, 3H, OCH_3), 3.61 – 3.57 (m, 1H, H-7A), 3.51 (t, *J* = 9.7 Hz, 1H, H-4), 3.44 (s, 3H, OCH_3), 3.19 (dd, *J* = 10.1, 7.8 Hz, 1H, H-3), 2.29 – 2.23 (m, 1H, H-5). ^{13}C NMR (101 MHz, MeOD) δ 129.7 (C-1/C-6), 127.5 (C-1/C-6), 86.9 (C-3), 83.1 (C-2), 71.6 (C-4), 63.2 (C-7), 60.8 (OCH_3), 57.0 (OCH_3), 47.7 (C-5). HRMS (ESI) m/z: [M+Na$^+$] calcd for $C_9H_{16}O_4Na$ 211.0946, found 211.0947.

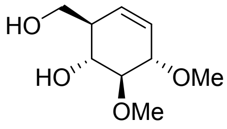

((((1*R*,4*S*,5*R*,6*R*)-6-(benzyloxy)-4,5-dimethoxycyclohex-2-en-1-yl)methoxy)methyl)benzene (42) Compound **41** (28 mg, 0.15 mmol, 1.0 eq) was dissolved in anhydrous DMF (3 mL) and cooled to 0°C. NaH (60% dispersion in mineral oil, 18 mg, 0.45 mmol, 3.0 eq) was added and the reaction was stirred for 15 min at 0°C. Subsequently, TBAI (3 mg, 7.5 µmol, 0.05 eq) was added followed by dropwise addition of BnBr (45 µL, 0.38 mmol, 2.5 eq). The reaction was stirred for 5 h at rt, diluted with Et_2O and quenched with MeOH at 0°C. The organic layer was washed with H_2O and brine (2x), dried over $MgSO_4$, filtered and concentrated *in vacuo*. The crude material was purified by silica gel flash column chromatography (5%→25% EtOAc in pentane) to obtain **42** (52 mg, 0.14 mmol, 94%) as a colorless oil. ^{1}H NMR (400 MHz, $CDCl_3$) δ 7.38 – 7.23 (m, 10H, CH_{Ar}), 5.71 – 5.64 (m, 2H, H-1, H-6), 4.89 (d, *J* = 10.9 Hz, 1H, CH*H*Ph), 4.49 – 4.38 (m, 3H, 3x CH*H*Ph), 3.91 (ddd, *J* = 7.8, 3.2, 1.8 Hz, 1H, H-2), 3.68 (s, 3H, OCH_3), 3.57 (t, *J* = 9.8 Hz, 1H, H-4), 3.51 (dd, *J* = 4.1, 1.6 Hz, 2H, H-7a, H-7b), 3.48 (s, 3H, OCH_3), 3.42 (dd, *J* = 10.1, 7.8 Hz, 1H, H-3), 2.47 (dddd, *J* = 9.1, 5.6, 2.8, 1.0 Hz, 1H, H-5). ^{13}C NMR (101 MHz, $CDCl_3$) δ 138.8, 138.4 (2x C_{qAr}), 129.4, 128.5, 128.3, 127.9, 127.78, 127.76, 126.5 (CH_{Ar}), 86.8 (C-3), 82.5 (C-2), 78.5 (C-4), 75.3, 73.2 (2x CH_2Ph), 69.2 (C-7), 60.9 (OCH_3), 57.2 (OCH_3), 44.3 (C-5). HRMS (ESI) m/z: [M+Na$^+$] calcd for $C_{23}H_{28}O_4Na$ 391.1885, found 391.1887.

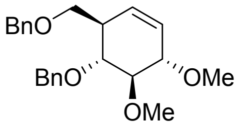

(1*S*,2*S*,3*S*,4*R*,5*S*,6*S*)-4-(benzyloxy)-3-((benzyloxy)methyl)-5,6-dimethoxycyclohexane-1,2-diol (43) and (1*R*,2*R*,3*S*,4*R*,5*S*,6*S*)-4-(benzyloxy)-3-((benzyloxy)methyl)-5,6-dimethoxycyclohexane-1,2-diol (44)
A solution of $NaIO_4$ (39 mg, 0.18 mmol, 1.5 eq) and $RuCl_3 \cdot H_2O$ (1.7 mg, 8.4 µmol, 0.07 eq) in water (1.0 mL) was added dropwise to an ice-cooled and vigorously stirred solution of cyclohexene **42** (44 mg, 0.12 mmol, 1.0 eq) in EtOAc/MeCN 1:1 (3.6 mL). The reaction was stirred for 2 h at 0°C after which TLC analysis indicated full conversion. The reaction was quenched with sat. aq. $Na_2S_2O_3$ and the aqueous layer was extracted with EtOAc (3x). The combined organic layers were washed with brine, dried over $MgSO_4$, filtered and concentrated *in vacuo*. The crude material was purified by silica gel flash column chromatography (5%→ 30% acetone in pentane) to obtain **43** (19 mg, 47 µmol, 39%) and **44** (14 mg, 35 µmol, 29%) as white solids.

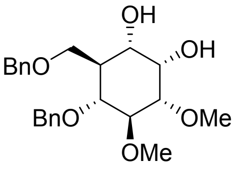

(**43**): ^{1}H NMR (400 MHz, $CDCl_3$) δ 7.37 – 7.24 (m, 10H, CH_{Ar}), 4.85 (d, *J* = 10.8 Hz, 1H, CH*H*Ph), 4.54 – 4.43 (m, 3H, 3x CH*H*Ph), 4.19 (t, *J* = 2.8 Hz, 1H, H-1), 3.85 (dd, *J* = 9.0, 2.7 Hz, 1H, H-7b), 3.63 (s, 5H, H-6, H-7a, OCH_3), 3.58 (t, *J* = 9.4 Hz, 1H, h-3), 3.51 (s, 3H, OCH_3), 3.25 (dd, *J* = 11.0, 9.2 Hz, 1H, H-4), 3.22 – 3.16 (s, 1H, OH), 3.05 (dd, *J* = 9.6, 2.8 Hz, 1H, H-2), 2.51 (s, 1H, OH), 2.20 – 2.12 (m, 1H, H-5). ^{13}C NMR (101 MHz, $CDCl_3$) δ 138.6, 138.1 (2x C_{qAr}), 128.7, 128.6, 128.5, 128.23, 128.20, 128.1, 127.91, 127.87, 127.8 (CH_{Ar}), 84.6 (C-3), 82.3 (C-2), 77.8 (C-4), 75.2 (CH_2Ph), 73.5 (CH_2Ph), 70.0 (C-6), 69.6 (C-1), 68.4 (C-7), 61.2 (OCH_3), 58.2 (OCH_3), 43.0 (C-5). HRMS (ESI) m/z: [M+H$^+$] calcd for $C_{23}H_{31}O_6$ 403.2121, found 403.2122.

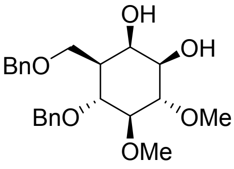

(**44**): ^{1}H NMR (400 MHz, $CDCl_3$) δ 7.38 – 7.24 (m, 10H, CH_{Ar}), 4.87 (d, *J* = 10.7 Hz, 1H, CH*H*Ph), 4.53 – 4.44 (m, 3H, 3x CH*H*Ph), 4.24 (t, *J* = 2.5 Hz, 1H, H-6), 3.88 (dd, *J* = 9.0, 5.5 Hz, 1H, H-7b), 3.78 (dd, *J* = 11.4, 9.1 Hz, 1H, H-4), 3.71 (dd, *J* = 9.0, 3.1 Hz, 1H, H-7a), 3.68 (s, 3H, OCH_3), 3.66 (s, 3H, OCH_3), 3.51 – 3.42 (m, 1H, H-2), 3.42 – 3.37 (m, 1H, H-1), 3.34 (s, 1H, 6-OH), 3.16 (t, *J* = 9.0 Hz, 1H, H-3), 2.50 (d, *J* = 4.9 Hz, 1H, 1-OH), 1.67 (dddd, *J* = 10.9, 5.4, 3.1, 2.0 Hz, 1H, H-5). ^{13}C NMR (101 MHz, $CDCl_3$) δ 138.6, 137.7 (2x C_{qAr}), 128.7, 128.6, 128.2, 128.1, 127.91, 127.89 (CH_{Ar}), 88.8 (C-3), 83.9 (C-2), 77.3 (C-4), 75.5 (CH_2Ph), 74.4 (C-1), 73.7 (CH_2Ph), 71.0 (C-6), 69.0 (C-7), 61.3 (OCH_3), 61.0 (OCH_3), 43.3 (C-5). HRMS (ESI) m/z: [M+H$^+$] calcd for $C_{23}H_{31}O_6$ 403.2121, found 403.2123.

(3a*S*,4*R*,5*R*,6*S*,7*R*,7a*R*)-5-(benzyloxy)-4-((benzyloxy)methyl)-6,7-dimethoxyhexahydrobenzo[*d*][1,3,2]dioxathiole 2,2-dioxide (45) $SOCl_2$ (9.4 µL, 0.13 mmol, 3.5 eq) was

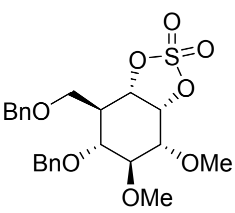

added dropwise over 5 min to a ice- cooled solution of diol **43** (15 mg, 37 µmol, 1.0 eq) and Et_3N (21 µL, 0.15 mmol, 4.0 eq) in DCM. The reaction was stirred for 1,5 h at 0°C after which TLC analysis indicated full conversion of the starting material. The reaction mixture was diluted with cold Et_2O and the organic layer was washed with cold water and brine, dried over $MgSO_4$, filtered and concentrated *in vacuo*. Final traces of Et_3N were removed under high vacuum. The crude material was dissolved in EtOAc/ACN 1:1 (3 mL) and a solution of $NaIO_4$ (16 mg, 74 µmol, 2.0 eq) and $RuCl_3 \cdot H_2O$ (1.0 mg, 3.7 µmol, 0.1 eq) in water (1.5 mL) was added at 0°C. The reaction was stirred for 2,5 h at this temperature and subsequently diluted with EtOAc and quenched with sat. aq. $N_2S_2O_3$. The layers were separated and the aqueous layer was extracted with EtOAc. The combined organic layers were washed with water and brine, dried over $MgSO_4$, filtered and concentrated *in vacuo*. The crude product was purified by silica gel flash column chromatography (0%→15% EtOAc in pentane) to obtain cyclosulfate **45** (13 mg, 27 µmol, 73%) as a colorless oil. ^{1}H NMR (400 MHz, $CDCl_3$) δ 7.37 – 7.24 (m, 10H, CH_{Ar}), 5.21 (dd, *J* = 5.1, 3.4 Hz, 1H, H-1), 5.11 (dd, *J* = 10.0, 5.1 Hz, 1H, H-6), 4.83 (d, *J* = 10.9 Hz, 1H, CH*H*Ph), 4.51 (d, *J* = 10.8 Hz, 1H, CH*H*Ph), 4.49 – 4.45 (m, 1H, CH*H*Ph), 4.42 (d, *J* = 11.7 Hz, 1H, CH*H*Ph), 3.86 (dd, *J* = 9.4, 2.2 Hz, 1H, H-7b), 3.60 (s, 3H, OCH_3), 3.59 – 3.54 (m, 5H, H-3, H-7a, OCH_3), 3.49 – 3.44 (m, 1H, H-4), 3.44 – 3.40 (m, 1H, H-2), 2.45 (ddt, *J* = 12.1, 10.1, 2.2 Hz, 1H, H-5). ^{13}C NMR (101 MHz, $CDCl_3$) δ 138.2, 137.7 (2x C_{qAr}), 128.7, 128.6, 128.1, 128.04, 128.02 (CH_{Ar}), 83.6 (C-3), 80.4 (C-1), 80.2 (C-6), 78.6 (C-4), 75.4 (C-2), 75.2, 73.5 (2x CH*H*Ph), 64.1 (C-7), 60.7 (OCH_3), 59.6 (OCH_3), 43.4 (C-5). HRMS (ESI) m/z: [M+Na$^+$] calcd for $C_{23}H_{28}O_8SNa$ 487.1403, found 487.1404.

(3a*S*,4*R*,5*R*,6*S*,7*R*,7a*R*)-5-hydroxy-4-(hydroxymethyl)-6,7-dimethoxyhexahydrobenzo[*d*][1,3,2]dioxathiole 2,2-dioxide (9) Cyclosulfate **45** (10 mg, 22 µmol, 1.0 eq) was dissolved in MeOH/THF 3:1 (1.0 mL) and purged with N_2. Pd/C (10 wt%, 9.4 mg, 8.8 µmol, 0.4 eq) was added to the solution and the reaction mixture was again purged with N_2. The reaction mixture was flushed for 5 min with H_2 before being left to stir under H_2 atmosphere for 4 h. The reaction mixture was flushed with N_2 and filtered over whatman filter paper. The filtrate was concentrated *in vacuo* and the crude material was purified by silica gel flash column chromatography (0%→15% MeOH in DCM) to obtain **9** (5.8 mg, 20 µmol, 93%) as a white solid. ^1H NMR (500 MHz, MeOD) δ 5.45 (dd, *J* = 4.6, 3.4 Hz, 1H, H-1), 5.15 (dd, *J* = 10.2, 4.6 Hz, 1H, H-6), 4.03 (dd, *J* = 11.2, 2.4 Hz, 1H, H-7b), 3.65 (dd, *J* = 10.9, 2.4 Hz, 1H, H-7a), 3.62 (s, 3H, OC*H*3), 3.55 (s, 3H, OC*H*3), 3.49 (dd, *J* = 9.3, 3.4 Hz, 1H, H-2), 3.45 (dd, *J* = 11.6, 9.0 Hz, 1H, H-4), 3.29 (d, *J* = 9.1 Hz, 1H, H-3), 2.13 (ddt, *J* = 11.4, 10.1, 2.5 Hz, 1H, H-5). ^{13}C NMR (126 MHz, MeOD) δ 84.6 (C-3), 82.8 (C-1), 82.6 (C-6), 79.6 (C-2), 68.2 (C-4), 61.4 (O*C*H3), 59.0 (O*C*H3), 57.0 (C-7), 46.6 (C-5). HRMS (ESI) m/z: [M+Na$^+$] calcd for $C_9H_{16}O_8SNa$ 307.0464, found 307.0465.

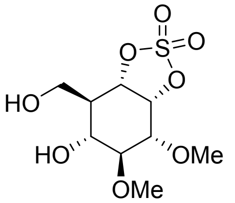

((1*R*,4*S*,5*S*,6*R*)-4,5-bis(benzyloxy)-6-methoxycyclohex-2-en-1-yl)methanol (46)

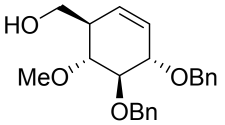

Cyclohexene **29** (0.17 g, 0.5 mmol, 1.0 eq) was dissolved in anhydrous DMF (5 mL) and TrtCl (0.17 g, 0.6 mmol, 1.2 eq) were added and the reaction mixture was stirred overnight rt. The reaction mixture was diluted with Et_2O and the organic layer was washed with sat. aq. $NaHCO_3$, water and brine, dried over Na_2SO_4 and concentrated *in vacuo*. Final traces of Et_3N were removed under high vacuum and the crude material was used without further purification. The obtained oil was dissolved in anhydrous DMF (10 mL) and cooled to 0°C. NaH (60% dispersion in mineral oil, 30 mg, 0.75 mmol, 1.5 eq) was added and the solution was stirred for 15 min at 0 °C. MeI (37 µL, 0.6 mmol, 1.2 eq) was added dropwise and the reaction was stirred for 3 h at rt. The reaction was diluted with Et_2O and quenched with MeOH at 0°C. The organic layer was washed with water and brine (2x), dried over $MgSO_4$, filtered and concentrated *in vacuo*. The obtained crude oil was dissolved in DCM/MeOH 1:3 (2.5 mL) and *p*-TsOH (29 mg, 0.15 mmol, 0.3 eq) was added. The reaction was stirred for 4 h at rt and quenched with Et_3N until pH 6-7 was reached. The reaction mixture was diluted with DCM and the organic layer was washed with sat. aq. $NaHCO_3$ and brine. The crude material was purified by silica gel flash column chromatography (5%→25 EtOAc in pentane) to obtain **46** (0.11 g, 0.31 mmol, 62% over 3 steps) as a colorless oil. ^1H NMR (500 MHz, $CDCl_3$) δ 7.41 – 7.37 (m, 2H, CH_{Ar}), 7.36 – 7.26 (m, 8H, CH_{Ar}), 5.74 (ddd, *J* = 10.2, 2.9, 2.2 Hz, 1H, H-6), 5.53 (dt, *J* = 10.1, 2.1 Hz, 1H, H-1), 4.88 (s, 2H, 2x CH*H*Ph), 4.71 – 4.64 (m, 2H, 2x CH*H*Ph), 4.18 (ddt, *J* = 7.6, 3.4, 2.1 Hz, 1H, H-2), 3.78 – 3.71 (m, 3H, H-7a, H-7b, H-3), 3.62 (s, 3H, OCH_3), 3.39 (dd, *J* = 10.0, 9.2 Hz, 1H, H-4), 2.46 (dddd, *J* = 11.4, 7.0, 3.2, 1.5 Hz, 1H, H-5), 2.08 (s, 1H, 7-OH). ^{13}C NMR (126 MHz, $CDCl_3$) δ 138.9, 138.5 (2x C_{qAr}), 128.53 (C-6), 128.49, 128.4, 128.1, 128.0, 127.82, 127.78 (CH_{Ar}), 127.7 (C-1), 84.8 (C-3), 82.0 (C-4), 80.6 (C-2), 75.2, 72.2 (2x *C*H2Ph), 64.0 (C-7), 61.0 (O*C*H3), 45.7 (C-5). HRMS (ESI) m/z: [M+Na$^+$] calcd for $C_{22}H_{26}O_4Na$ 377.1729, found 377.1731.

((((1*S*,2*S*,5*R*,6*R*)-5-((benzyloxy)methyl)-6-methoxycyclohex-3-ene-1,2-diyl)bis(oxy))bis(methylene))dibenzene (47) Compound **46** (0.1 g, 0.28 mmol, 1.0 eq) was dissolved in

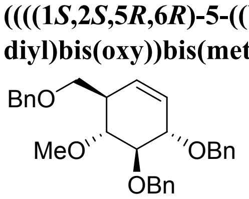

anhydrous DMF (5.6 mL) and cooled to 0°C. NaH (60% dispersion in mineral oil, 17 mg, 0.42 mmol, 1.5 eq) was added and the reaction was stirred for 15 min at 0°C. Subsequently, TBAI (5.2 mg, 14 µmol, 0.05 eq) was added followed by dropwise addition of BnBr (40 µL, 0.34 mmol, 1.2 eq). The reaction was stirred for 2 h at rt, diluted with Et_2O and quenched with MeOH at 0°C. The organic layer was washed with H_2O and brine (2x), dried over $MgSO_4$, filtered and concentrated *in vacuo*. The crude material was purified by silica gel flash column chromatography (5%→15% EtOAc in pentane) to obtain **47** (0.12 g, 0.27 mmol, 95%) as a colorless oil. ^1H NMR (500 MHz, $CDCl_3$) δ 7.41 – 7.37 (m, 2H, CH_{Ar}), 7.37 – 7.24 (m, 13H, CH_{Ar}), 5.72 – 5.68 (m, 1H, H-6), 5.66 (dt, *J* = 10.2, 1.7 Hz, 1H, H-1), 4.88 (s, 1H, CH*H*Ph), 4.88 (s, 1H, CH*H*Ph), 4.68 (s, 1H, CH*H*Ph), 4.68 (s, 1H, CH*H*Ph), 4.58 – 4.54 (m, 1H, CH*H*Ph), 4.49 (d, *J* = 12.2 Hz, 1H, CH*H*Ph), 4.19 (ddd, *J* = 7.8, 3.5, 1.7 Hz, 1H, H-2), 3.71 (dd, *J* = 10.1, 7.8 Hz, 1H, H-3), 3.58 (dd, *J* = 9.0, 3.4 Hz, 1H, H-7b), 3.55 (dd, *J* = 9.0, 5.0 Hz, 1H, H-7a), 3.50 (s, 3H, OCH_3), 3.38 (t, *J* = 9.8 Hz, 1H, H-4), 2.48 – 2.42 (m, 1H, H-5).

^{13}C NMR (126 MHz, $CDCl_3$) δ 139.1, 138.7, 138.4 (3x C_{qAr}), 129.2 (C-1), 128.48, 128.45, 128.1, 128.0, 127.9, 127.8, 127.7, 127.6 (CH_{Ar}), 127.0 (C-6), 85.3 (C-3), 80.8 (C-2), 80.6 (C-4), 75.3, 73.3, 72.2 (3x CH_2Ph), 69.5 (C-7), 61.1 (OCH_3), 44.5 (C-5). HRMS (ESI) m/z: [M+Na$^+$] calcd for $C_{29}H_{32}O_4Na$ 467.2198, found 467.2200.

(3a*R*,4*R*,5*S*,6*R*,7*R*,7a*S*)-4,5-bis(benzyloxy)-7-((benzyloxy)methyl)-6-methoxyhexahydrobenzo[*d*][1,3,2]dioxathiole 2,2-dioxide (50) A solution of $NaIO_4$ (0.13 g, 0.63 mmol, 2.5 eq) and $RuCl_3 \cdot H_2O$ (3.7 mg, 18 µmol, 0.07 eq) in water (2.0 mL) was added dropwise to an ice-cooled and vigorously stirred solution of cyclohexene **47** (0.11 g, 0.25 mmol, 1.0 eq) in EtOAc/MeCN 1:1 (7.5 mL). The reaction was stirred for 2 h at 0°C after which TLC analysis indicated full conversion. The reaction was quenched with sat. aq. $Na_2S_2O_3$ and the aqueous layer was extracted with EtOAc (3x). The combined organic layers were washed with brine, dried over $MgSO_4$, filtered and concentrated *in vacuo*. The crude material was purified by silica gel flash column chromatography (5%→30% acetone in pentane) to obtain **48** as white solid. $SOCl_2$ (25 µL, 0.35 mmol, 3.5 eq) was added dropwise over 5 min to an ice- cooled solution of diol **48** (48 mg, 0.1 mmol, 1.0 eq) and Et_3N (56 mg, 0.4 mmol, 4.0 eq) in DCM (5 mL). The reaction was stirred for 1,5 h at 0°C after which TLC analysis indicated full conversion of the starting material. The reaction mixture was diluted with cold Et_2O and the organic layer was washed with cold water and brine, dried over $MgSO_4$, filtered and concentrated *in vacuo*. Final traces of Et_3N were removed under high vacuum. The crude material was dissolved in EtOAc/MeCN 1:1 (8.0 mL) and a solution of $NaIO_4$ (43 mg, 0.2 mmol, 2.0 eq) and $RuCl_3 \cdot H_2O$ (2.1 mg, 10 µmol, 0.1 eq) in water (4.0 mL) was added at 0°C. The reaction was stirred for 2,5 h at this temperature and subsequently diluted with EtOAc and quenched with sat. aq. $N_2S_2O_3$. The layers were separated and the aqueous layer was extracted with EtOAc. The combined organic layers were washed with water and brine, dried over $MgSO_4$, filtered and concentrated *in vacuo*. The crude product was purified by silica gel flash column chromatography (0%→15% EtOAc in pentane) to obtain cyclosulfate **50** (39 mg, 73 µmol, 29% over 3 steps) as a colorless oil. ^1H NMR (500 MHz, $CDCl_3$) δ 7.37 – 7.26 (m, 15H, CH_{Ar}), 5.07 – 5.02 (m, 2H, H-1, H-6), 4.75 – 4.70 (m, 4H, 4x CH*H*Ph), 4.51 (s, 2H, 2x CH*H*Ph), 3.83 – 3.77 (m, 2H, H-3, H-7b), 3.68 – 3.64 (m, 1H, H-2), 3.57 (dd, J = 9.5, 2.2 Hz, 1H, H-7a), 3.48 (s, 3H, OCH_3), 3.23 (dd, J = 11.7, 8.2 Hz, 1H, H-4), 2.41 (ddt, J = 11.6, 9.5, 2.2 Hz, 1H, H-5). ^{13}C NMR (126 MHz, $CDCl_3$) δ 138.0, 137.8, 137.1 (3x C_{qAr}), 128.8, 128.62, 128.61, 128.4, 128.3, 128.11, 128.09, 128.05, 128.0 (CH_{Ar}), 81.8 (C-3), 81.0 (C-1), 80.1 (C-6), 77.3 (C-4), 75.7 (C-2), 75.0, 73.8, 73.5 (3x CH_2Ph), 64.2 (C-7), 60.9 (OCH_3), 43.4 (C-5). HRMS (ESI) m/z: [M+Na$^+$] calcd for $C_{29}H_{32}O_8SNa$ 563.1716, found 563.1717.

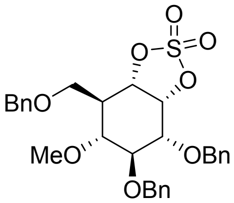

(3a*R*,4*R*,5*R*,6*R*,7*R*,7a*S*)-4,5-dihydroxy-7-(hydroxymethyl)-6-methoxyhexahydrobenzo[*d*][1,3,2]dioxathiole 2,2-dioxide (7) Cyclosulfate **50** (35 mg, 65 µmol, 1.0 eq) was dissolved in MeOH/THF 4:1 (1.0 mL) and purged with N_2. Pd/C (10 wt%, 28 mg, 26 µmol, 0.4 eq) was added to the solution and the reaction mixture was again purged with N_2. The reaction mixture was flushed for 5 min with H_2 before being left to stir under H_2 atmosphere for 5 h. The reaction mixture was flushed with N_2 and filtered over whatman filter paper. The filtrate was concentrated *in vacuo* and the crude material was purified by silica gel flash column chromatography (0%→20% MeOH in DCM, silica prewashed with MeOH) to obtain **50** (17 mg, 63 µmol, 97%) as a white solid. ^1H NMR (500 MHz, MeOD) δ 5.26 (dd, J = 4.5, 2.9 Hz, 1H, H-1), 5.18 (dd, J = 10.2, 4.5 Hz, 1H, H-6), 3.91 (dd, J = 11.1, 2.3 Hz, 1H, H-7b), 3.73 – 3.64 (m, 3H, H-2, H-3, H-7a), 3.60 (s, 3H, OCH_3), 3.10 (ddd, J = 11.4, 7.8, 1.1 Hz, 1H, H-4), 2.08 (ddt, J = 11.4, 10.2, 2.3 Hz, 1H, H-5). ^{13}C NMR (126 MHz, MeOD) δ 85.8 (C-1), 82.8 (C-6), 78. (C-5), 74.8 (C-3), 70.5 (C-2), 61.0 (OCH_3), 56.8 (C-7), 46.1 (C-5). HRMS (ESI) m/z: [M+Na$^+$] calcd for $C_8H_{14}O_8SNa$ 293.2418, found 293.2419.

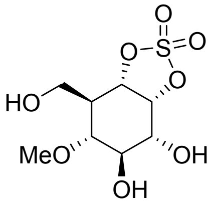

((1*R*,4*S*,5*R*,6*R*)-4,5,6-tris(benzyloxy)cyclohex-2-en-1-yl)methanol (51)

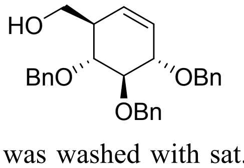

Cyclohexene **29** (0.17 g, 0.5 mmol, 1.0 eq) was dissolved in anhydrous DMF (5 mL) and Et_3N (0.14 mL, 1.0 mmol, 2.0 eq). Subsequently, DMAP (12 mg, 0.1 mmol, 0.1 eq) and TrtCl (0.17 g, 0.6 mmol, 1.2 eq) were added and the reaction mixture was stirred overnight rt. The reaction mixture was diluted with Et_2O and the organic layer was washed with sat. aq. $NaHCO_3$, water and brine, dried over Na_2SO_4 and concentrated *in vacuo*. Final traces of Et_3N were removed under high vacuum and the crude material was used without further purification. The obtained oil was dissolved in anhydrous DMF (10 mL) and cooled to 0°C. NaH (60% dispersion in mineral oil, 30 mg, 0.75 mmol 1.5 eq) was added and the solution was stirred for 15 min at 0°C. TBAI (9.2 mg, 25 μmol, 0.05 eq) was added followed by dropwise addition of BnBr (71 μL, 0.6 mmol, 1.2 eq) and the reaction was stirred for 6 h at rt. The reaction was diluted with Et_2O and quenched with MeOH at 0°C. The organic layer was washed with water and brine (2x), dried over $MgSO_4$, filtered and concentrated *in vacuo*. The obtained crude oil was dissolved in DCM/MeOH 1:3 2.5 mL and *p*TsOH (29 mg, 0.15 mmol, 0.3 eq) was added. The reaction was stirred for 4 h at rt and quenched with Et_3N until pH6-7 was reached. The reaction mixture was diluted with DCM and the organic layer was washed with sat. aq. $NaHCO_3$ and brine. The crude material was purified by silica gel flash column chromatography (5%→25 EtOAc in pentane) to obtain cyclohexene **51** (0.15 g, 0.36 mmol, 71% over 3 steps) as a colorless oil. ^1H NMR (500 MHz, $CDCl_3$) δ 7.39 – 7.24 (m, 15H, CH_{Ar}), 5.75 (ddd, *J* = 10.2, 2.9, 2.2 Hz, 1H, H-6), 5.54 (dt, *J* = 10.1, 2.0 Hz, 1H, H-1), 4.99 (d, *J* = 11.2 Hz, 1H, CH*H*Ph), 4.94 (d, *J* = 11.1 Hz, 1H, CH*H*Ph), 4.91 (d, *J* = 11.1 Hz, 1H, CH*H*Ph), 4.71 – 4.62 (m, 3H, 3x CH*H*Ph), 4.23 (ddt, *J* = 7.6, 3.4, 2.0 Hz, 1H, H-2), 3.84 (dd, *J* = 10.1, 7.7 Hz, 1H, H-3), 3.69 – 3.59 (m, 3H, H-4, H-7A, H-7B), 2.52 – 2.43 (m, 1H, H-5), 1.62 (d, *J* = 39.7 Hz, 1H, 7-OH). ^{13}C NMR (126 MHz, $CDCl_3$) δ 138.9, 138.5, 138.4 (3x C_{qAr}), 128.6, 128.53, 128.49, 128.4 (CH_{Ar}), 128.30 (C-1/C-6), 128.25 (C-1/C-6), 128.02, 127.98, 127.8, 127.7 (CH_{Ar}), 85.2 (C-3), 80.9 (C-2), 78.7 (C-4), 75.3, 75.2, 72.2 (3x CH_2Ph), 63.3 (C-7), 45.9 (C-5). HRMS (ESI) m/z: [$M+Na^+$] calcd for $C_{28}H_{30}O_4Na$ 453.2042, found 453.2043.

((((1*R*,2*R*,3*S*,6*R*)-6-(methoxymethyl)cyclohex-4-ene-1,2,3-triyl)tris(oxy))tris(methylene))tribenzene

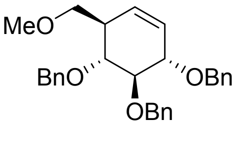

(52) Cyclohexene **51** (0.13 g, 0.3 mmol, 1.0 eq) was dissolved in anhydrous DMF (6 ml) and cooled to 0°C. NaH (60% dispersion in mineral oil, 18 mg, 0.45 mmol, 1.5 eq) was added and the reaction was stirred for 15 min at 0°C. Subsequently, MeI (22 μL, 0.36 mmol, 1.2 eq) was added dropwise. The reaction was stirred for 2 h at rt, diluted with Et_2O and quenched with MeOH at 0°C. The organic layer was washed with H_2O and brine (2x), dried over $MgSO_4$, filtered and concentrated *in vacuo*. The crude material was purified by silica gel flash column chromatography (5%→15% EtOAc in pentane) to obtain **52** (0.12 g, 0.28 mmol, 92%) as a colorless oil. ^1H NMR (500 MHz, $CDCl_3$) δ 7.38 – 7.24 (m, 15H, CH_{Ar}), 5.72 (ddd, *J* = 10.2, 2.8, 2.1 Hz, 1H, H-6), 5.63 (dt, *J* = 10.1, 2.0 Hz, 1H, H-1), 4.96 (d, *J* = 11.1 Hz, 1H, CH*H*Ph), 4.92 (s, 2H, 2x CH*H*Ph), 4.69 (s, 2H, 2x CH*H*Ph), 4.59 (d, *J* = 11.1 Hz, 1H, CH*H*Ph), 4.25 (ddt, *J* = 7.6, 3.7, 2.0 Hz, 1H, H-2), 3.81 (dd, *J* = 10.1, 7.8 Hz, 1H, H-3), 3.66 (t, *J* = 9.9 Hz, 1H, H-4), 3.43 (d, *J* = 3.9 Hz, 2H, H-7a, H-7b), 3.24 (s, 3H, OCH_3), 2.53 – 2.47 (m, 1H, H-5). ^{13}C NMR (126 MHz, $CDCl_3$) δ 139.0, 138.8, 138.6 (3x C_{qAr}), 129.2 (C-1), 128.51, 128.49, 128.4, 128.2, 128.01, 127.98, 127.8, 127.7, 127.6 (CH_{Ar}), 127.2 (C-6), 85.5 (C-3), 80.9 (C-2), 78.3 (C-4), 75.5, 75.4, 72.2 (3x CH_2Ph), 72.0 (C-7), 59.1 (OCH_3), 44.5 (C-5). HRMS (ESI) m/z: [$M+Na^+$] calcd for $C_{29}H_{32}O_4Na$ 467.2198, found 467.2199.

(1*S*,2*S*,3*S*,4*S*,5*R*,6*S*)-3,4,5-tris(benzyloxy)-6-(methoxymethyl)cyclohexane-1,2-diol (53) and (1*R*,2*R*,3*S*,4*S*,5*R*,6*S*)-3,4,5-tris(benzyloxy)-6-(methoxymethyl)cyclohexane-1,2-diol (54)

A solution of $NaIO_4$ (0.13 g, 0.63 mmol, 2.5 eq) and $RuCl_3 \cdot H_2O$ (3.7 mg, 18 μmol, 0.07 eq) in water (2.0 mL) was added dropwise to an ice-cooled and vigorously stirred solution of cyclohexene **52** (0.11 g, 0.25 mmol, 1.0 eq) in EtOAc/MeCN 1:1 (7.5 mL). The reaction was stirred for 2 h at 0°C after which TLC analysis indicated full conversion. The reaction was quenched with sat. aq. $Na_2S_2O_3$ and the aqueous layer was extracted with EtOAc (3x). The combined organic layers were washed with brine, dried over $MgSO_4$, filtered and concentrated *in vacuo*. The crude material was purified by silica gel flash column chromatography (5%→ 30% acetone in pentane) to obtain diols **53** (47 mg, 98 μmol, 39%) and **54** (38 mg, 80 μmol, 32%) as white solids.

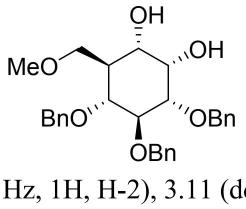

(**53**): ^{1}H NMR (400 MHz, $CDCl_3$) δ 7.39 – 7.26 (m, 15H, CH_{Ar}), 4.89 (d, *J* = 10.8 Hz, 1H, CH*H*Ph), 4.81 (d, *J* = 10.8 Hz, 1H, CH*H*Ph), 4.70 (s, 1H, CH*H*Ph), 4.70 (s, 1H, CH*H*Ph), 4.56 (d, *J* = 11.8 Hz, 1H, CH*H*Ph), 4.51 (d, *J* = 11.8 Hz, 1H, CH*H*Ph), 4.12 (t, *J* = 2.8 Hz, 1H, H-1), 3.90 – 3.81 (m, 2H, H-3, H-7b), 3.69 (dd, *J* = 9.0, 5.2 Hz, 1H, H-7a), 3.66 – 3.60 (m, 1H, H-6), 3.50 (s, 3H, OCH_3), 3.36 (dd, *J* = 9.6, 2.8 Hz, 1H, H-2), 3.11 (dd, *J* = 11.0, 9.3 Hz, 1H, H-4), 3.05 (d, *J* = 6.2 Hz, 1H, 6-OH), 2.59 (s, 1H, 1-OH), 2.10 (tdd, *J* = 10.9, 5.2, 2.6 Hz, 1H, H-5). ^{13}C NMR (101 MHz, $CDCl_3$) δ 138.9, 138.1, 138.0 (3x C_{qAr}), 128.5, 128.43, 128.37, 128.0, 127.93, 127.89, 127.7, 127.64, 127.57 (CH_{Ar}), 82.7 (C-3), 79.9 (C-2), 79.6 (C-4), 75.6, 73.4, 72.6 (3x CH_2Ph), 70.4 (C-1), 69.4 (C-6), 67.9 (C-7), 60.9 (OCH_3), 43.2 (C-5). HRMS (ESI) m/z: [M+Na^+] calcd for $C_{29}H_{34}O_6Na$ 501.2253, found 467.2254.

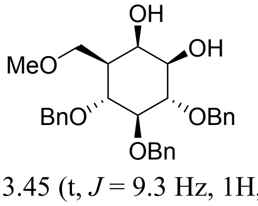

(**54**): ^{1}H NMR (500 MHz, $CDCl_3$) δ 7.39 – 7.26 (m, 15H, CH_{Ar}), 4.93 (d, *J* = 11.2 Hz, 1H, CH*H*Ph), 4.87 (s, 2H, 2x CH*H*Ph), 4.76 (d, *J* = 11.2 Hz, 1H, CH*H*Ph), 4.57 (d, *J* = 11.8 Hz, 1H, CH*H*Ph), 4.54 (d, *J* = 11.8 Hz, 1H, CH*H*Ph), 4.23 (t, *J* = 2.5 Hz, 1H, H-6), 3.90 (dd, *J* = 9.0, 5.8 Hz, 1H, H-7b), 3.80 – 3.72 (m, 2H, H-2, H-7a), 3.61 (dd, *J* = 11.3, 9.1 Hz, 1H, H-4), 3.51 (s, 3H, OCH_3), 3.49 (d, *J* = 10.2 Hz, 1H, H-1), 3.45 (t, *J* = 9.3 Hz, 1H, H-3), 3.23 (s, 1H, 6-OH), 2.38 (s, 1H, 1-OH), 1.66 (dddd, *J* = 11.2, 5.6, 3.1, 2.1 Hz, 1H, H-5). ^{13}C NMR (126 MHz, $CDCl_3$) δ 138.83, 138.80, 137.8 (3x C_{qAr}), 128.69, 128.67, 128.5, 128.14, 128.05, 128.0, 127.9, 127.8, 127.7 (CH_{Ar}), 86.5 (C-3), 82.3 (C-2), 79.4 (C-4), 75.8, 75.6 (2x CH_2Ph), 74.6 (C-1), 73.7 (CH_2Ph), 70.9 (C-6), 68.9 (C-7), 61.2 (OCH_3), 43.6 (C-5). HRMS (ESI) m/z: [M+Na^+] calcd for $C_{29}H_{34}O_6Na$ 501.2253, found 467.2254.

(3a*R*,4*R*,5*S*,6*R*,7*R*,7a*S*)-4,5,6-tris(benzyloxy)-7-(methoxymethyl)hexahydrobenzo[*d*][1,3,2]dioxathiole 2,2-dioxide (55) $SOCl_2$ (21 µL, 0.29 mmol, 3.5 eq) was added dropwise over 5 min to an ice- cooled solution of diol **53** (40 mg, 84 µg, 1.0 eq) and Et_3N (47 µL, 0.34 mmol, 4.0 eq) in DCM (4.2 mL). The reaction was stirred for 1,5 h at 0°C after which TLC analysis indicated full conversion of the starting material. The reaction mixture was diluted with cold Et_2O and the organic layer was washed with cold water and brine, dried over $MgSO_4$, filtered and concentrated *in vacuo*. Final traces of Et_3N were removed under high vacuum. The crude material was dissolved in EtOAc/MeCN 1:1 (6.7 mL) and a solution of $NaIO_4$ (36 mg, 0.17 mmol, 2.0 eq) and $RuCl_3 \cdot H_2O$ (1.7 mg, 8.4 µmol, 0.1 eq) in water was added at 0°C. The reaction was stirred for 2,5 h at this temperature and subsequently diluted with EtOAc and quenched with sat. aq. $N_2S_2O_3$. The layers were separated and the aqueous layer was extracted with EtOAc. The combined organic layers were washed with water and brine, dried over $MgSO_4$, filtered and concentrated *in vacuo*. The crude product was purified by silica gel flash column chromatography (0%→15% EtOAc in pentane) to obtain cyclosulfate **55** (34 mg, 62 µmol, 74%) as a colorless oil. ^{1}H NMR (400 MHz, $CDCl_3$) δ 7.39 – 7.26 (m, 15H, CH_{Ar}), 5.08 – 5.00 (m, 2H, H-1, H-6), 4.82 (d, *J* = 11.0 Hz, 1H, CH*H*Ph), 4.79 – 4.73 (m, 3H, 3x CH*H*Ph), 4.71 (d, *J* = 10.9 Hz, 1H, CH*H*Ph), 4.54 (d, *J* = 11.0 Hz, 1H, CH*H*Ph), 3.91 (t, *J* = 7.9 Hz, 1H, H-3), 3.72 (dq, *J* = 7.6, 2.6 Hz, 2H, H-2, H-7b), 3.50 – 3.45 (m, 1H, H-4)), 3.45 – 3.41 (m, 1H, H-7a), 3.27 (s, 3H, OCH_3), 2.49 (ddt, *J* = 11.7, 9.5, 2.2 Hz, 1H, H-5). ^{13}C NMR (101 MHz, $CDCl_3$) δ 138.0, 137.8, 137.0 (C_{qAr}), 128.7, 128.52, 128.49, 128.3, 128.2, 127.97, 127.95, 127.89, 127.85 (CH_{Ar}), 81.8 (C-3), 80.9 (C-1), 79.8 (C-6), 75.6 (C-2), 75.3 (C-4), 75.1, 74.9, 73.7 (3x CH_2Ph), 66.3 (C-7), 58.9 (OCH_3), 43.3 (C-5). HRMS (ESI) m/z: [M+Na^+] calcd for $C_{29}H_{34}O_8SNa$ 563.1716, found 563.1717.

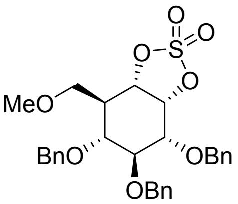

(3a*R*,4*R*,5*S*,6*R*,7*R*,7a*S*)-4,5,6-trihydroxy-7-(methoxymethyl)hexahydrobenzo[*d*][1,3,2]dioxathiole 2,2-dioxide (8) Cyclosulfate **55** (30 mg, 55 µmol, 1.0 eq) was dissolved in MeOH/THF 3:1 (2 mL) and purged with N_2. Pd/C (10 wt%, 23 mg, 22 µmol, 0.4 eq) was added to the solution and the reaction mixture was again purged with N_2. The reaction mixture was flushed for 5 min with H_2 before being left to stir under H_2 atmosphere for 5 h. The reaction mixture was flushed with N_2 and filtered over whatman filter paper. The filtrate was concentrated *in vacuo* and the crude material was purified by silica gel flash column chromatography (0%→20% MeOH in DCM, silica prewashed with MeOH) to obtain **8** (14 mg, 51 µmol, 93%) as a white solid. ^{1}H NMR (500 MHz, MeOD) δ 5.28 – 5.24 (m, 1H, H-1), 5.17 (dd, *J* = 10.2, 4.4 Hz, 1H, H-6), 3.81 (dd, *J* = 9.5, 2.5 Hz, 1H, H-7b), 3.66 (dd, *J* = 9.9,

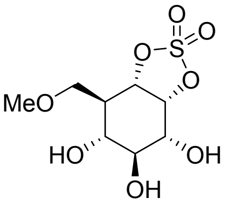

3.5 Hz, 1H, H-2), 3.56 (dd, J = 9.9, 9.2 Hz, 1H, H-3), 3.48 (dd, J = 9.5, 2.5 Hz, 1H, H-7a), 3.38 (s, 3H, OCH_3), 3.36 – 3.34 (m, 1H, H-4), 2.16 (ddt, J = 11.3, 10.1, 2.5 Hz, 1H, H-5). ^{13}C NMR (126 MHz, MeOD) δ 86.2 (C-1), 83.3 (C-6), 74.7 (C-3), 70.4 (C-2), 68.9 (C-4), 67.7 (C-7), 59.4 (OCH_3), 45.8 (C-5). HRMS (ESI) m/z: [M+Na$^+$] calcd for $C_8H_{14}O_8SNa$ 293.2418, found 293.2419.

***N*-(Naphtalenyl-2-methoxy)-pentyl-1,2-Dideoxy-1,2-azabicyclo[4.1.0]-carba-α-D-glucose (13).**

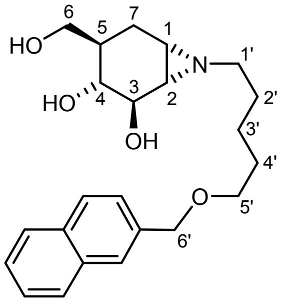

Compound **56** (16 mg, 0.1 mmol), prepared according to literature procedures[1–4], was dissolved in anhydrous DMF (2.0 mL, 0.05 M) followed by the addition of naphtalenyl-2-methoxypentyl iodide (71 mg, 0.2 mmol, 2.0 eq.), prepared according to literature procedures[3,4], and K_2CO_3 (138 mg, 0.3 mmol, 3.0 eq.). The reaction was stirred for 3 hours at 100 °C under protective atmosphere. Upon full conversion was observed (R_f 0.7 (MeOH:DCM, 2:8, v:v), the reaction mixture was concentrated and purified by flash column chromatography (2:98 MeOH:DCM → 8:92 MeOH:DCM). A second flash column purification (40:60 acetone:DCM → 60:40 acetone:DCM) yielded the title compound (17.0 mg, 44 µmol, 44%) as a colorless oil. ^{1}H NMR (500 MHz, MeOD, HH-COSY, HSQC): δ 7.86 – 7.78 (m, 4H, CH_{arom}), 7.51 – 7.43 (m, 3H, CH_{arom}), 4.65 (d, J = 0.8 Hz, 2H, H-6'), 3.60 – 3.50 (m, 5H, H-3, H-6, H-5'), 3.07 (dd, J = 10.9, 8.2 Hz, 1H, H-4), 2.32 (dt, J = 11.7, 7.3 Hz, 1H, H-1'), 2.21 (dt, J = 11.7, 7.4 Hz, 1H, H-1'), 2.04 (ddd, J = 14.0, 4.4, 1.2 Hz, 1H, H-7), 1.75 (ddd, J = 6.4, 3.4, 1.2 Hz, 1H, H-2), 1.69 – 1.53 (m, 6H, H-1, H-7, H-2', H-4'), 1.52 – 1.40 (m, 3H, H-5, H-3'); ^{13}C NMR (126 MHz, MeOD, HSQC): δ 137.4, 134.8, 134.5 ($C_{q\text{-}arom}$), 129.1, 128.9, 128.7, 127.5, 127.2, 126.9 (CH_{arom}), 76.3 (C-4), 74.9 (C-3), 74.0 (C-6'), 71.3, 64.5 (C-6, C-5'), 61.6 (C-1'), 45.6 (C-1), 40.9 (C-2), 37.5 (C-5), 30.7, 30.3 (C-2', C-4'), 28.2 (C-7), 25.1 (C-3'); HRMS (ESI) m/z: [M+Na$^+$] calcd for $C_{23}H_{31}NO_4Na$ 408.2151, found 408.2153.

***N*-(Biphenyl-4-methoxy)-pentyl-1,2-Dideoxy-1,2-azabicyclo[4.1.0]-carba-α-D-glucose (14).**

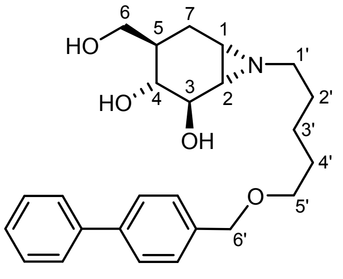

Compound **56** (16 mg, 0.1 mmol), prepared according to literature procedures[1–4], was dissolved in anhydrous DMF (2.0 mL, 0.05 M) followed by the addition of 1,1'-biphenyl-4-methoxypentyl iodide (76 mg, 0.2 mmol, 2.0 eq.), prepared according to literature procedures[3,4], and K_2CO_3 (138 mg, 0.3 mmol, 3.0 eq.). The reaction was stirred for 3 hours at 100 °C under protective atmosphere. Upon full conversion was observed (R_f 0.7 (MeOH:DCM, 2:8, v:v), the reaction mixture was concentrated and purified by flash column chromatography (2:98 MeOH:DCM → 8:92 MeOH:DCM). A second flash column purification (40:60 acetone:DCM → 60:40 acetone:DCM) yielded the title compound (10.0 mg, 24 µmol, 24%) as a colorless oil. ^{1}H NMR (500 MHz, MeOD, HH-COSY, HSQC): δ 7.64 – 7.57 (m, 4H, CH_{arom}), 7.45 – 7.38 (m, 4H, CH_{arom}), 7.35 – 7.30 (m, 1H, CH_{arom}), 4.54 (s, 2H, H-6'), 3.60 – 3.57 (m, 2H, H-6/H-5'), 3.55 – 3.50 (m, 3H, H-3, H-6/H-5'), 3.07 (dd, J = 10.9, 8.2 Hz, 1H, H-4), 2.33 (dt, J = 11.7, 7.3 Hz, 1H, H-1'), 2.24 (dt, J = 11.7, 7.3 Hz, 1H, H-1'), 2.06 (ddd, J = 14.1, 4.4, 1.3 Hz, 1H, H-7), 1.78 (ddd, J = 6.4, 3.4, 1.2 Hz, 1H, H-1), 1.68 – 1.55 (m, 6H, H-2, H-7, H-2', H-4'), 1.53 – 1.41 (m, 3H, H-5, H-3'); ^{13}C NMR (126 MHz, MeOD, HSQC): δ 142.1, 141.8, 139.0 ($C_{q\text{-}arom}$), 129.9, 129.4, 128.3, 128.0 (CH_{arom}), 76.3 (C-4), 74.9 (C-3), 73.6 (C-6'), 71.3, 64.5 (C-6, C-5'), 61.6 (C-1'), 45.6 (C-2), 40.9 (C-1), 37.5 (C-5), 30.7, 30.3 (C-2', C-4'), 28.2 (C-7), 25.1 (C-3'); HRMS (ESI) m/z: [M+Na$^+$] calcd for $C_{25}H_{33}NO_4Na$ 434.2307, found 434.2308.

***N*-(Adamantly-1-methoxy)-pentyl-1,2-Dideoxy-1,2-azabicyclo[4.1.0]-carba-α-D-glucose (17).**

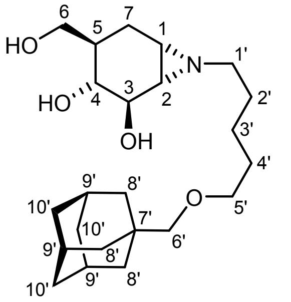

Compound **56** (16 mg, 0.1 mmol), prepared according to literature procedures[1–4], was dissolved in anhydrous DMF (2.0 mL, 0.05 M) followed by the addition of adamantyl-1-methoxypentyl iodide (72 mg, 0.2 mmol, 2.0 eq.), prepared according to literature procedures[3,4], and K_2CO_3 (138 mg, 0.3 mmol, 3.0 eq.). The reaction was stirred for 3 hours at 100 °C under protective atmosphere. Upon full conversion was observed (R_f 0.8 (MeOH:DCM, 2:8, v:v), the reaction mixture was concentrated and purified by flash column chromatography (2:98 MeOH:DCM → 8:92 MeOH:DCM) yielded the title compound (16.3 mg, 41 µmol, 41%) as a colorless oil. ^{1}H NMR (500 MHz, MeOD, HH-COSY, HSQC): δ 3.59 (d, *J* = 4.9 Hz, 2H, H-6), 3.53 (d, *J* = 8.2 Hz, 1H, H-3), 3.39 (t, *J* = 6.4 Hz, 2H, H-5'), 3.09 – 3.07 (m, 1H, H-4), 2.97 (s, 2H, H-6'), 2.36 (dt, *J* = 11.7, 7.2 Hz, 1H, H-1'), 2.23 (dt, *J* = 11.7, 7.4 Hz, 1H, H-1'), 2.07 (ddd, *J* = 14.2, 4.5, 1.3 Hz, 1H, H-7), 1.95 (p, *J* = 3.1 Hz, 3H, H-9', H-9', H-9'), 1.82 – 1.73 (m, 4H, H-10', H-10', H-10', H-1/H-2), 1.71 – 1.66 (m, 4H, H-10', H-10', H-10'), 1.63 – 1.54 (m, 12H, H-1/H-2, H-7, H-2', H-4', H-8', H-8', H-8'), 1.53 – 1.47 (m, 1H, H-5), 1.44 – 1.38 (m, 2H, H-3'); ^{13}C NMR (126 MHz, MeOD, HSQC): δ 83.1 (C-6'), 76.3 (C-4), 74.9 (C-3), 72.5 (C-5'), 64.5 (C-6), 61.7 (C-1'), 45.6, 40.9 (C-1, C-2), 40.9 (C-2'/C-4'/C-8'), 38.4 (C-10'), 37.5 (C-5), 35.2 (C-7'), 30.7 (C-2'/C-4'/C-8'), 30.4 (C-2'/C-4'/C-8'), 30.2 (C-9'), 28.2 (C-7), 25.2 (C-3'); HRMS (ESI) m/z: [M+Na$^+$] calcd for $C_{23}H_{39}NO_4Na$ 416.2777, found 416.2778.

***N*-(Naphtalenyl-1-methoxy)-pentyl-1,2-Dideoxy-1,2-azabicyclo[4.1.0]-carba-α-D-glucose (18).**

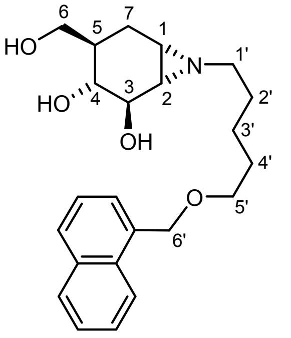

Compound **56** (16 mg, 0.1 mmol), prepared according to literature procedures[1–4], was dissolved in anhydrous DMF (2.0 mL, 0.05 M) followed by the addition of naphtalenyl-1-methoxypentyl bromide (61 mg, 0.2 mmol, 2.0 eq.), prepared according to literature procedures[3,4], and K_2CO_3 (138 mg, 0.3 mmol, 3.0 eq.). The reaction was stirred for 3 hours at 100 °C under protective atmosphere. Upon full conversion was observed (R_f 0.7 (MeOH:DCM, 2:8, v:v), the reaction mixture was concentrated and purified by flash column chromatography (2:98 MeOH:DCM → 8:92 MeOH:DCM). A second flash column purification (40:60 acetone:DCM → 70:30 acetone:DCM) yielded the title compound (14.5 mg, 38 µmol, 38%) as a colorless oil. ^{1}H NMR (500 MHz, MeOD, HH-COSY, HSQC): δ 8.14 – 8.11 (m, 1H, CH_{arom}), 7.90 – 7.81 (m, 2H, CH_{arom}), 7.55 – 7.41 (m, 4H, CH_{arom}), 4.94 (d, *J* = 1.9 Hz, 2H, H-6'), 3.59 – 3.55 (m, 4H, H-6, H-5'), 3.51 (d, *J* = 8.2 Hz, 1H, H-3), 3.06 (dd, *J* = 10.9, 8.2 Hz, 1H, H-4), 2.28 (dt, *J* = 11.7, 7.2 Hz, 1H, H-1'), 2.14 (dt, *J* = 11.7, 7.4 Hz, 1H, H-1'), 2.01 (ddd, *J* = 14.0, 4.5, 1.2 Hz, 1H, H-7), 1.73 – 1.68 (m, 1H, H-2, H-2'/H-4'), 1.67 – 1.57 (m, 2H), 1.57 – 1.36 (m, 7H, H-1, H-5, H-7, H-3', H-2'/H-4'); ^{13}C NMR (126 MHz, MeOD, HSQC): δ 135.3, 135.2, 133.2 ($C_{q\text{-}arom}$), 129.7, 129.5, 127.7, 127.1, 126.8, 126.2, 125.2 (CH_{arom}), 76.3 (C-4), 74.9 (C-3), 72.4 (C-6'), 71.1, 64.5 (C-6, C-5'), 61.5 (C-1'), 45.6 (C-1), 40.9 (C-2), 37.5 (C-5), 30.7, 30.2 (C-2', C-4'), 28.2 (C-7), 25.0 (C-3'); HRMS (ESI) m/z: [M+Na$^+$] calcd for $C_{23}H_{31}NO_4Na$ 408.2151, found 408.2152.

NMR Data; spectra of new and selected compounds

^{1}H NMR, 400MHz, MeOD of **30**

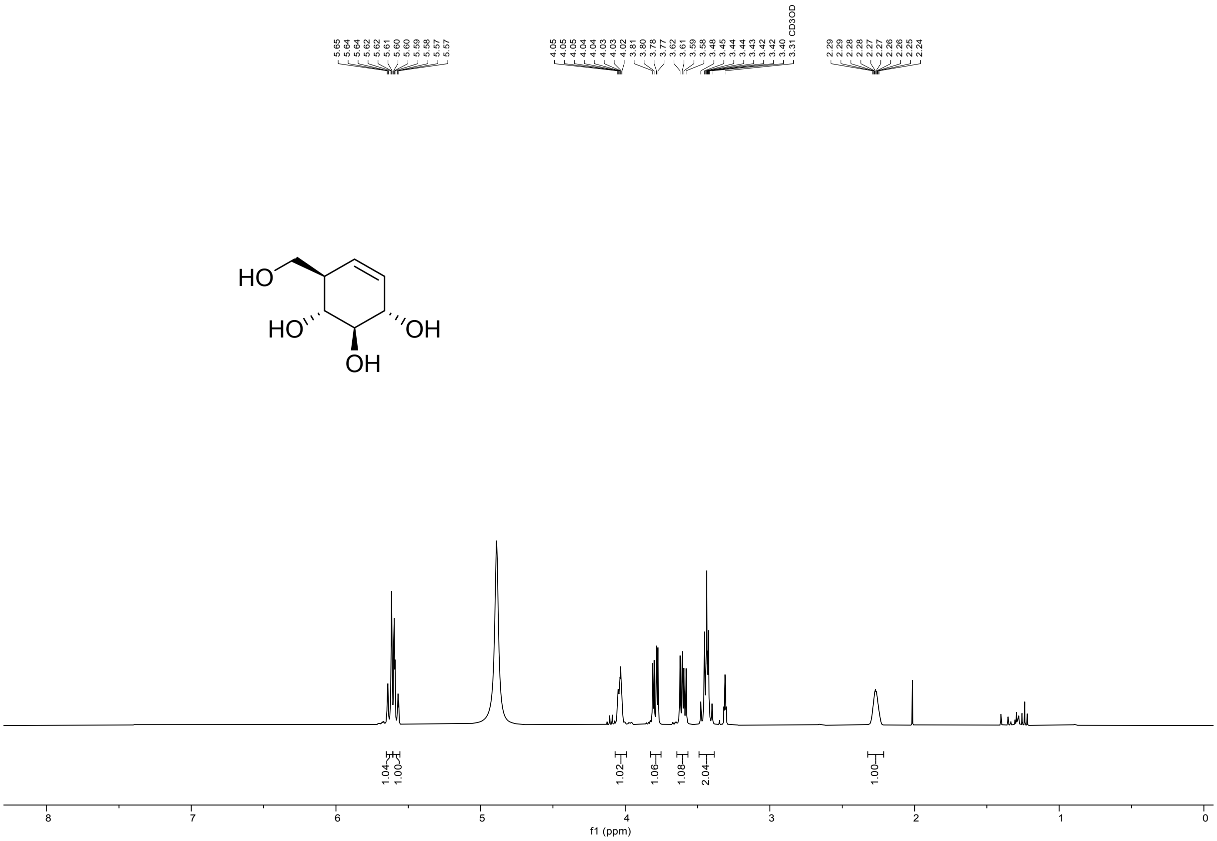

^{13}C NMR, 101MHz, MeOD of **30**

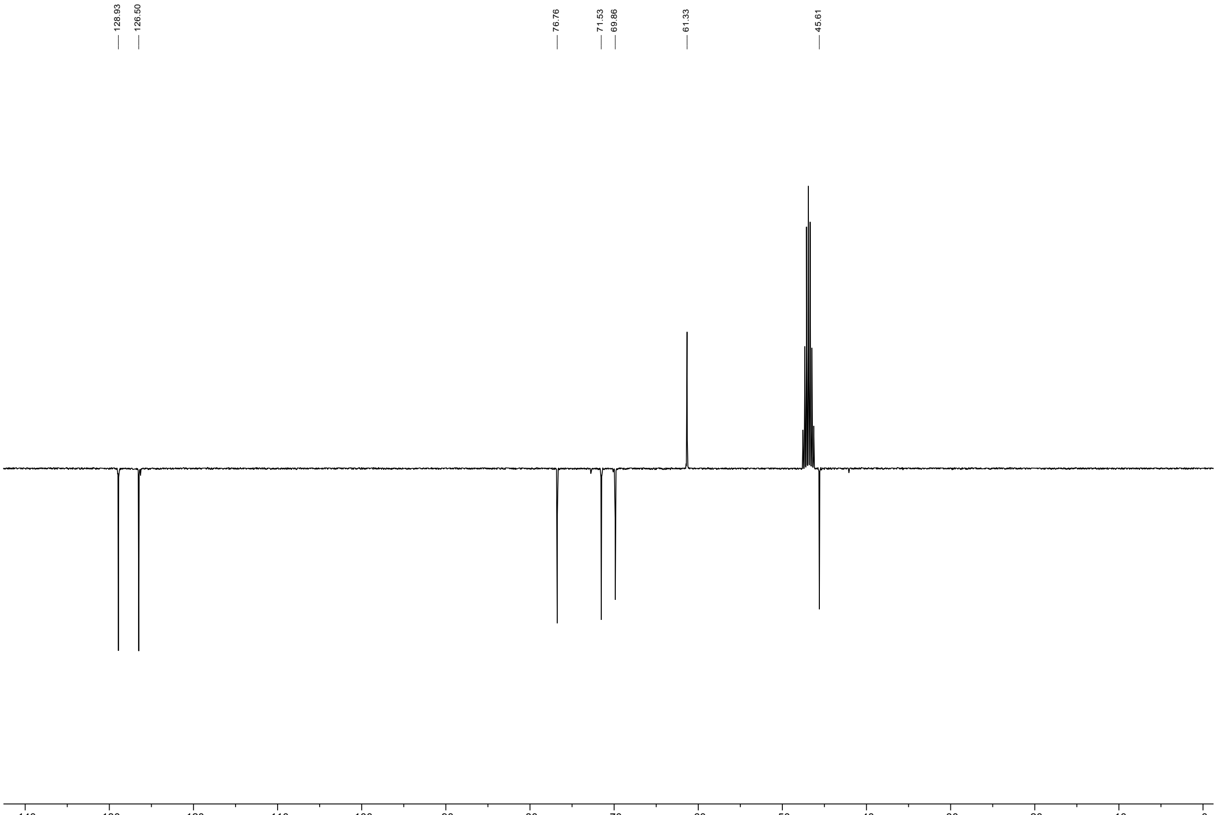

HH-COSY NMR, MeOD of **30**

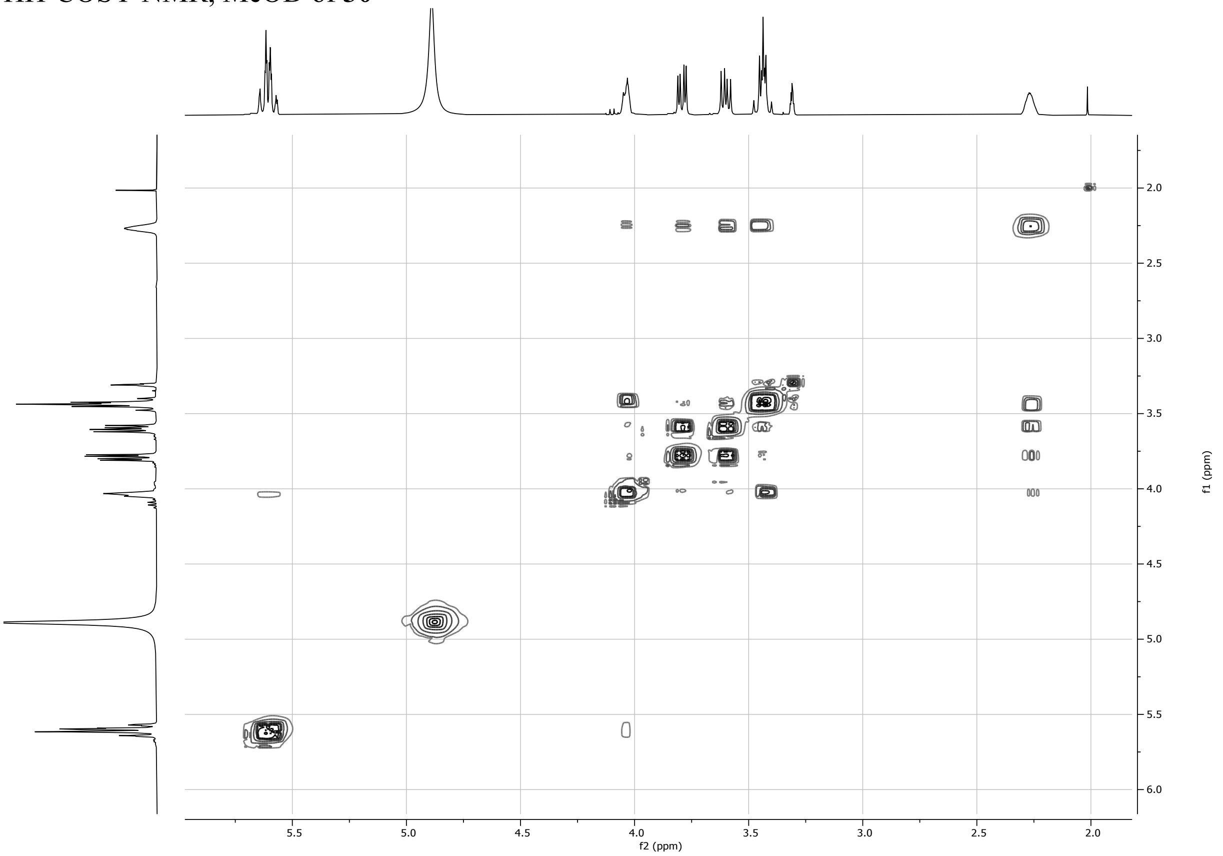

HSQC NMR, MeOD of **30**

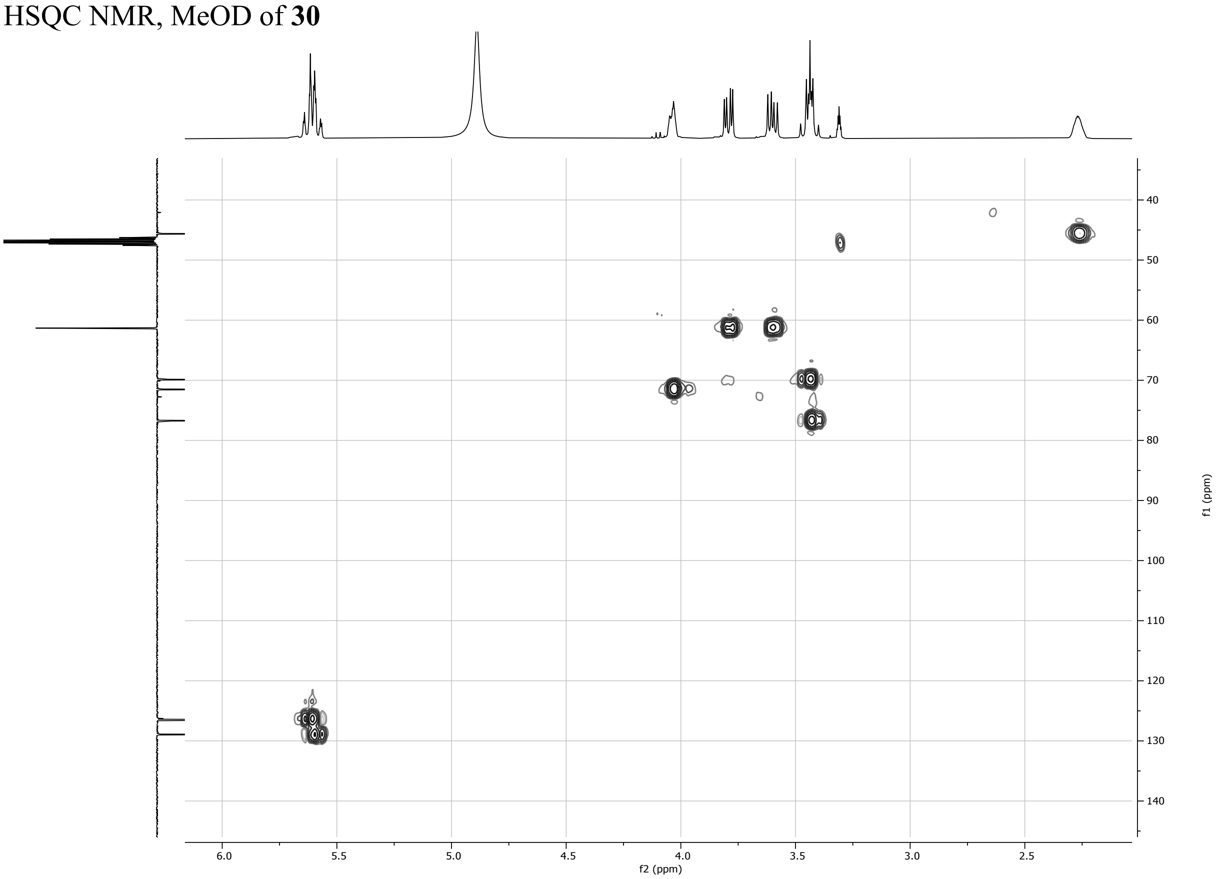

^{1}H NMR, 400MHz, $CDCl_3$ of **31**

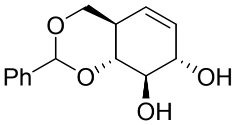

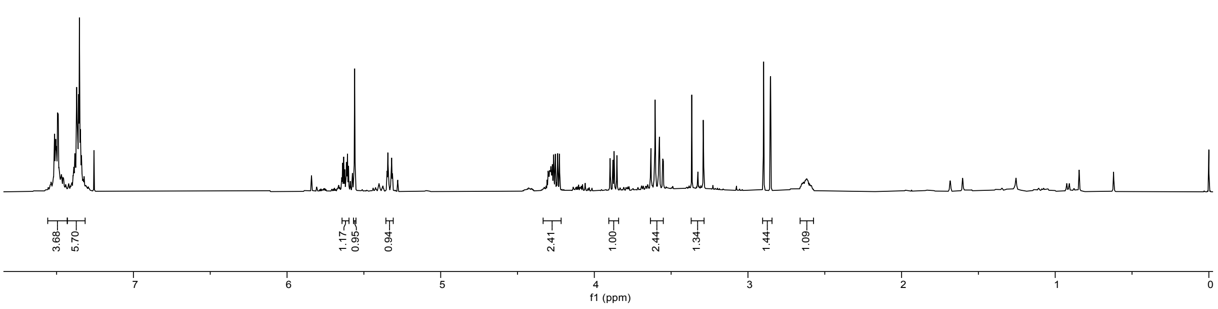

^{13}C NMR, 101MHz, $CDCl_3$ of **31**

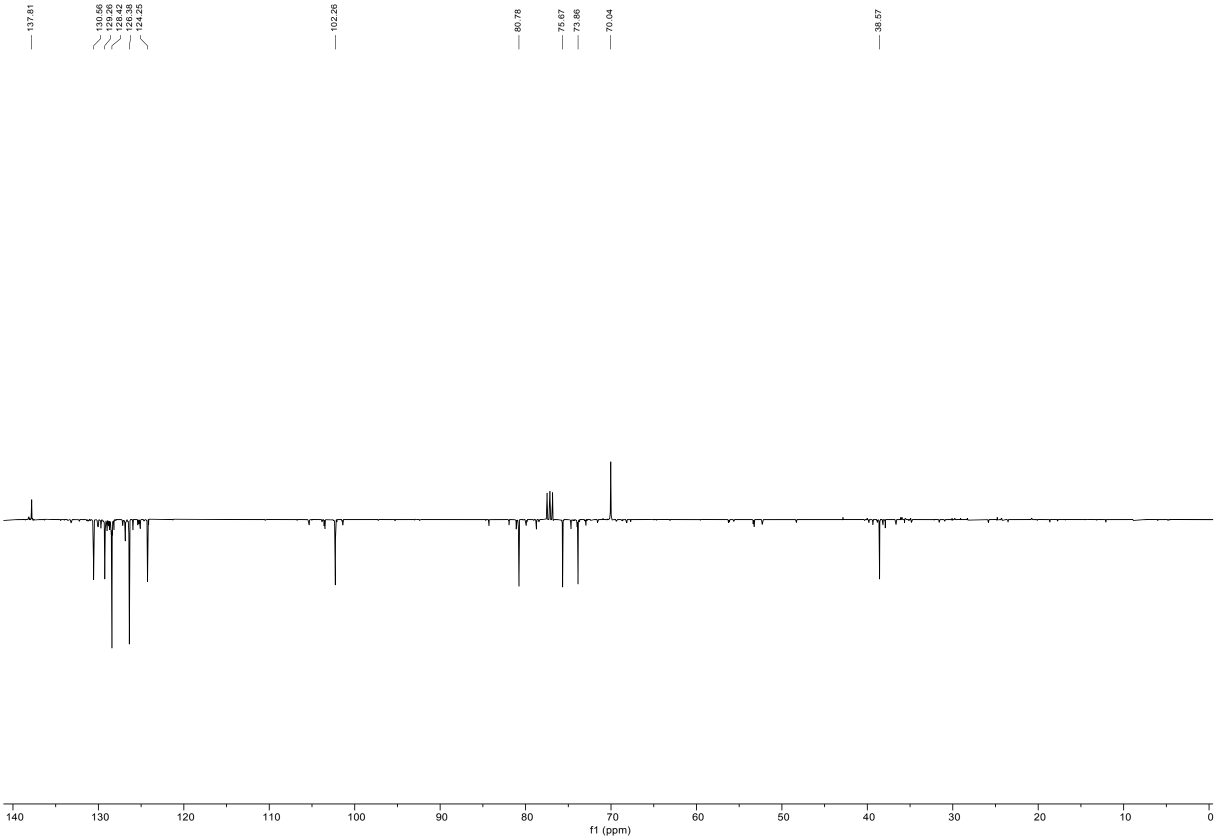

HH-COSY NMR, $CDCl_3$ of **31**

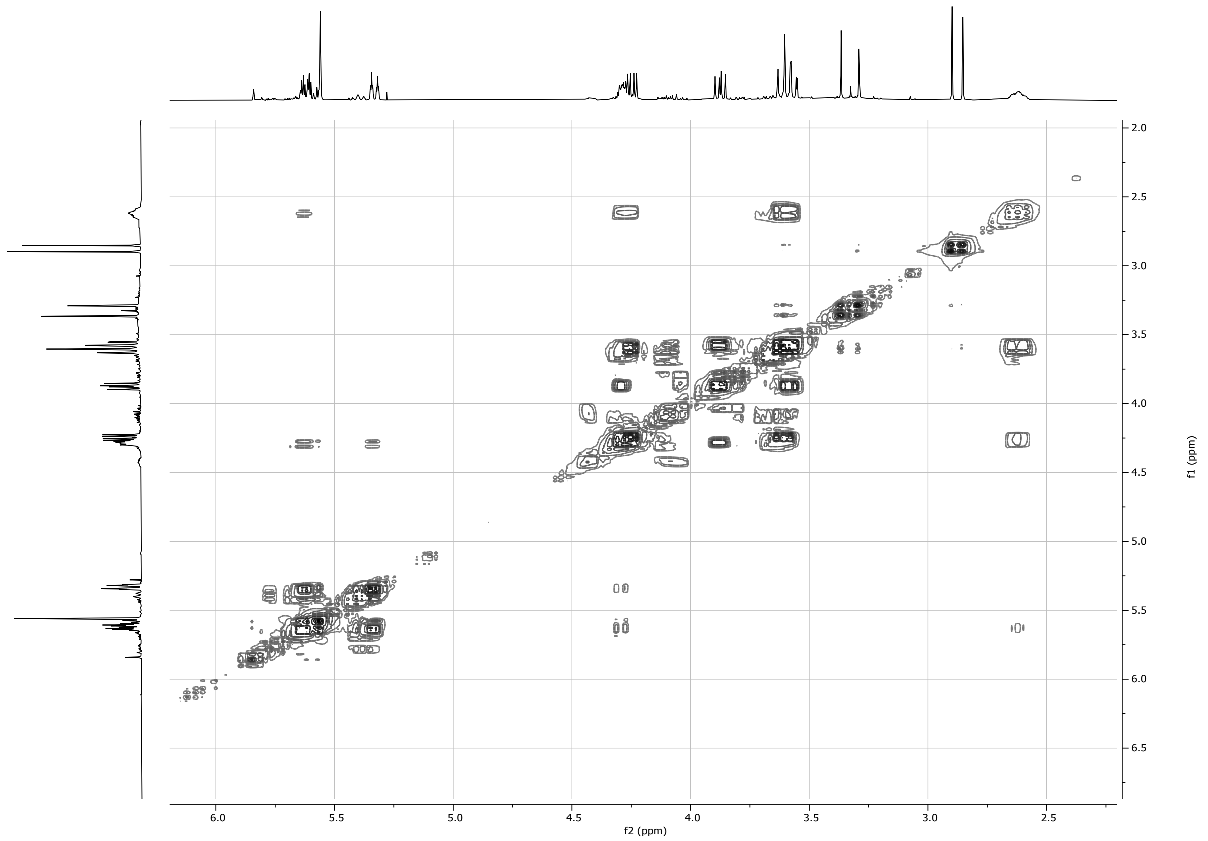

HSQC NMR, $CDCl_3$ of **31**

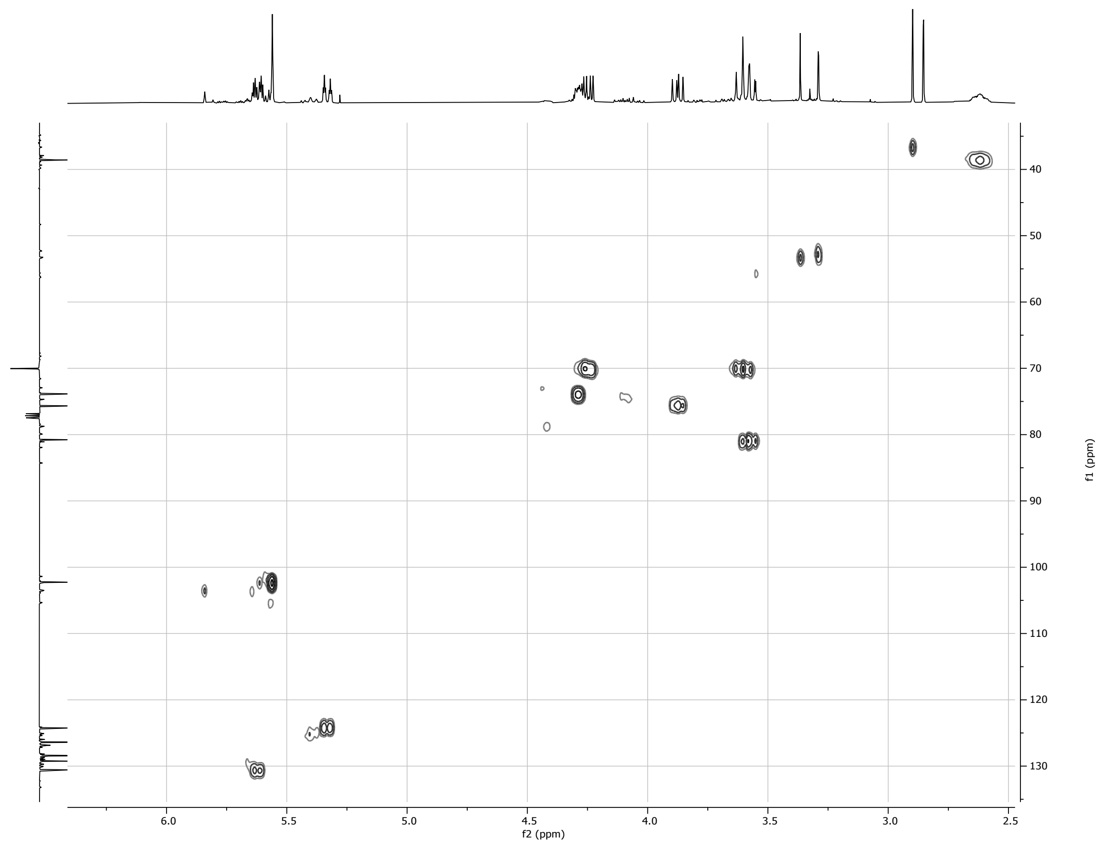

^{1}H NMR, 500MHz, $CDCl_3$ of **32**

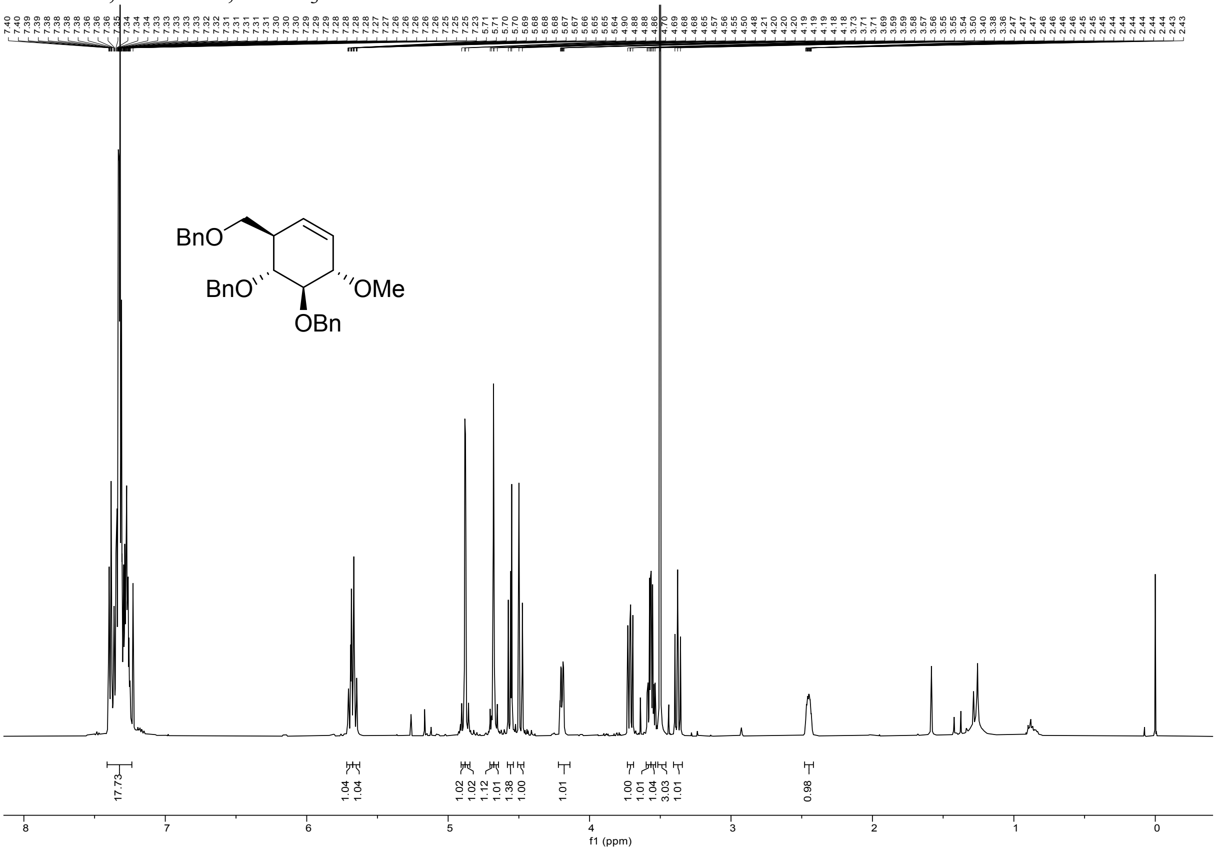

^{13}C NMR, 126MHz, $CDCl_3$ of **32**

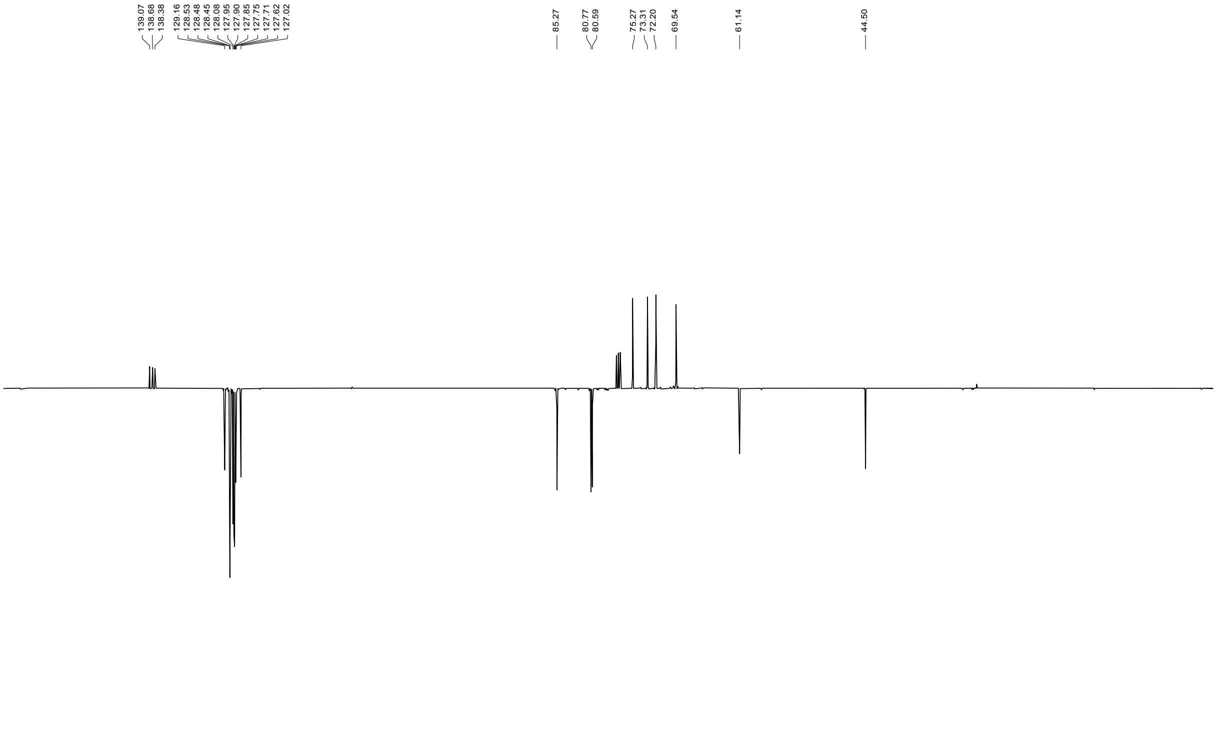

HH-COSY NMR, $CDCl_3$ of **32**

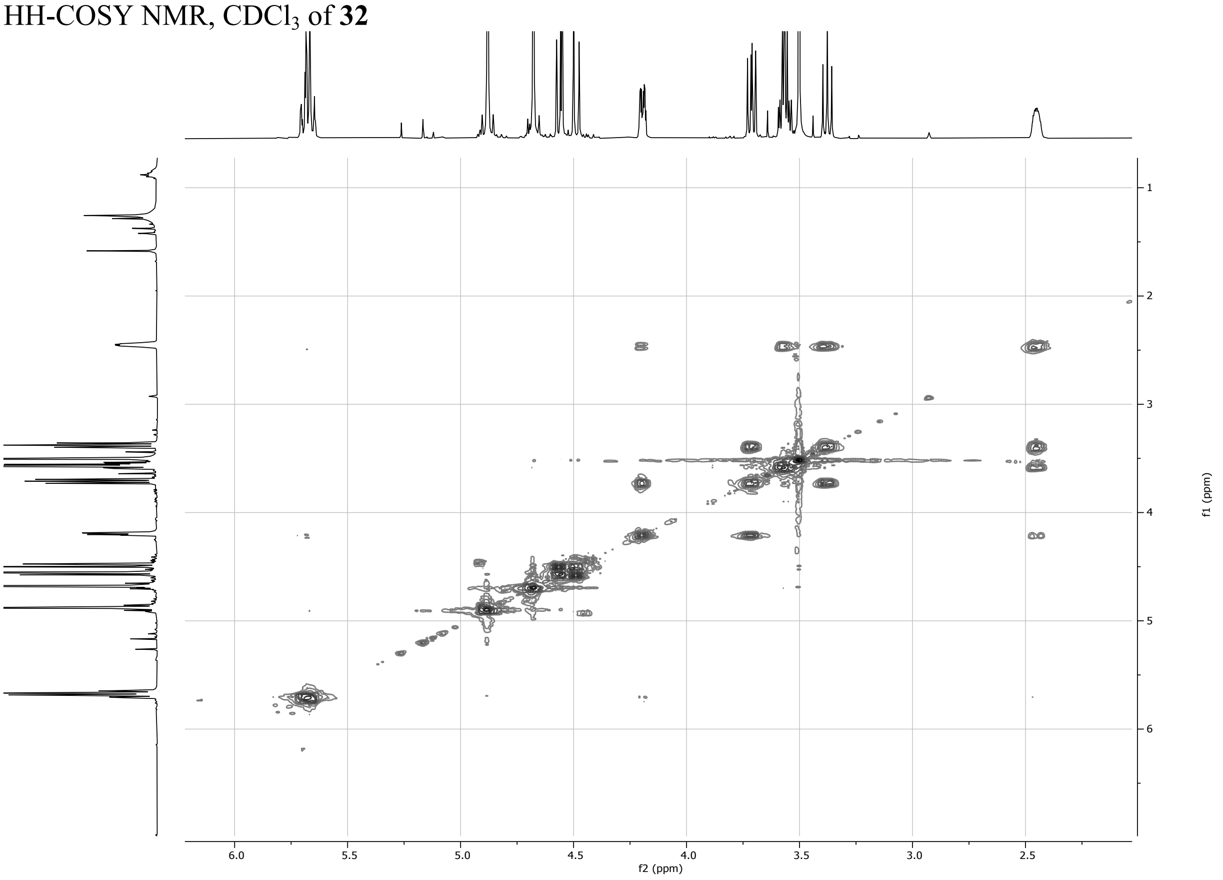

HSQC NMR, $CDCl_3$ of **32**

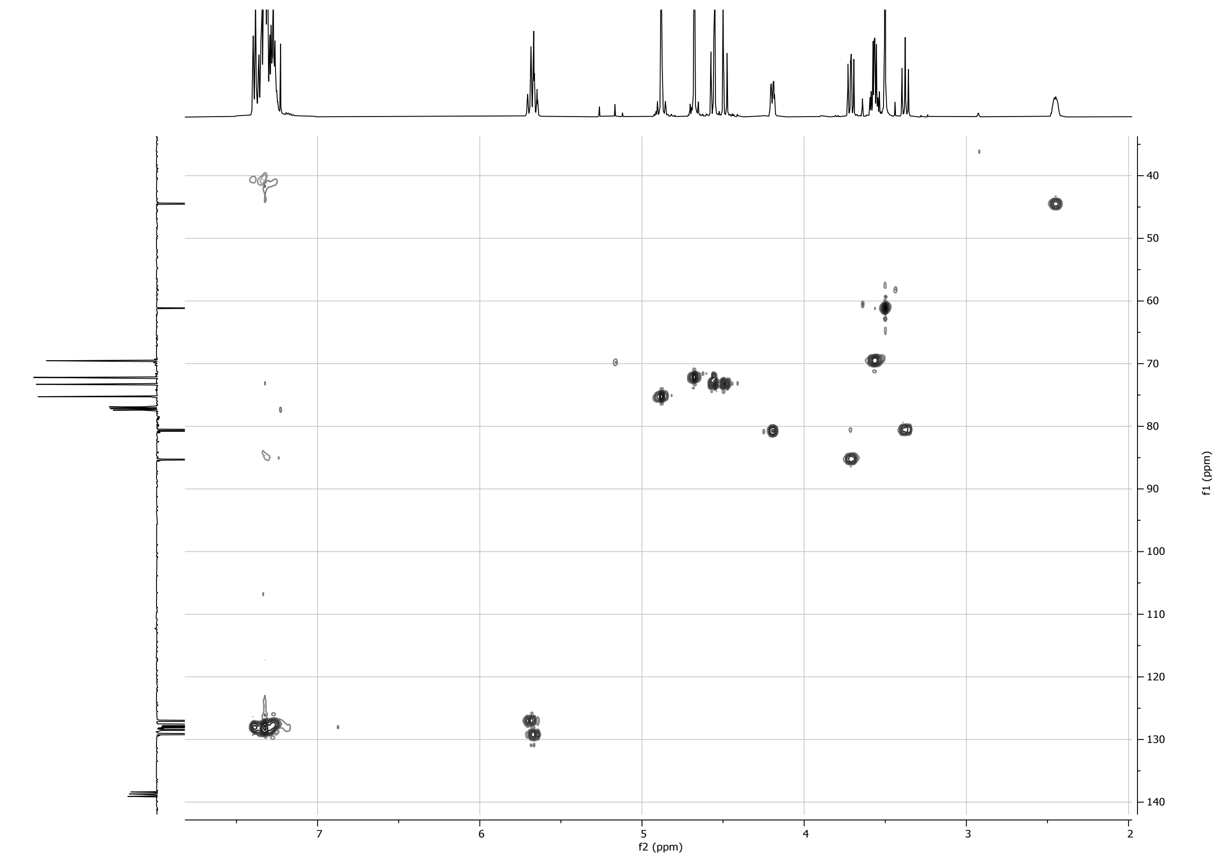

^{1}H NMR, 500MHz, $CDCl_3$ of **38**

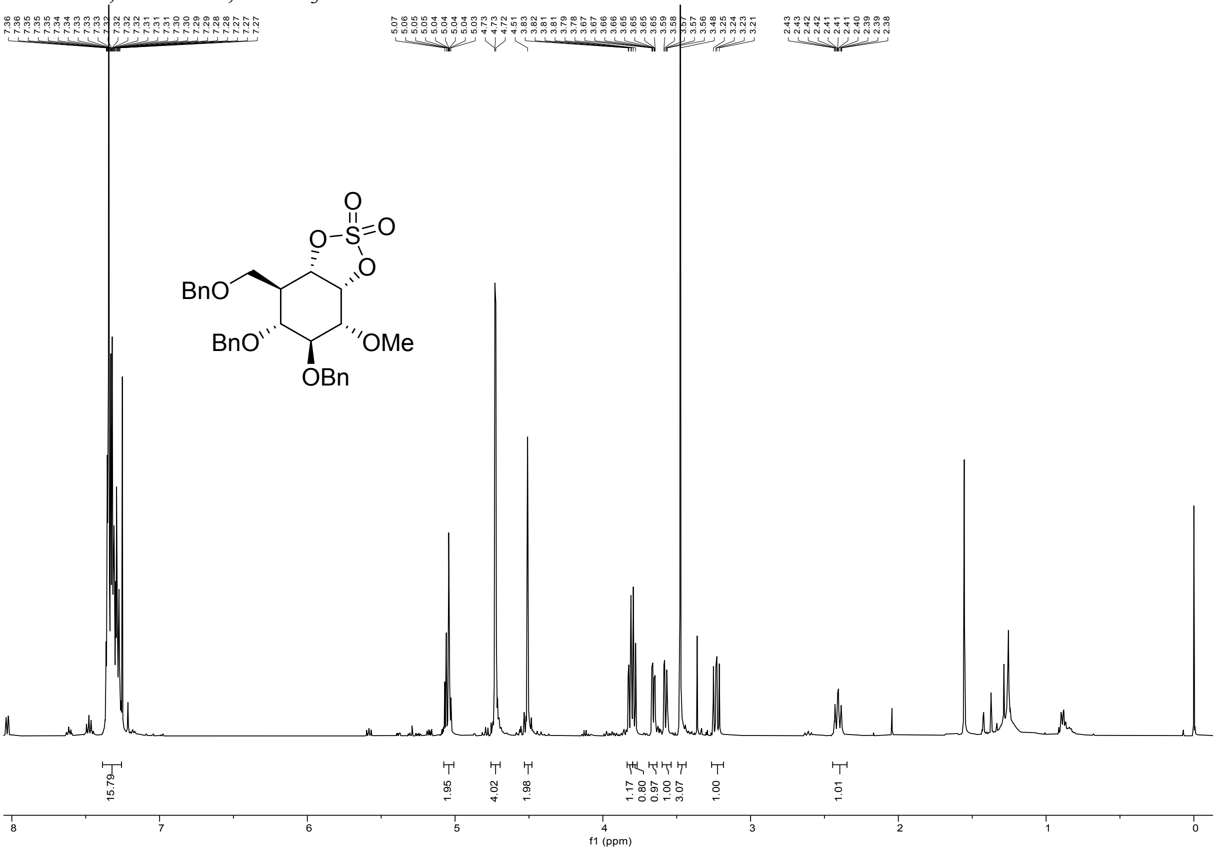

^{13}C NMR, 126MHz, $CDCl_3$ of **38**

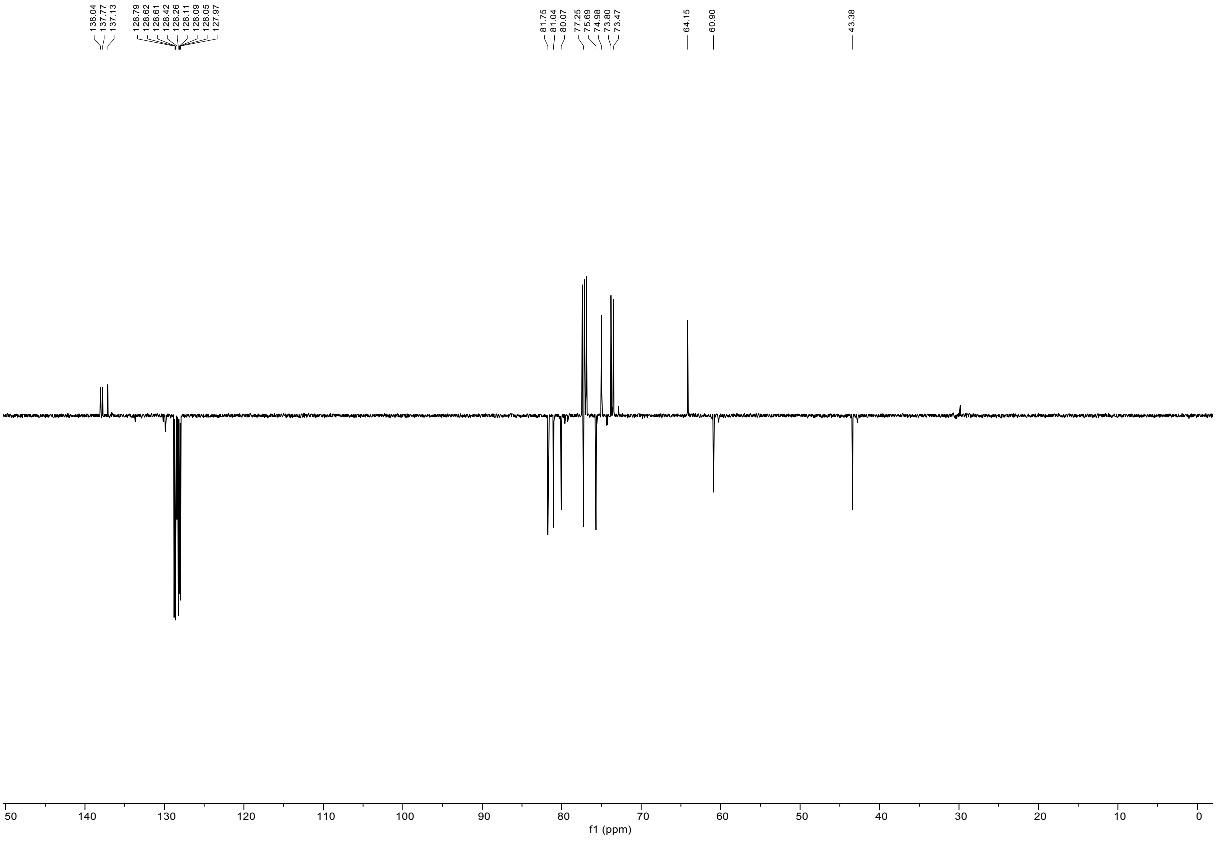

HH-COSY NMR, $CDCl_3$ of **38**

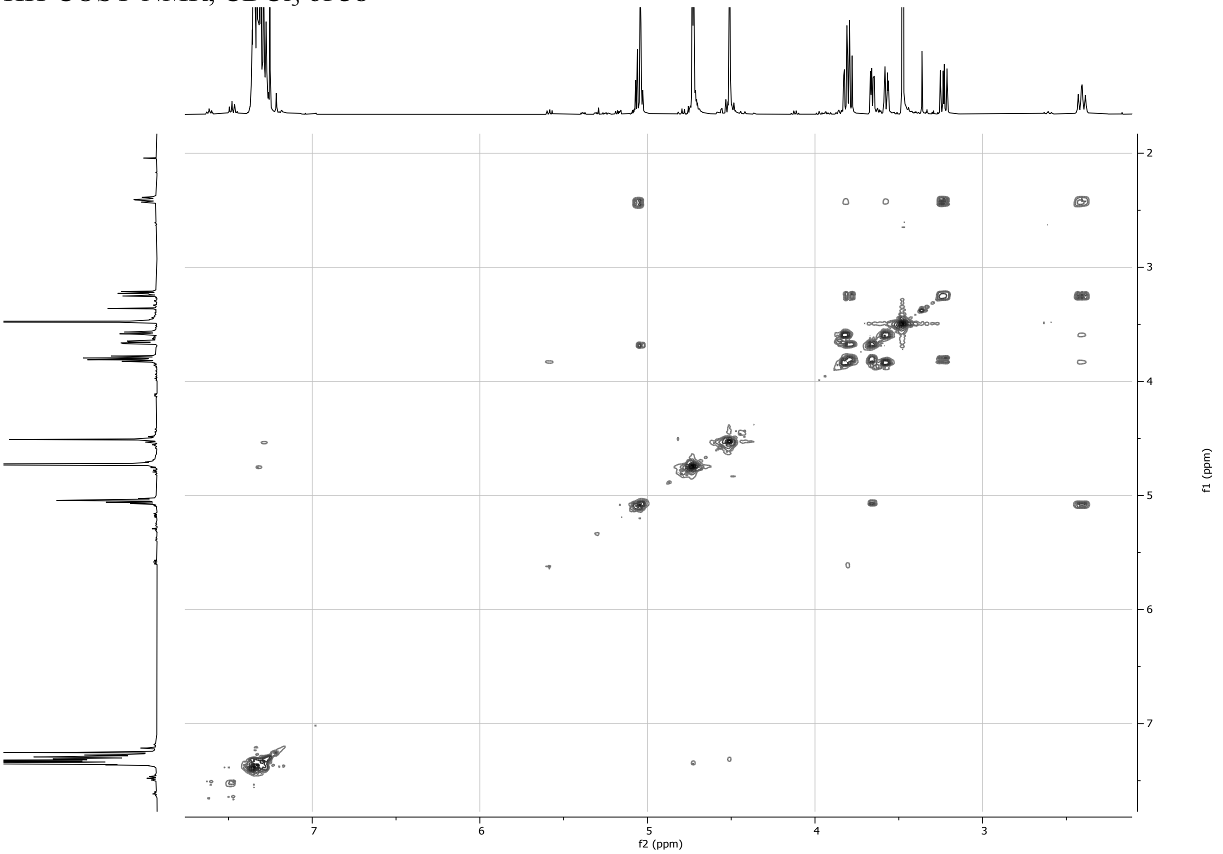

HSQC NMR, $CDCl_3$ of **38**

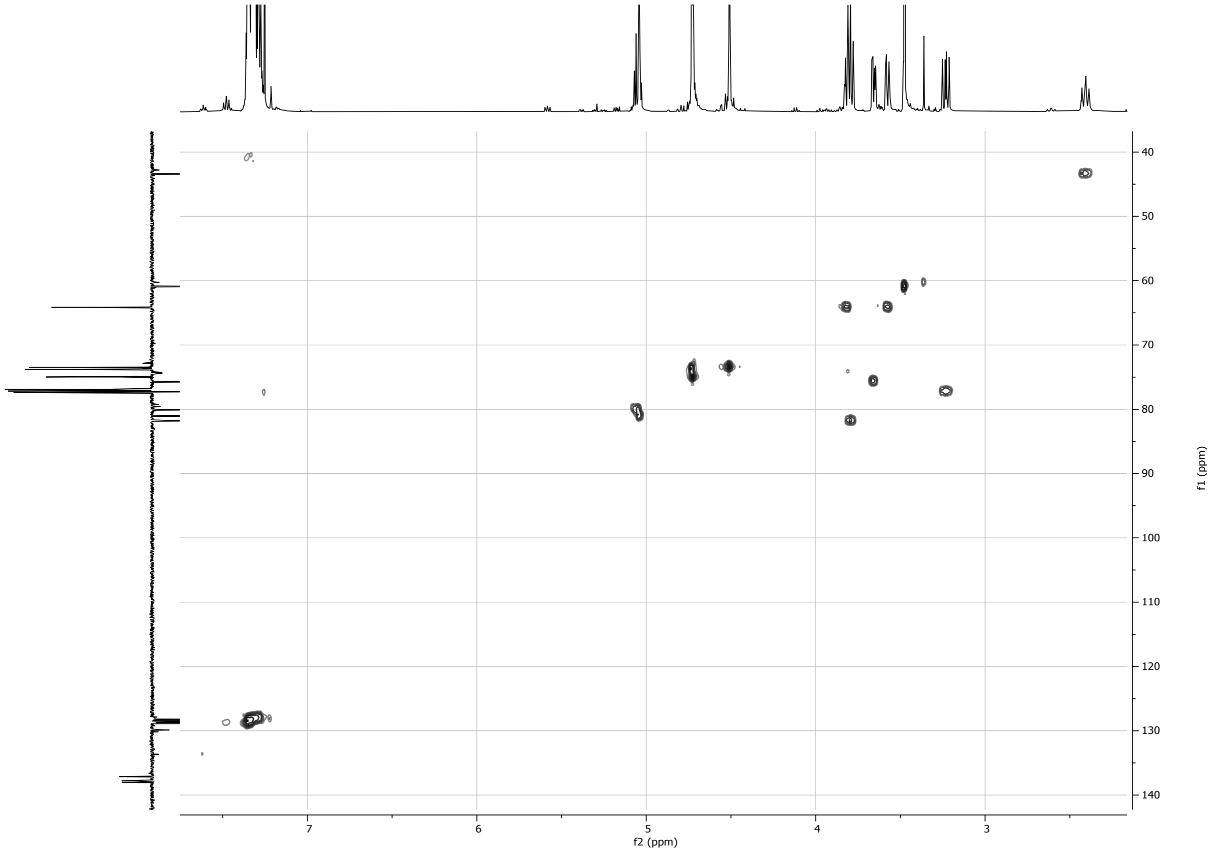

^{1}H NMR, 500MHz, MeOD of **5**

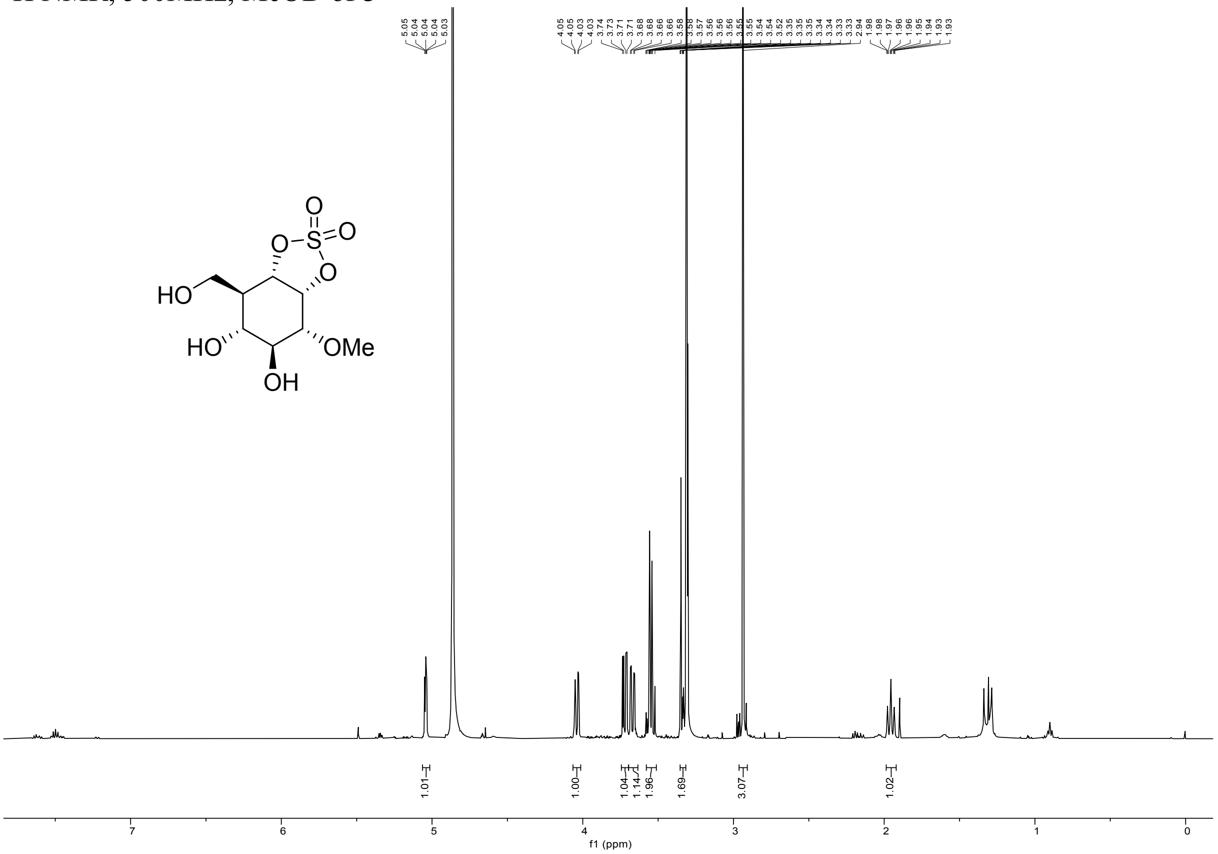

^{13}C NMR, 126MHz, MeOD of **5**

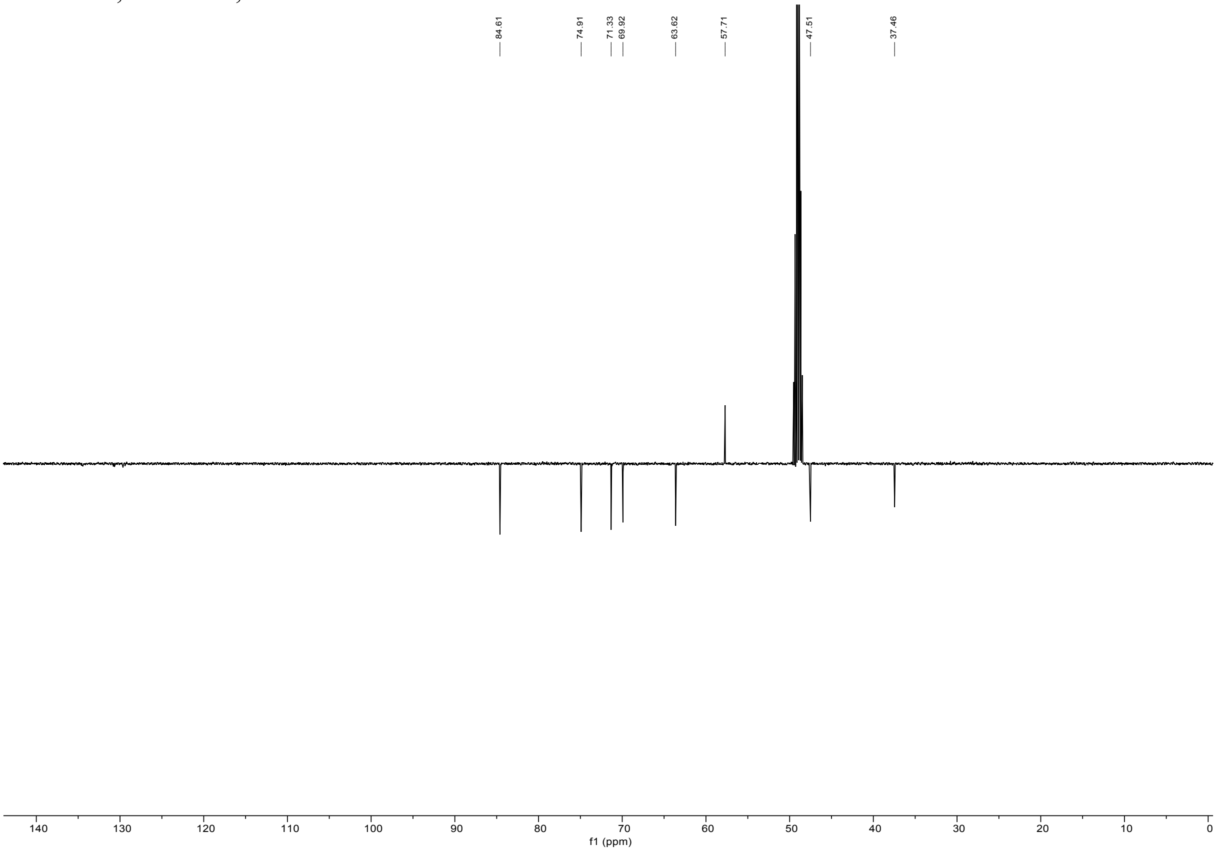

HH-COSY NMR, MeOD of **5**

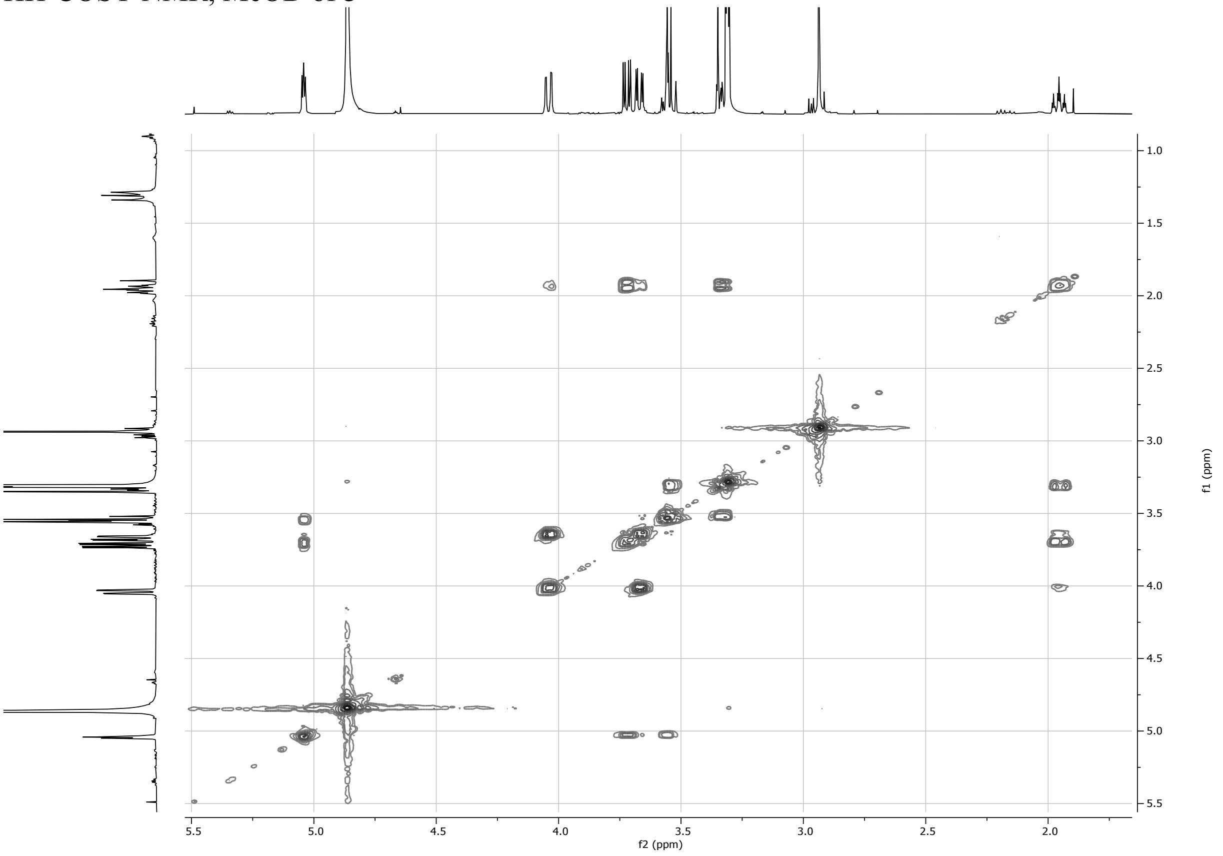

HSQC NMR, MeOD of **5**

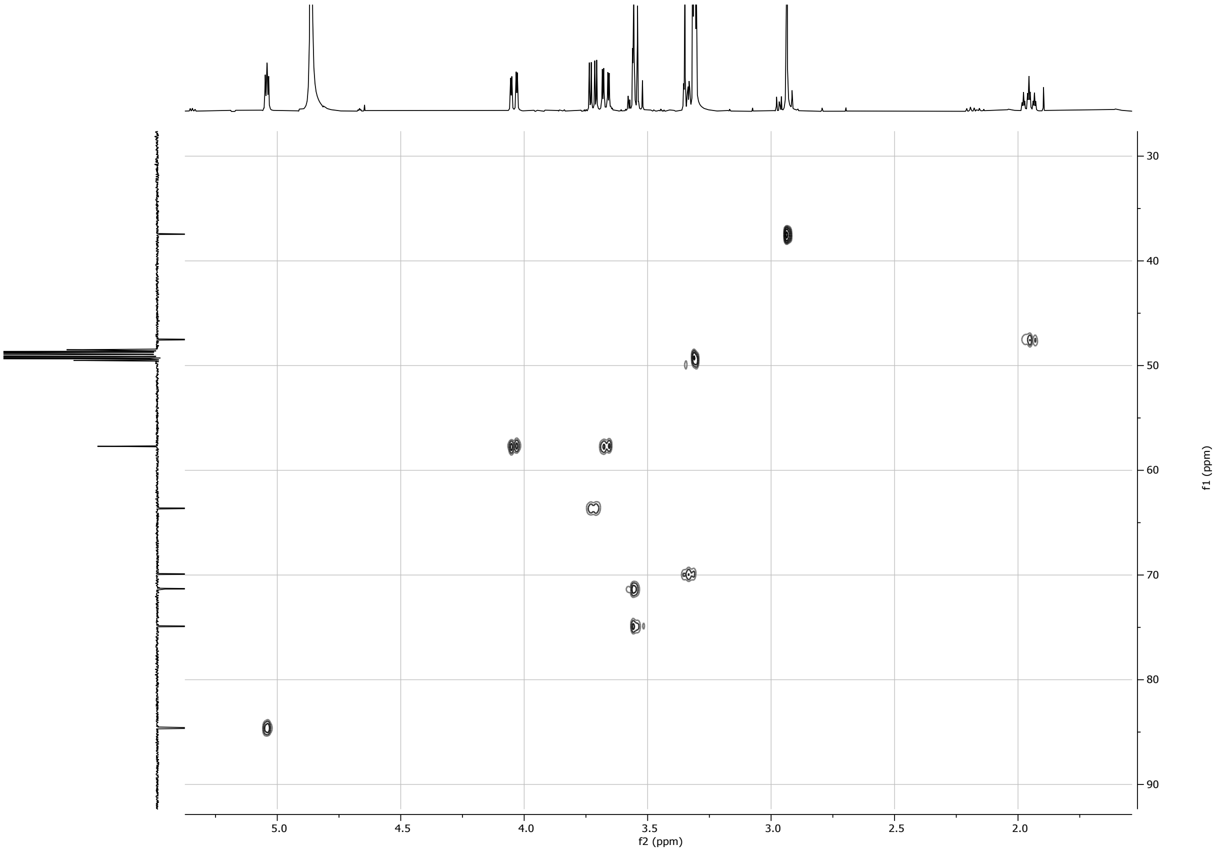

^{1}H NMR, 500MHz, $CDCl_3$ of **33**

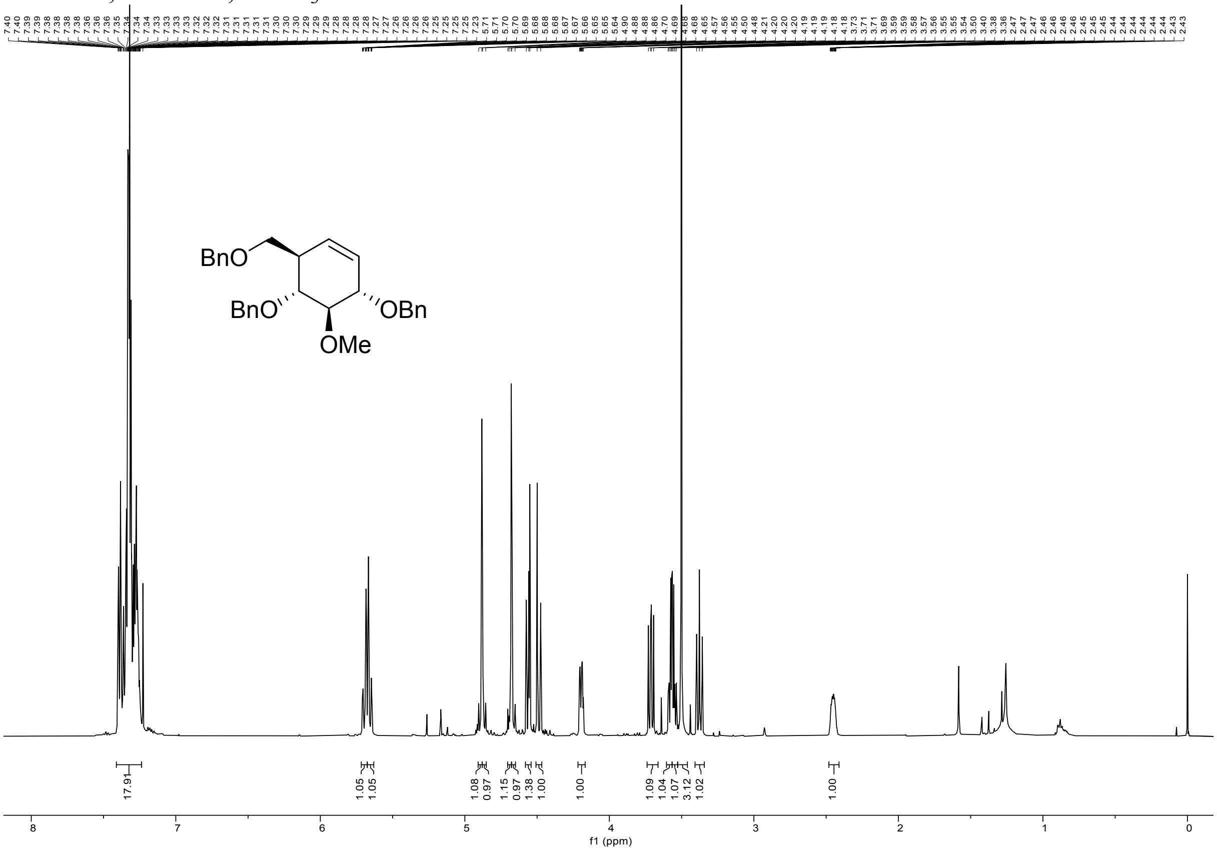

^{13}C NMR, 126MHz, $CDCl_3$ of **33**

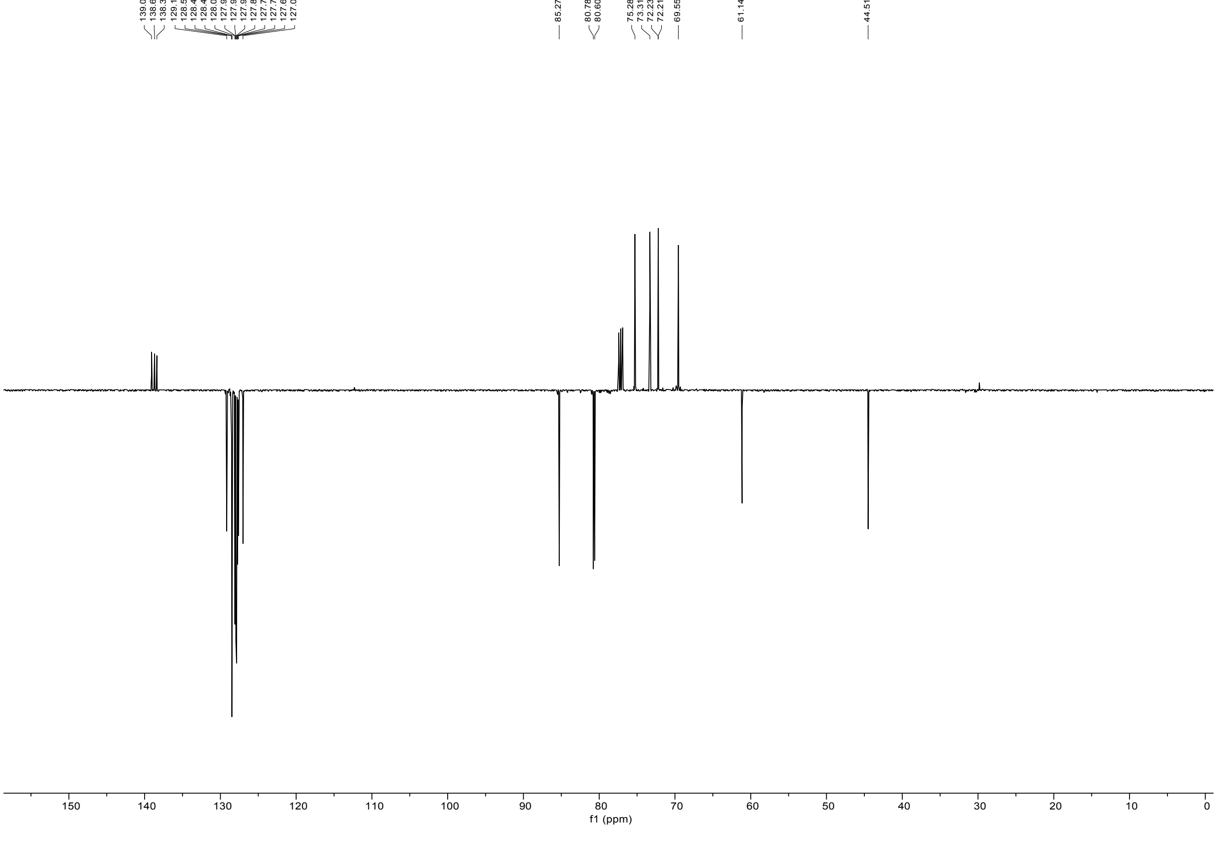

HH-COSY NMR, $CDCl_3$ of **33**

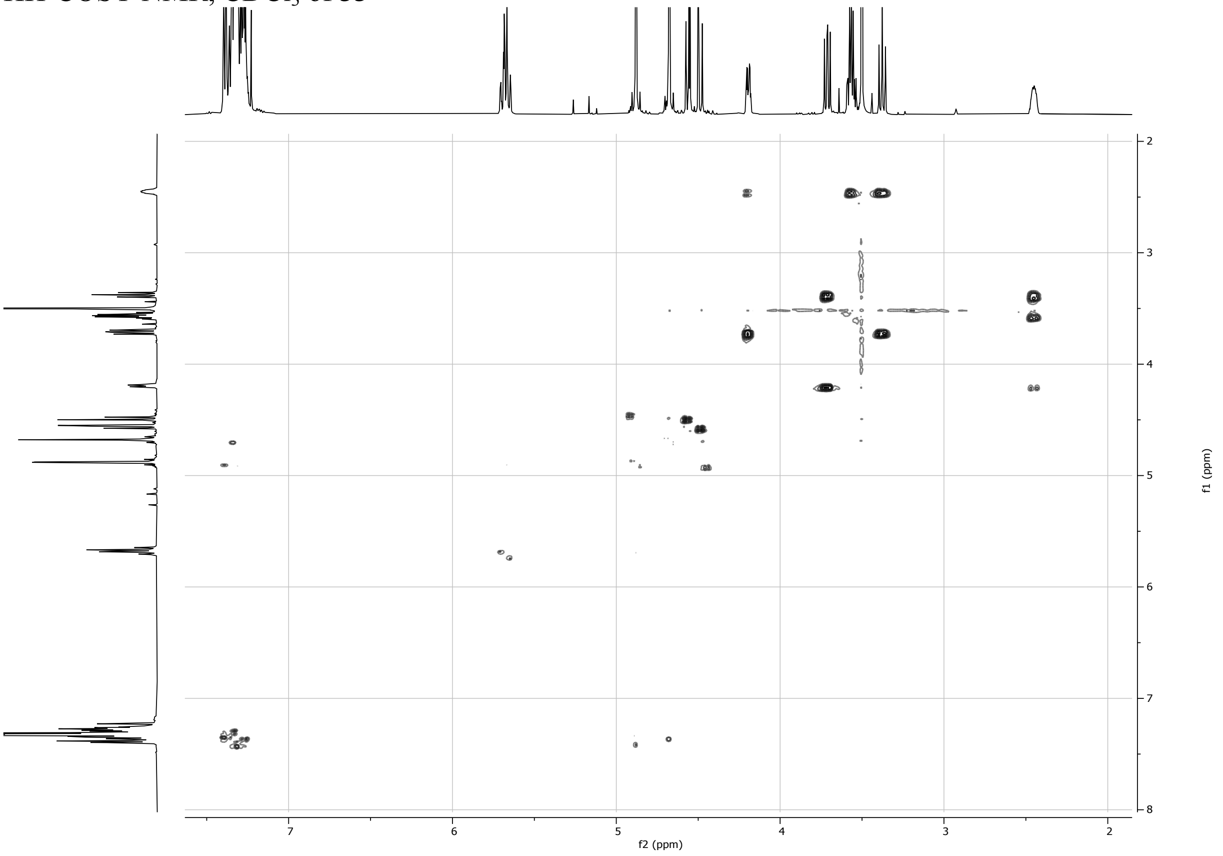

HSQC NMR, $CDCl_3$ of **33**

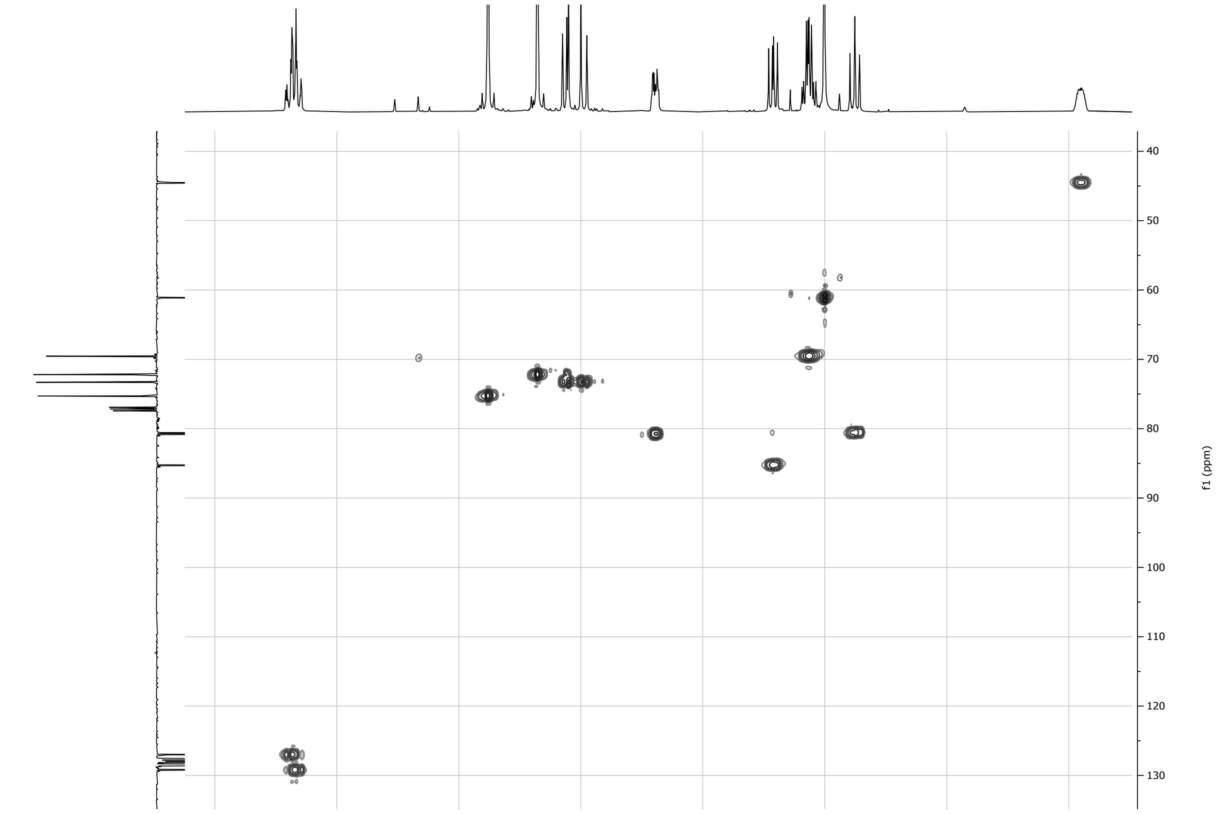

^{1}H NMR, 500MHz, $CDCl_3$ of **39**

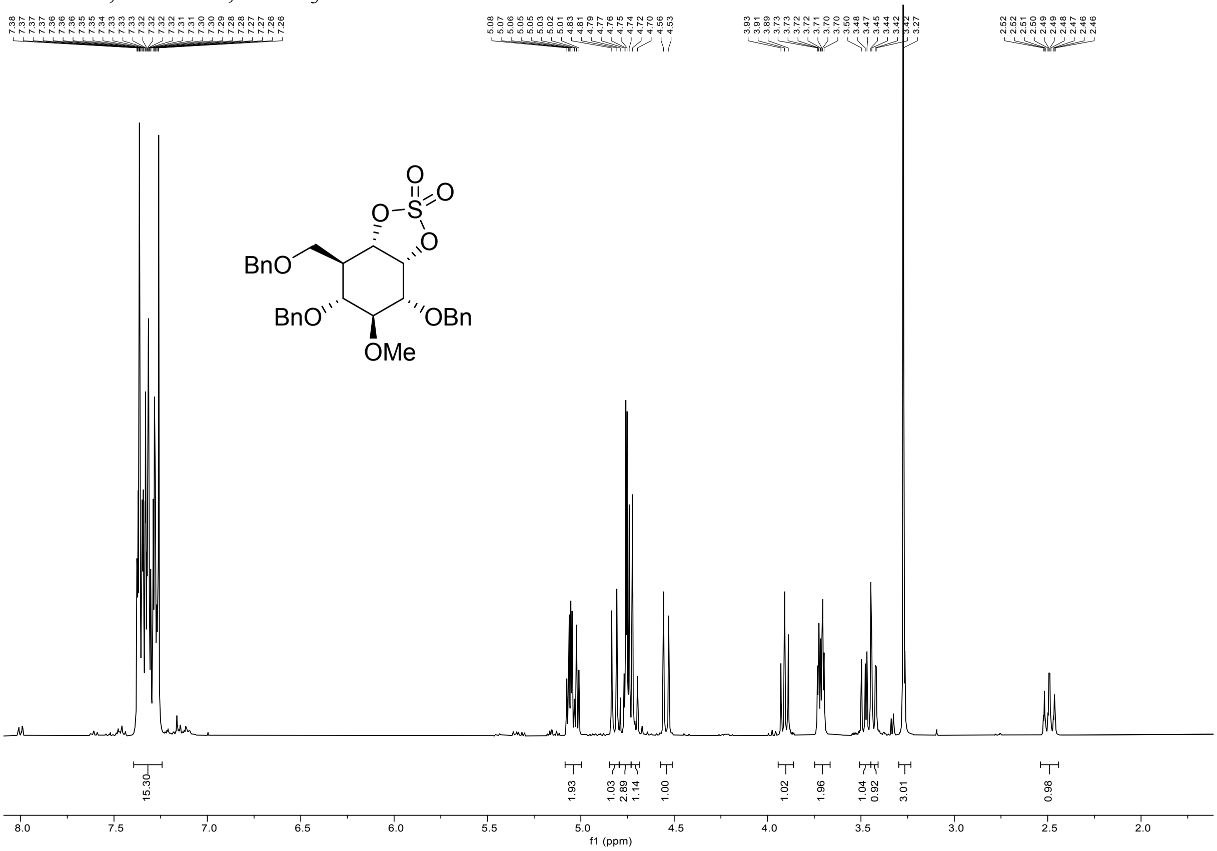

^{13}C NMR, 126MHz, $CDCl_3$ of **39**

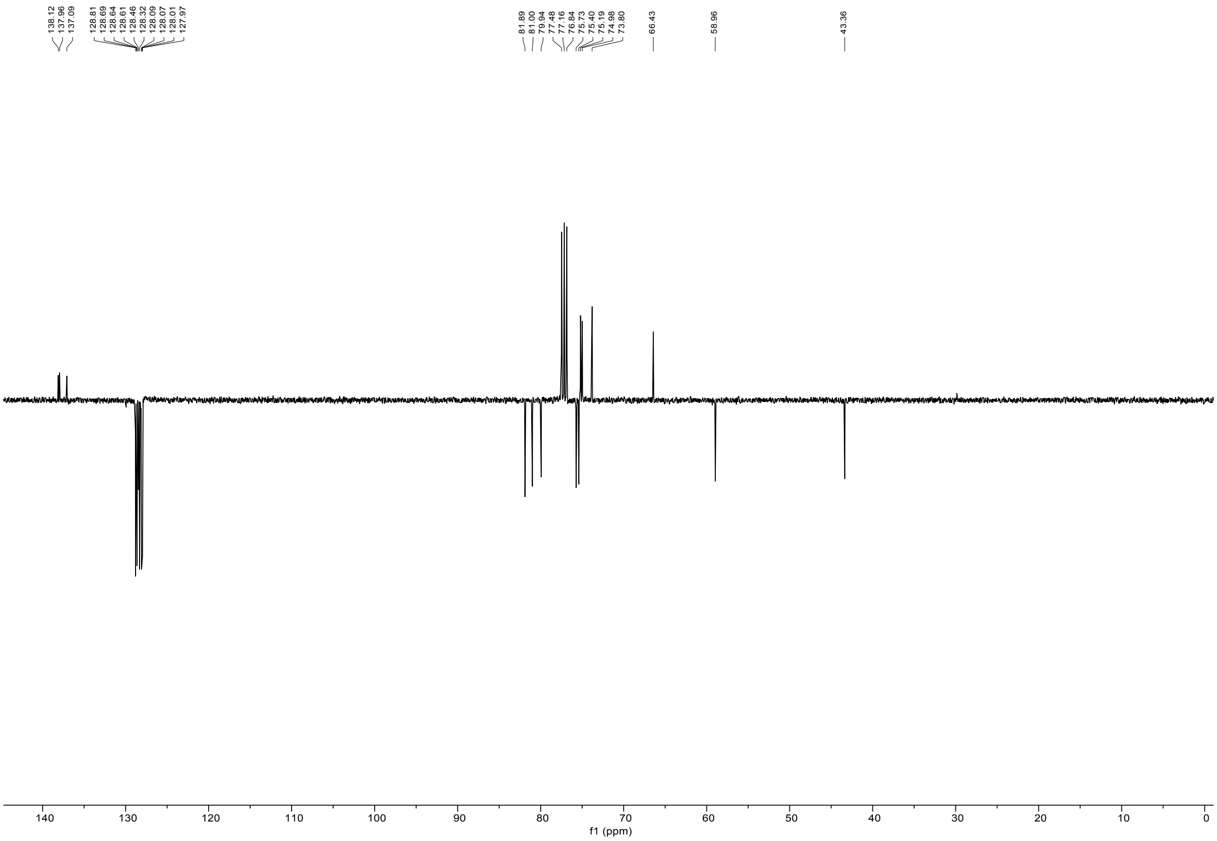

HH-COSY NMR, $CDCl_3$ of **39**

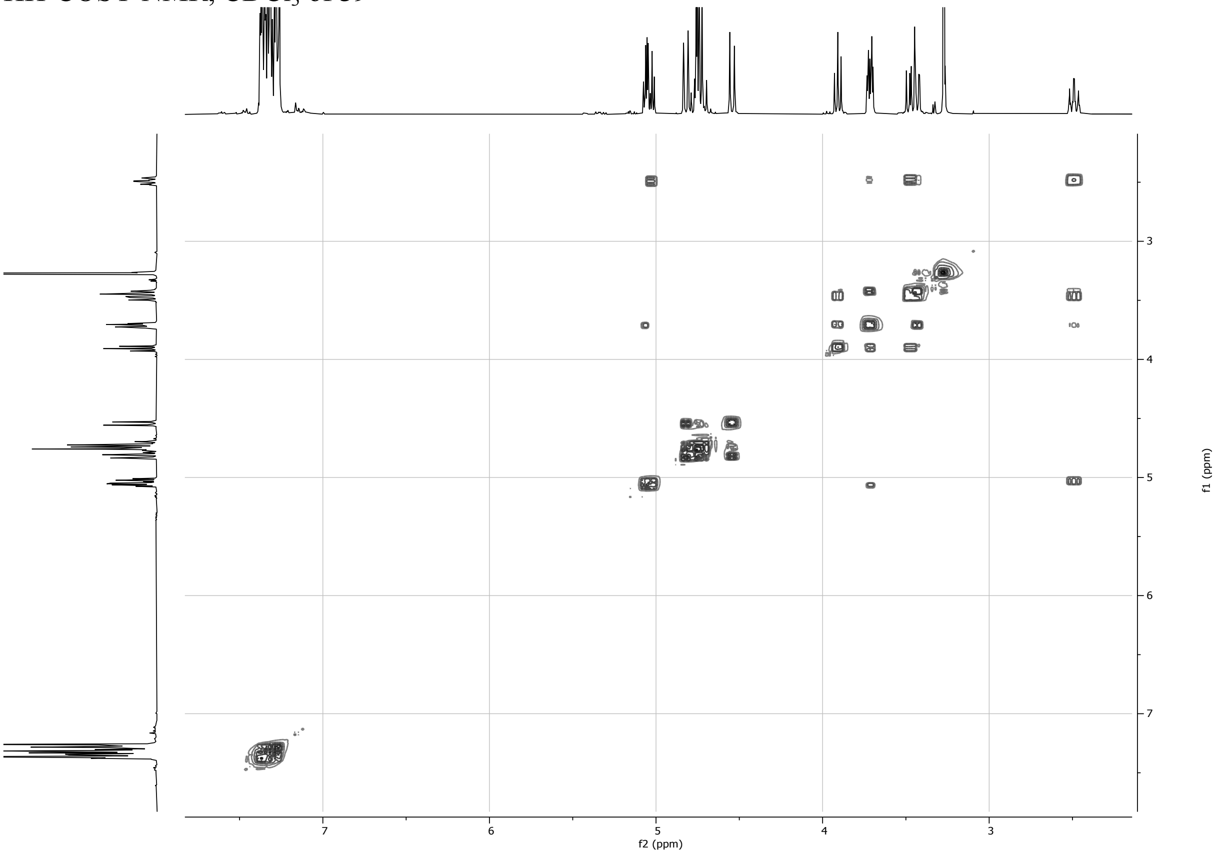

HSQC NMR, $CDCl_3$ of **39**

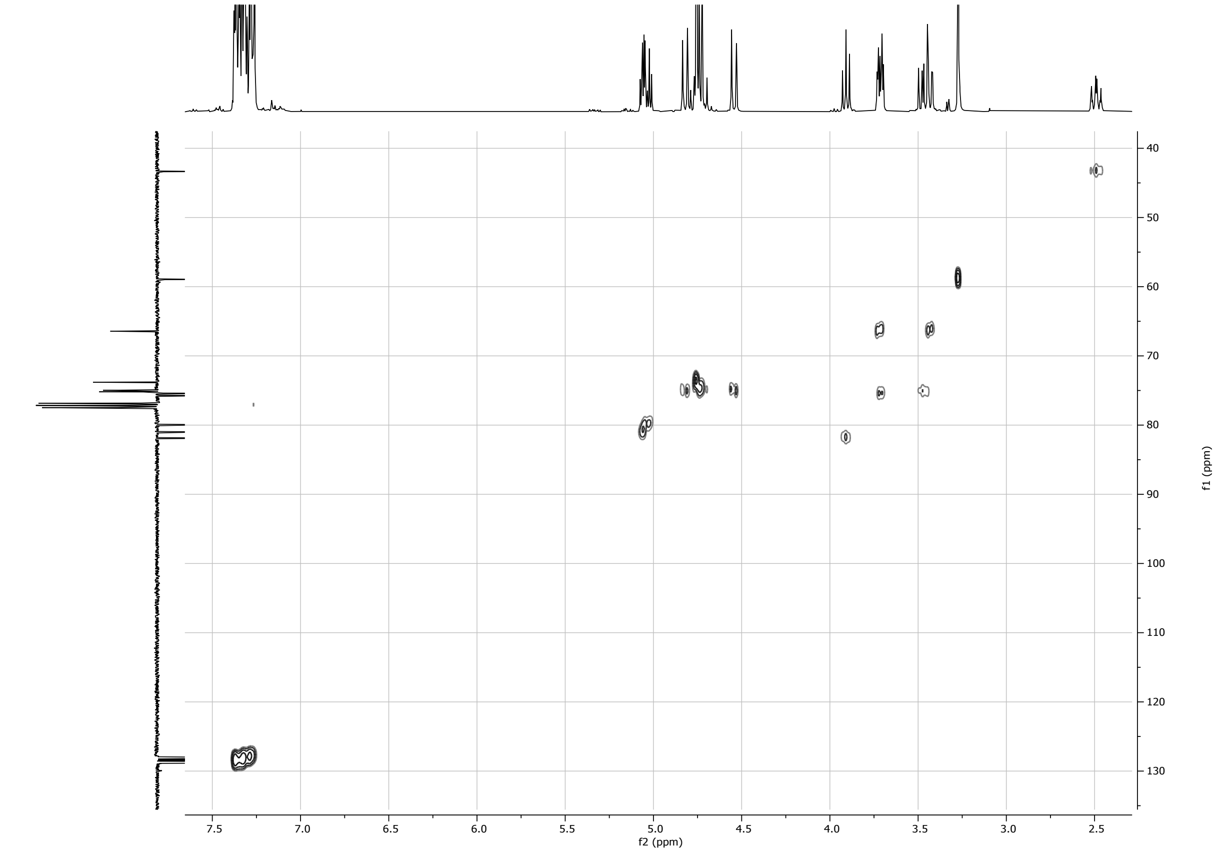

^{1}H NMR, 500MHz, MeOD of **6**

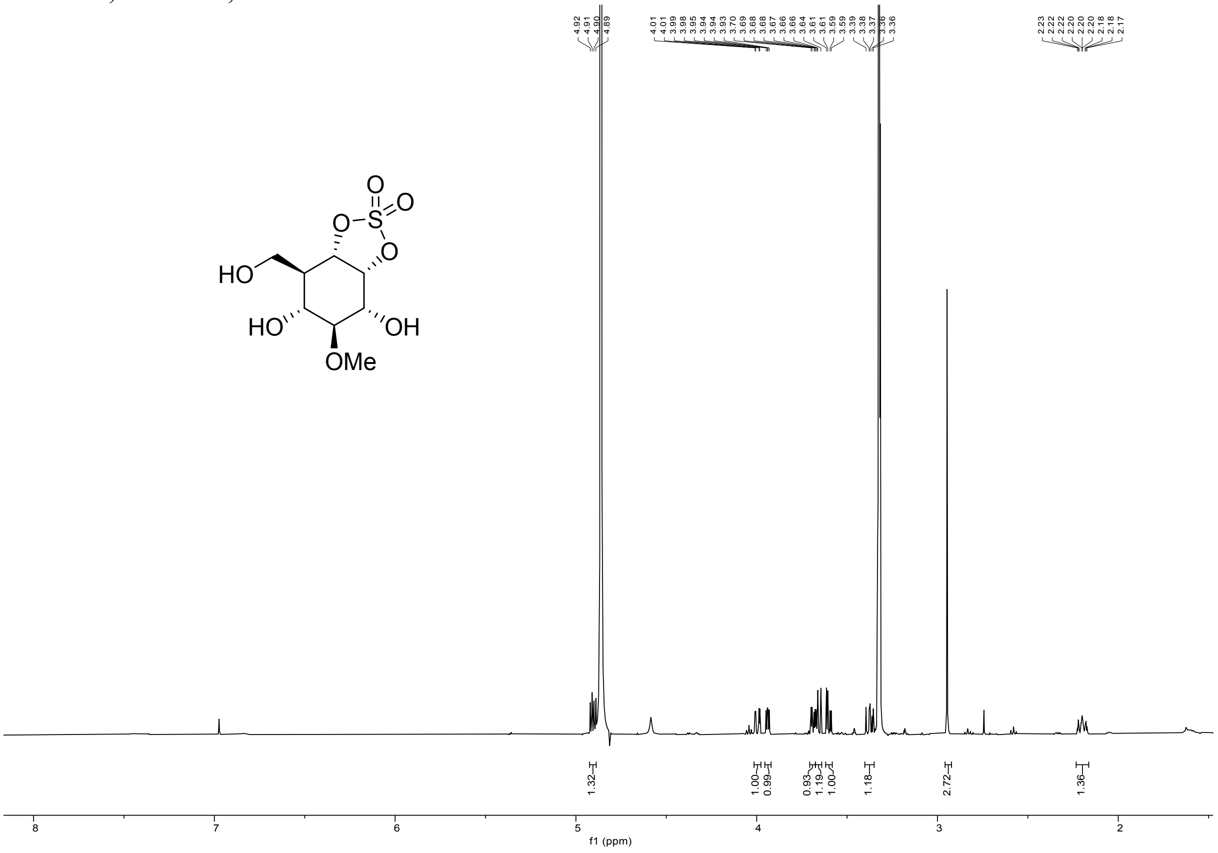

^{13}C NMR, 126MHz, MeOD of **6**

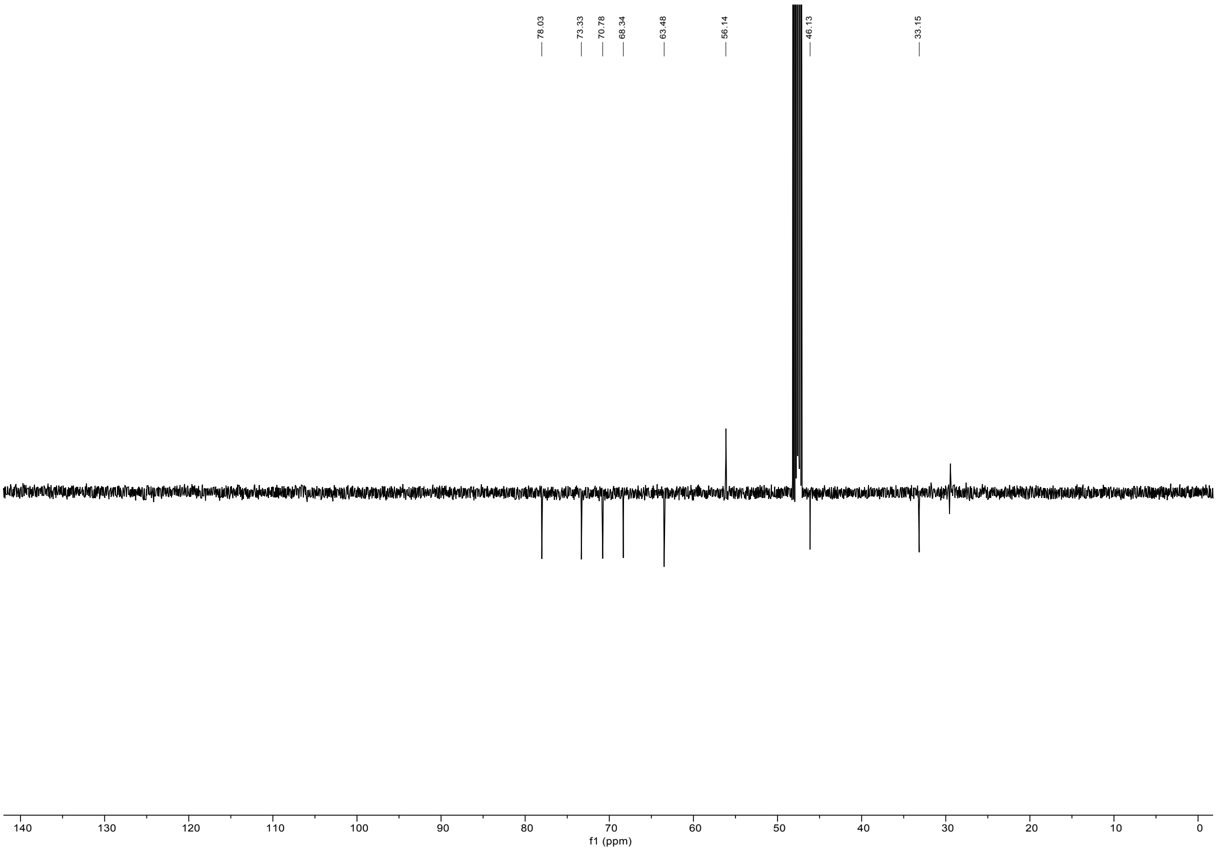

HH-COSY NMR, MeOD of **6**

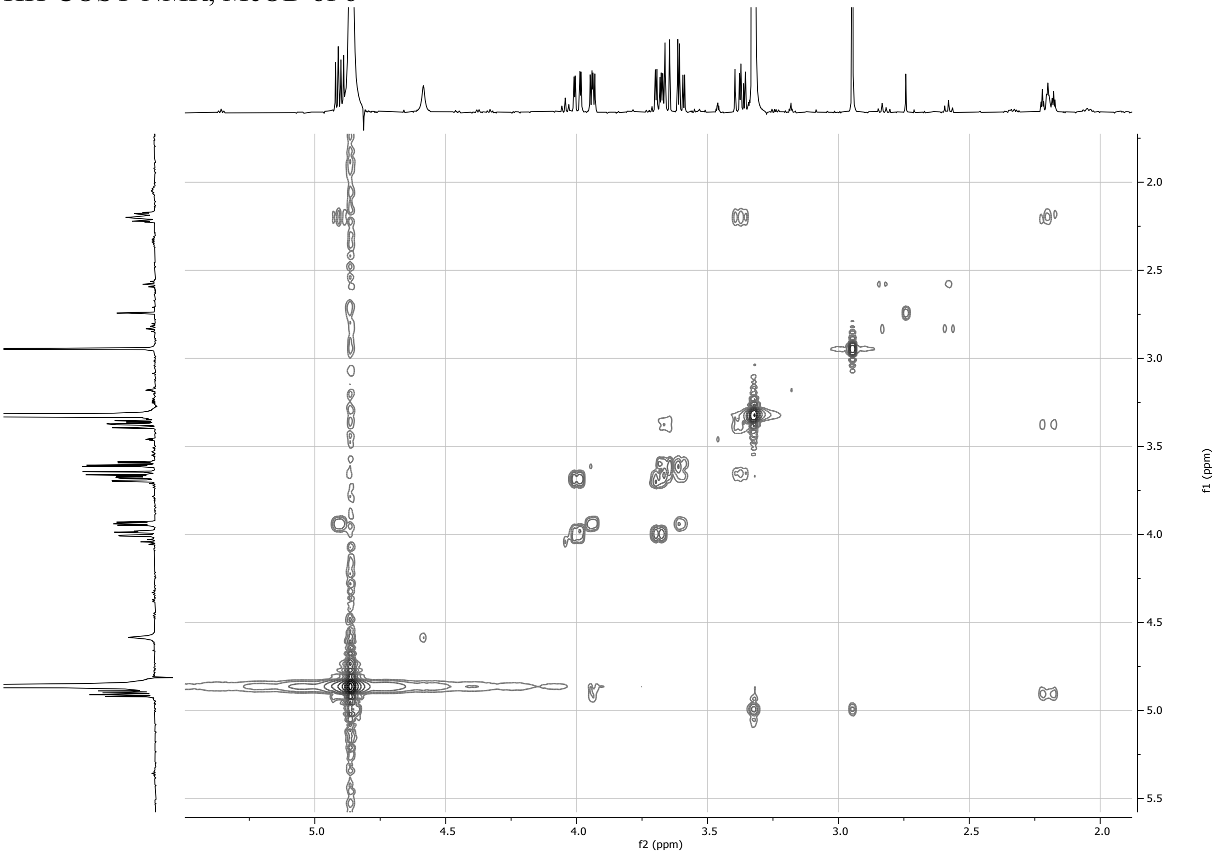

HSQC NMR, MeOD of **6**

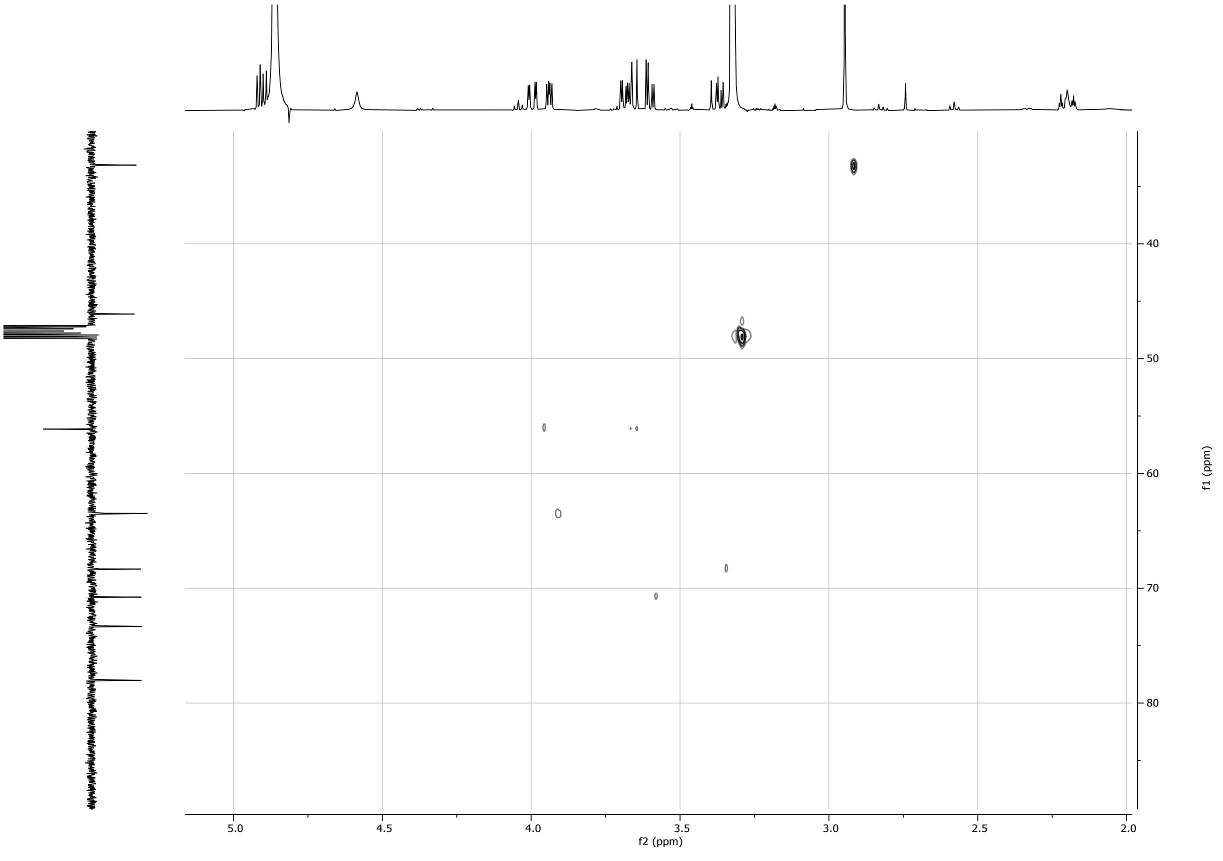

^{1}H NMR, 400MHz, $CDCl_3$ of **40**

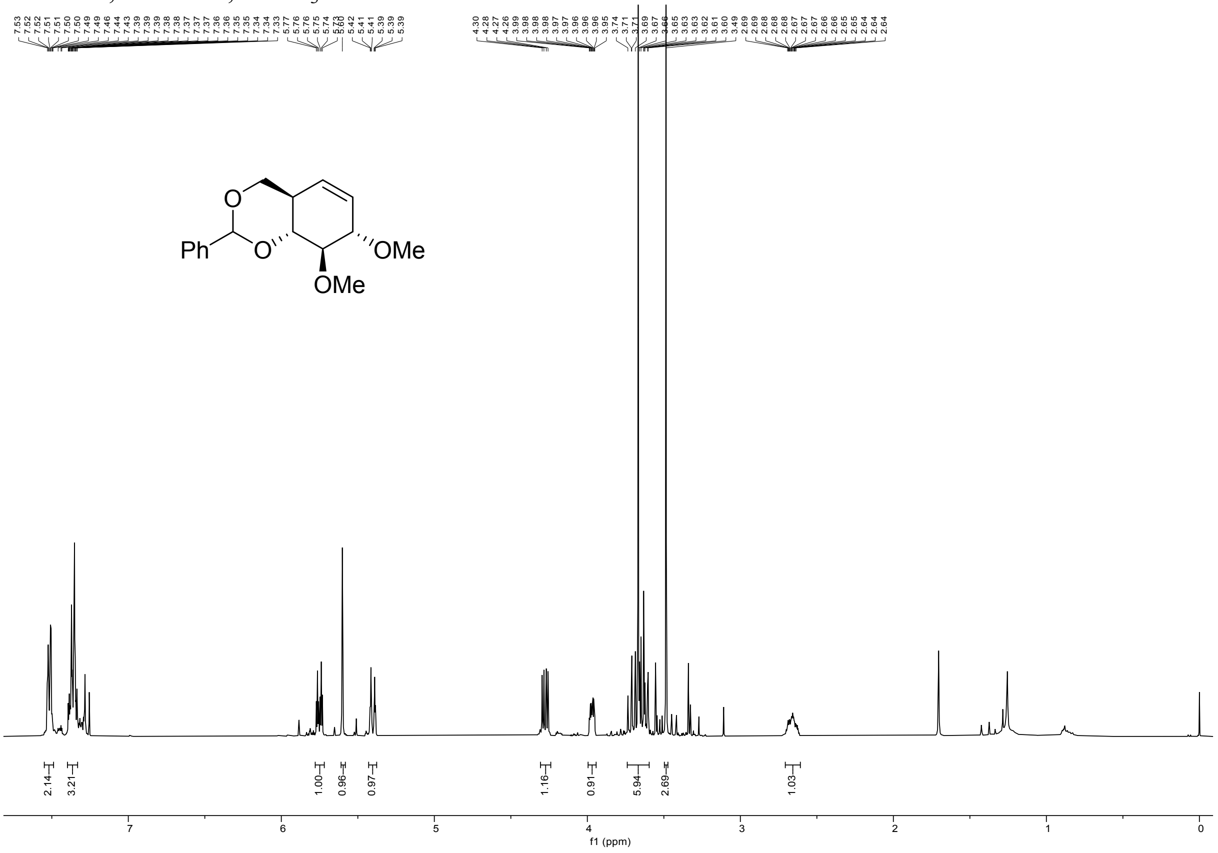

^{13}C NMR, 101MHz, $CDCl_3$ of **40**

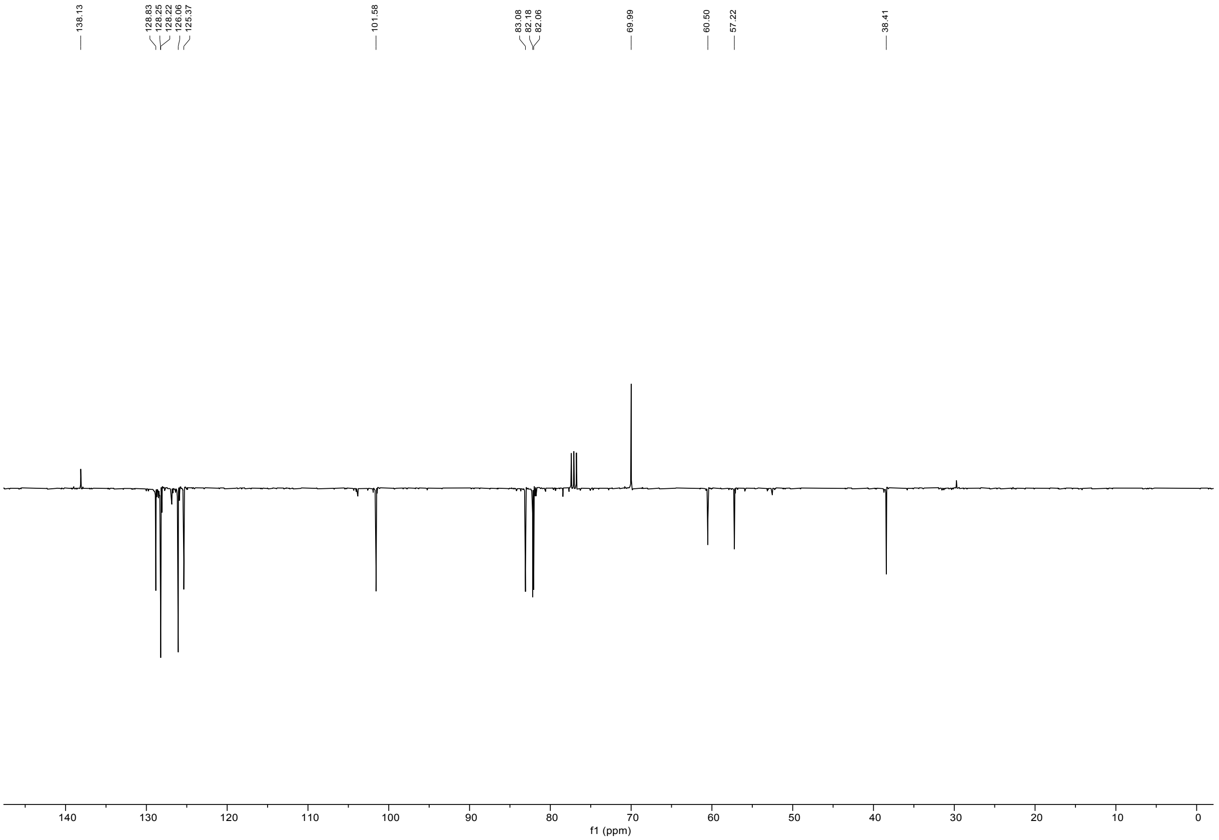

HH-COSY NMR, $CDCl_3$ of **40**

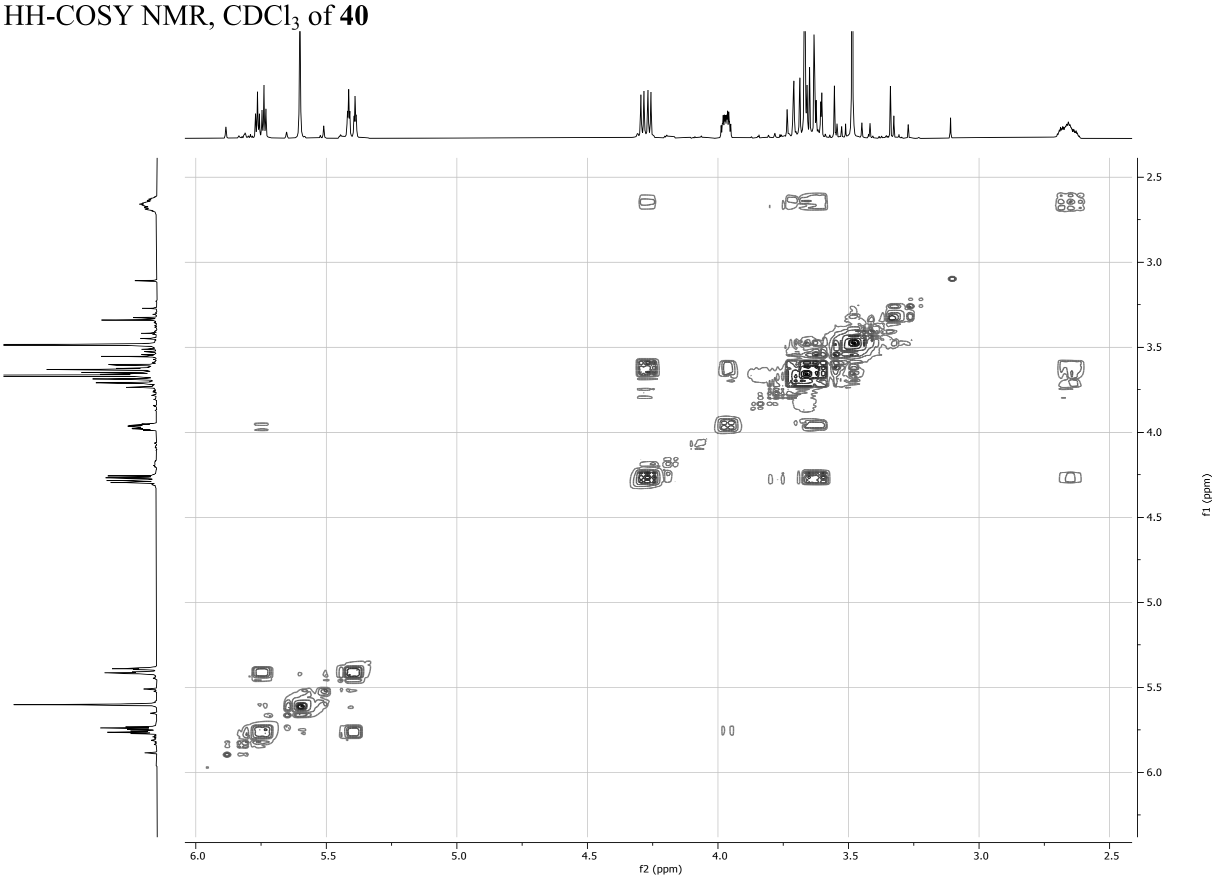

HSQC NMR, $CDCl_3$ of **40**

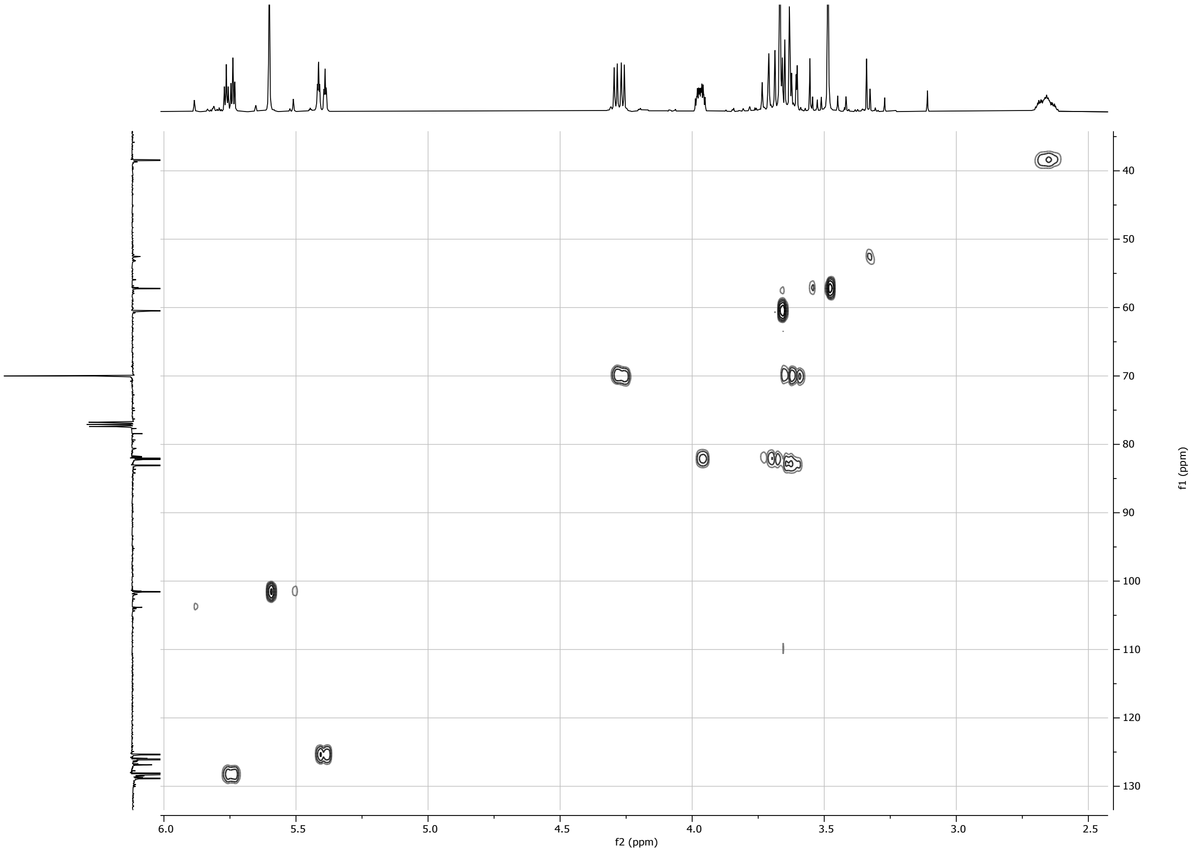

^{1}H NMR, 400MHz, MeOD of **41**

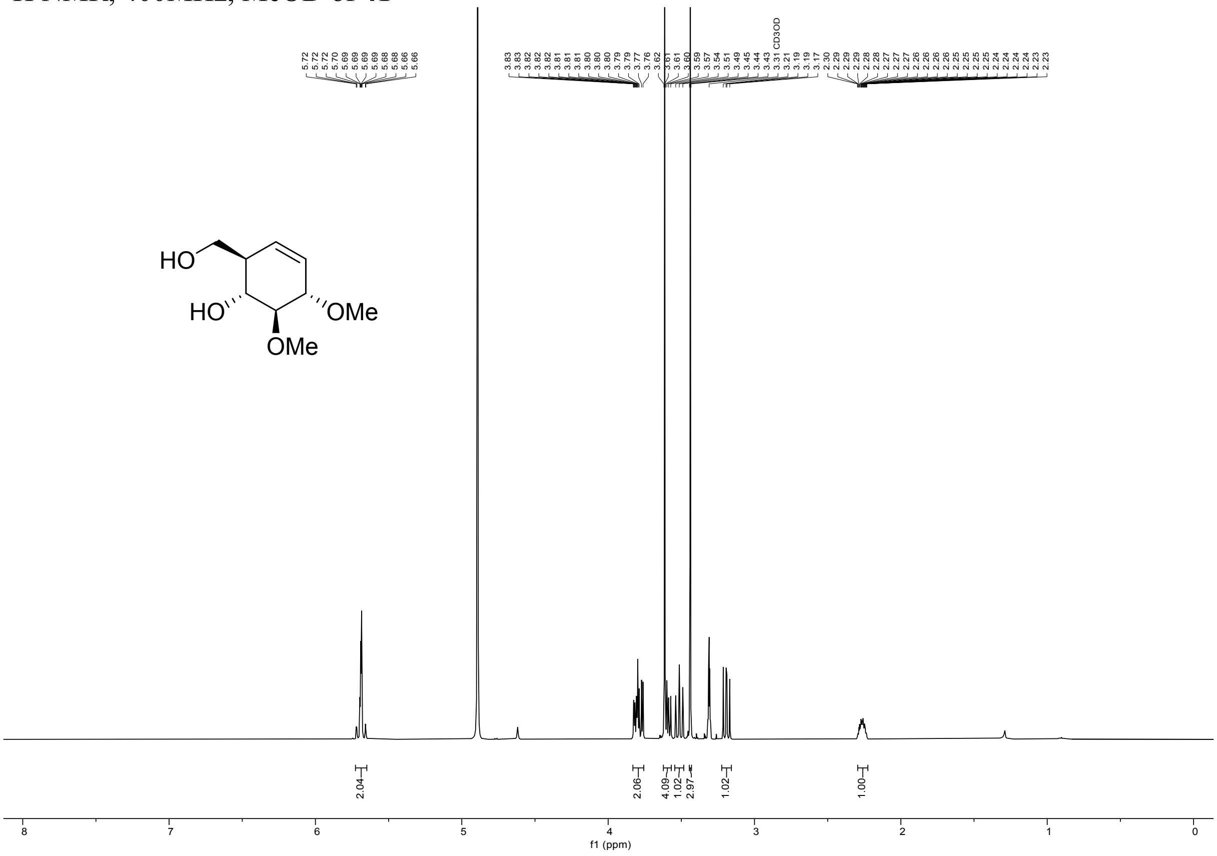

^{13}C NMR, 101MHz, MeOD of **41**

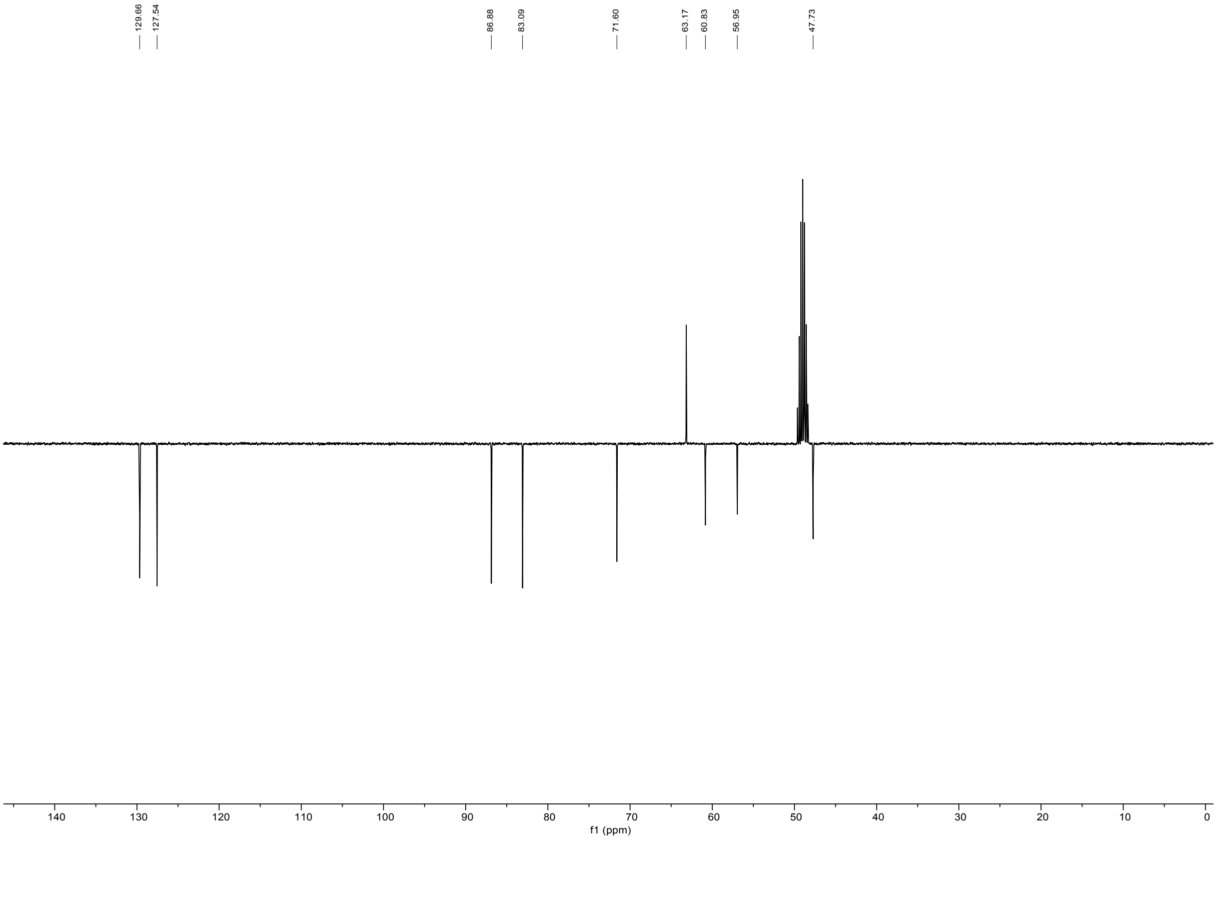

HH-COSY NMR, MeOD of **41**

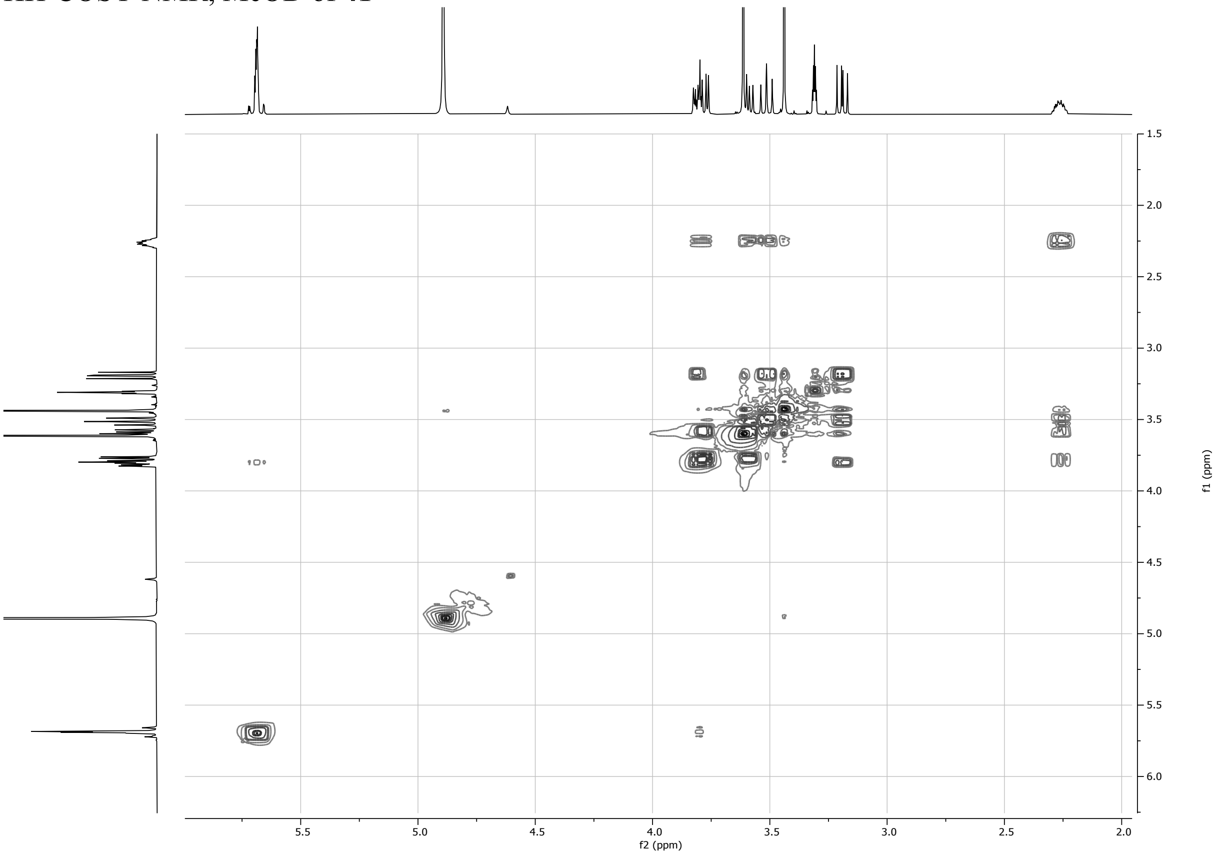

HSQC NMR, MeOD of **41**

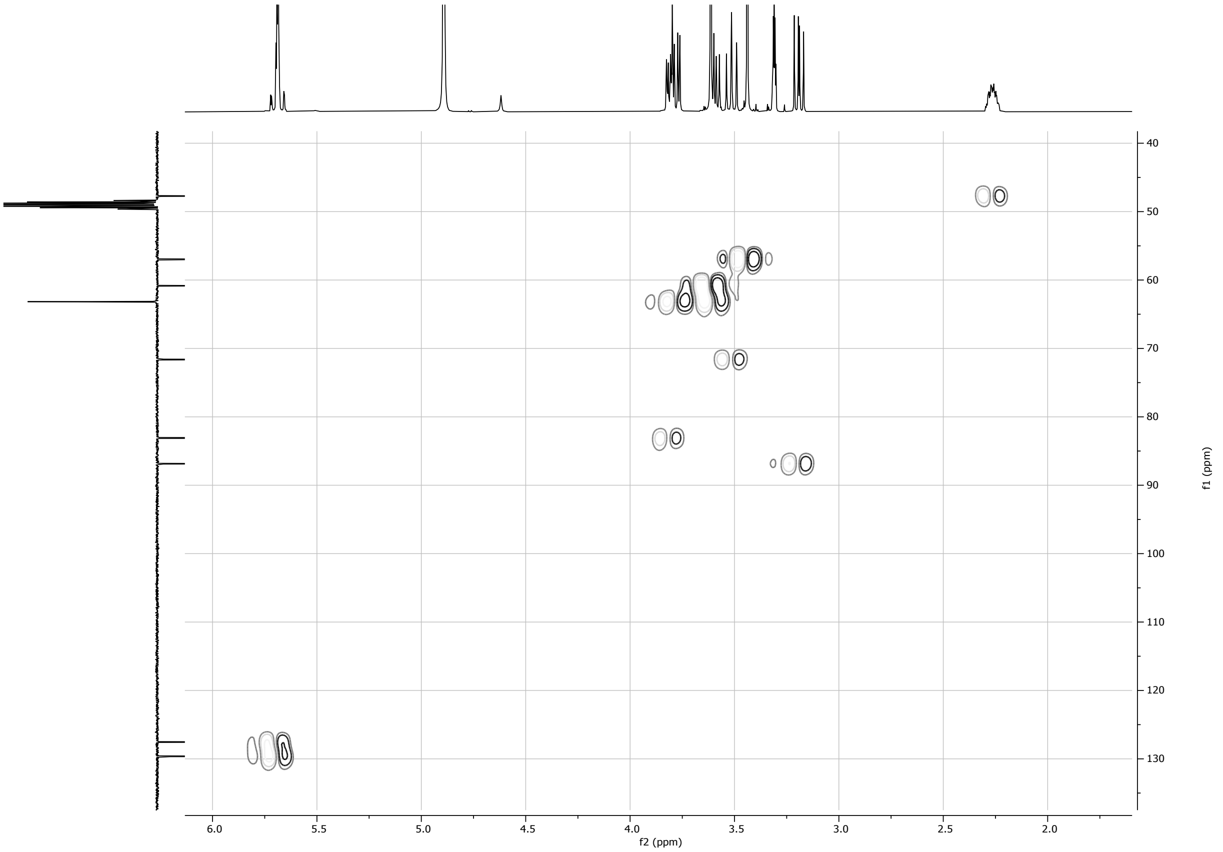

^{1}H NMR, 400MHz, $CDCl_3$ of **42**

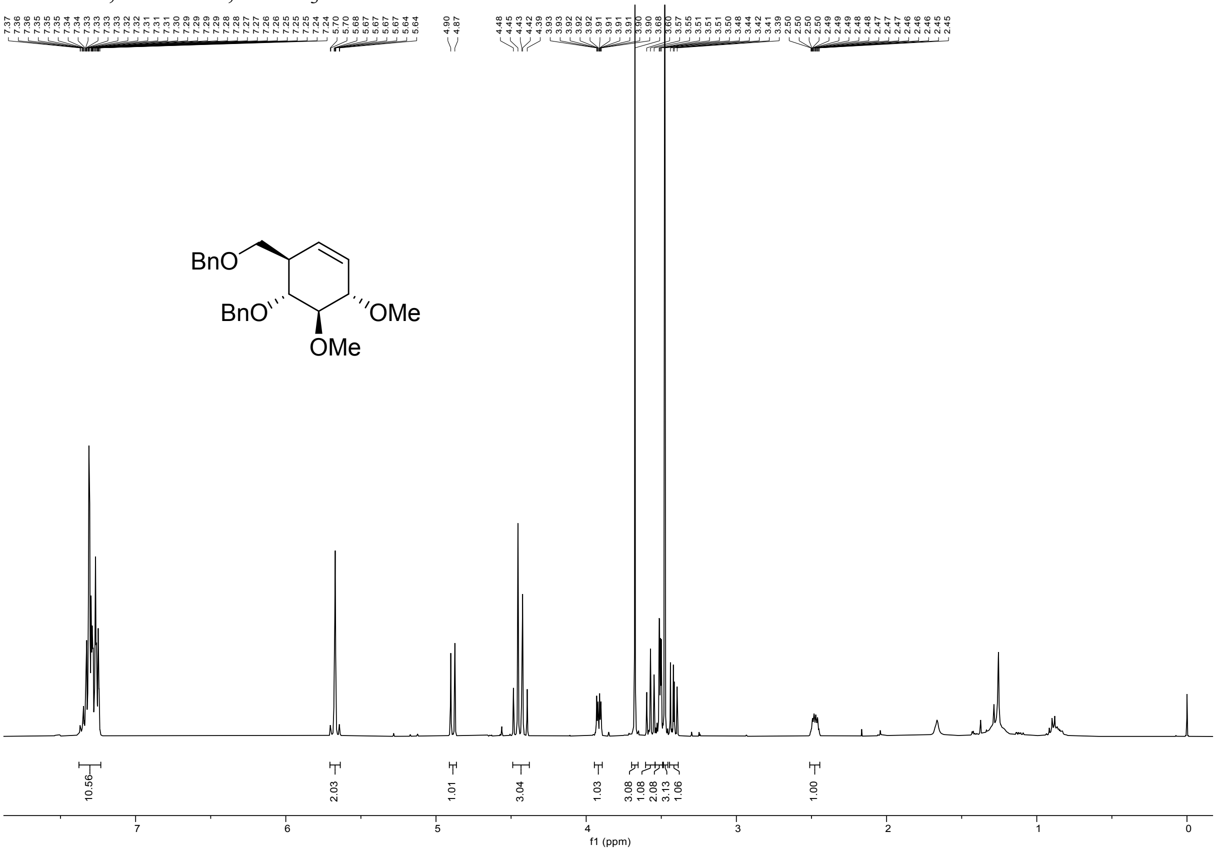

^{13}C NMR, 101MHz, $CDCl_3$ of **42**

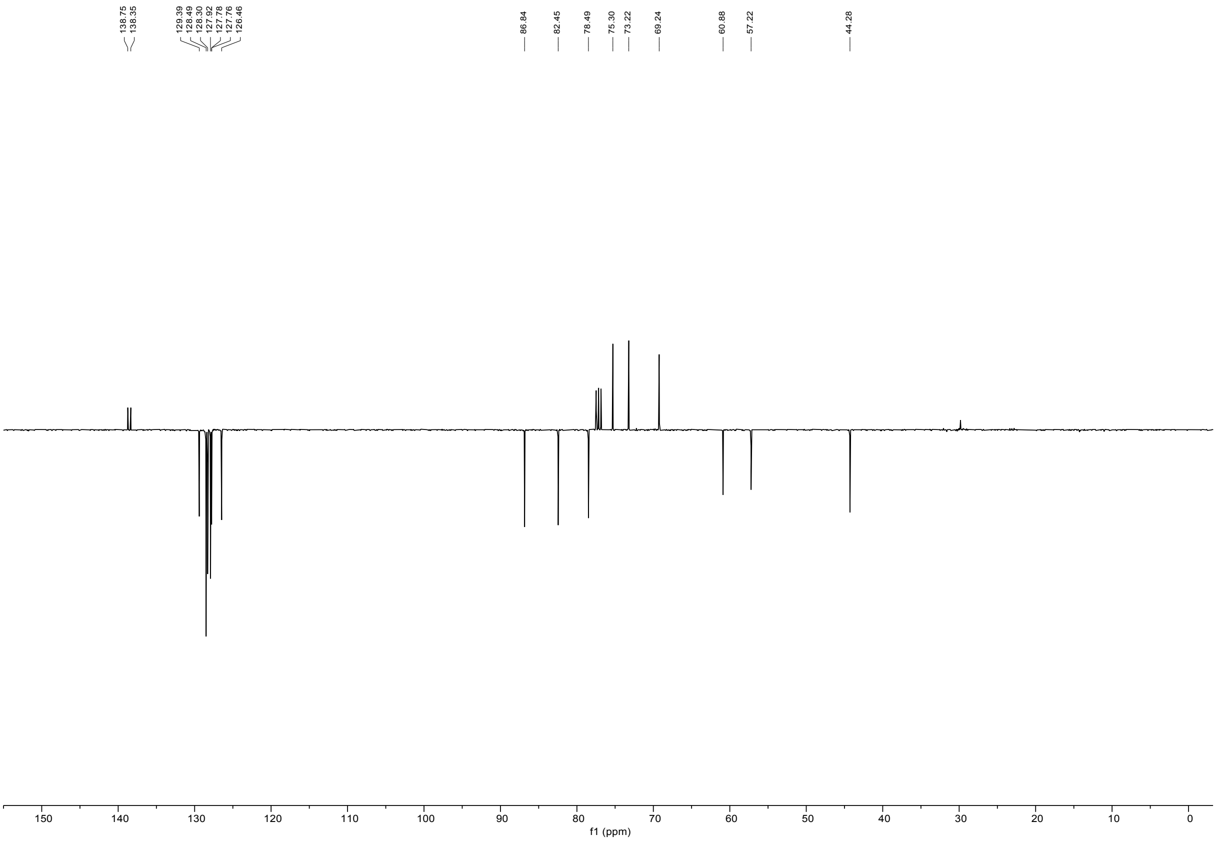

HH-COSY NMR, $CDCl_3$ of **42**

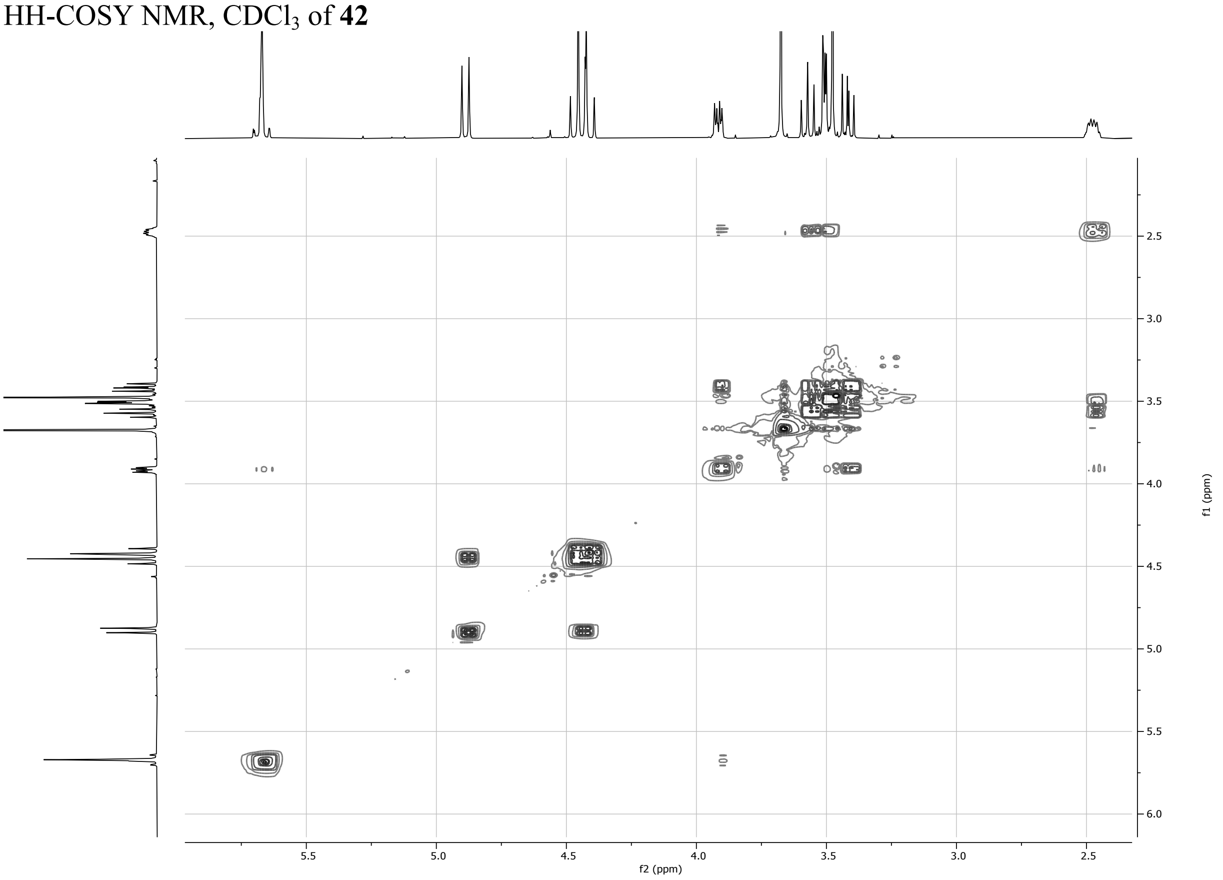

HSQC NMR, $CDCl_3$ of **42**

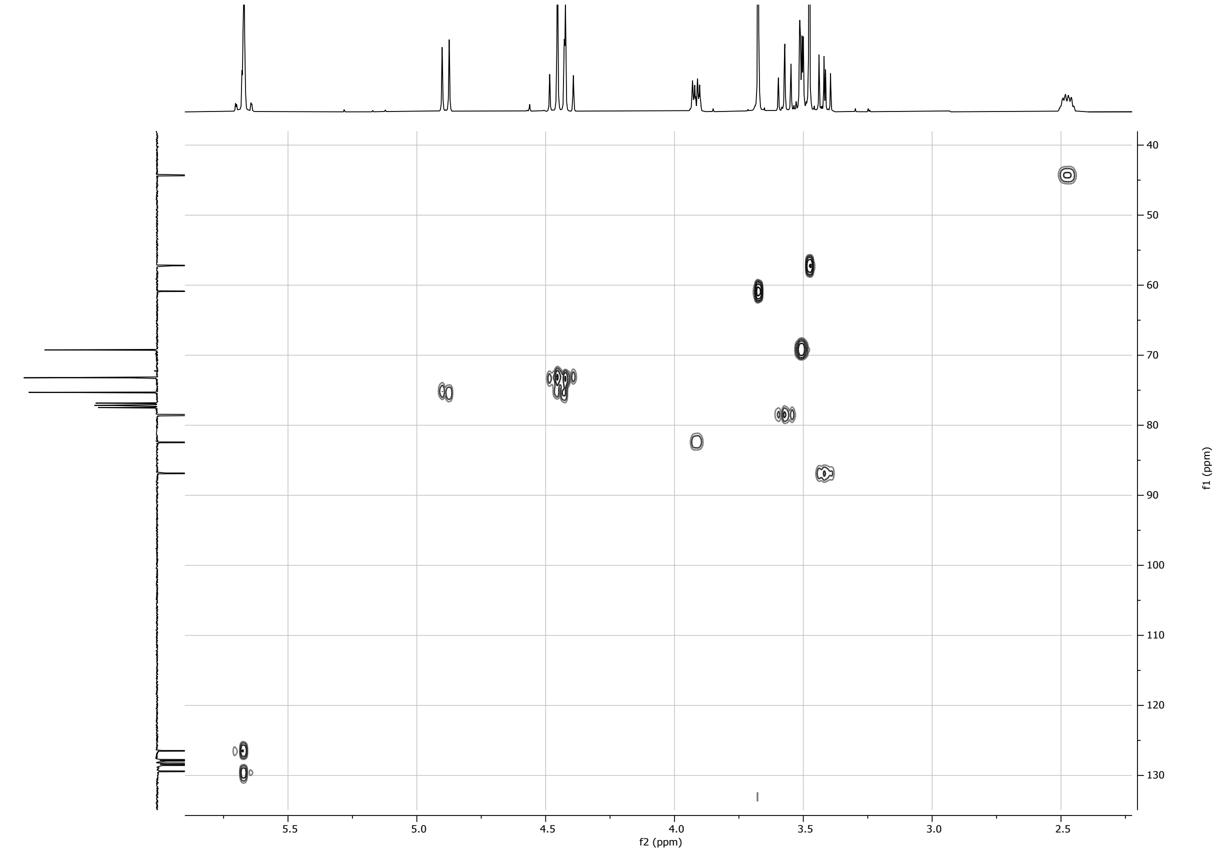

^{1}H NMR, 400MHz, $CDCl_3$ of **43**

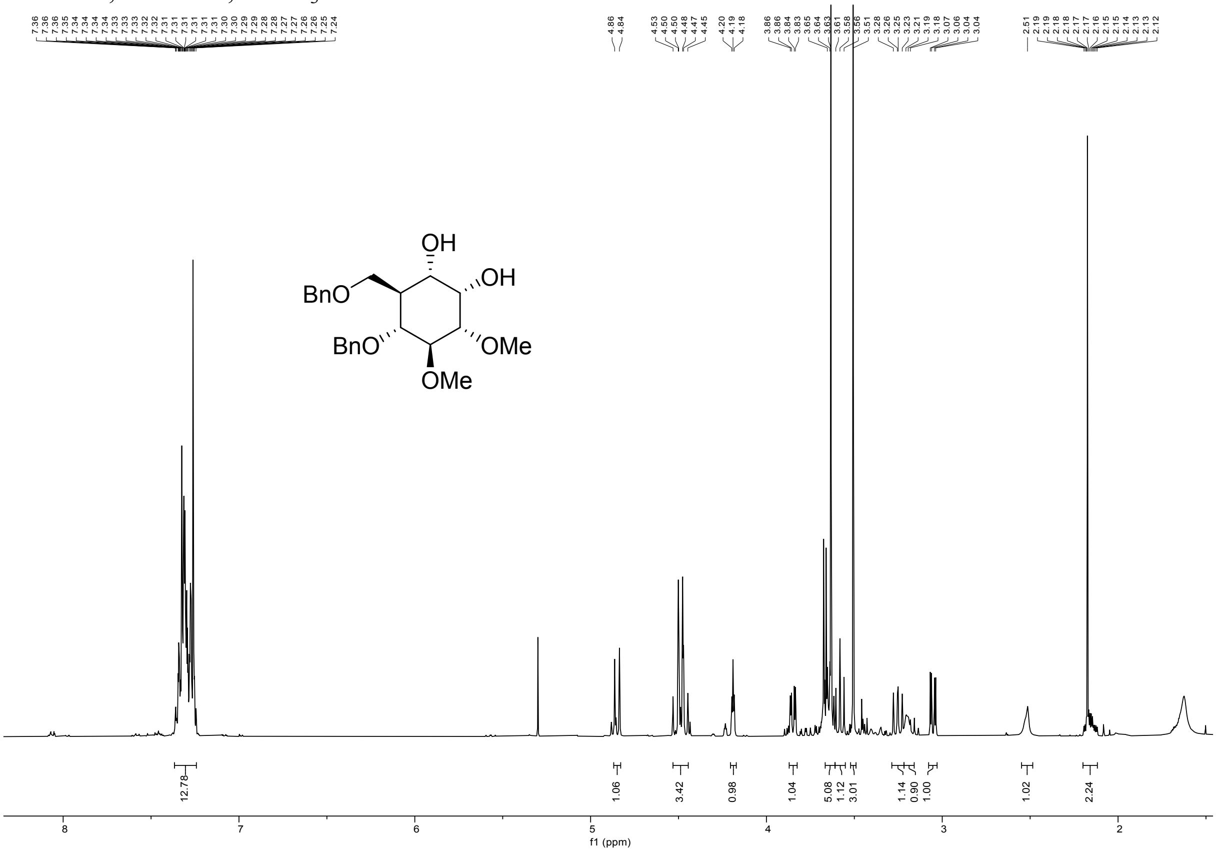

^{13}C NMR, 101MHz, $CDCl_3$ of **43**

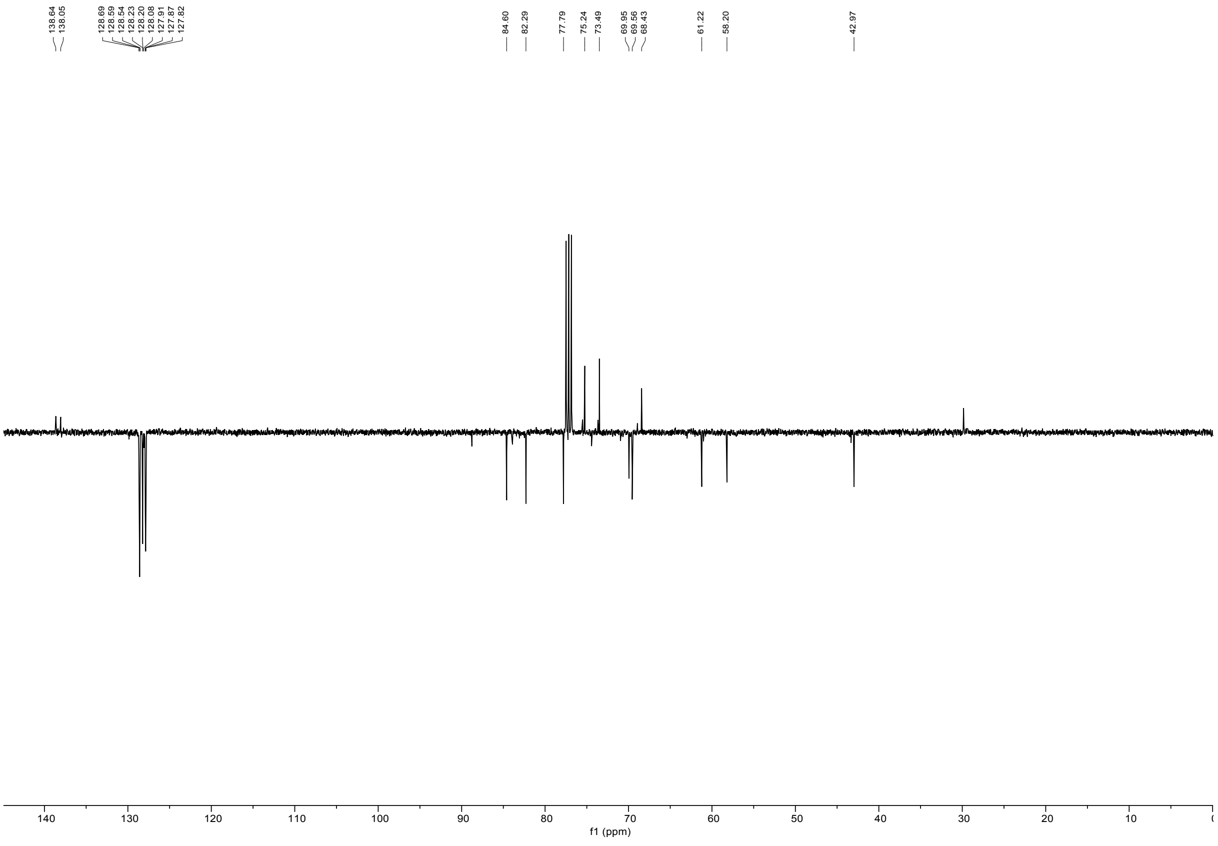

HH-COSY NMR, $CDCl_3$ of **43**

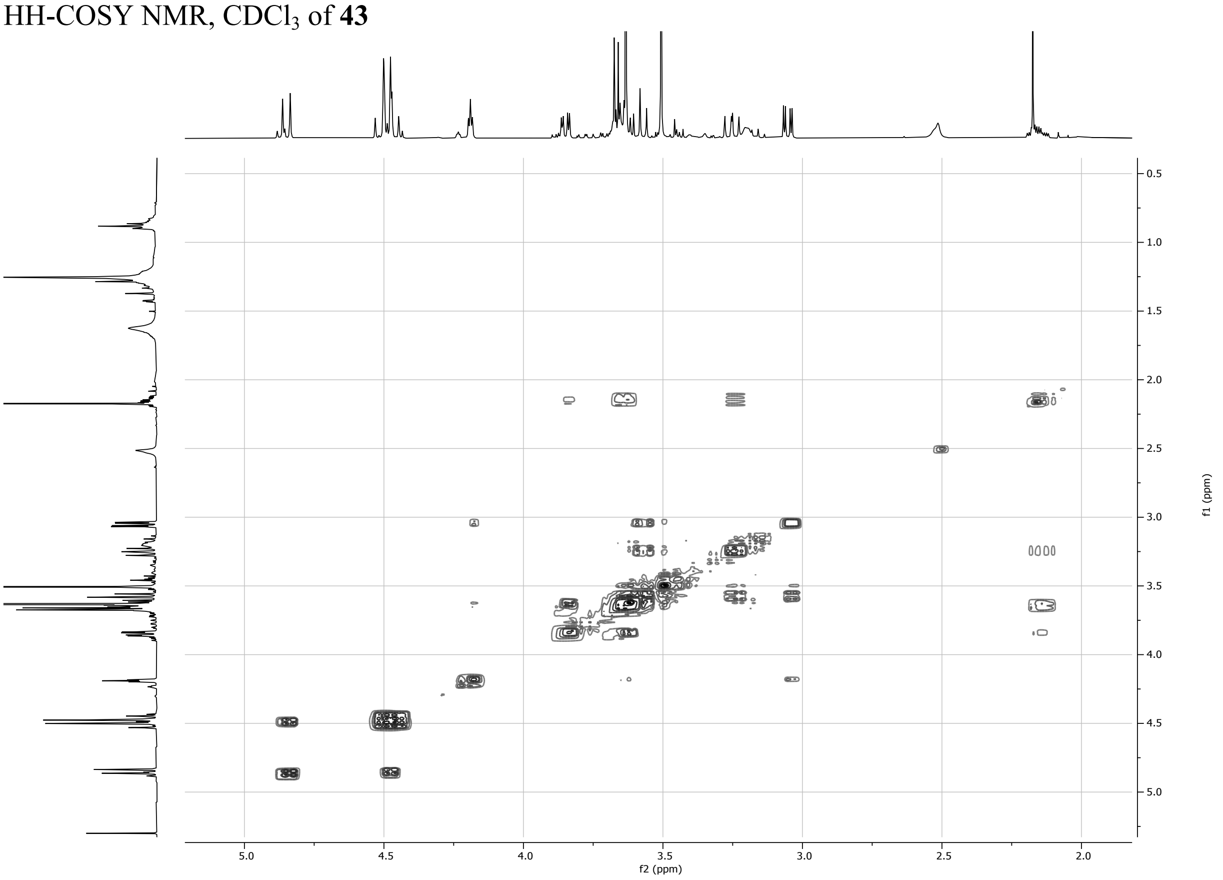

HSQC NMR, $CDCl_3$ of **43**

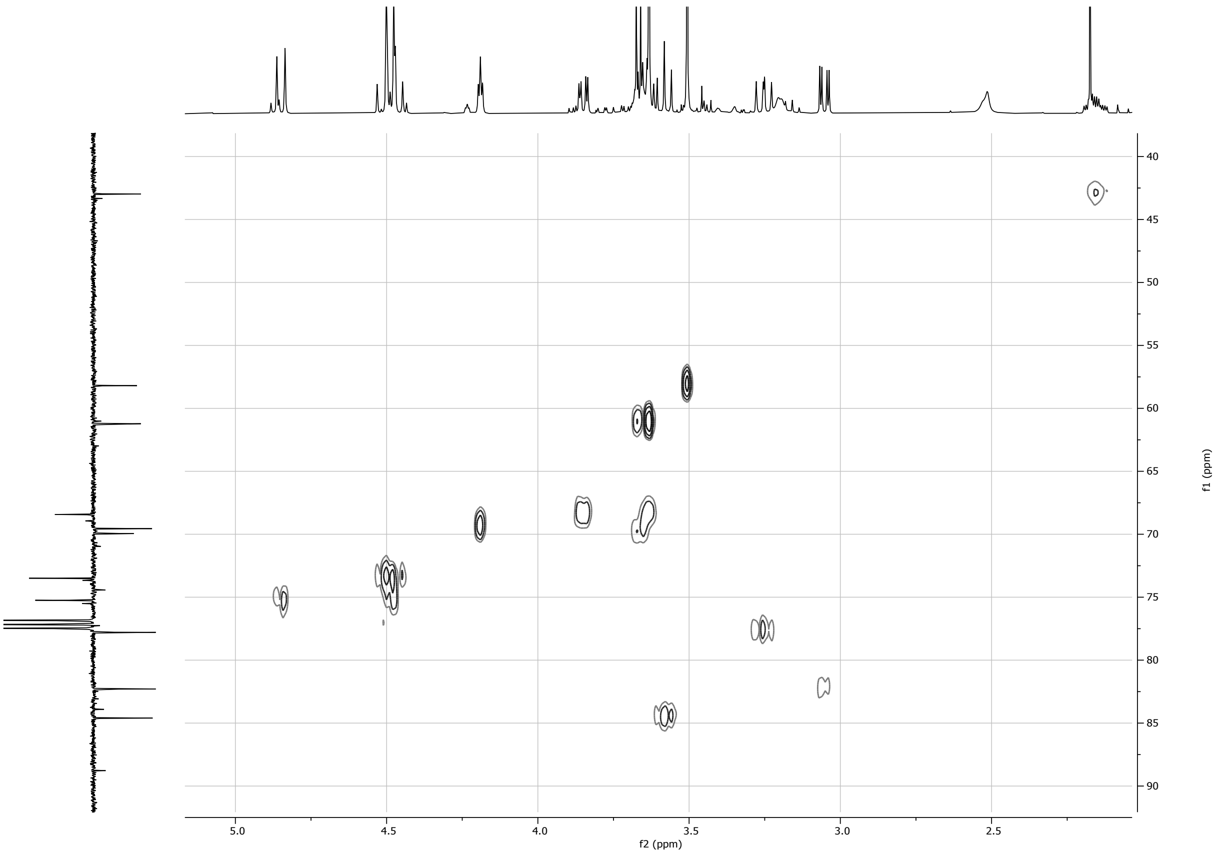

^{1}H NMR, 400MHz, $CDCl_3$ of **44**

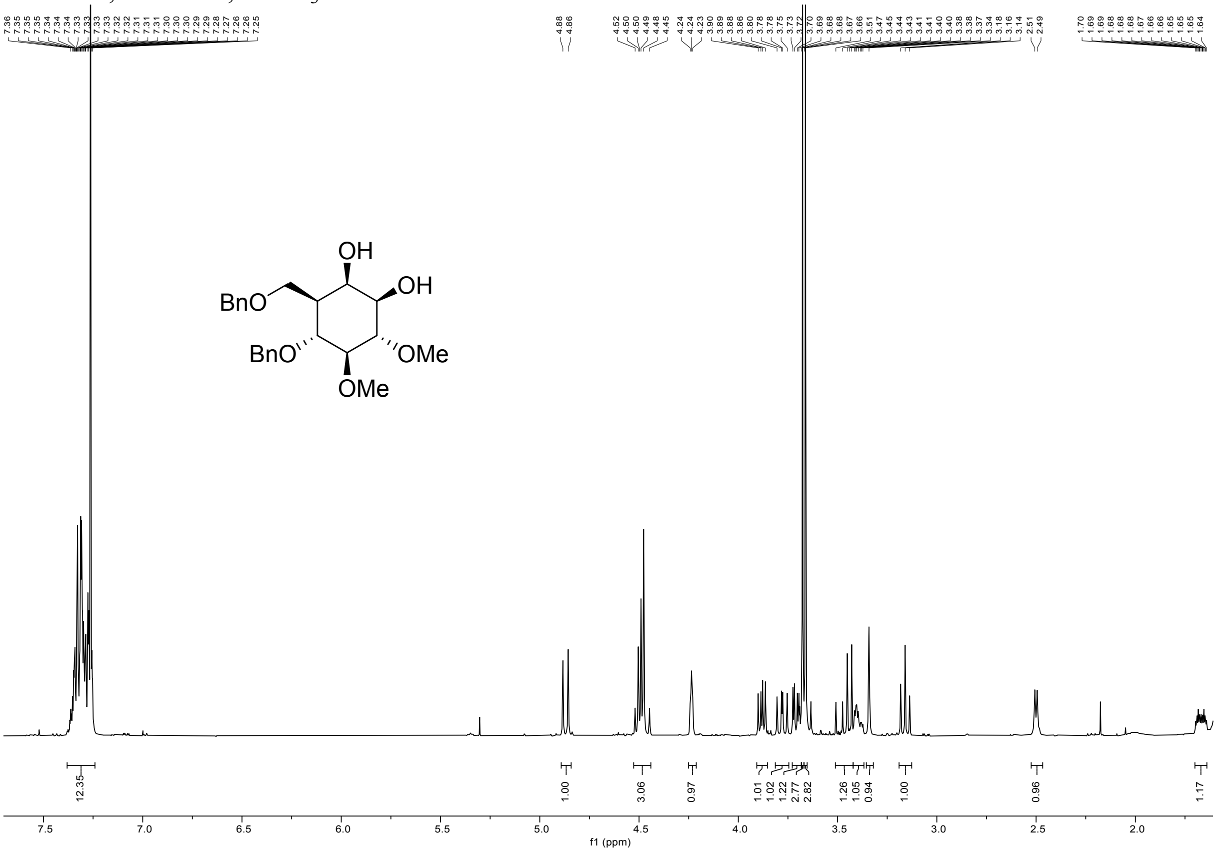

^{13}C NMR, 101MHz, $CDCl_3$ of **44**

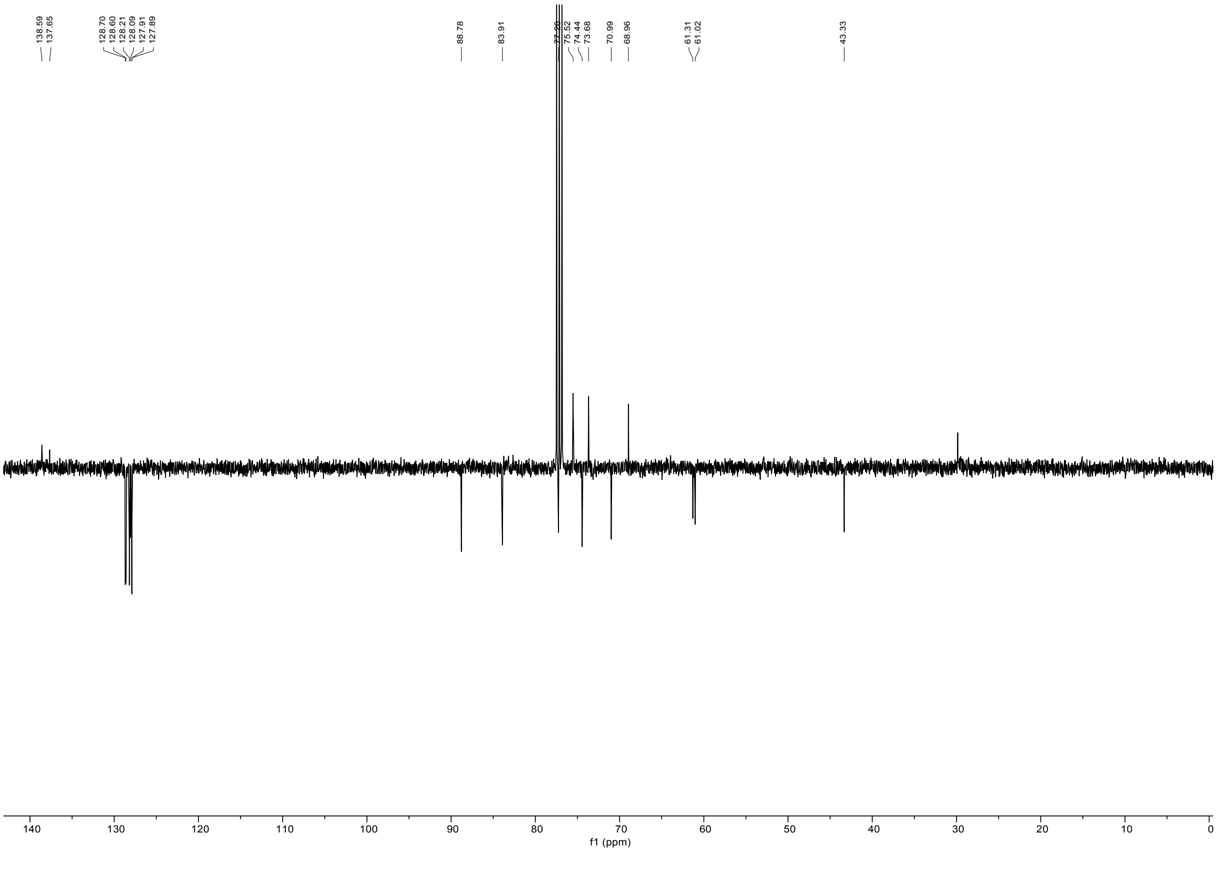

HH-COSY NMR, $CDCl_3$ of **44**

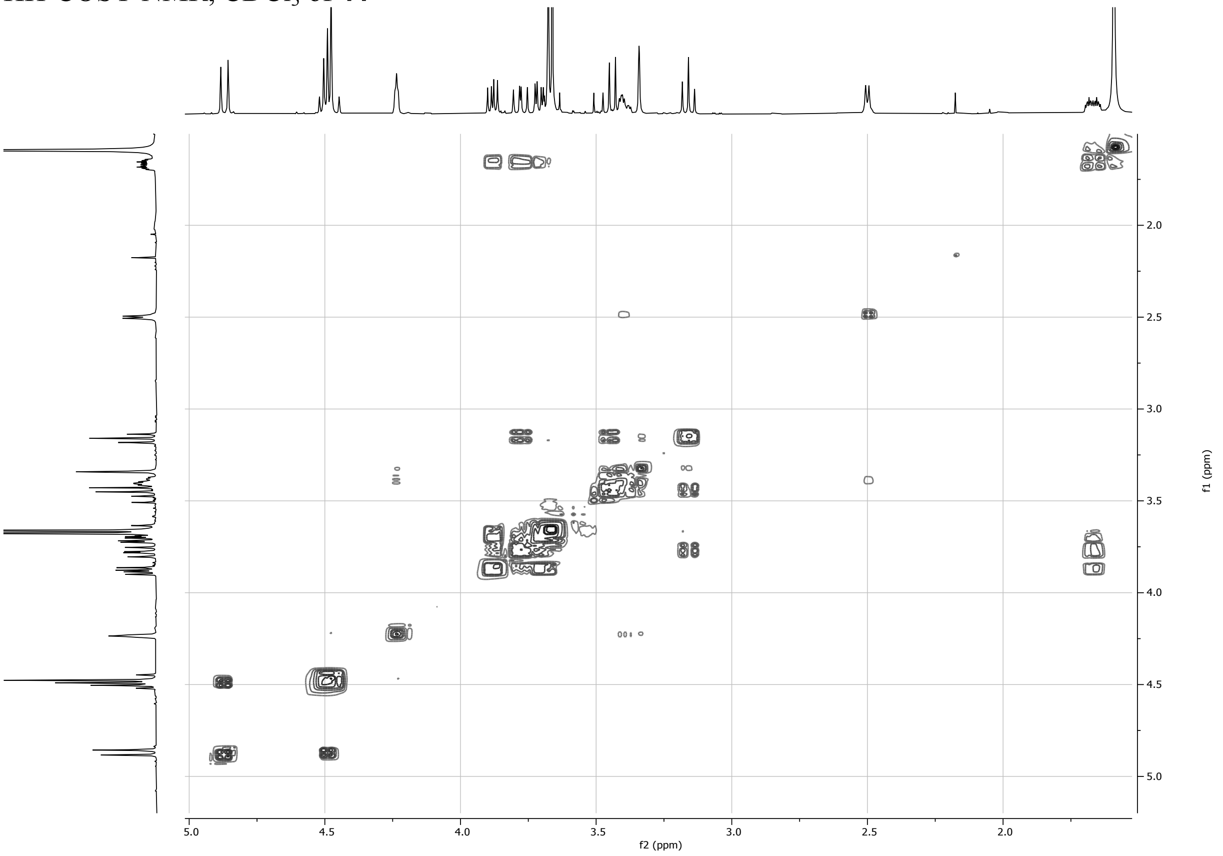

HSQC NMR, $CDCl_3$ of **44**

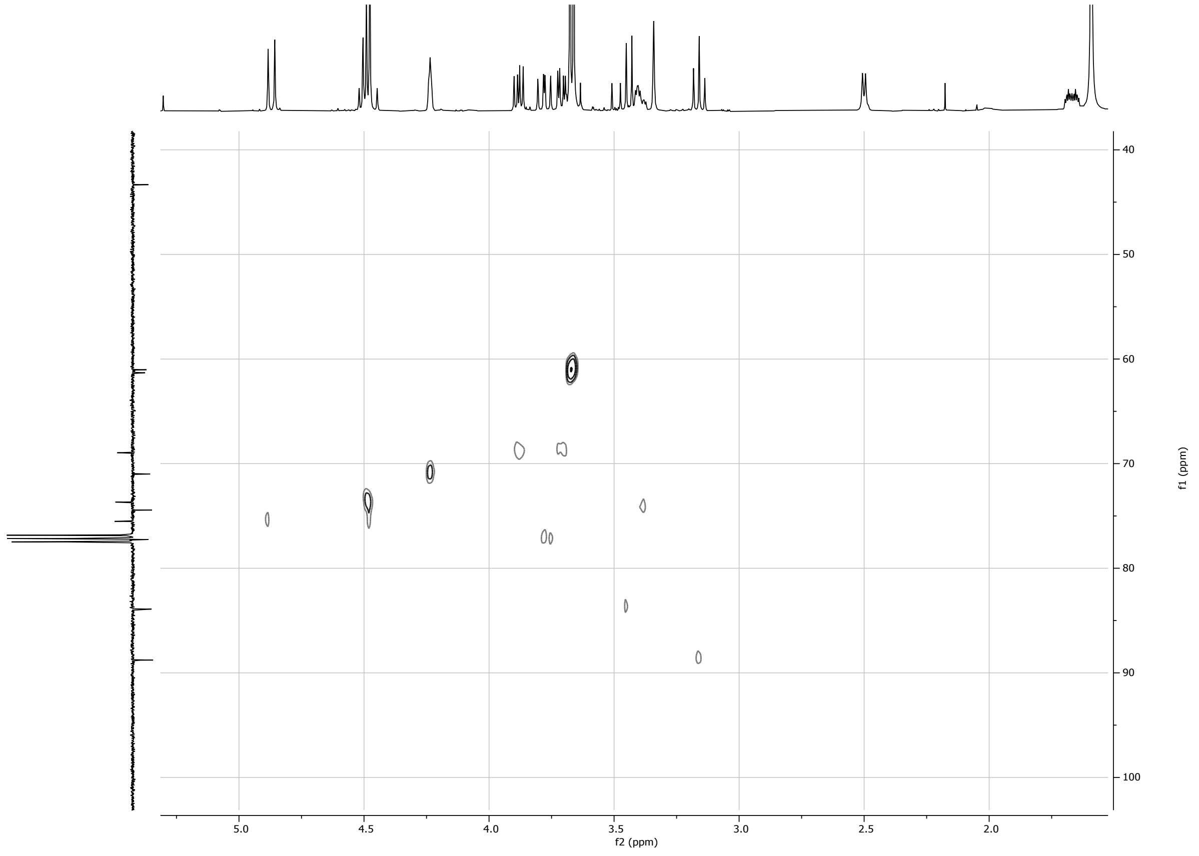

^{1}H NMR, 400MHz, $CDCl_3$ of **45**

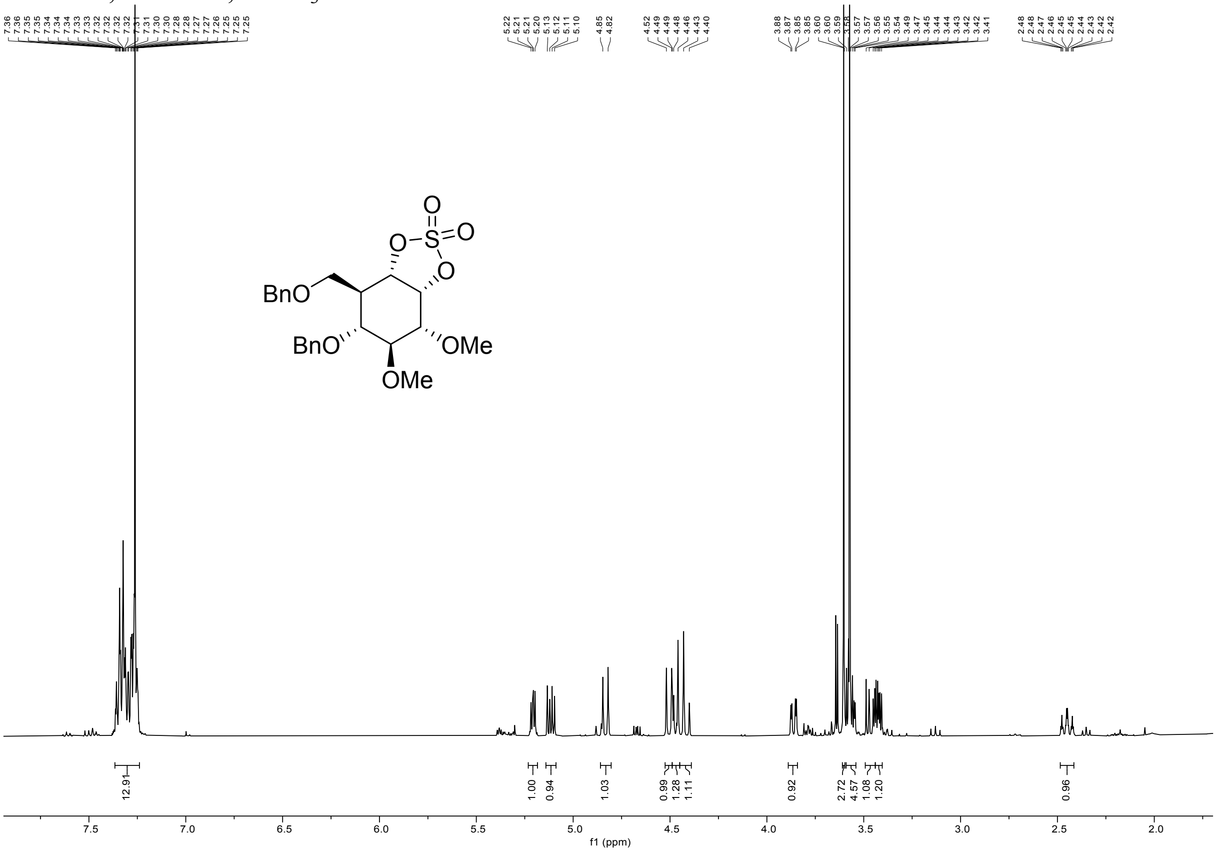

^{13}C NMR, 101MHz, $CDCl_3$ of **45**

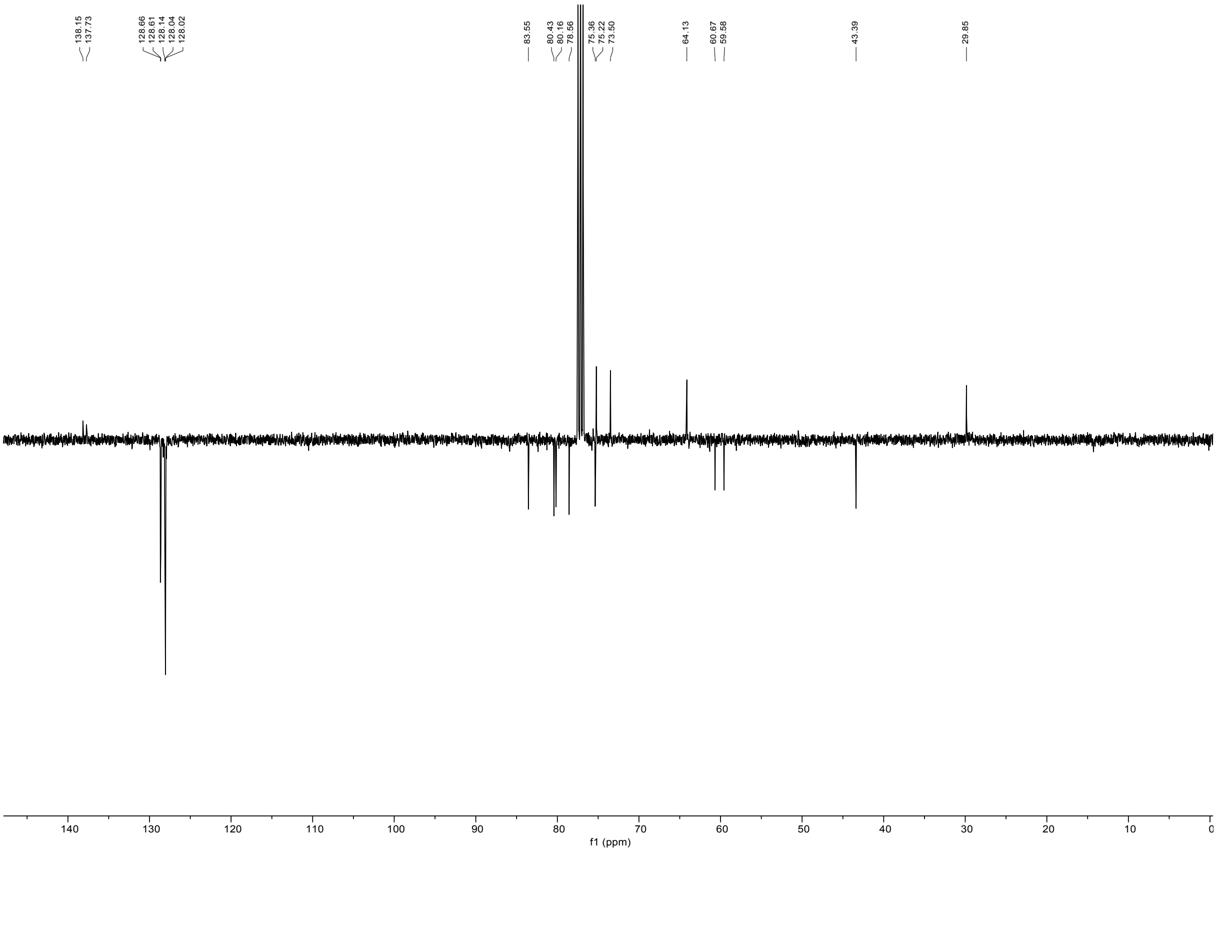

HH-COSY NMR, $CDCl_3$ of **45**

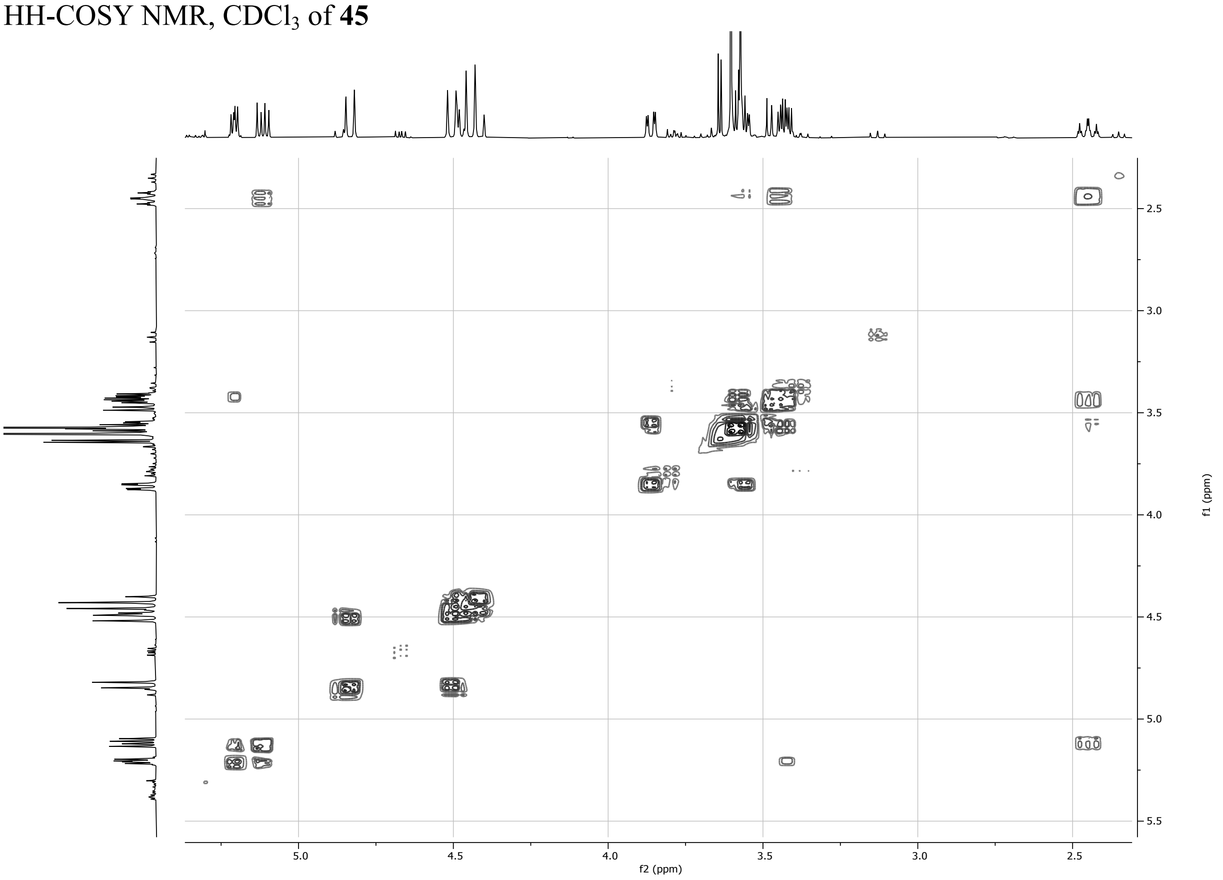

HSQC NMR, $CDCl_3$ of **45**

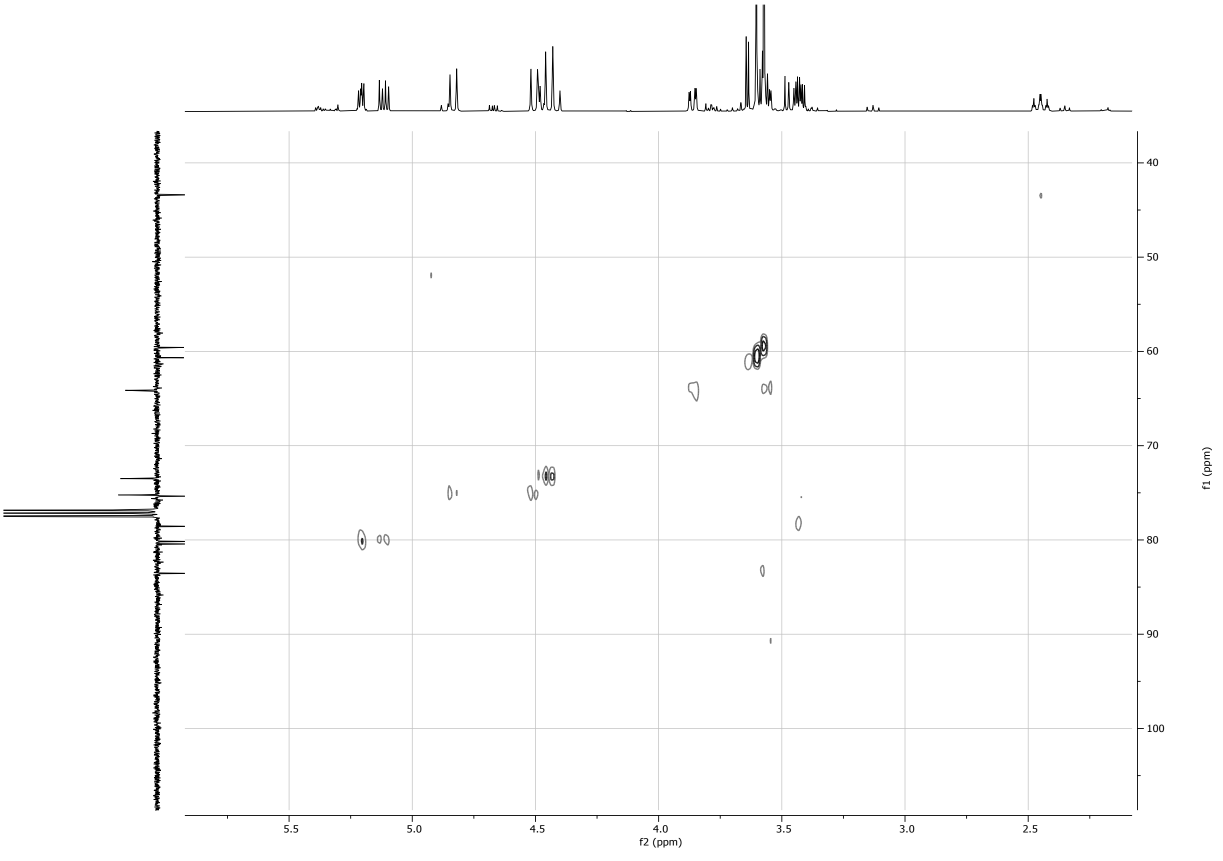

^{1}H NMR, 500MHz, MeOD of **9**

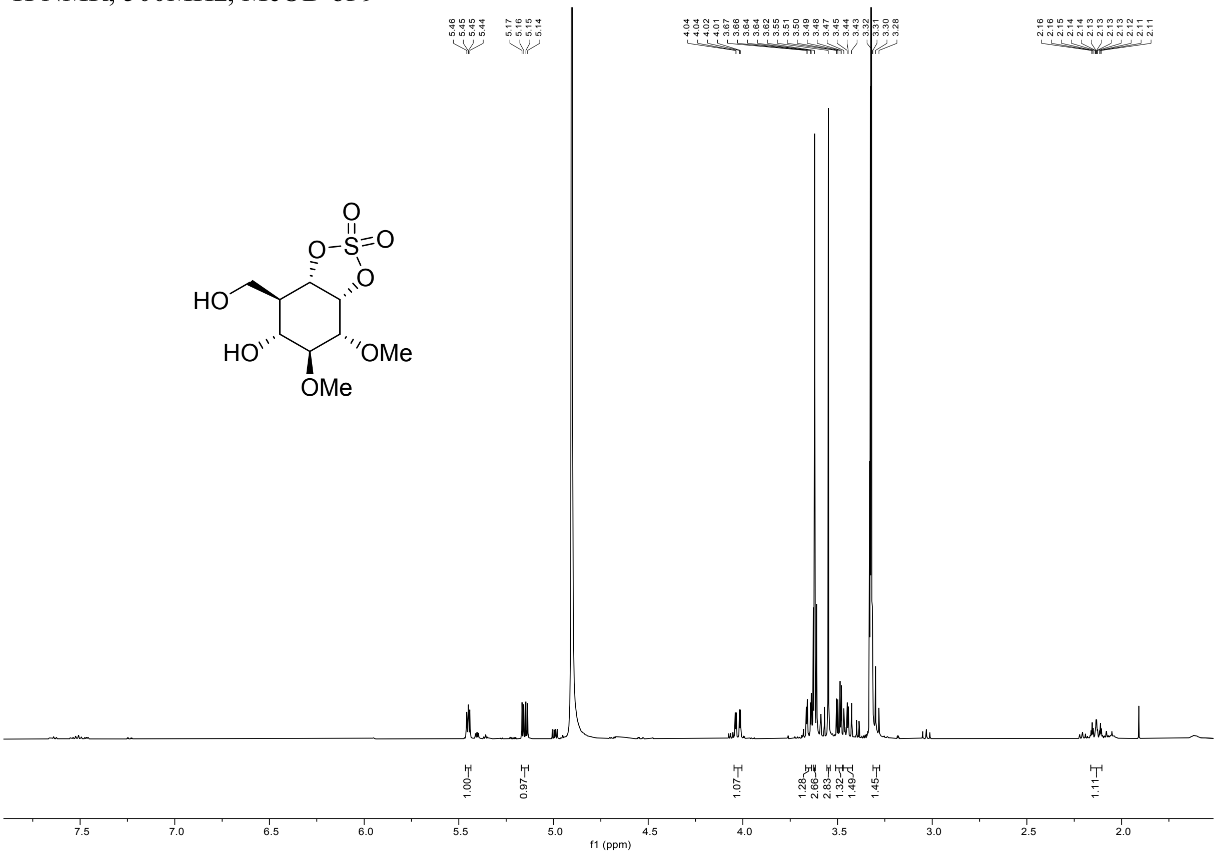

^{13}C NMR, 126MHz, MeOD of **9**

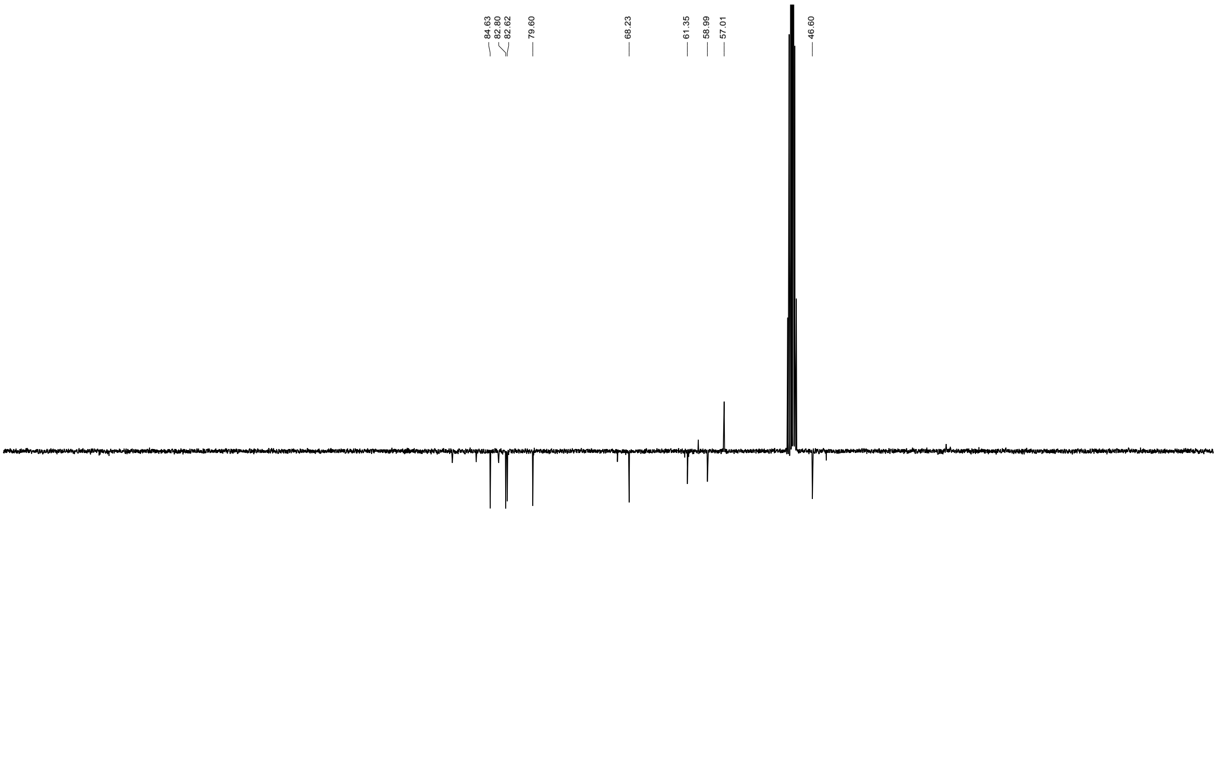

HH-COSY NMR, MeOD of **9**

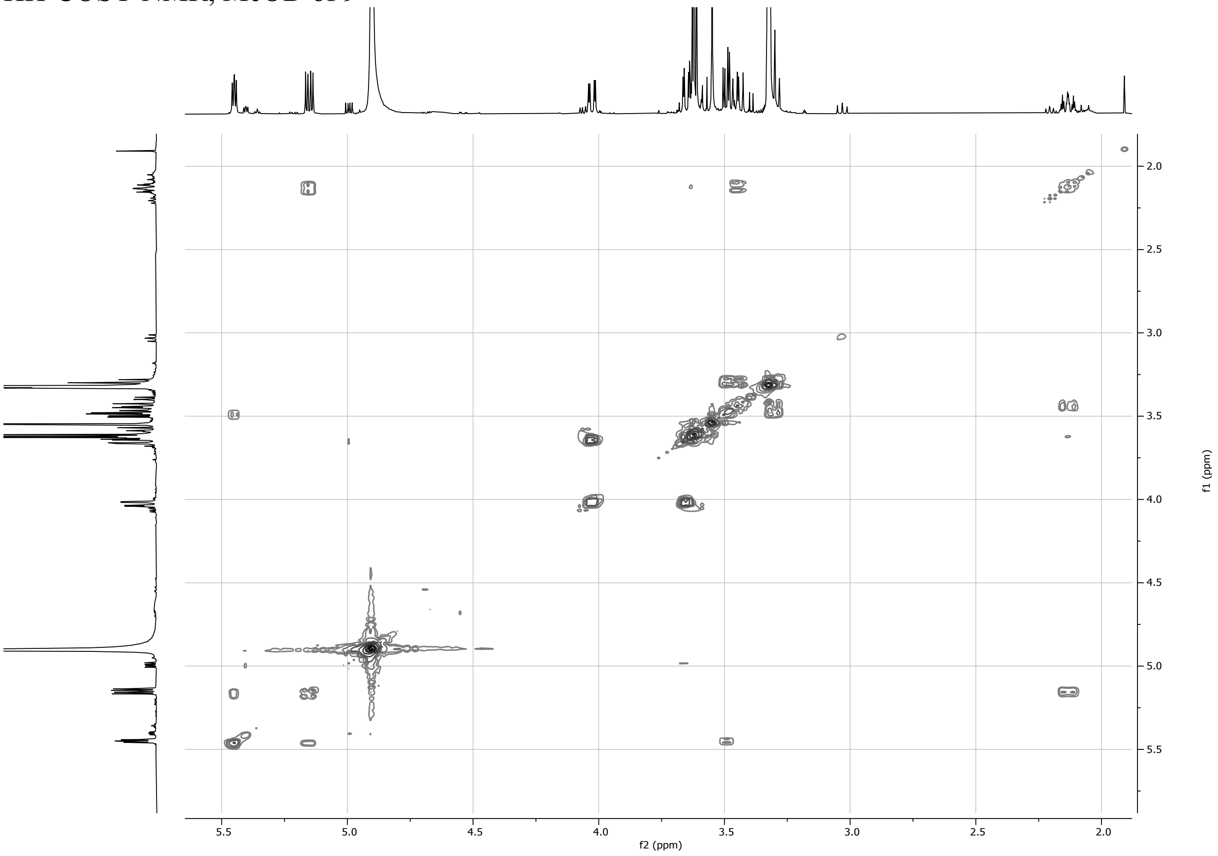

HSQC NMR, MeOD of **9**

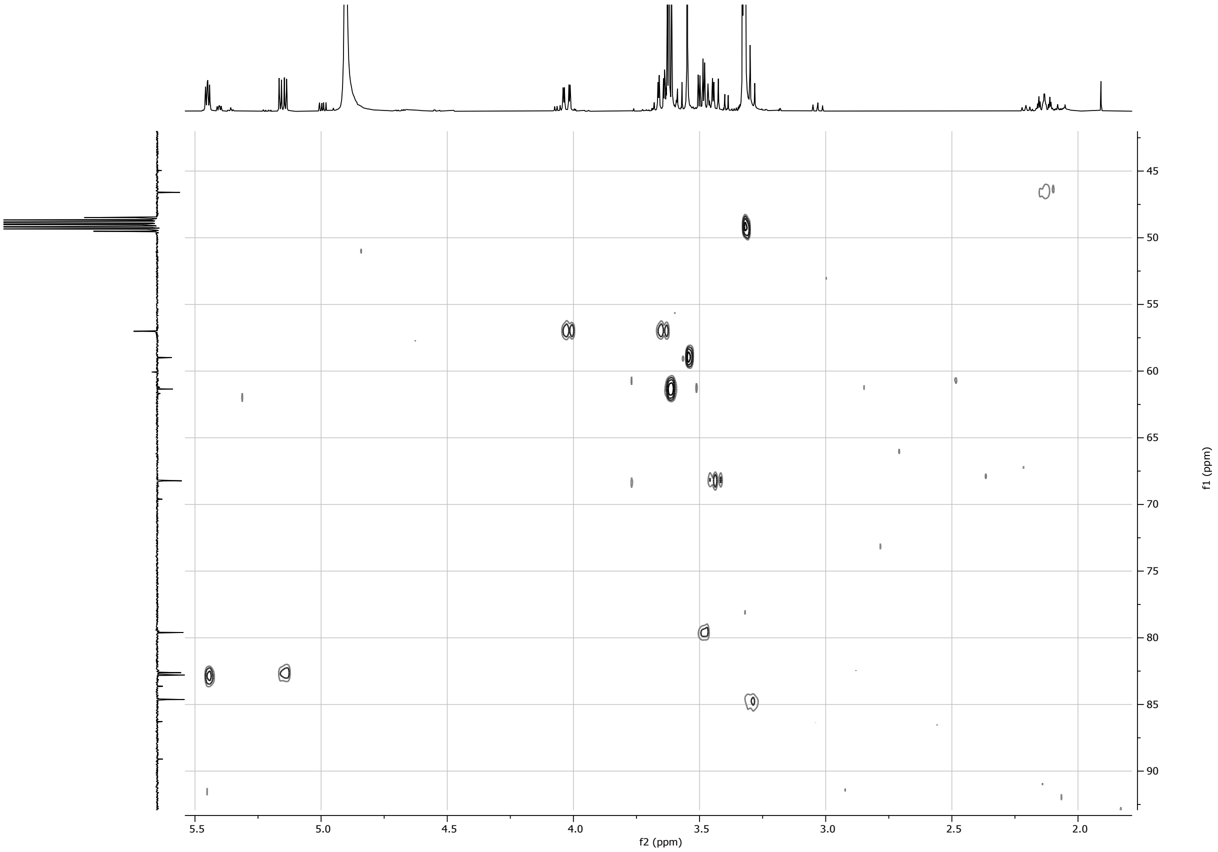

^{1}H NMR, 500MHz, $CDCl_3$ of **46**

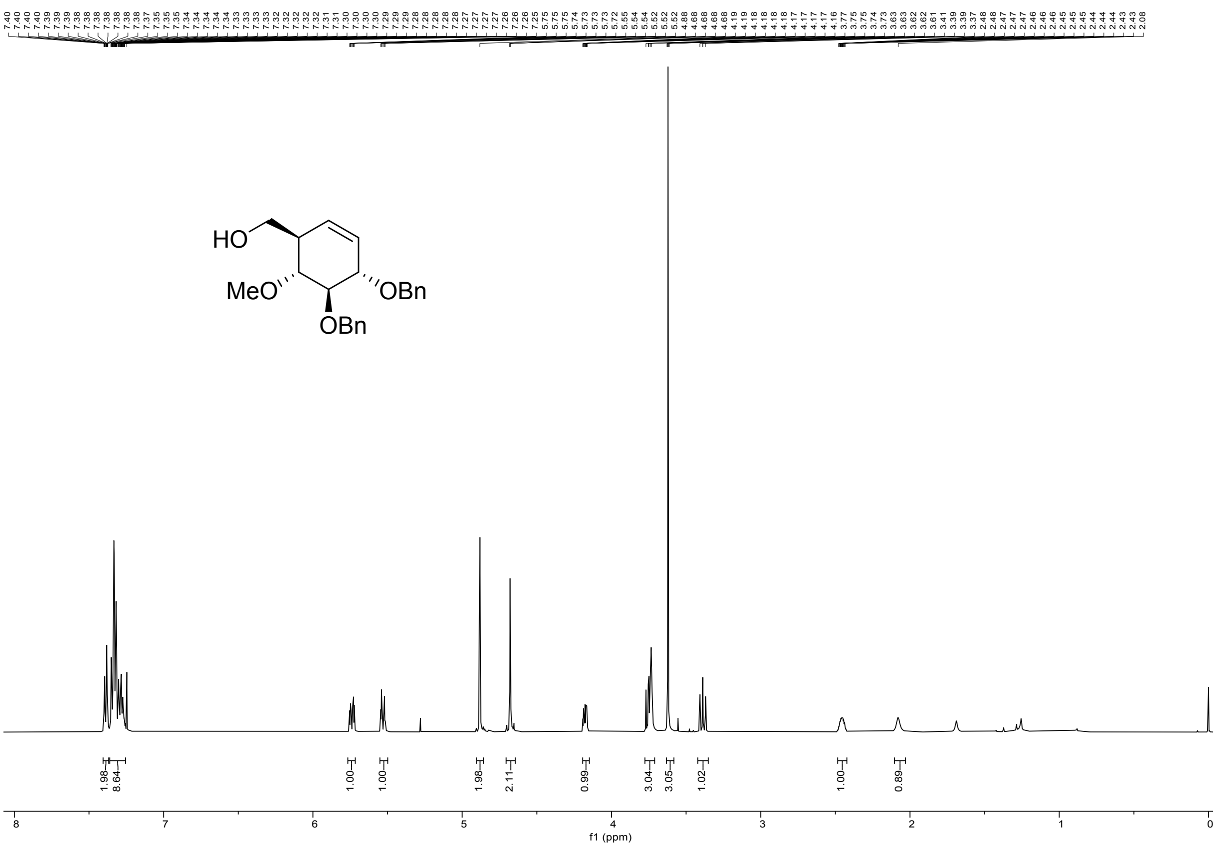

^{13}C NMR, 126MHz, $CDCl_3$ of **46**

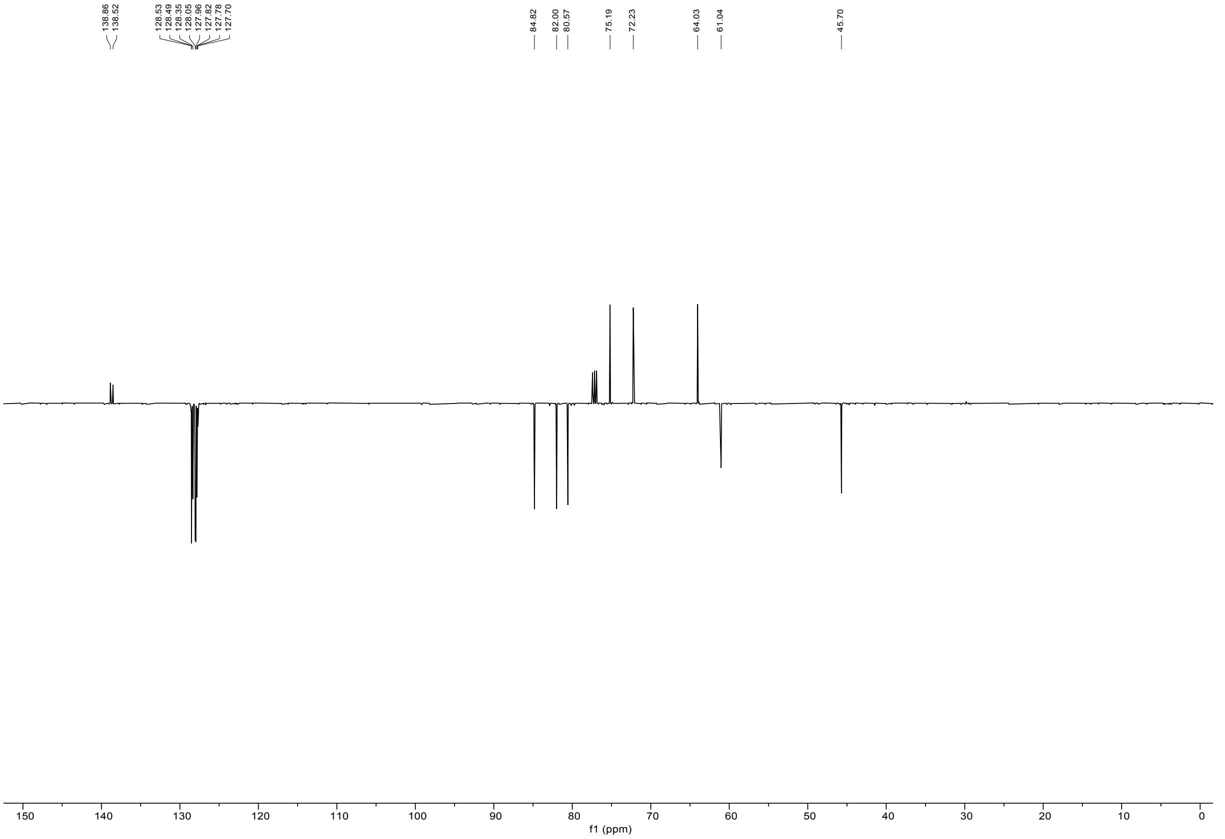

HH-COSY NMR, $CDCl_3$ of **46**

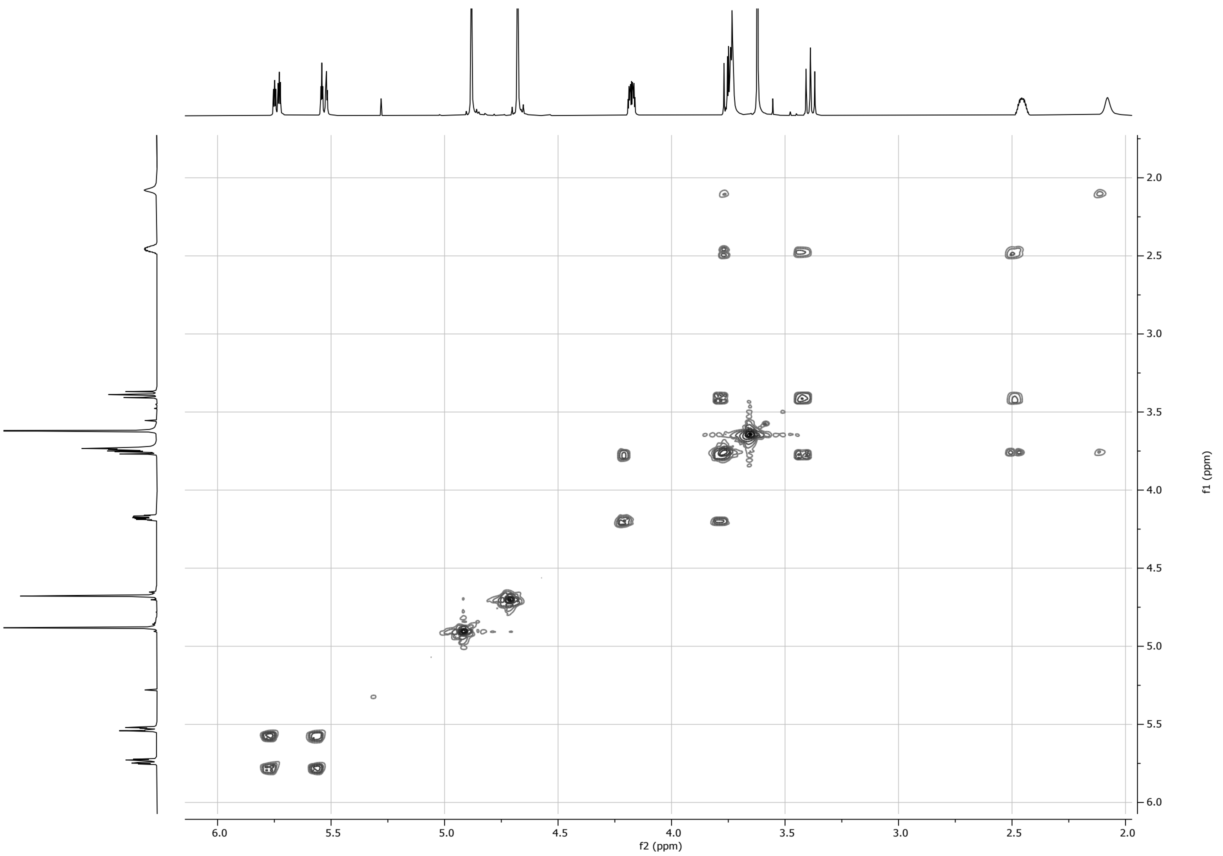

HSQC NMR, $CDCl_3$ of **46**

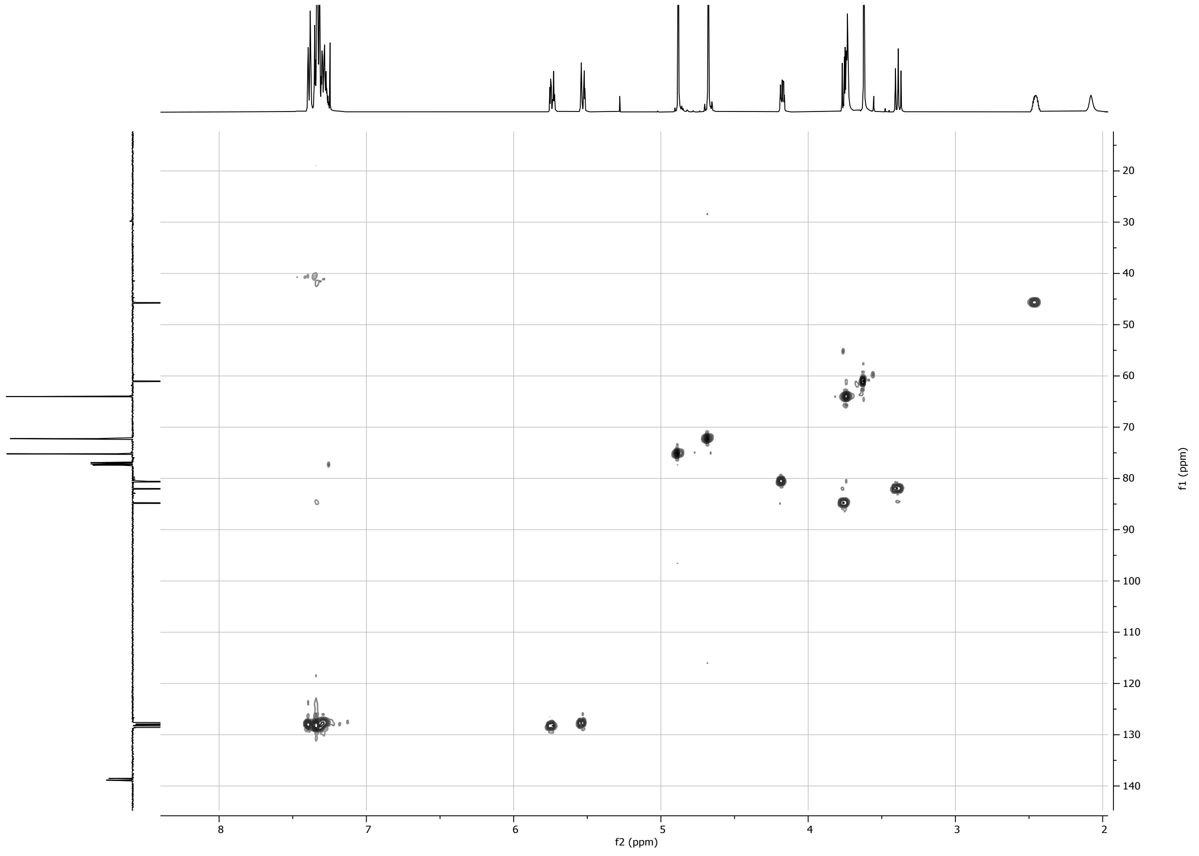

^{1}H NMR, 500MHz, $CDCl_3$ of **47**

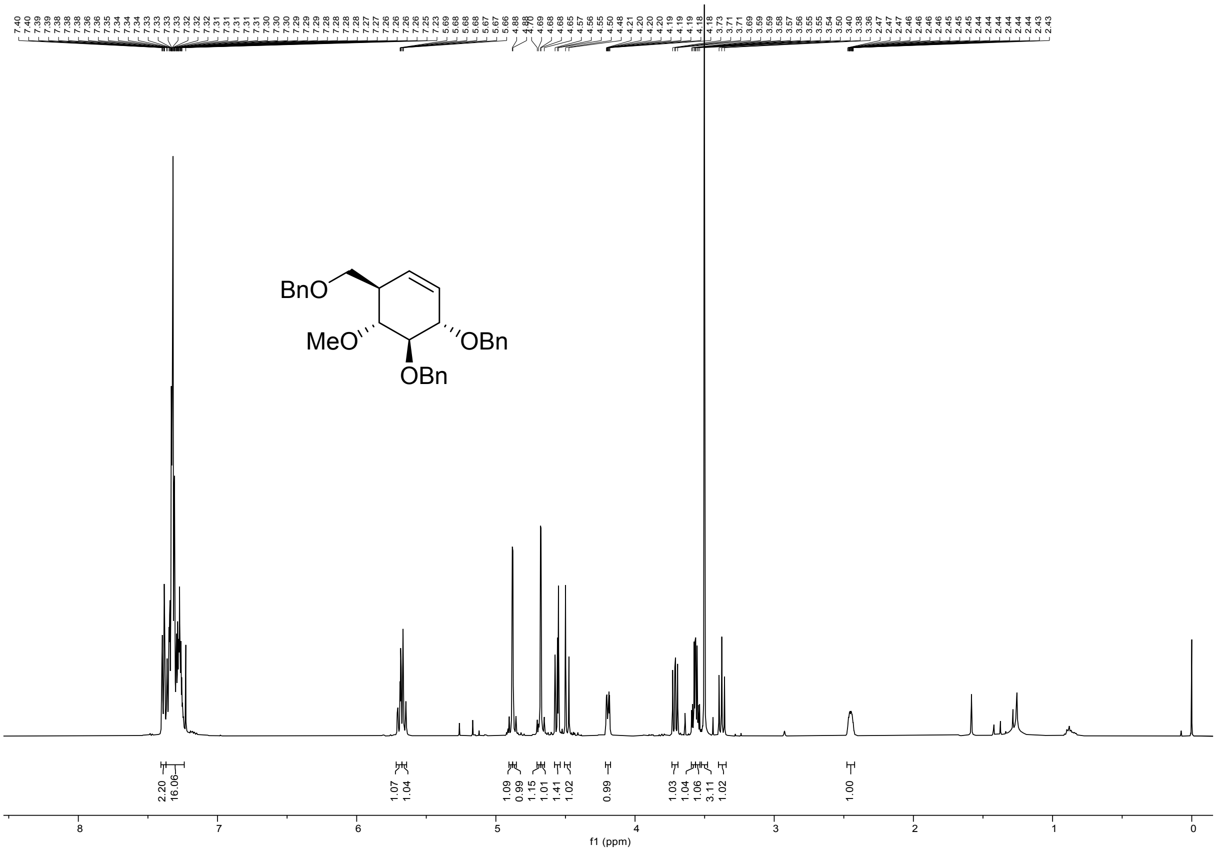

^{13}C NMR, 126MHz, $CDCl_3$ of **47**

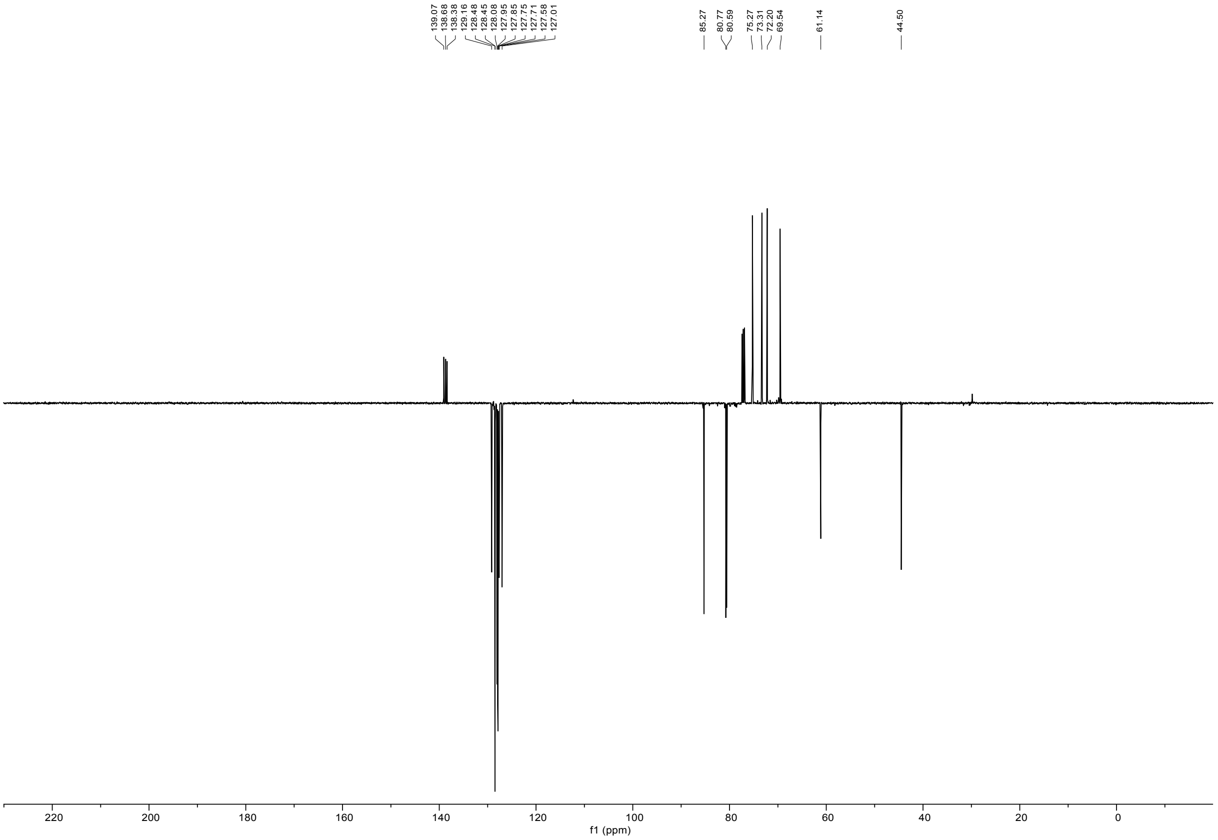

HH-COSY NMR, $CDCl_3$ of **47**

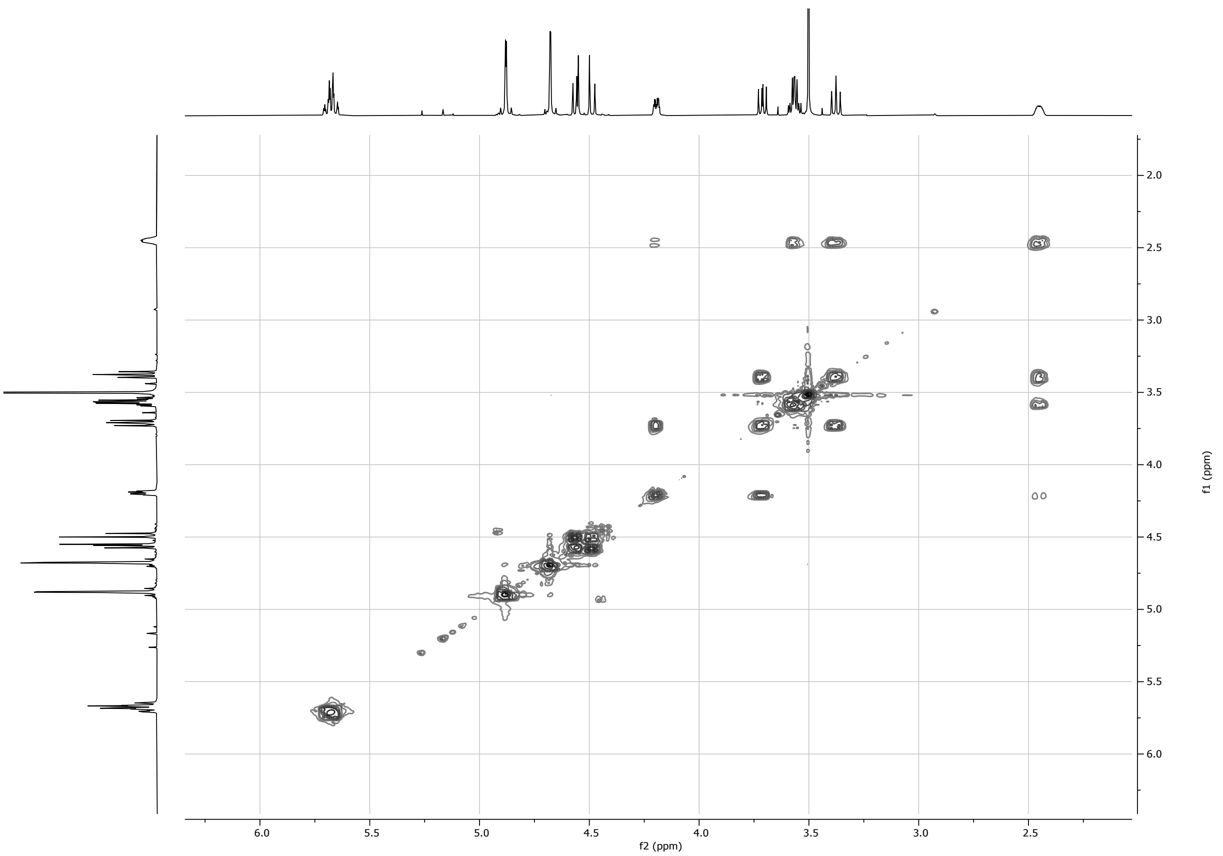

HSQC NMR, $CDCl_3$ of **47**

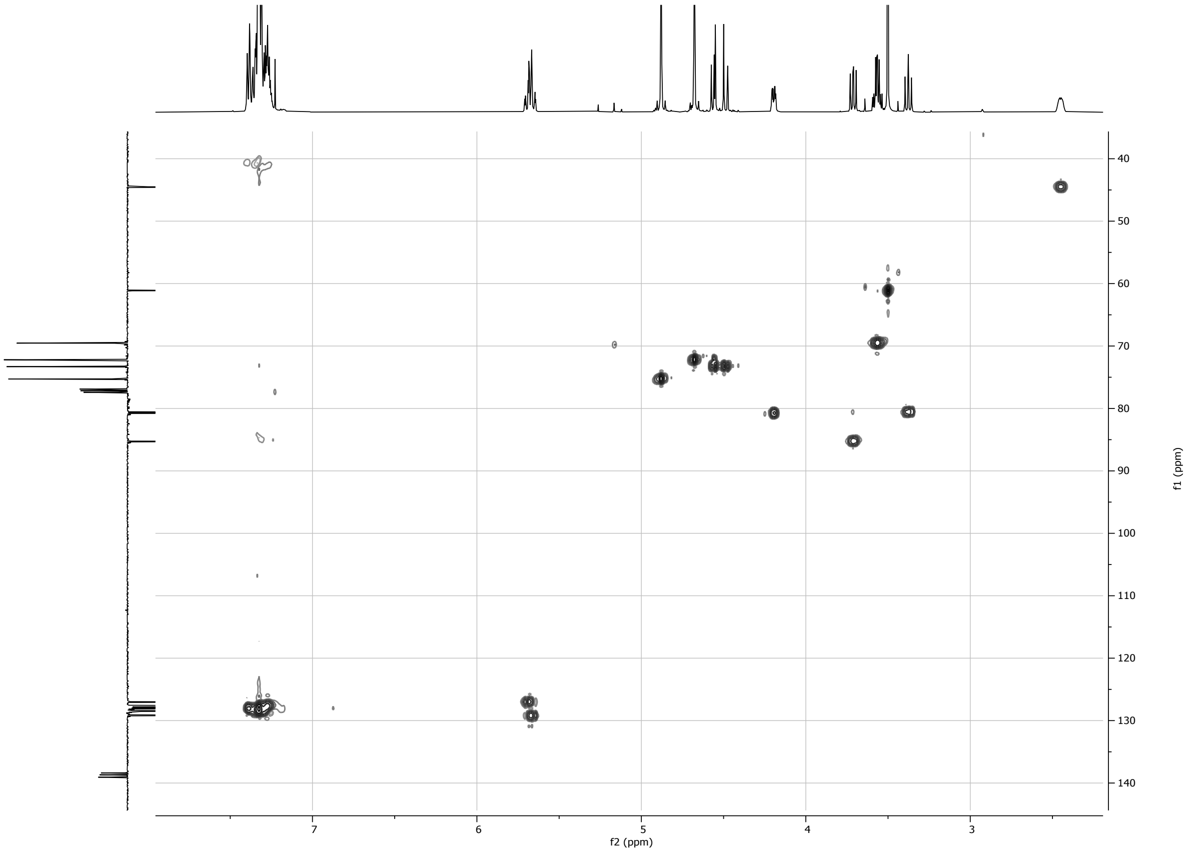

^{1}H NMR, 500MHz, $CDCl_3$ of **50**

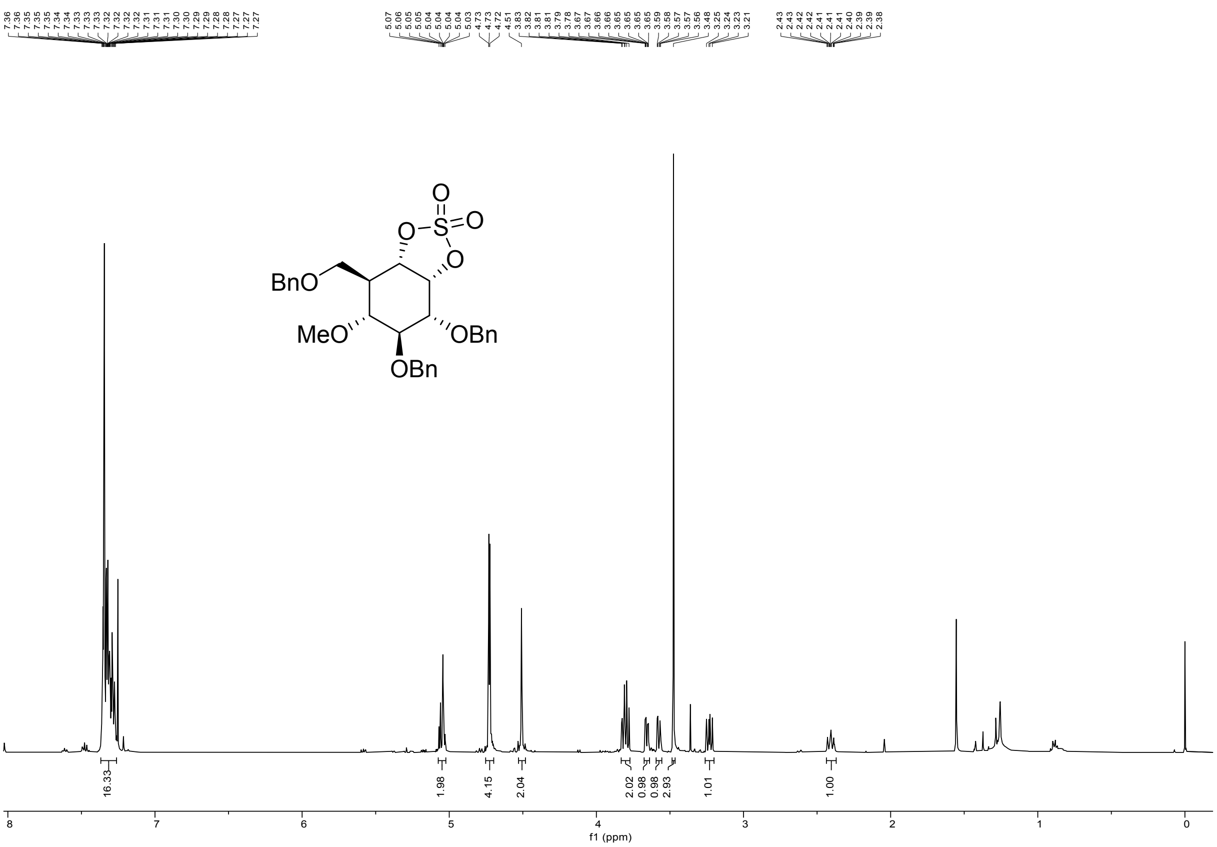

^{13}C NMR, 126MHz, $CDCl_3$ of **50**

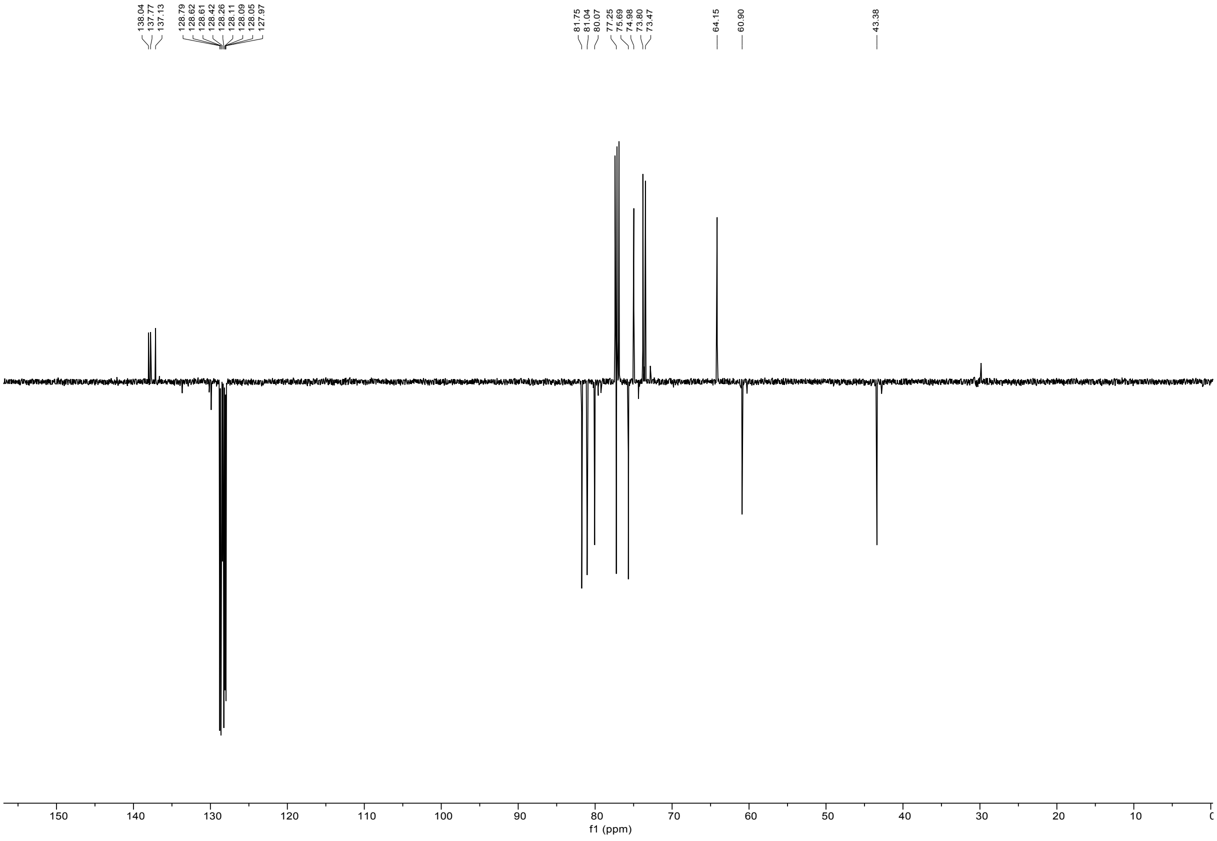

HH-COSY NMR, $CDCl_3$ of **50**

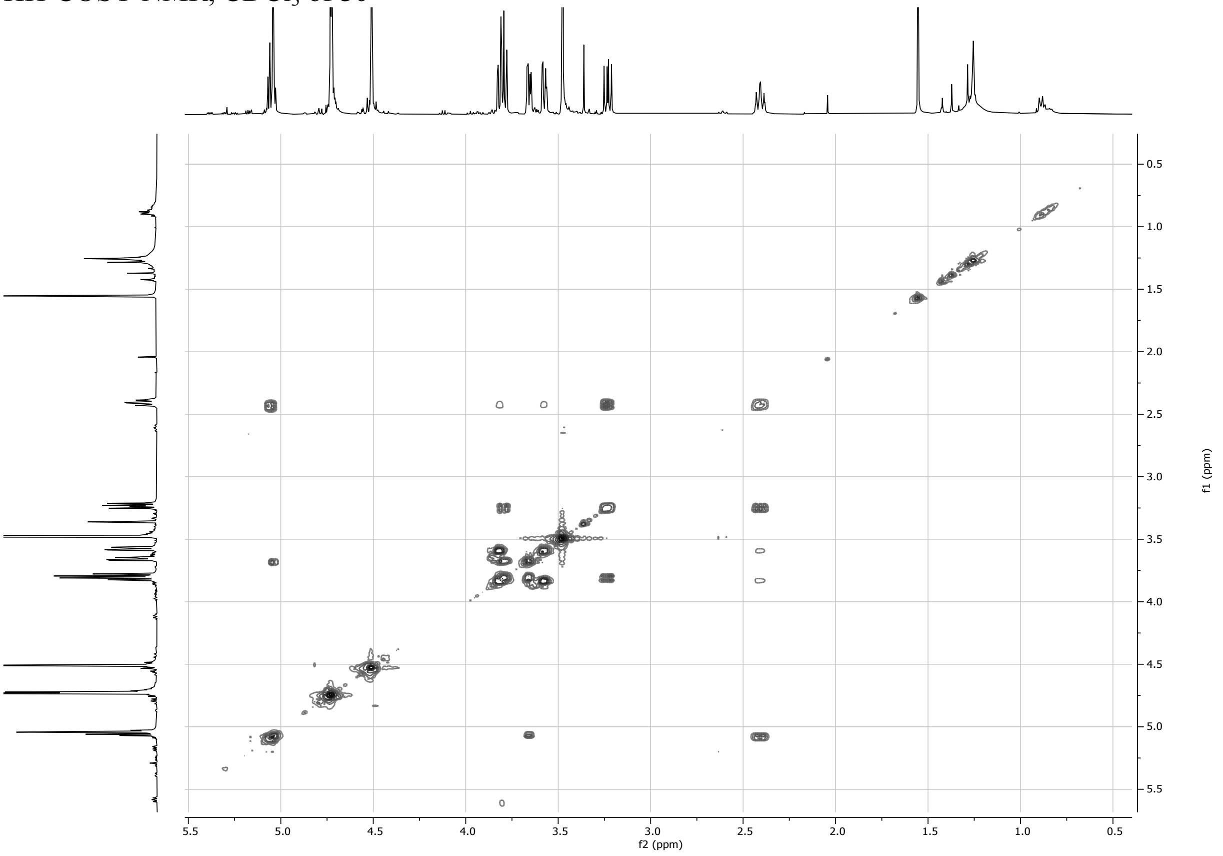

HSQC NMR, $CDCl_3$ of **50**

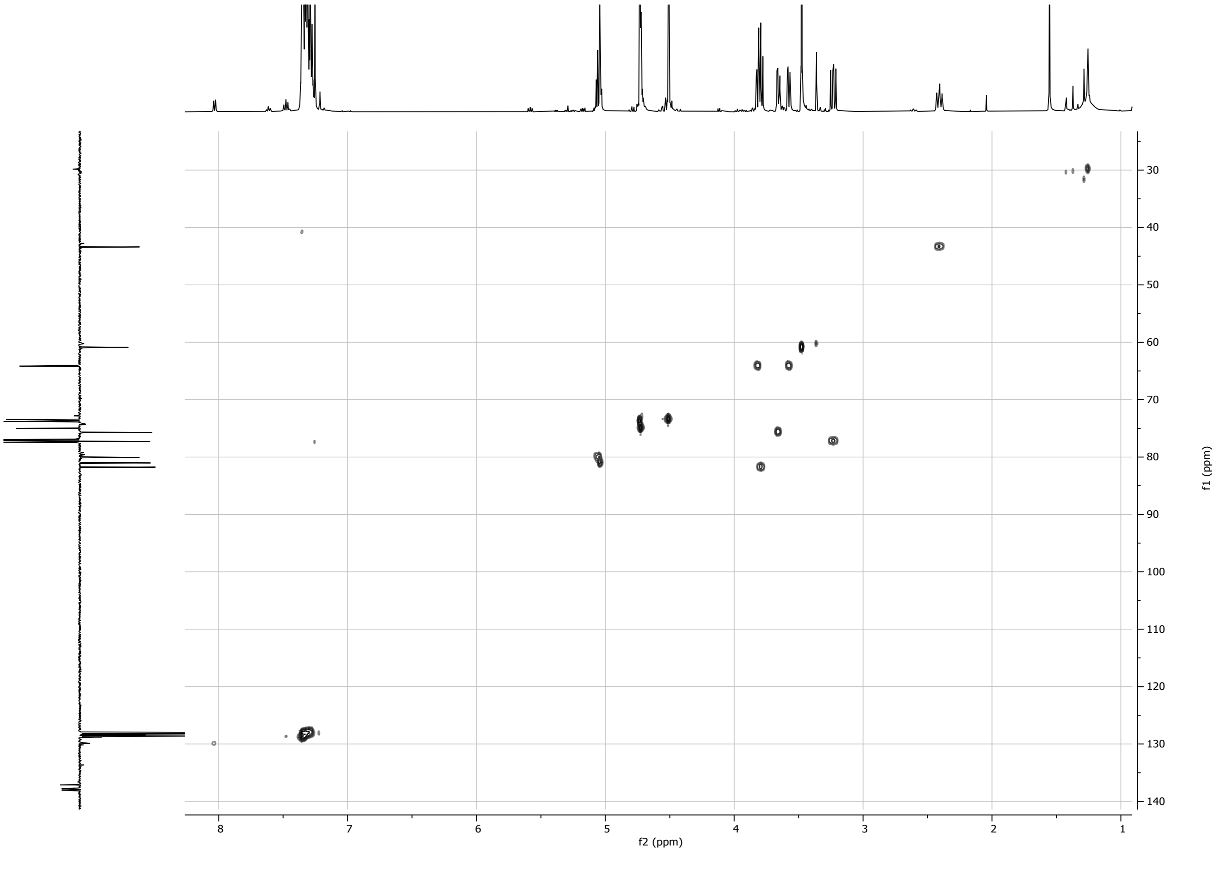

^{1}H NMR, 500MHz, MeOD of **7**

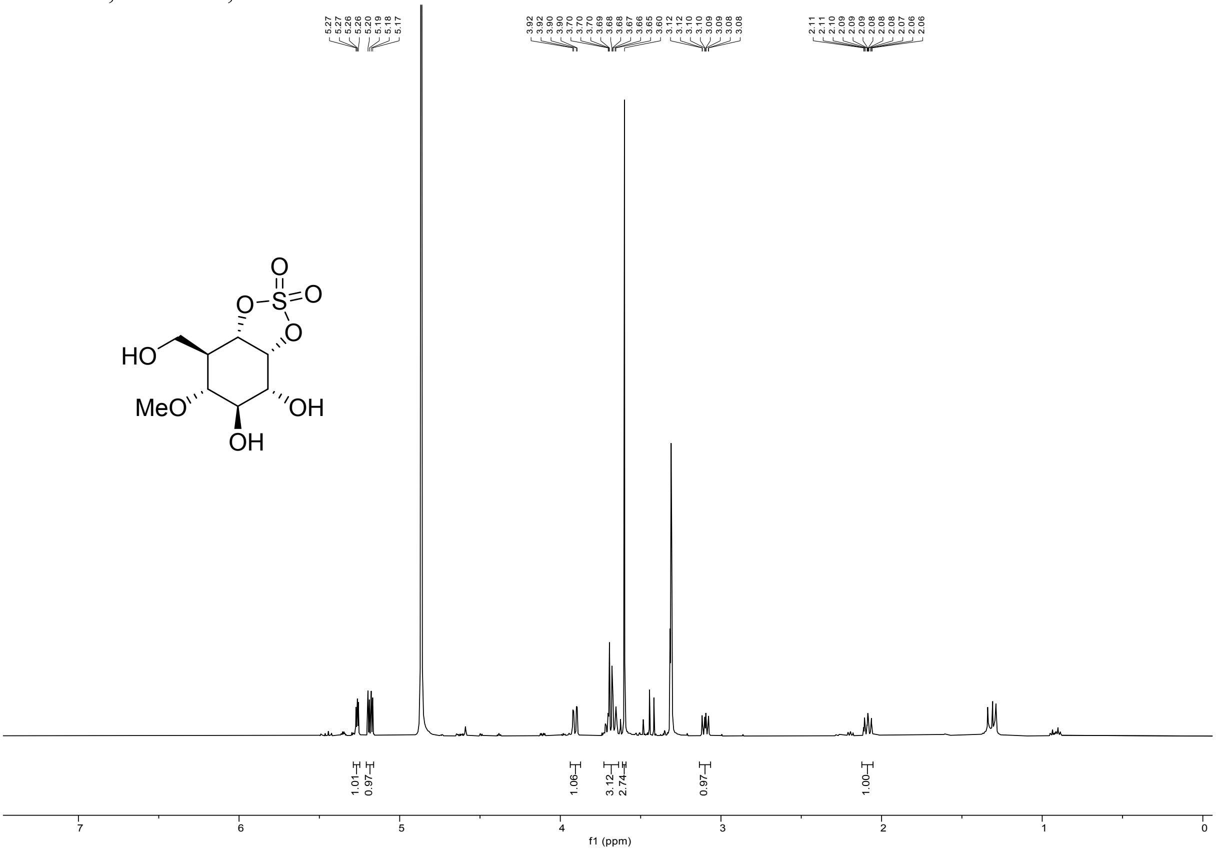

^{13}C NMR, 126MHz, MeOD of **7**

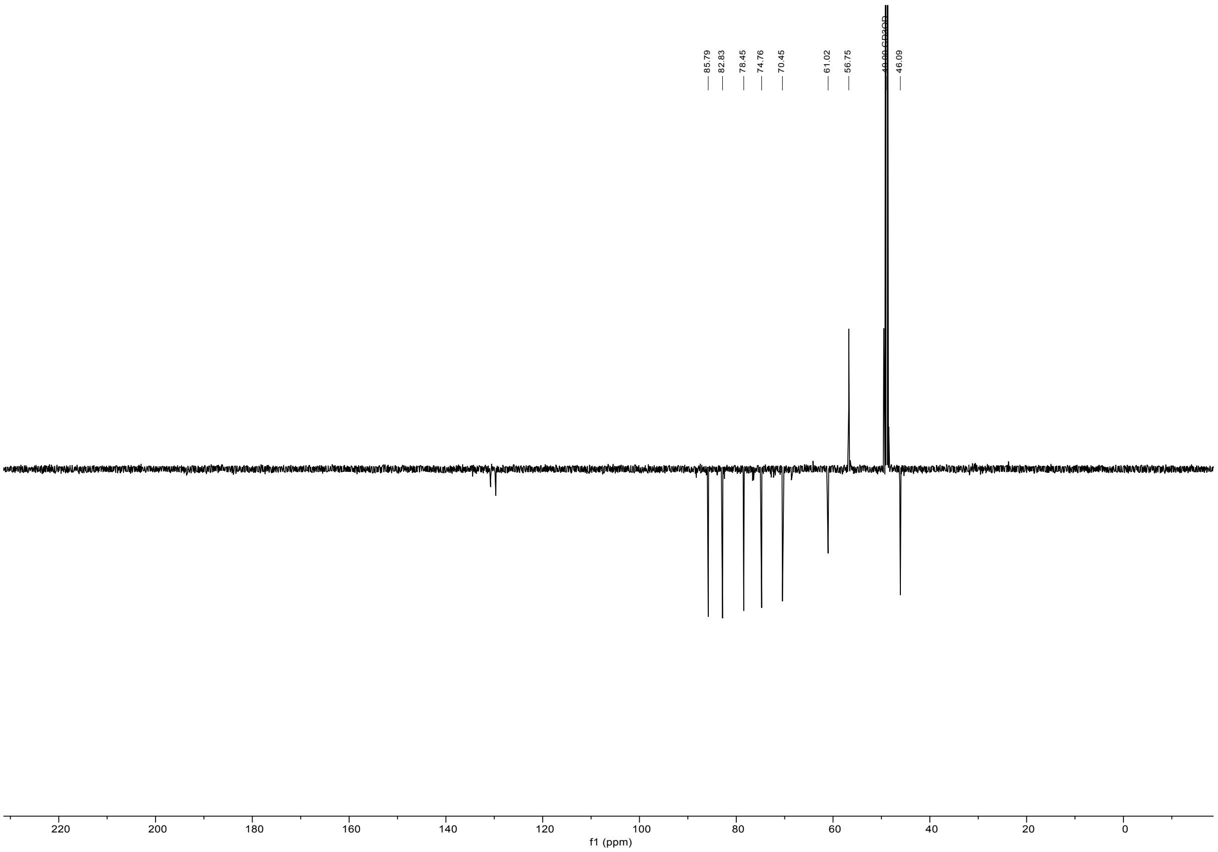

HH-COSY NMR, MeOD of **7**

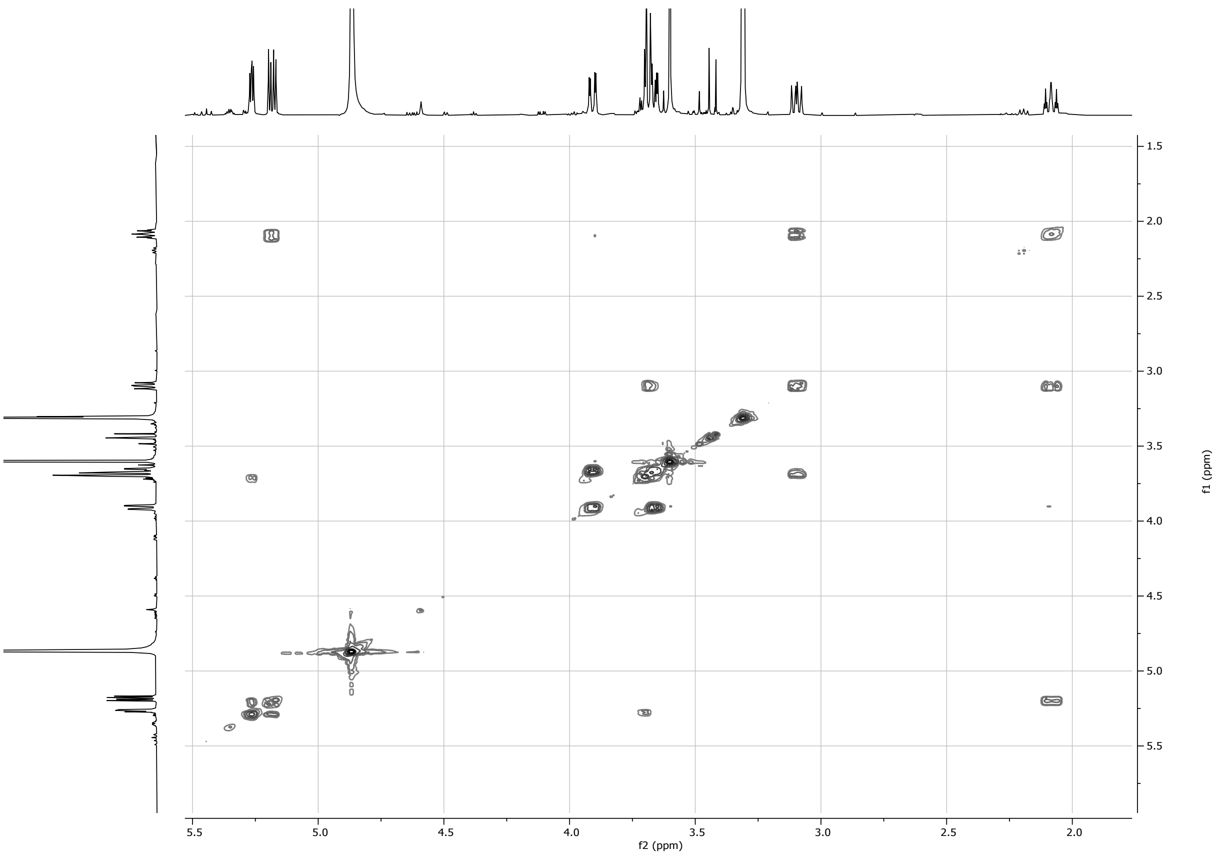

HSQC NMR, MeOD of **7**

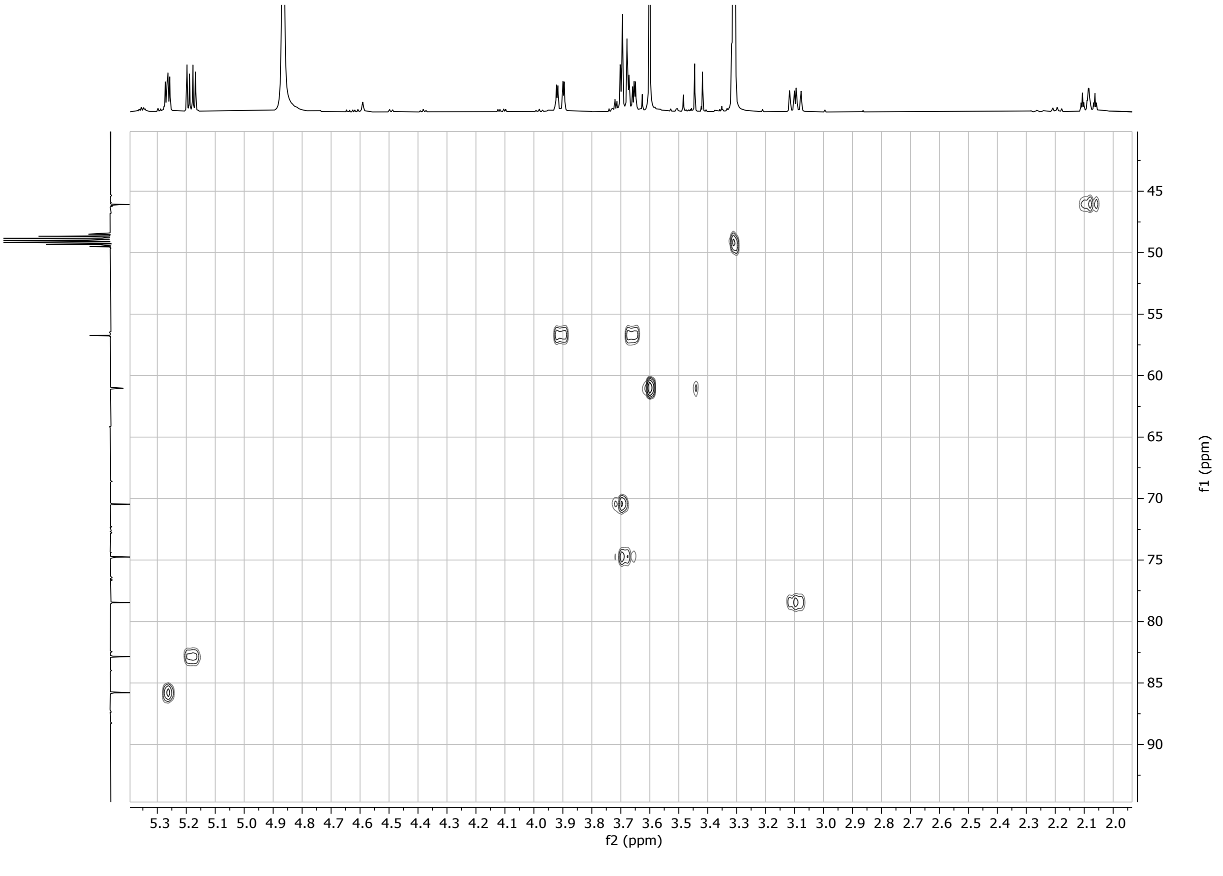

^{1}H NMR, 500MHz, $CDCl_3$ of **51**

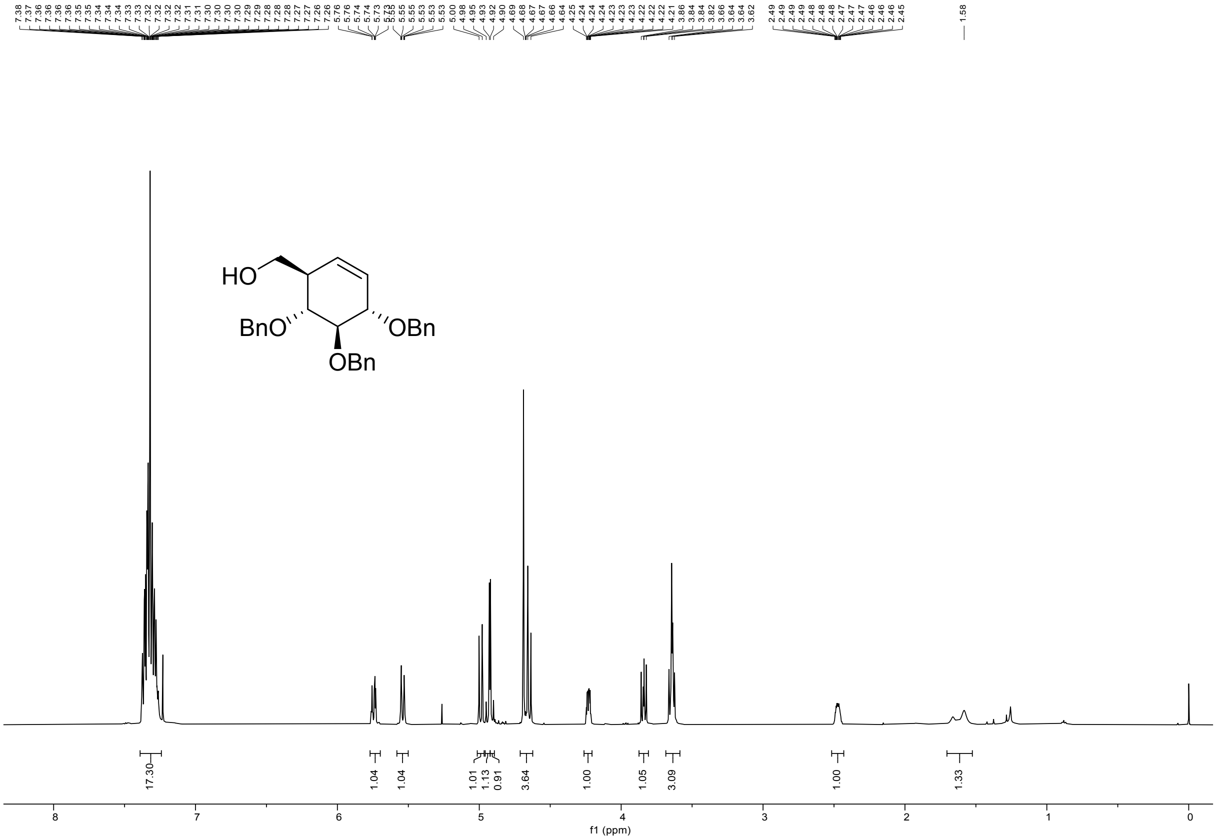

^{13}C NMR, 126MHz, $CDCl_3$ of **51**

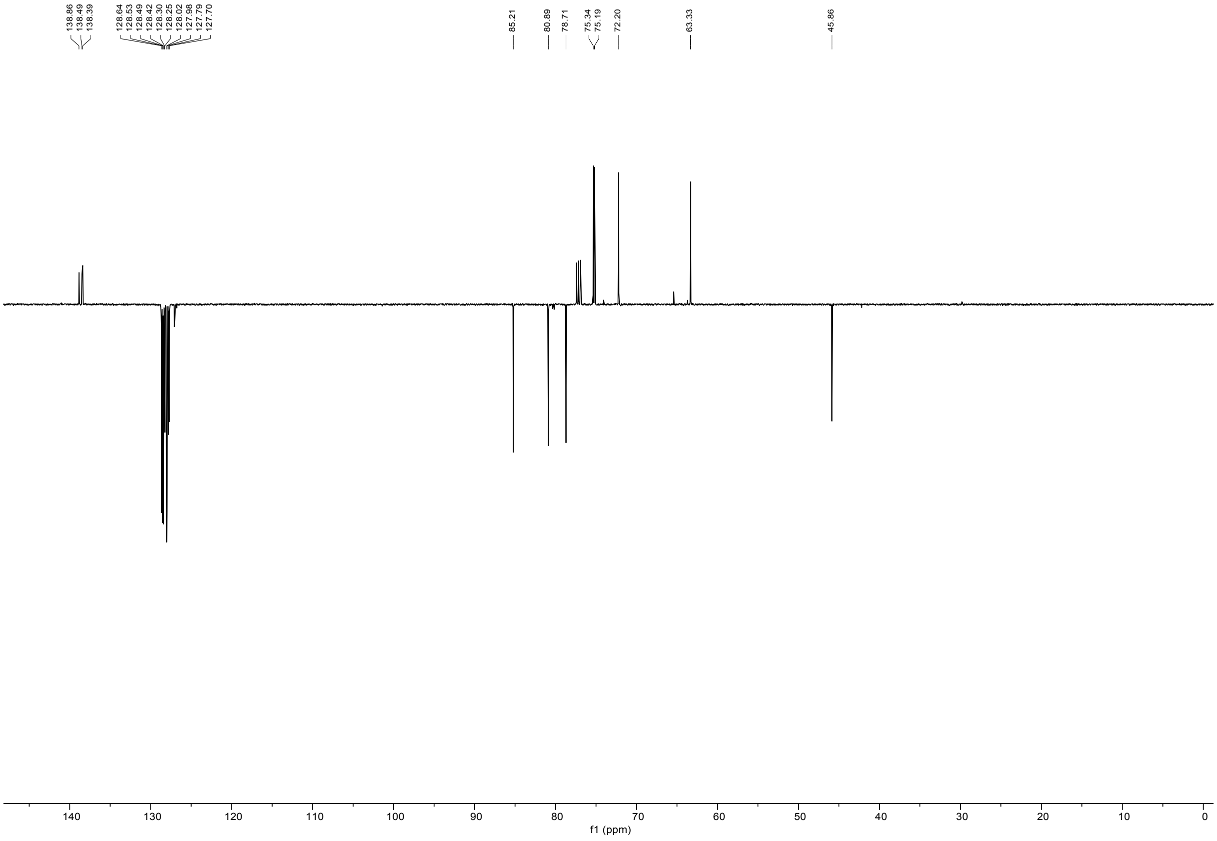

HH-COSY NMR, $CDCl_3$ of **51**

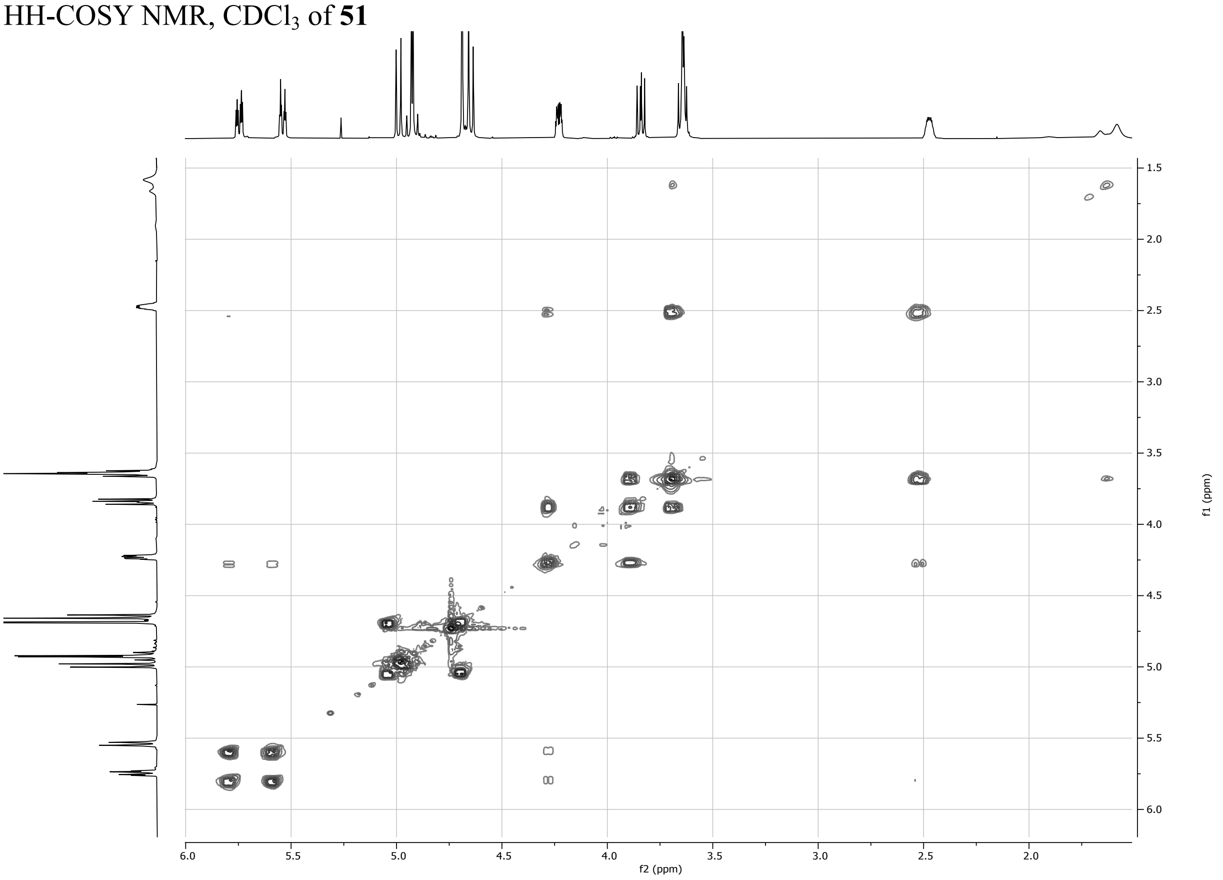

HSQC NMR, $CDCl_3$ of **51**

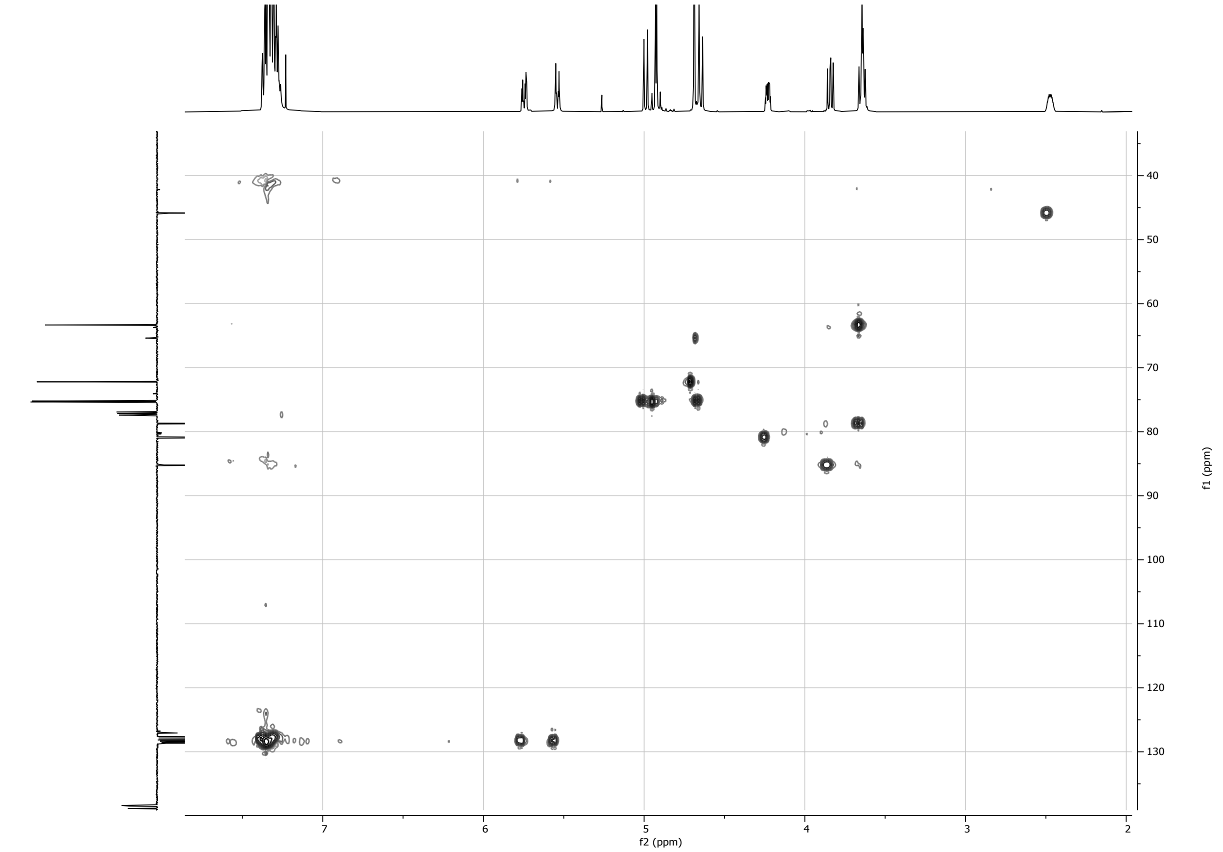

^{1}H NMR, 500MHz, $CDCl_3$ of **52**

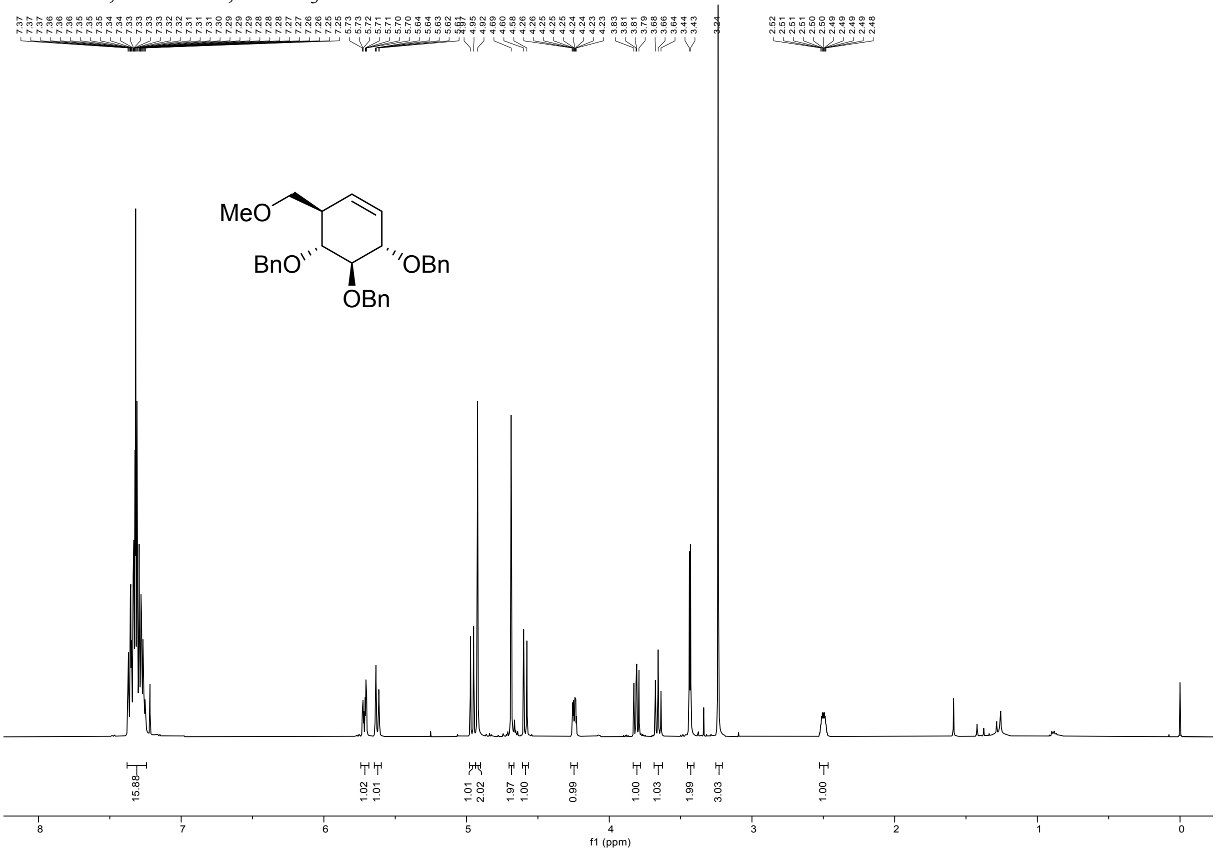

^{13}C NMR, 126MHz, $CDCl_3$ of **52**

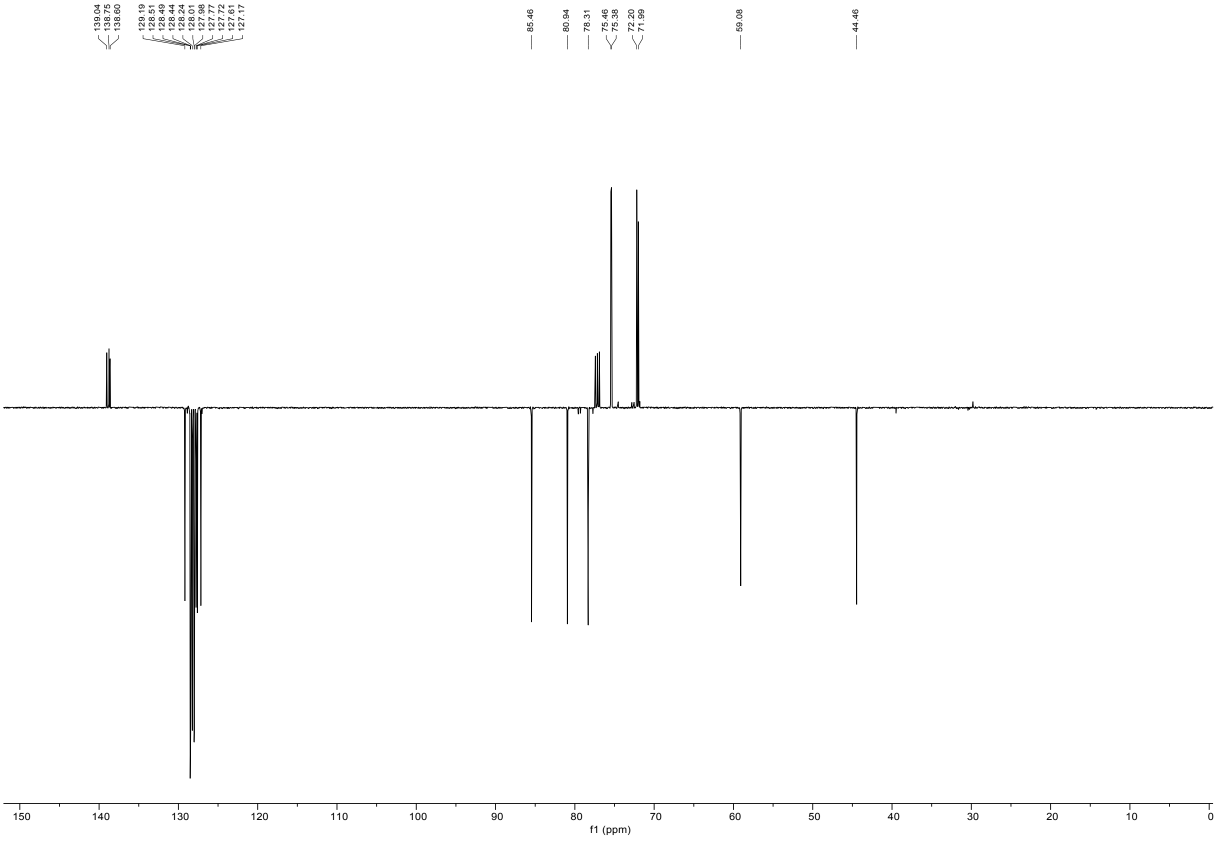

HH-COSY NMR, $CDCl_3$ of **52**

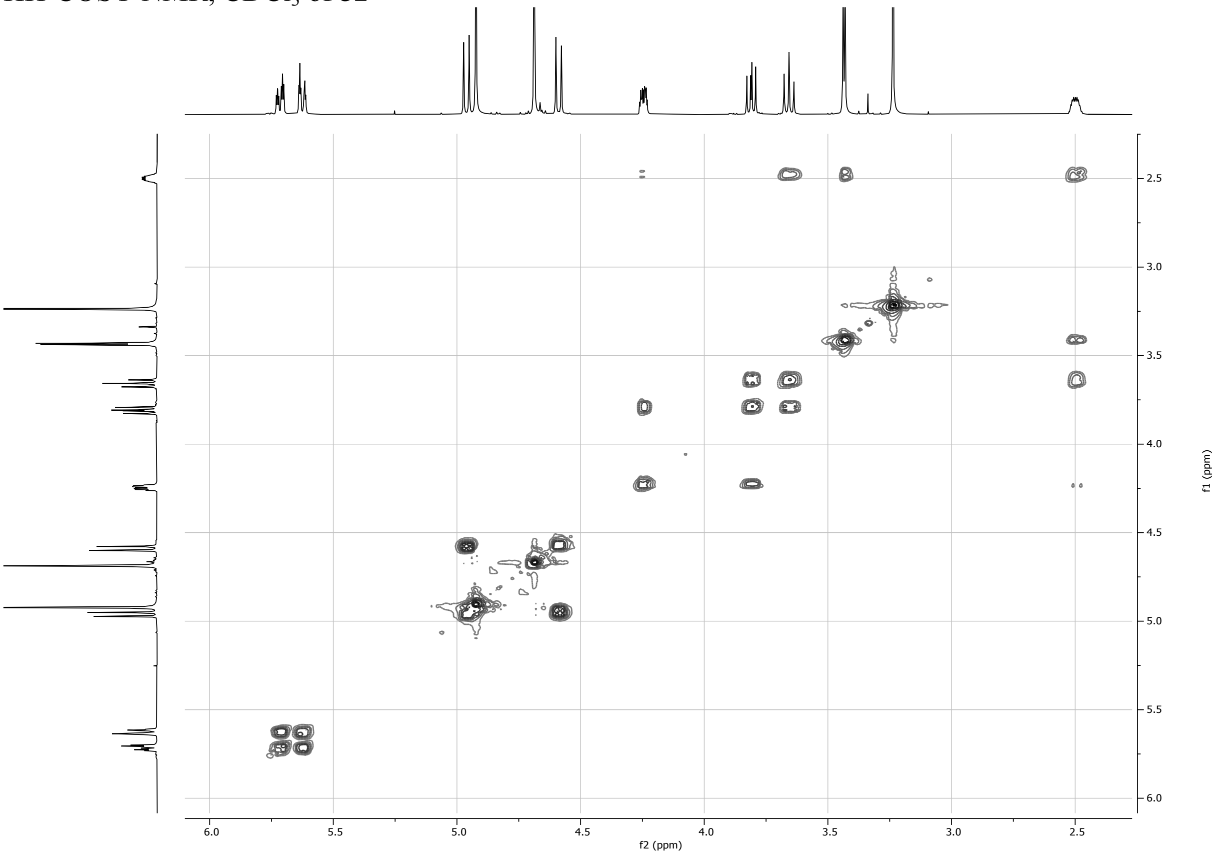

HSQC NMR, $CDCl_3$ of **52**

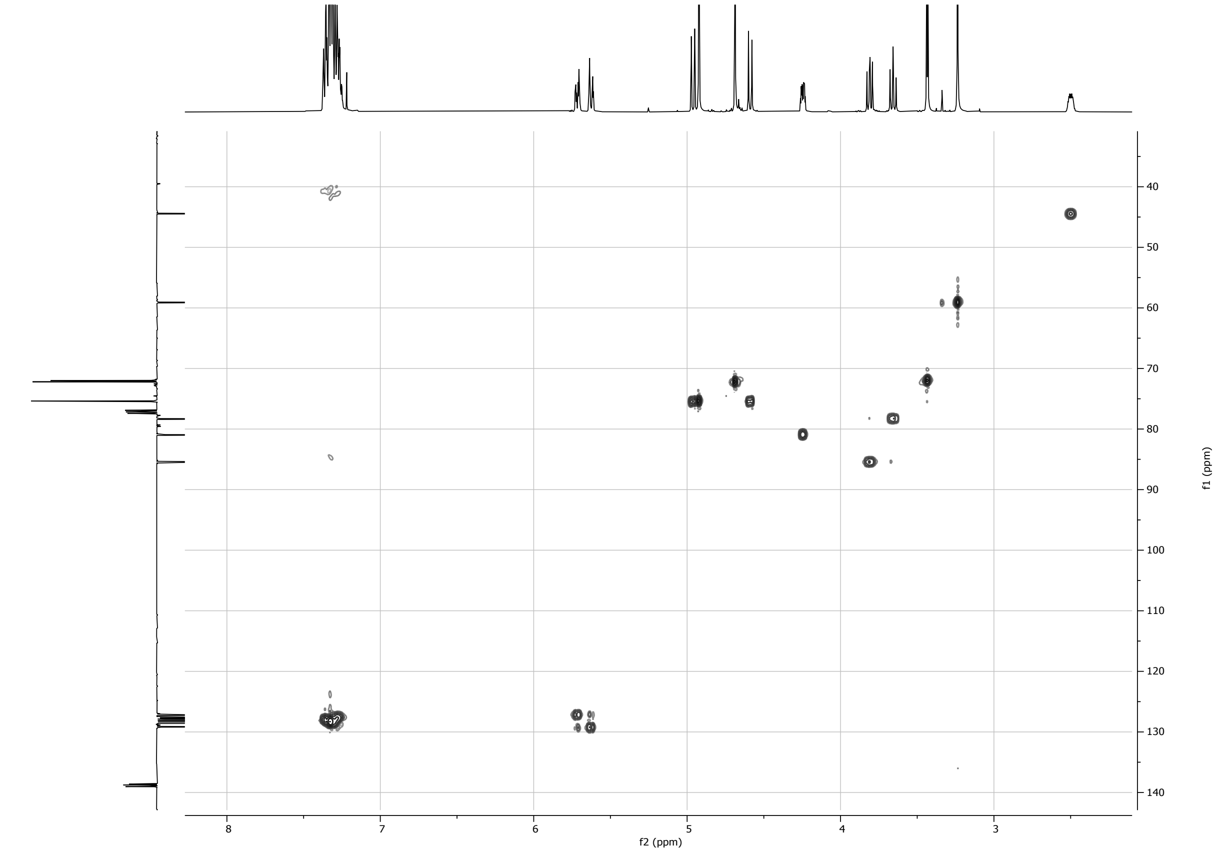

^{1}H NMR, 400MHz, $CDCl_3$ of **53**

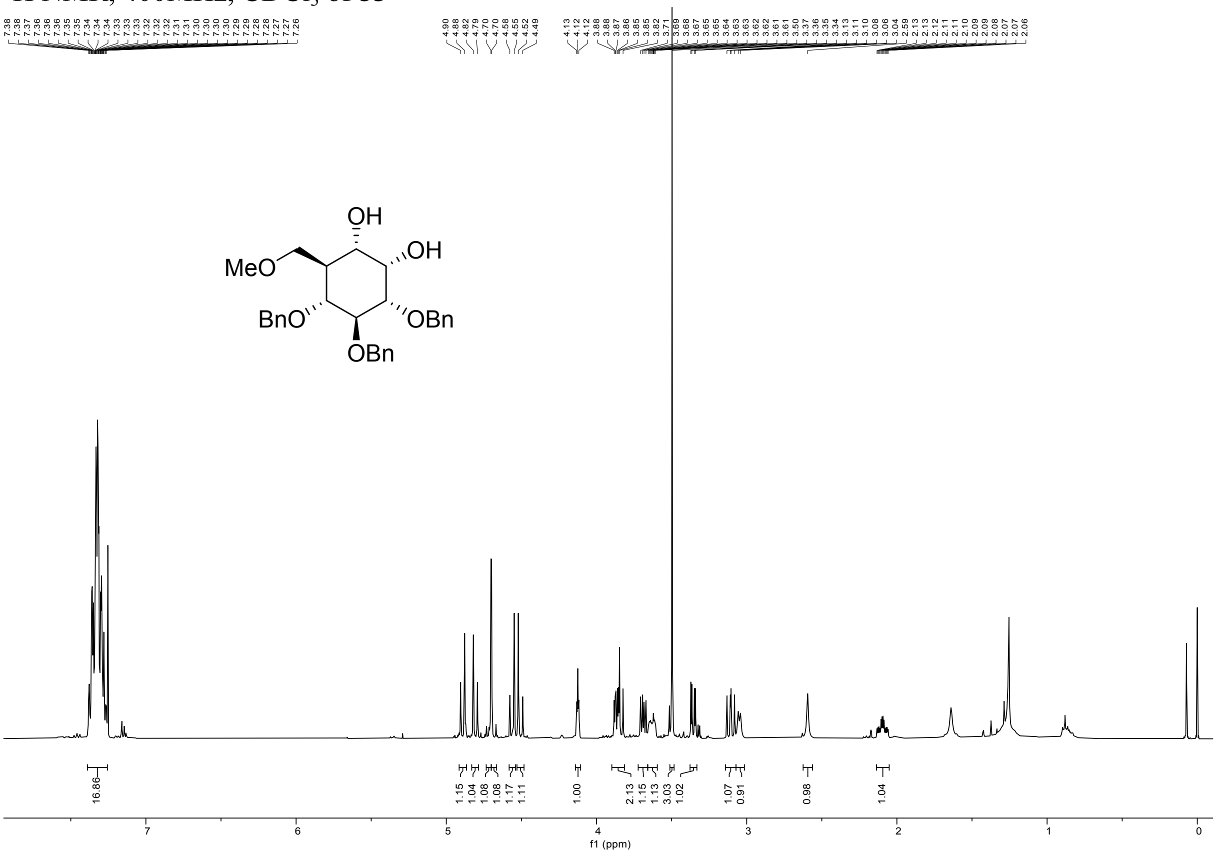

^{13}C NMR, 101MHz, $CDCl_3$ of **53**

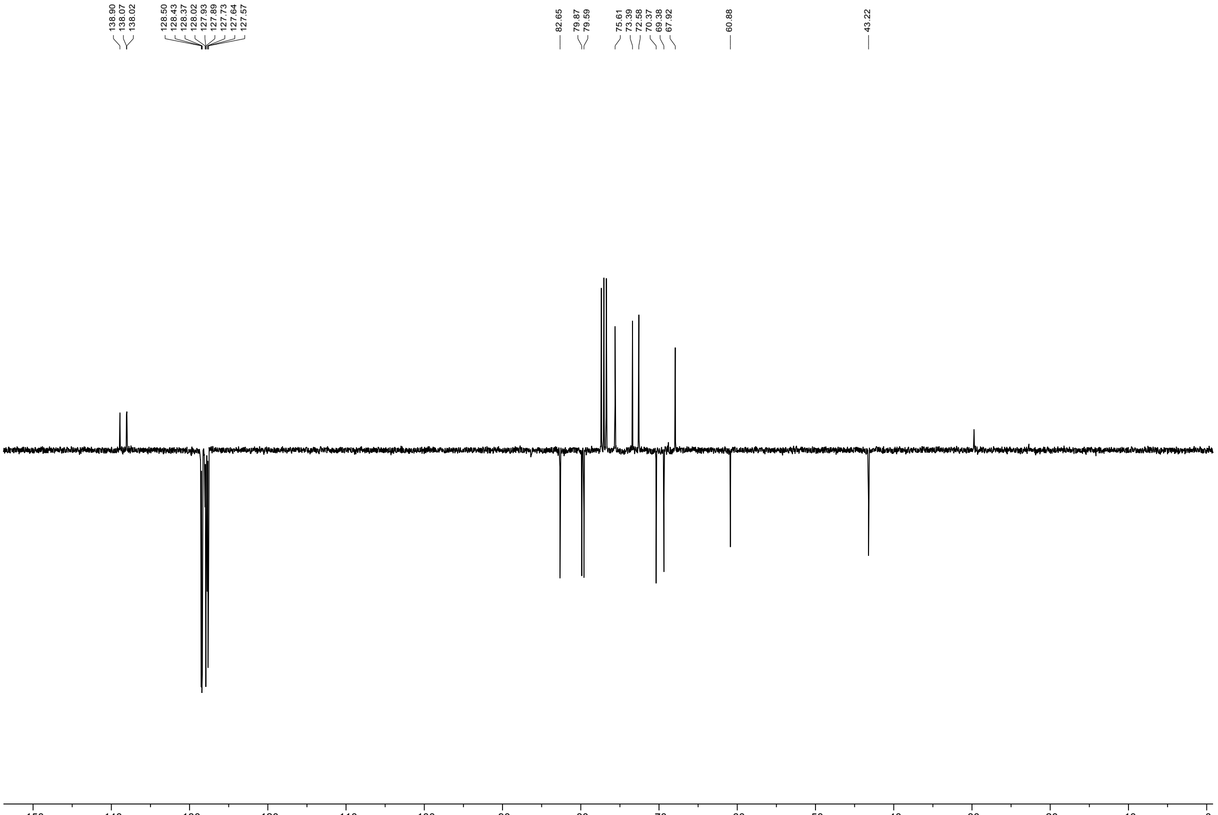

HH-COSY NMR, $CDCl_3$ of **53**

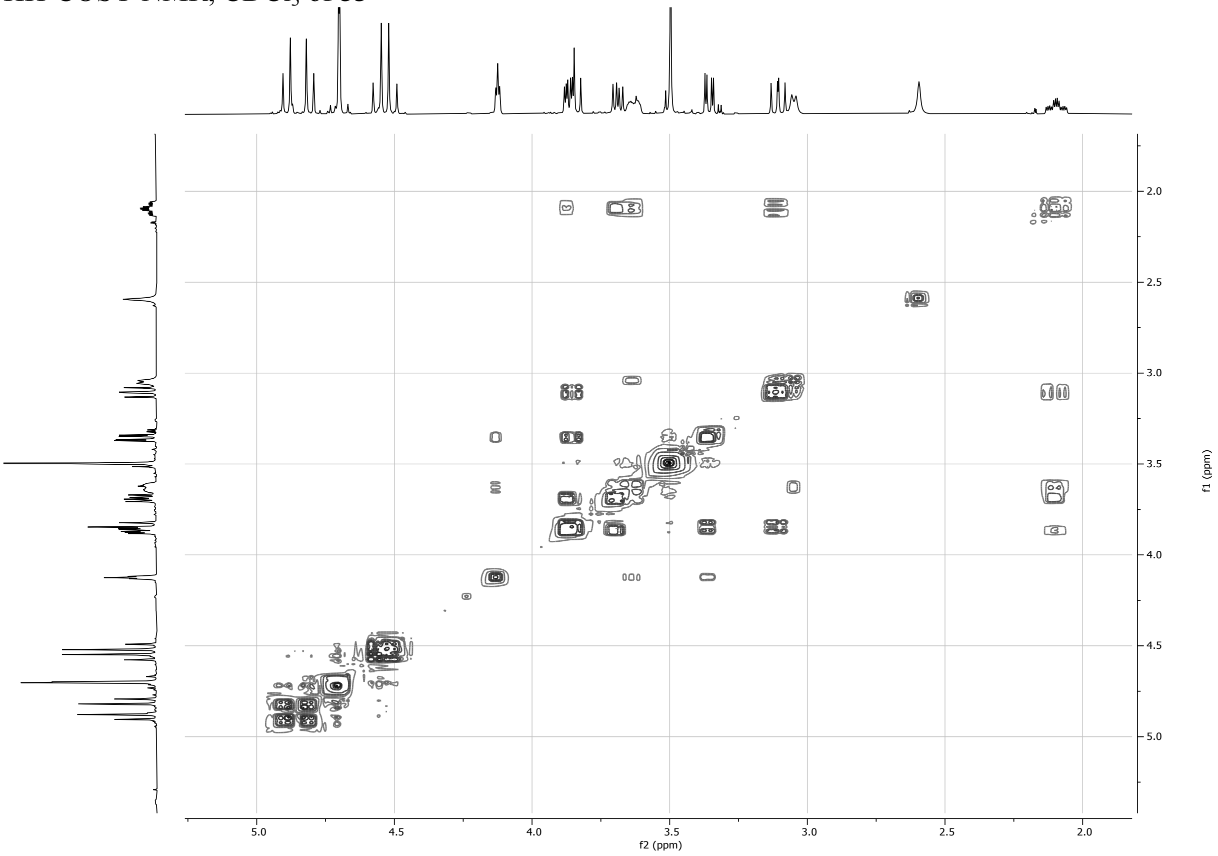

HSQC NMR, $CDCl_3$ of **53**

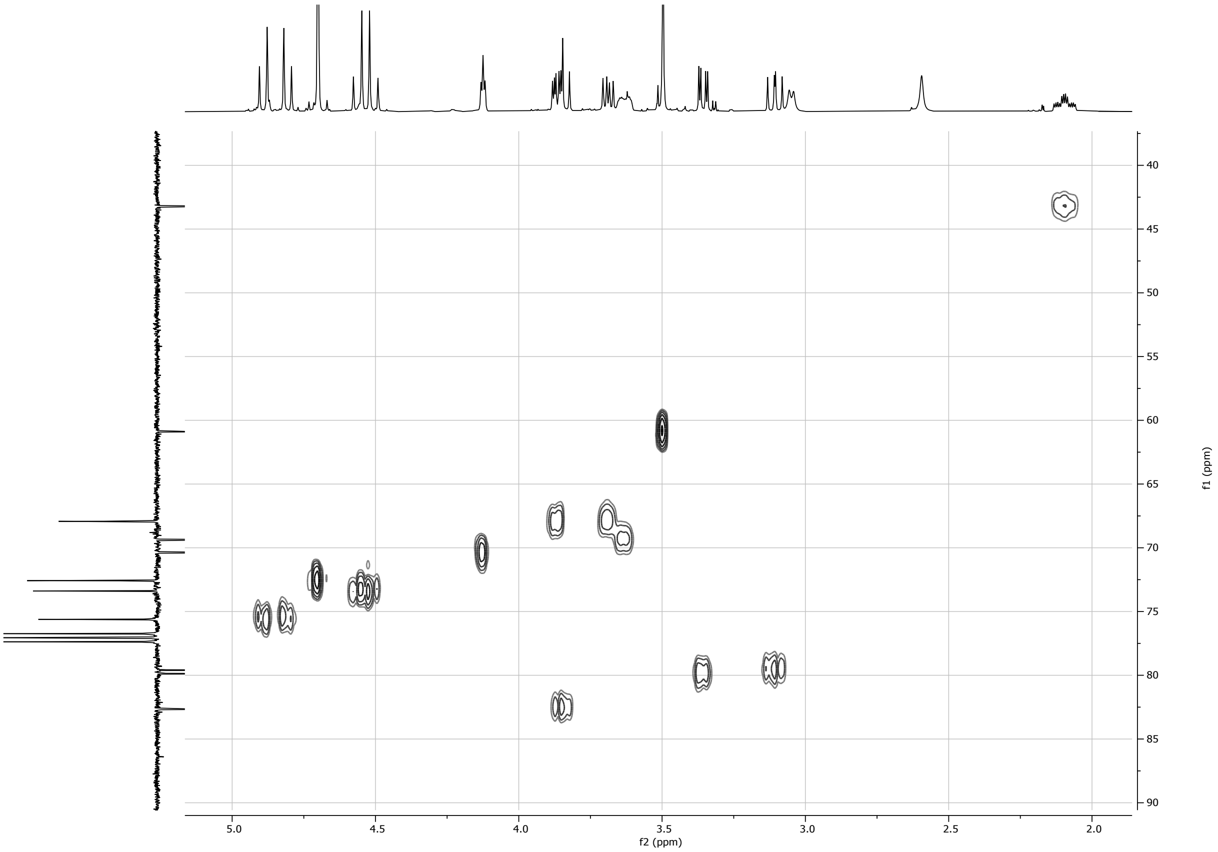

^{1}H NMR, 500MHz, $CDCl_3$ of **54**

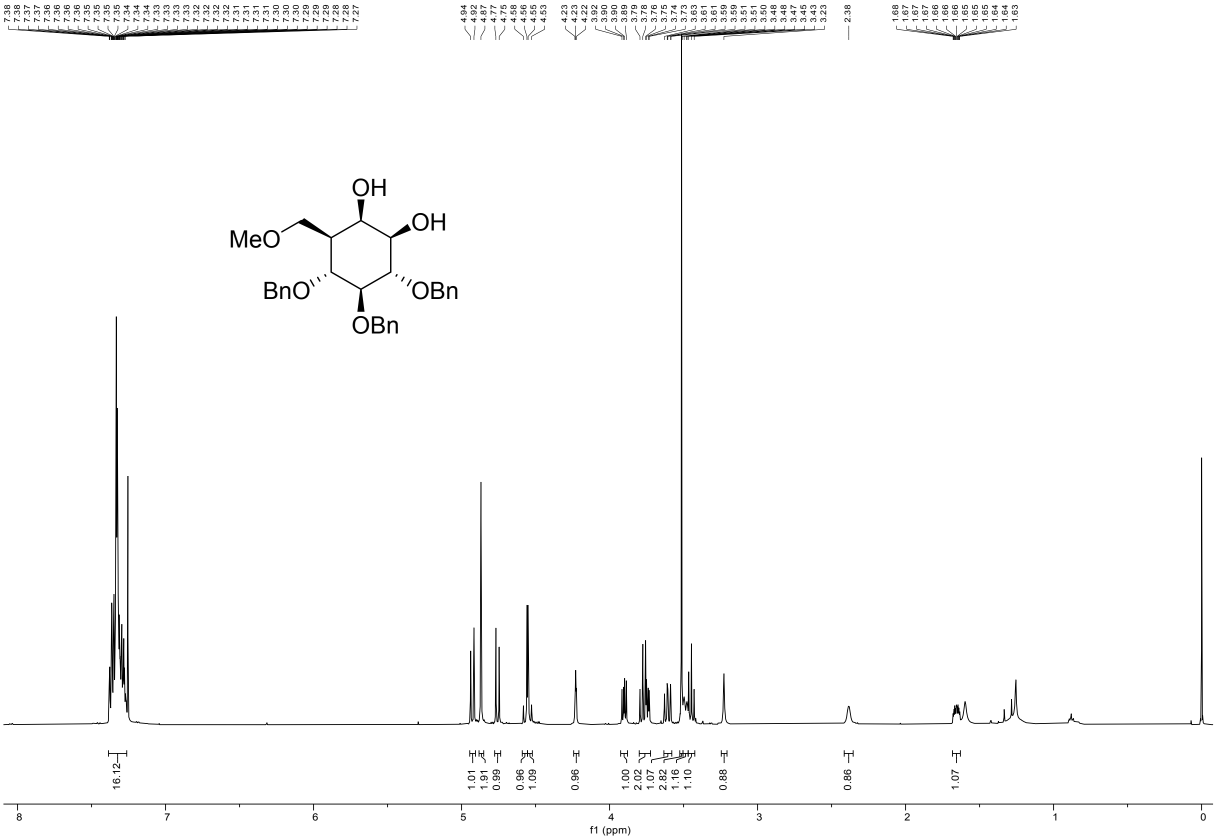

^{13}C NMR, 126MHz, $CDCl_3$ of **54**

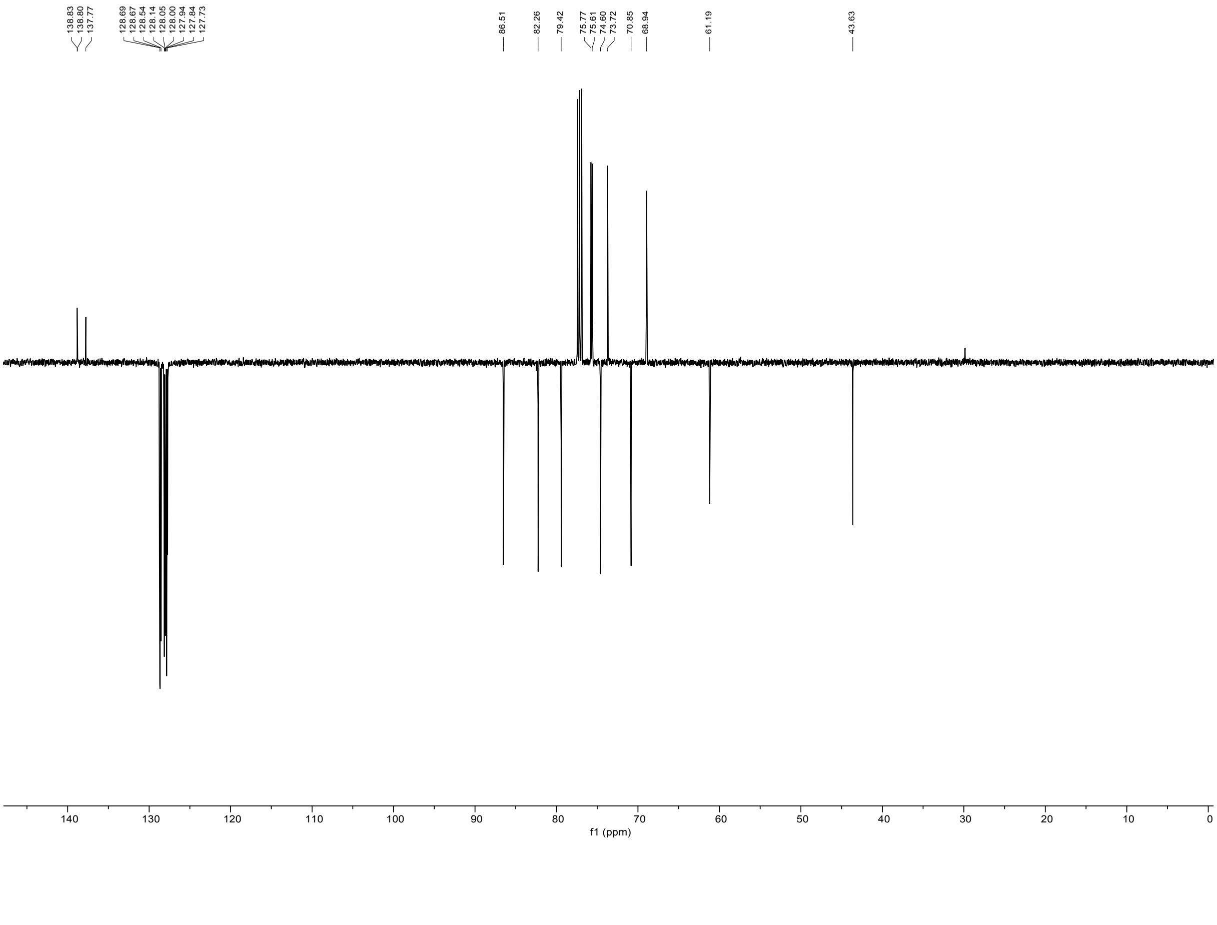

HH-COSY NMR, $CDCl_3$ of **54**

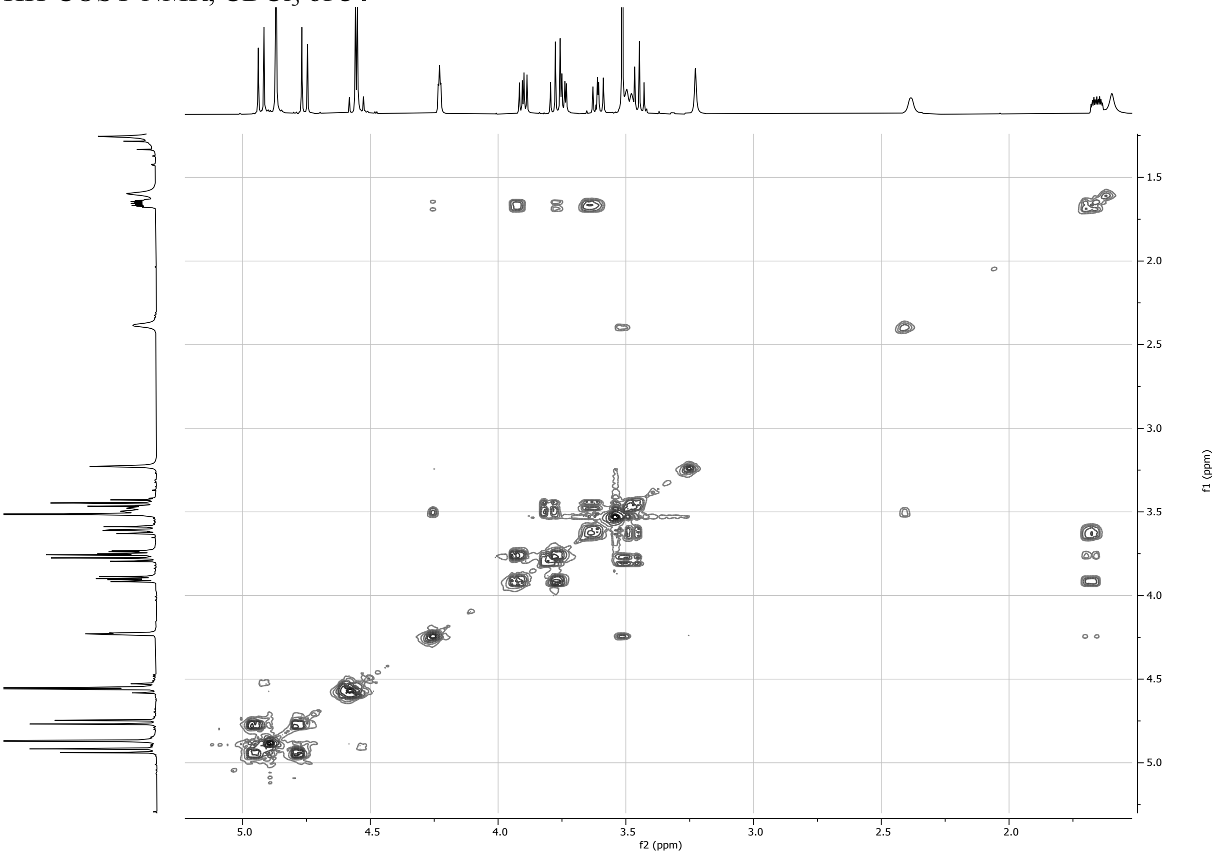

HSQC NMR, $CDCl_3$ of **54**

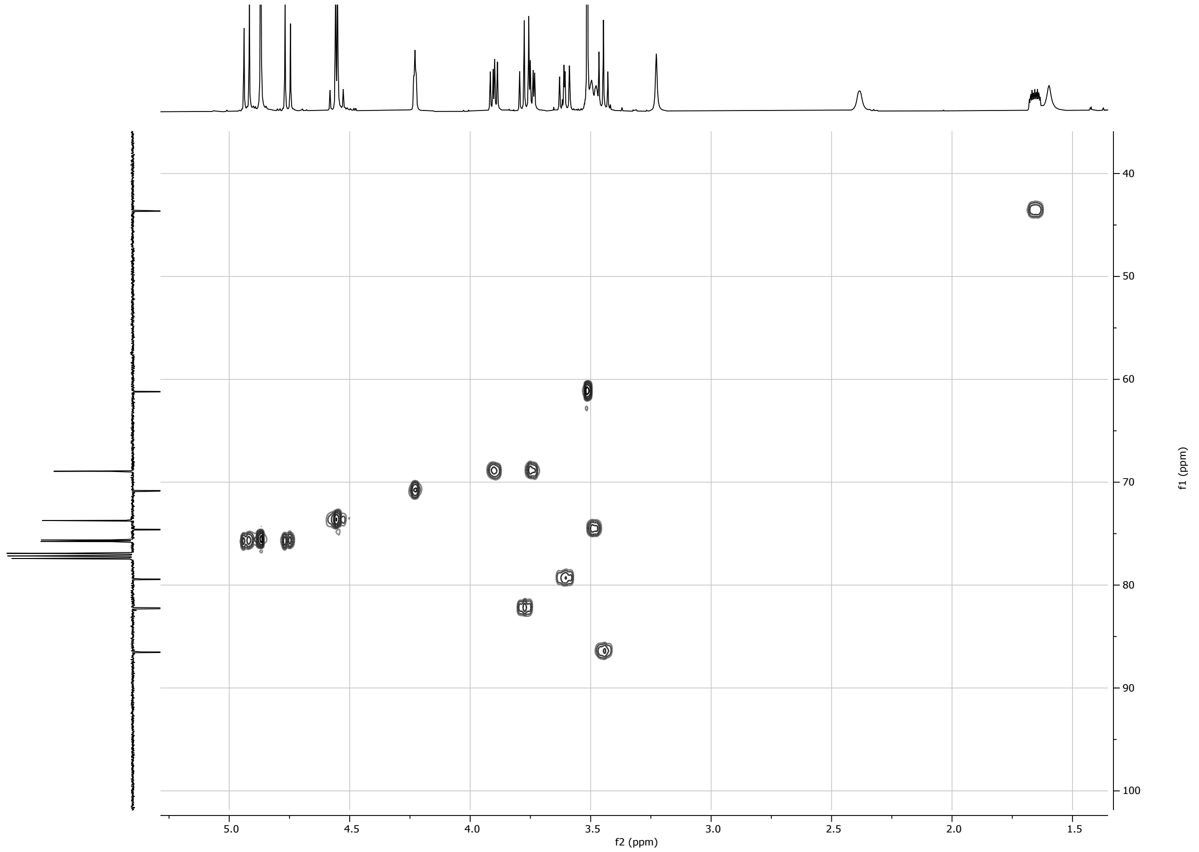

^{1}H NMR, 400MHz, $CDCl_3$ of **55**

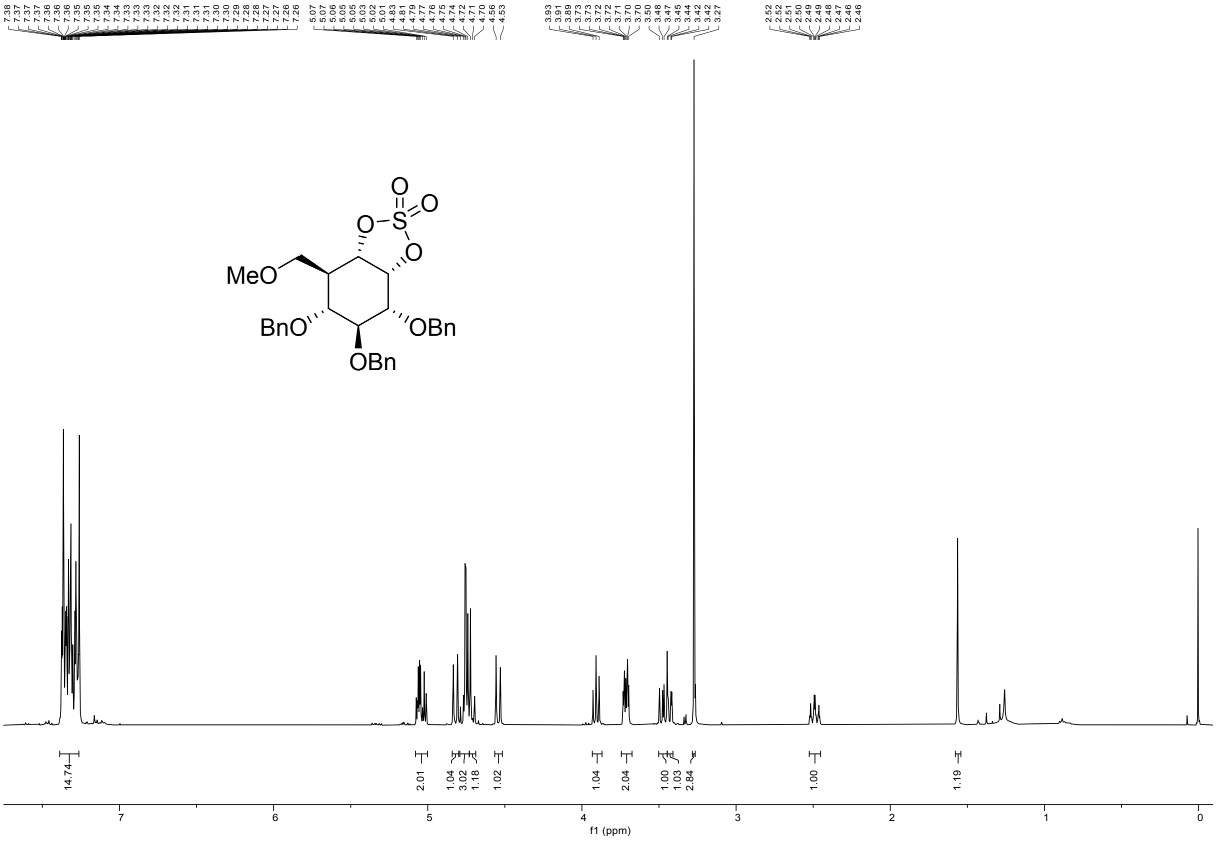

^{13}C NMR, 101MHz, $CDCl_3$ of **55**

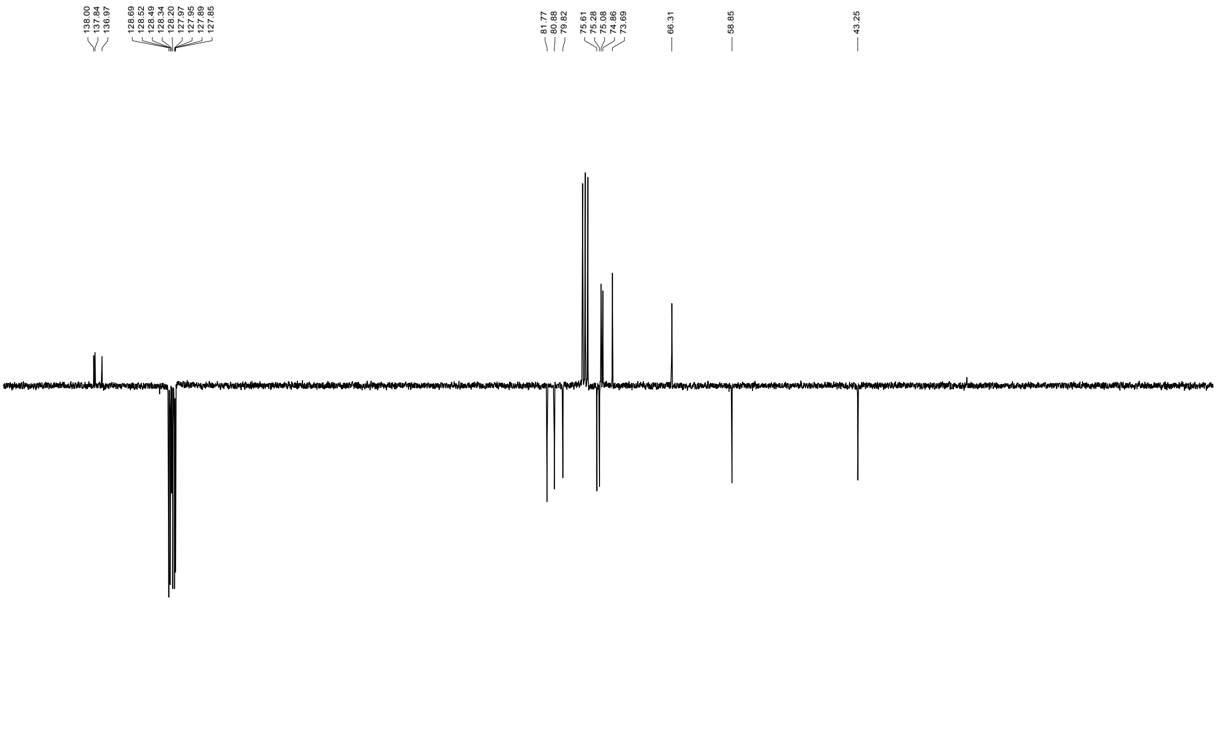

HH-COSY NMR, $CDCl_3$ of **55**

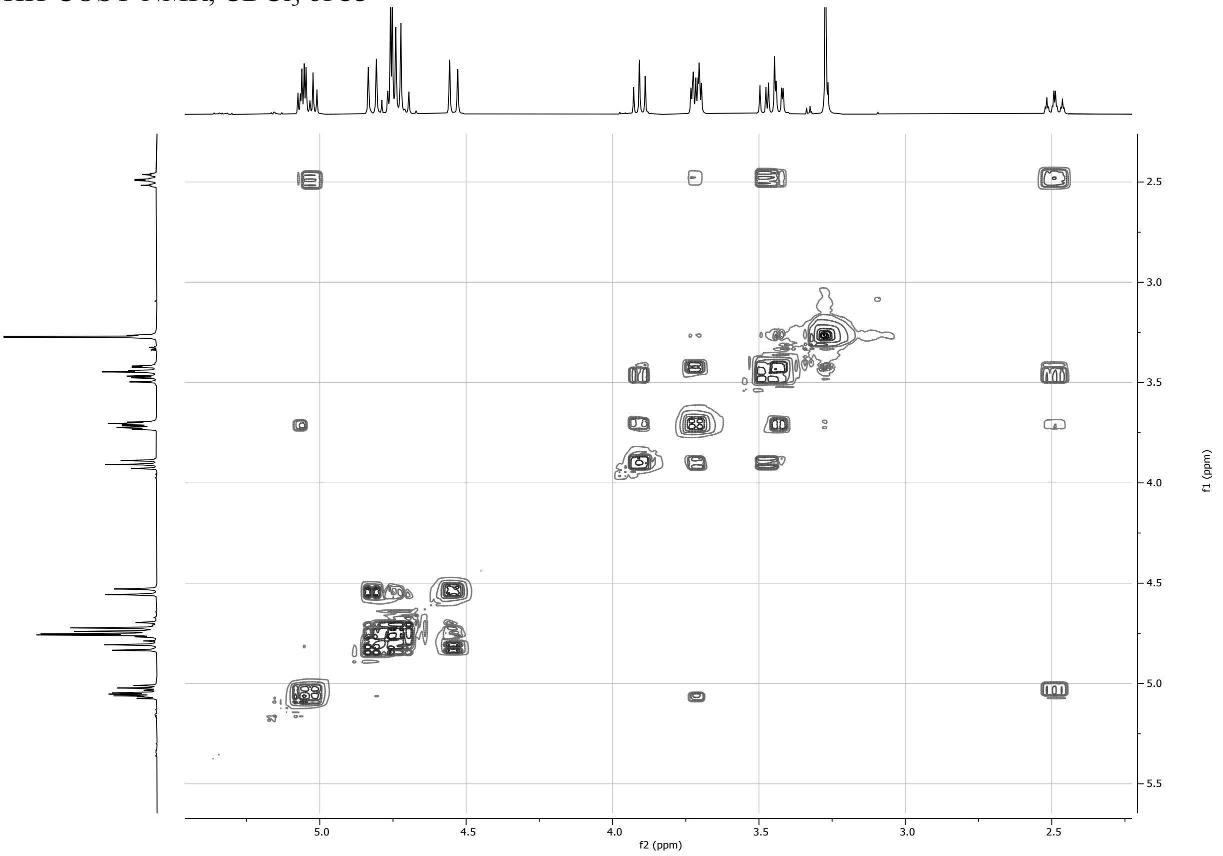

HSQC NMR, $CDCl_3$ of **55**

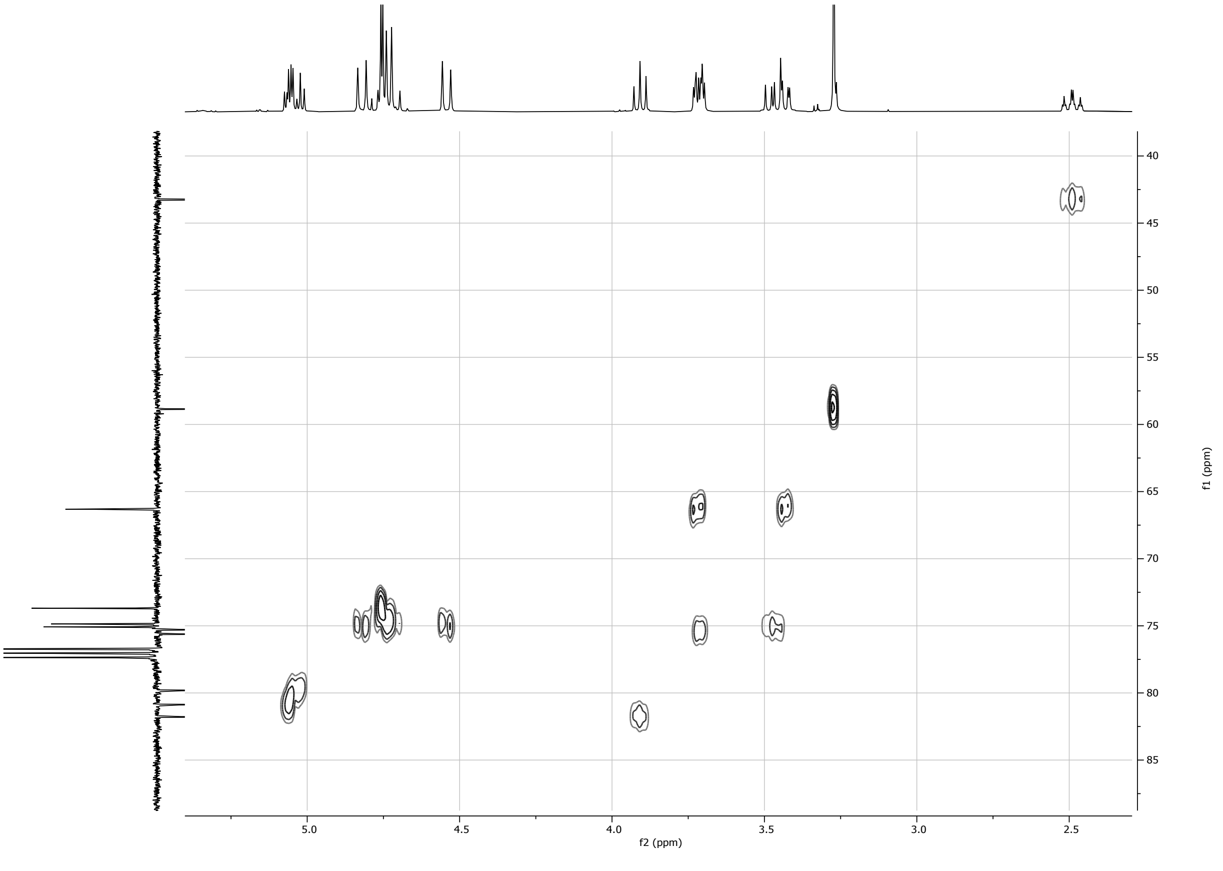

^{1}H NMR, 500MHz, MeOD of **8**

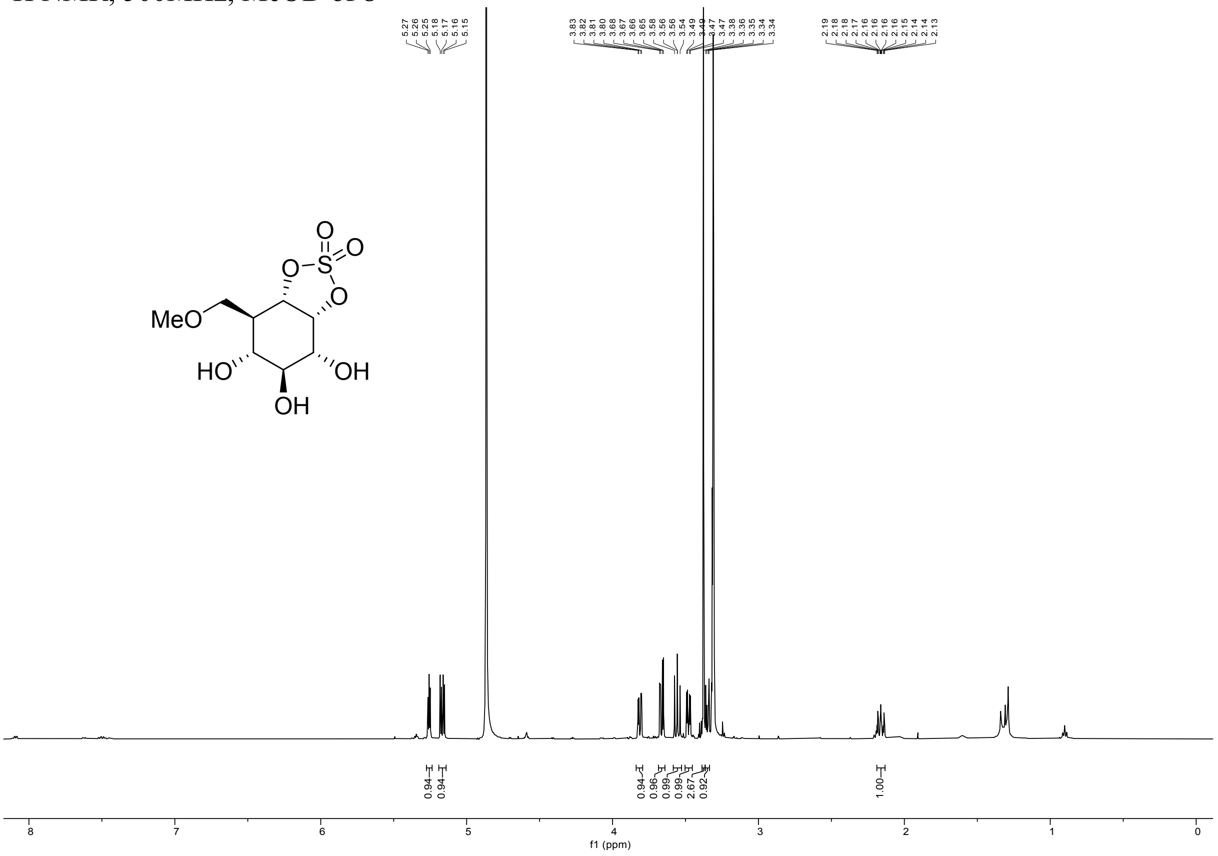

^{13}C NMR, 126MHz, MeOD of **8**

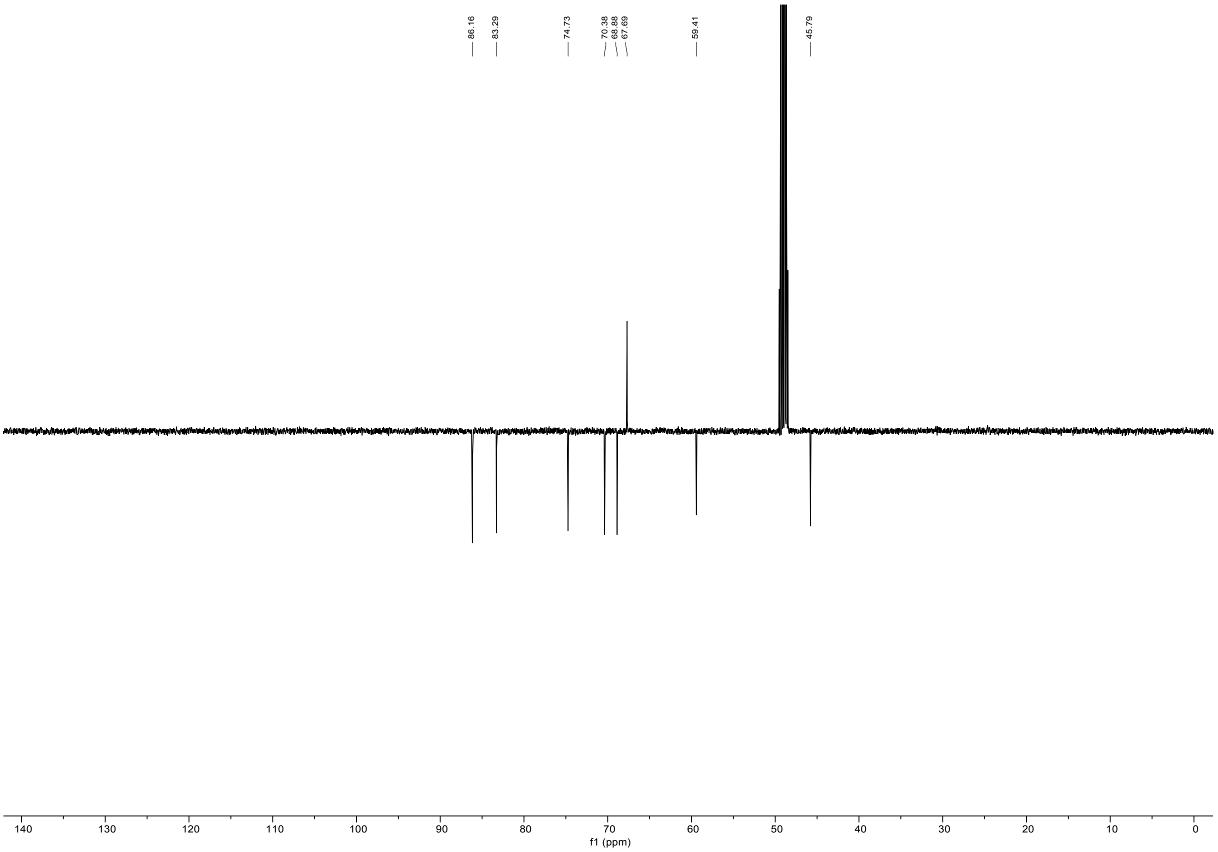

HH-COSY NMR, MeOD of **8**

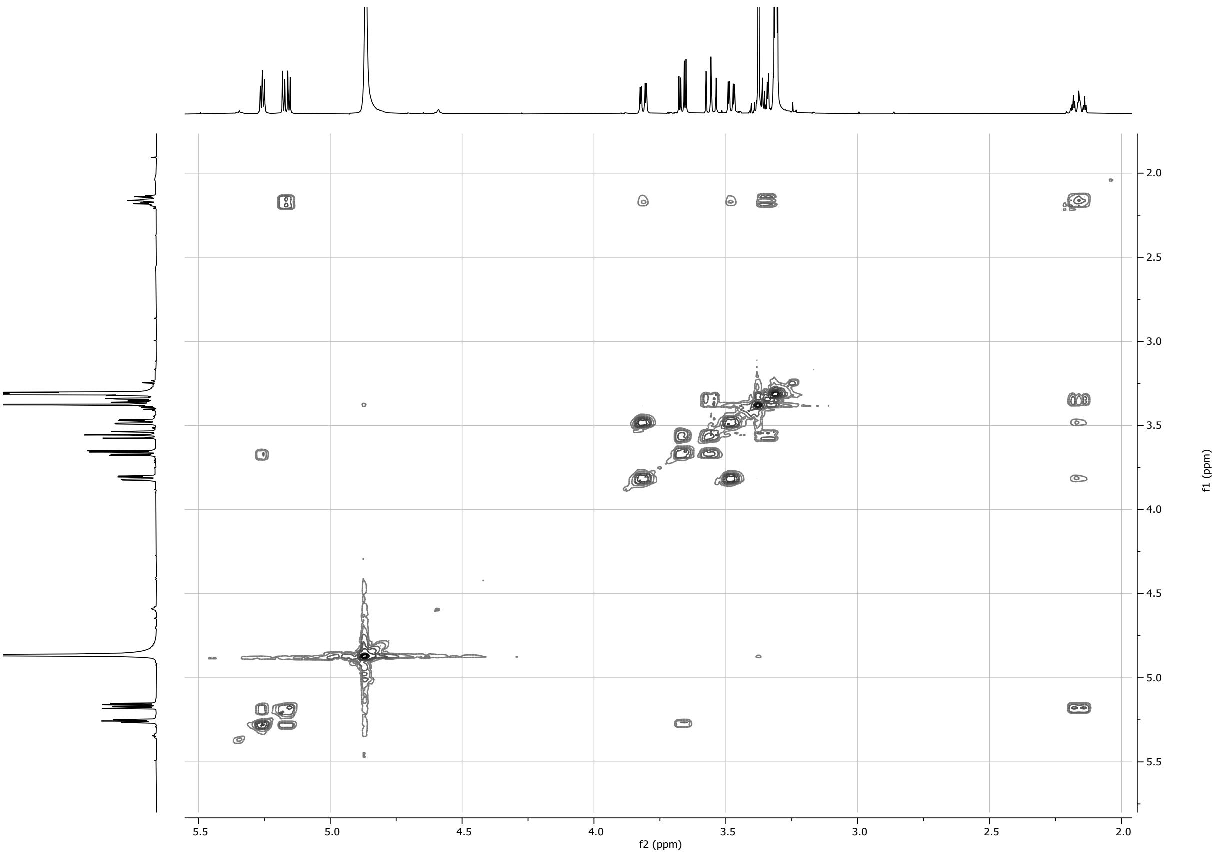

HSQC NMR, MeOD of **8**

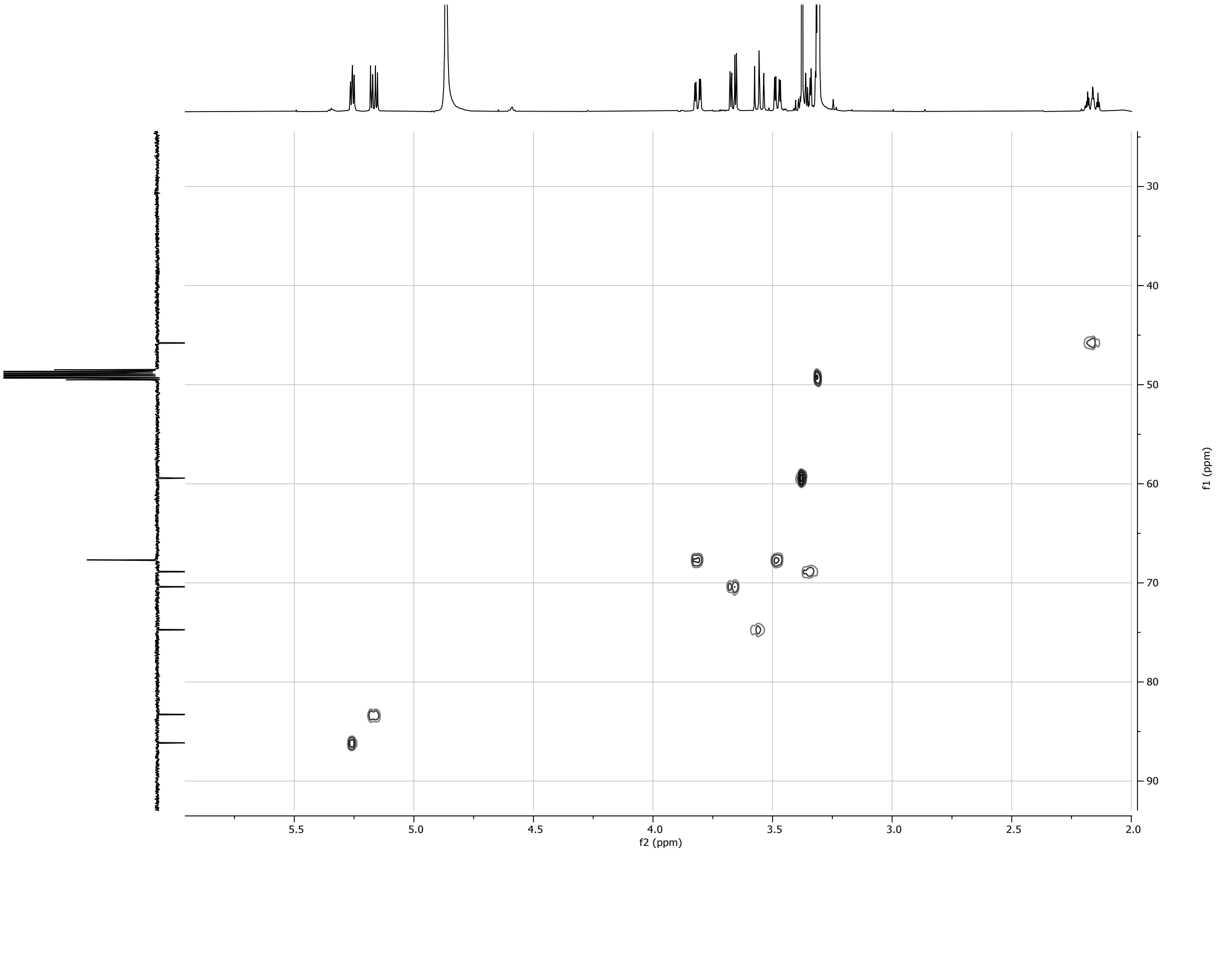

^{1}H NMR, 500MHz, MeOD of **13**

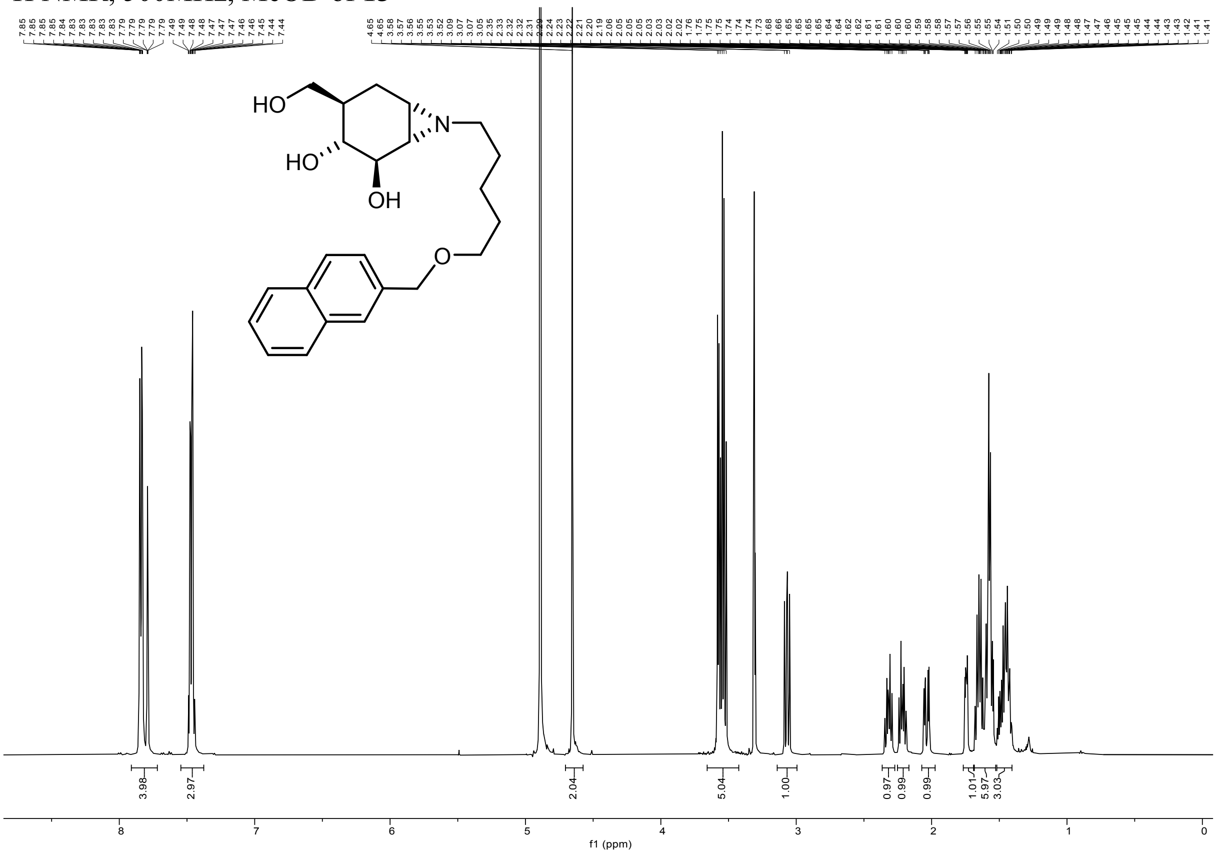

^{13}C NMR, 126MHz, MeOD of **13**

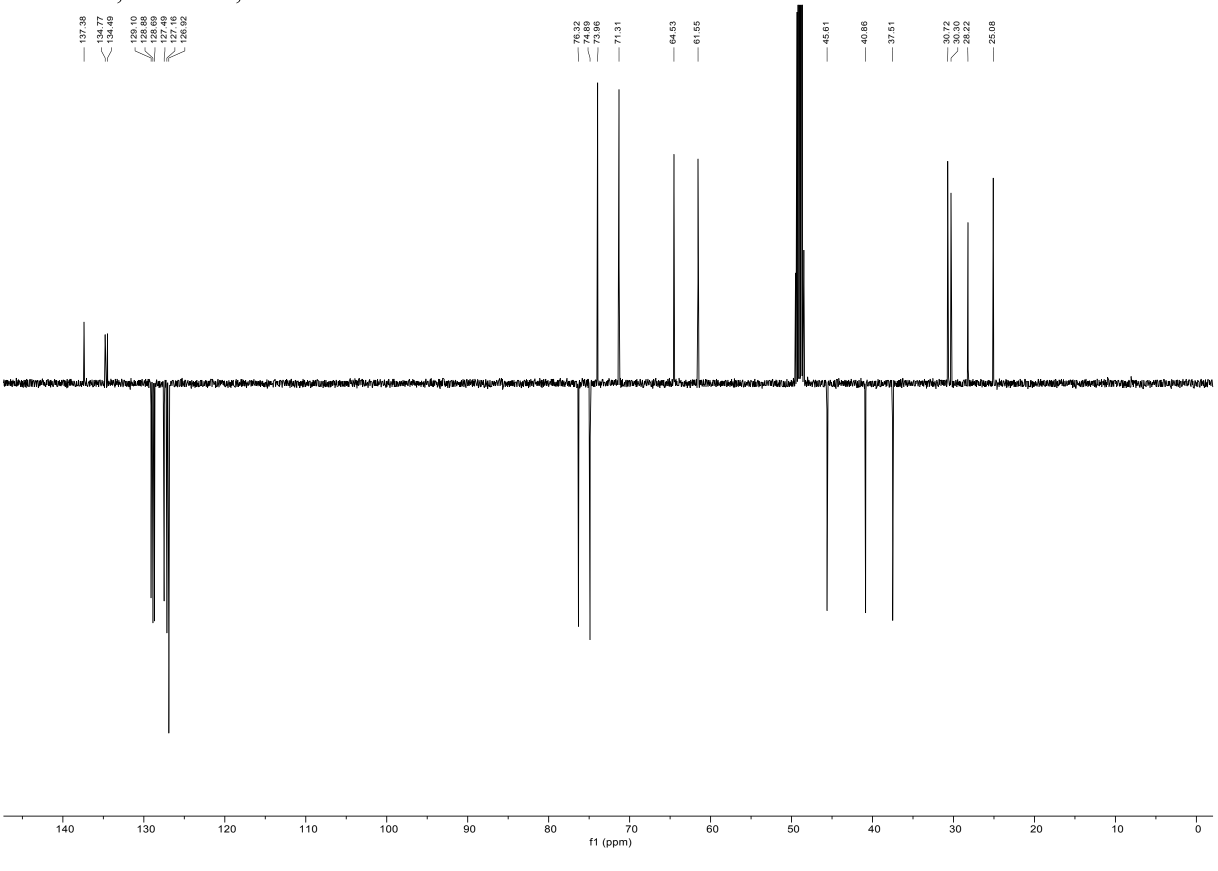

HH-COSY NMR, MeOD of **13**

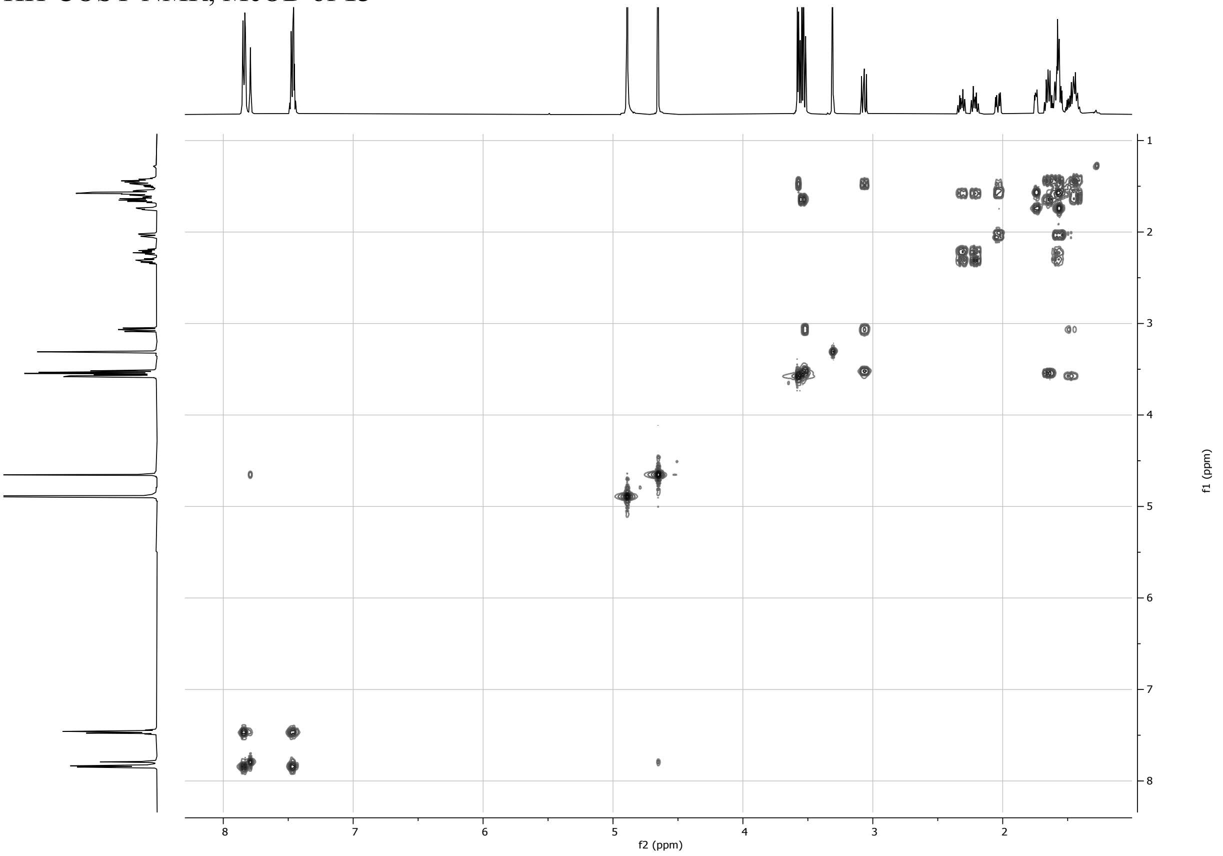

HSQC NMR, MeOD of **13**

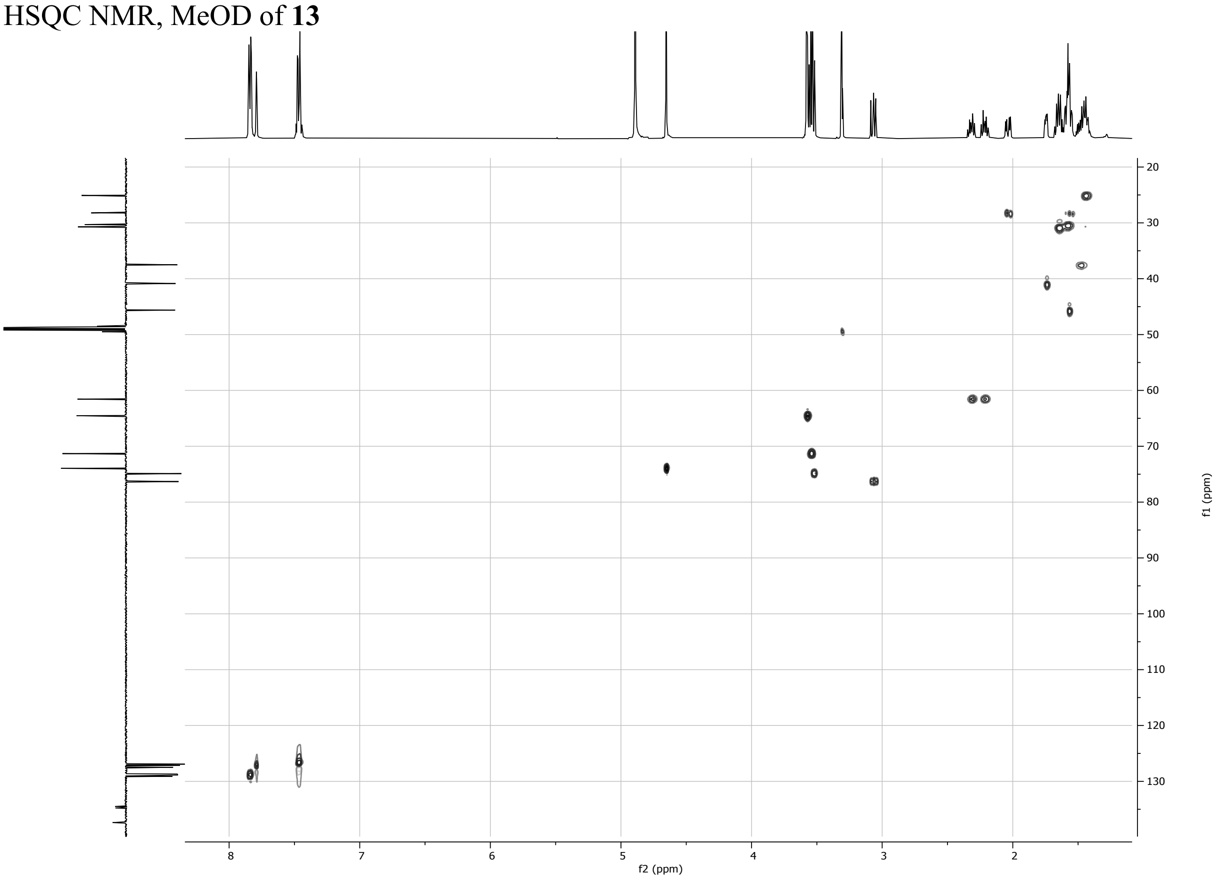

^{1}H NMR, 500MHz, MeOD of **14**

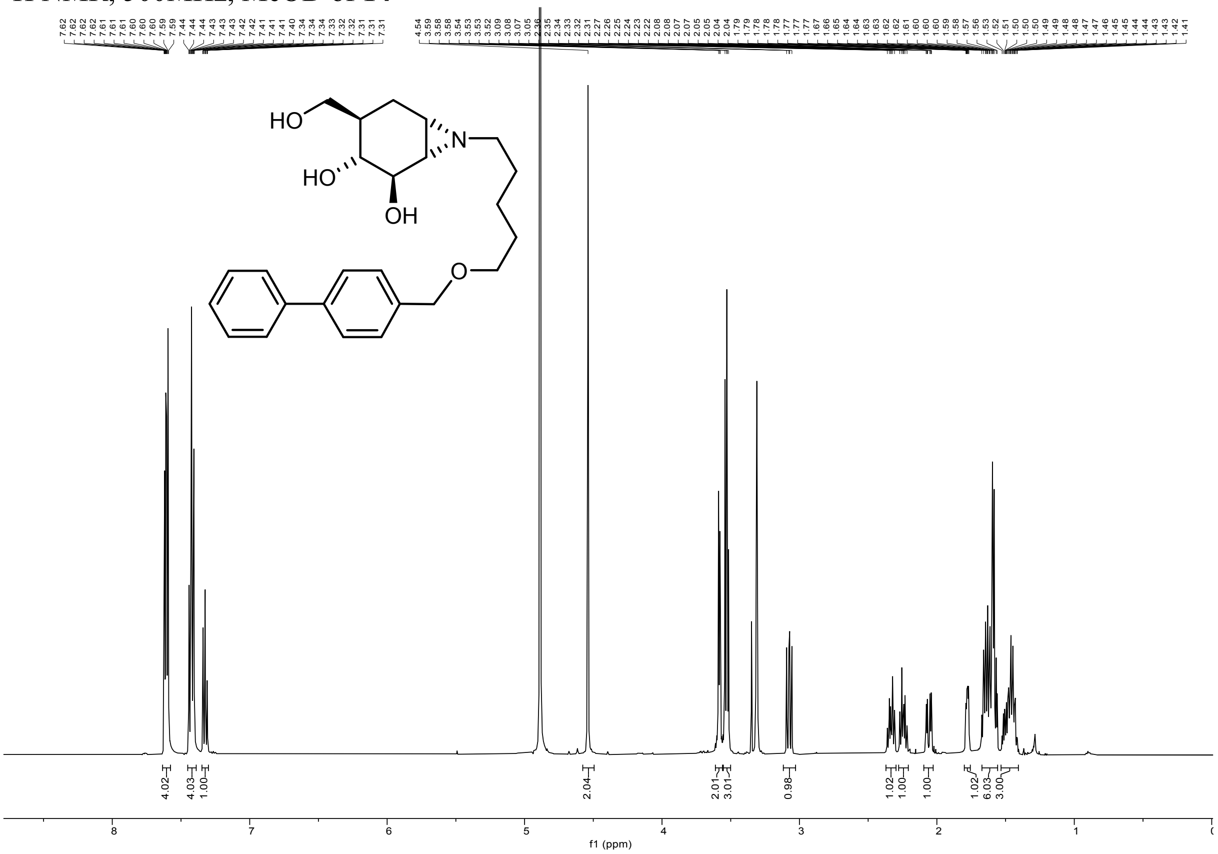

^{13}C NMR, 126MHz, MeOD of **14**

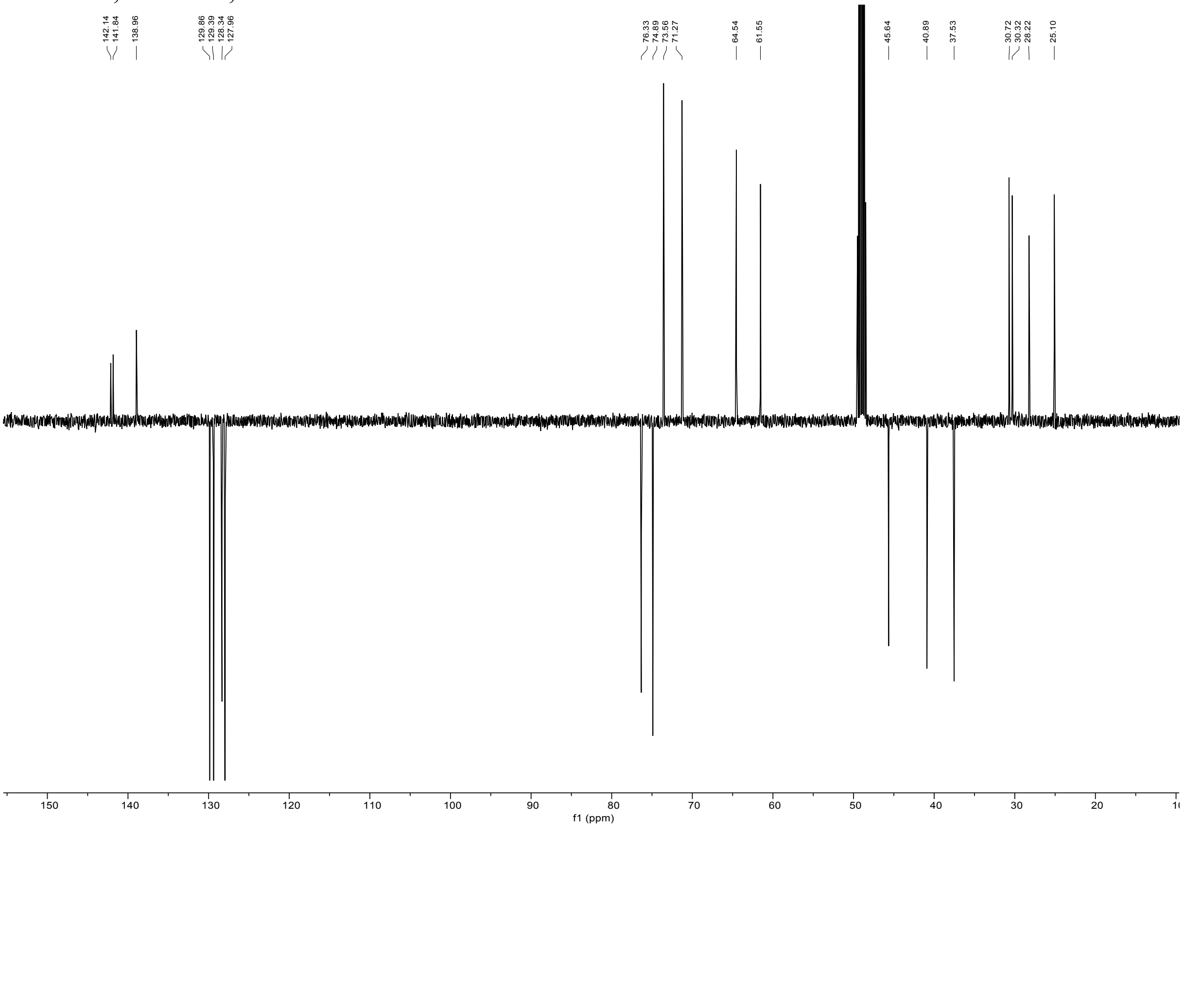

HH-COSY NMR, MeOD of **14**

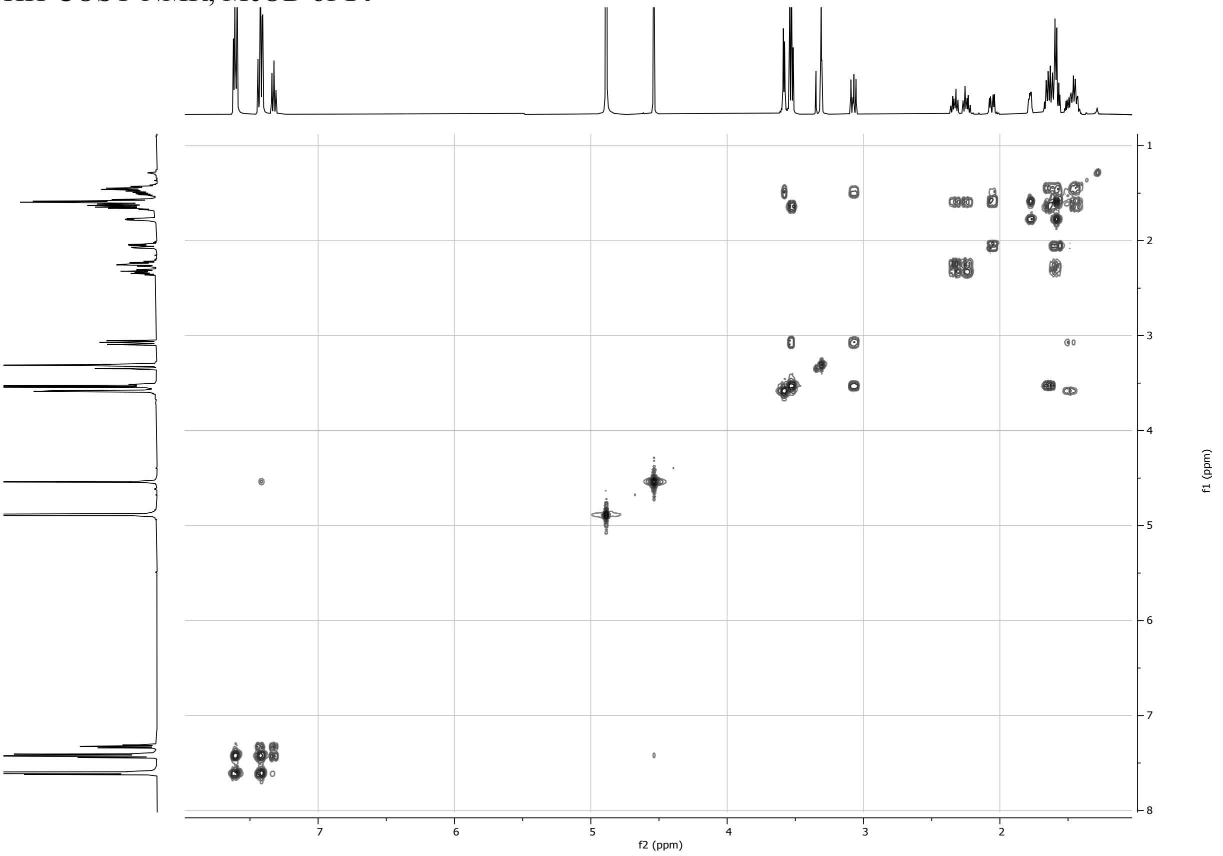

HSQC NMR, MeOD of **14**

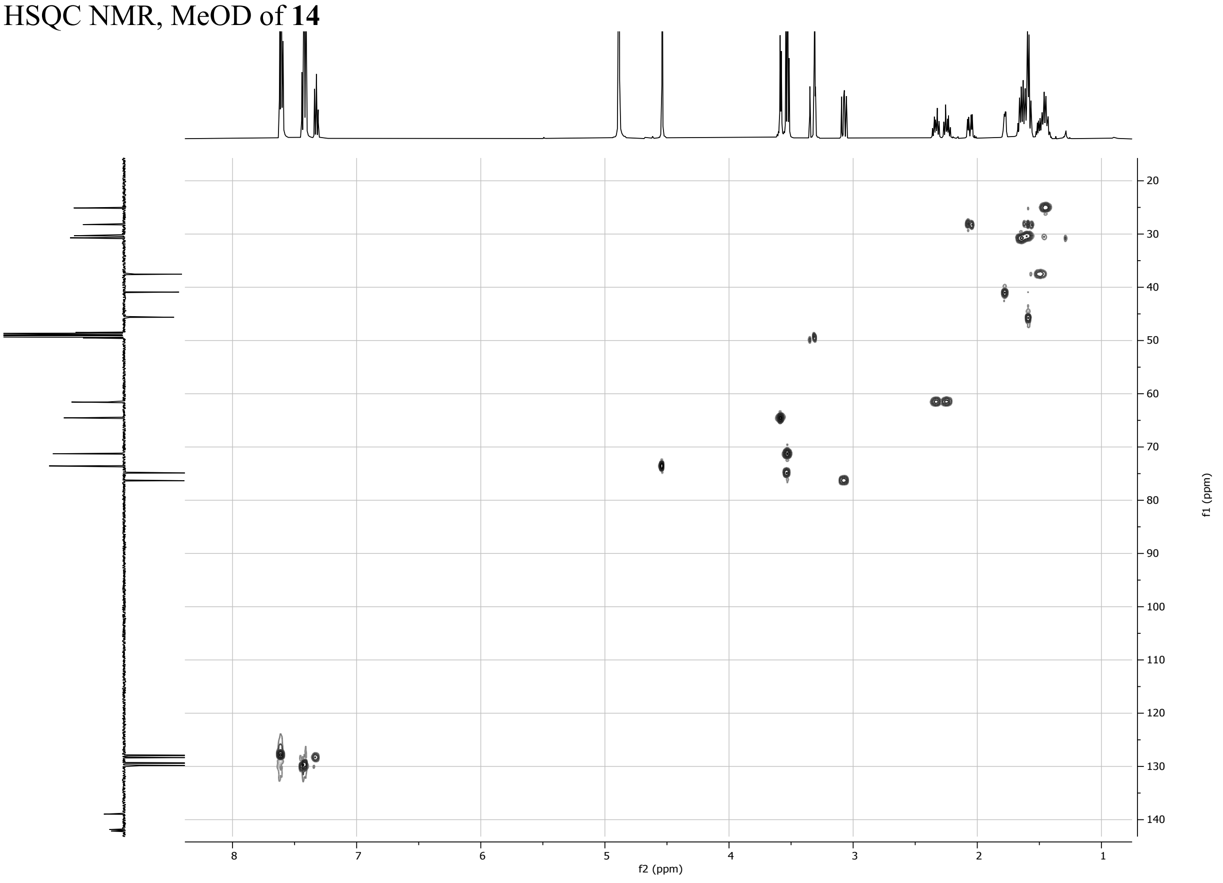

^{1}H NMR, 500MHz, MeOD of **17**

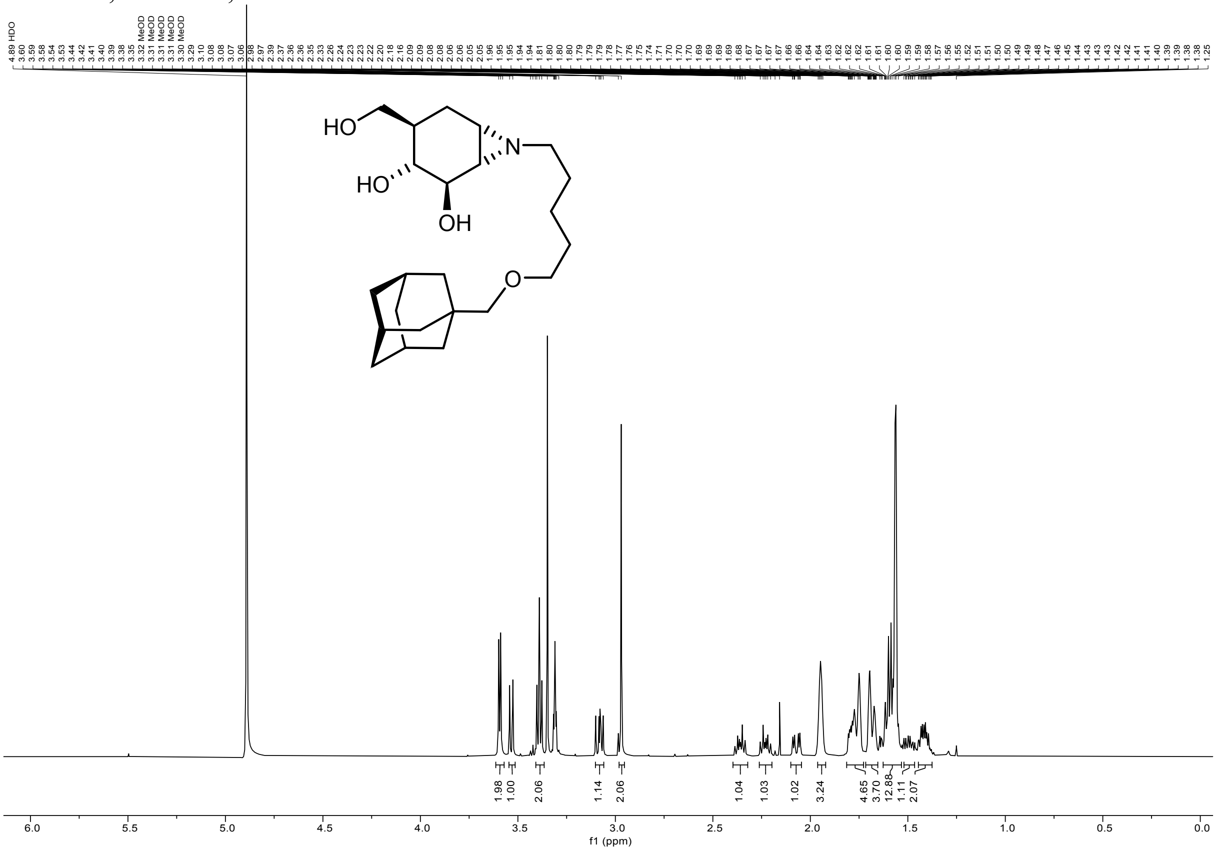

^{13}C NMR, 126MHz, MeOD of **17**

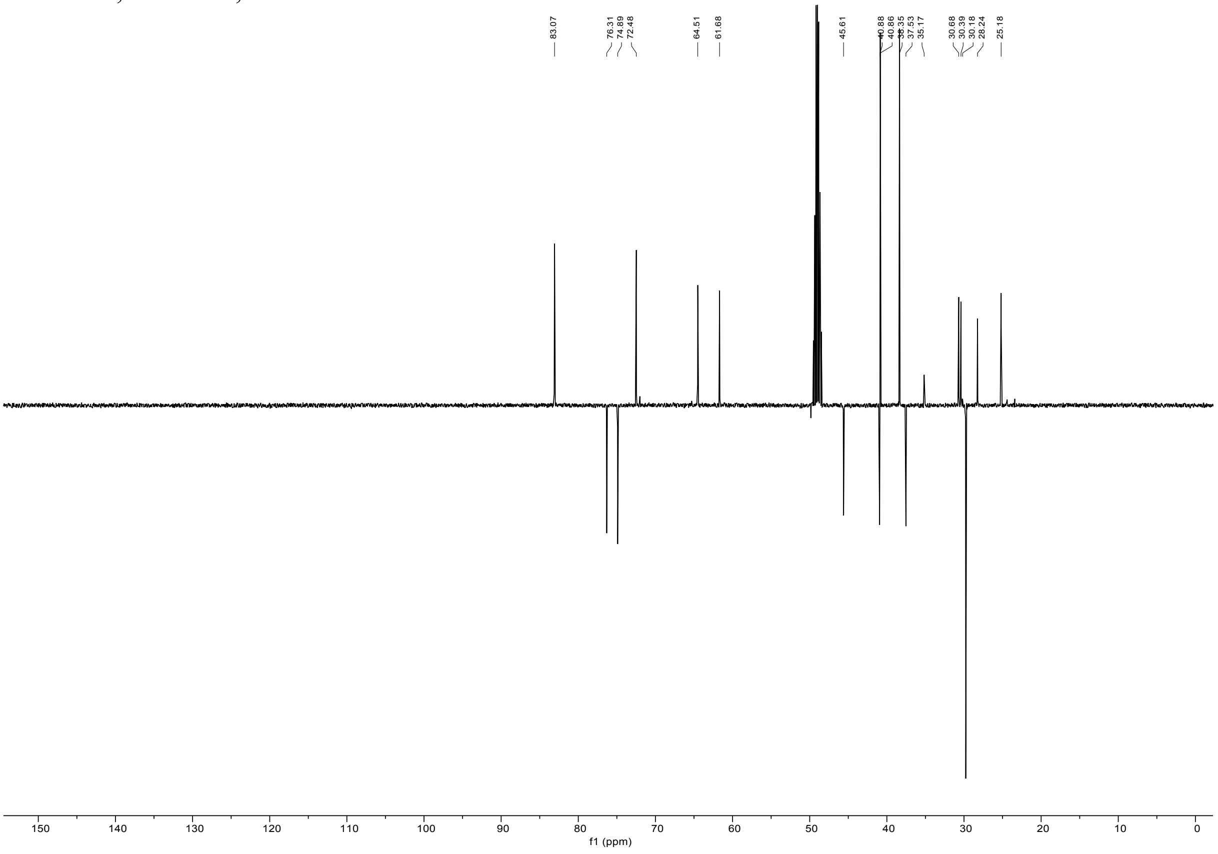

HH-COSY NMR, MeOD of **17**

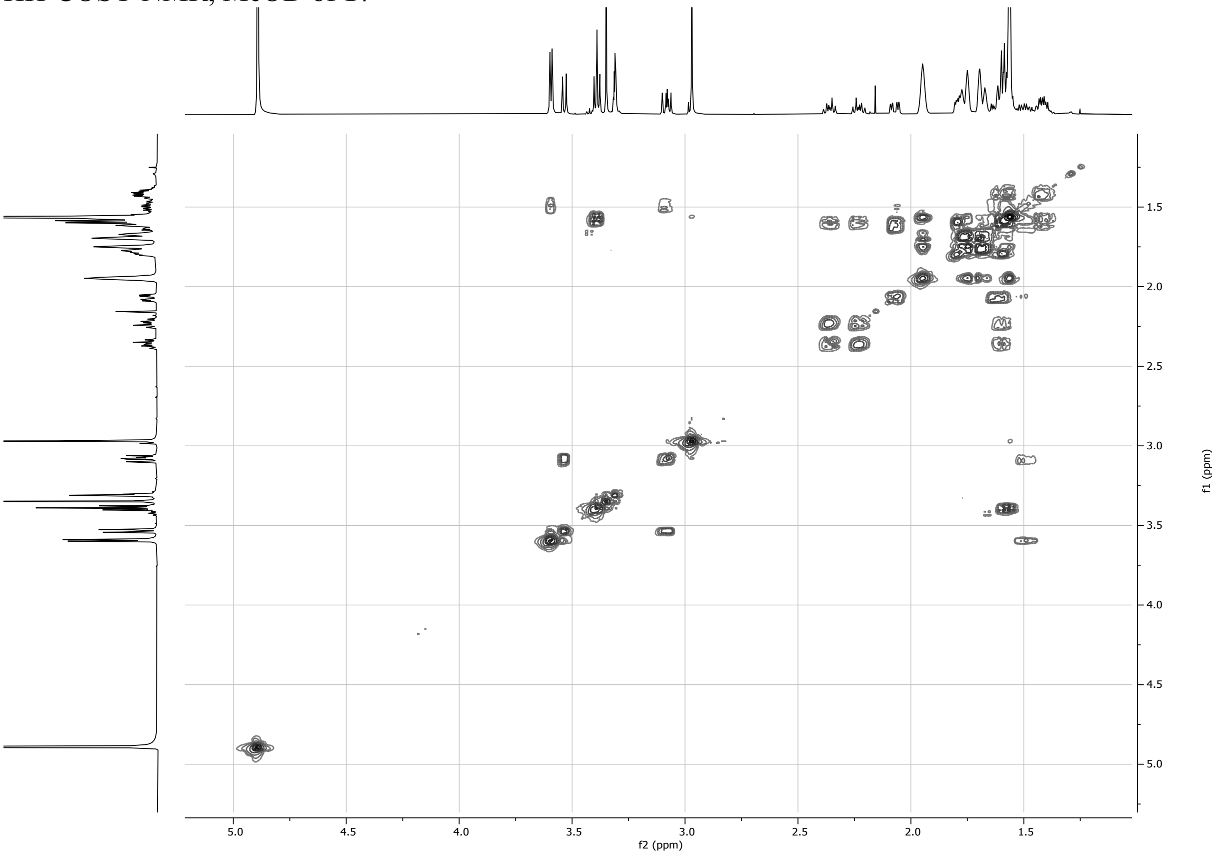

HSQC NMR, MeOD of **17**

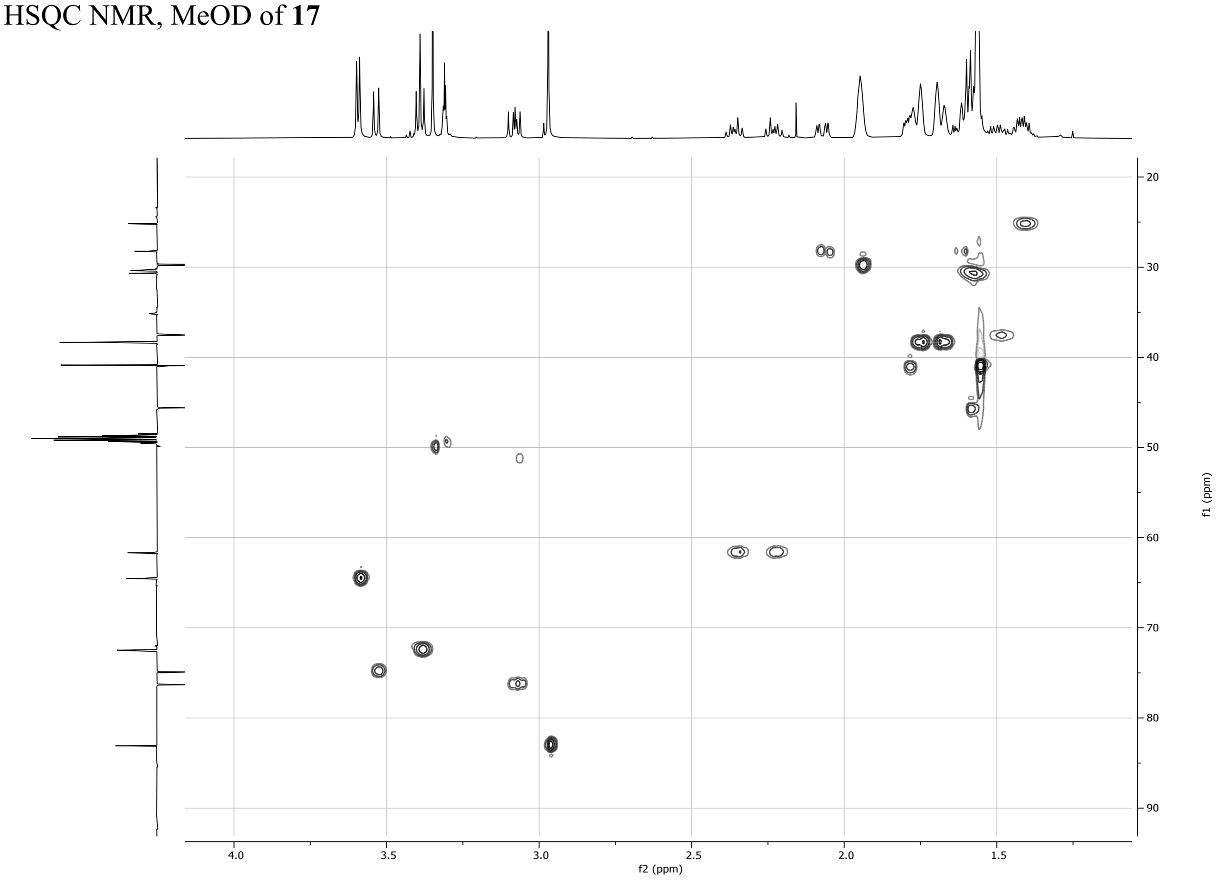

^{1}H NMR, 500MHz, MeOD of **18**

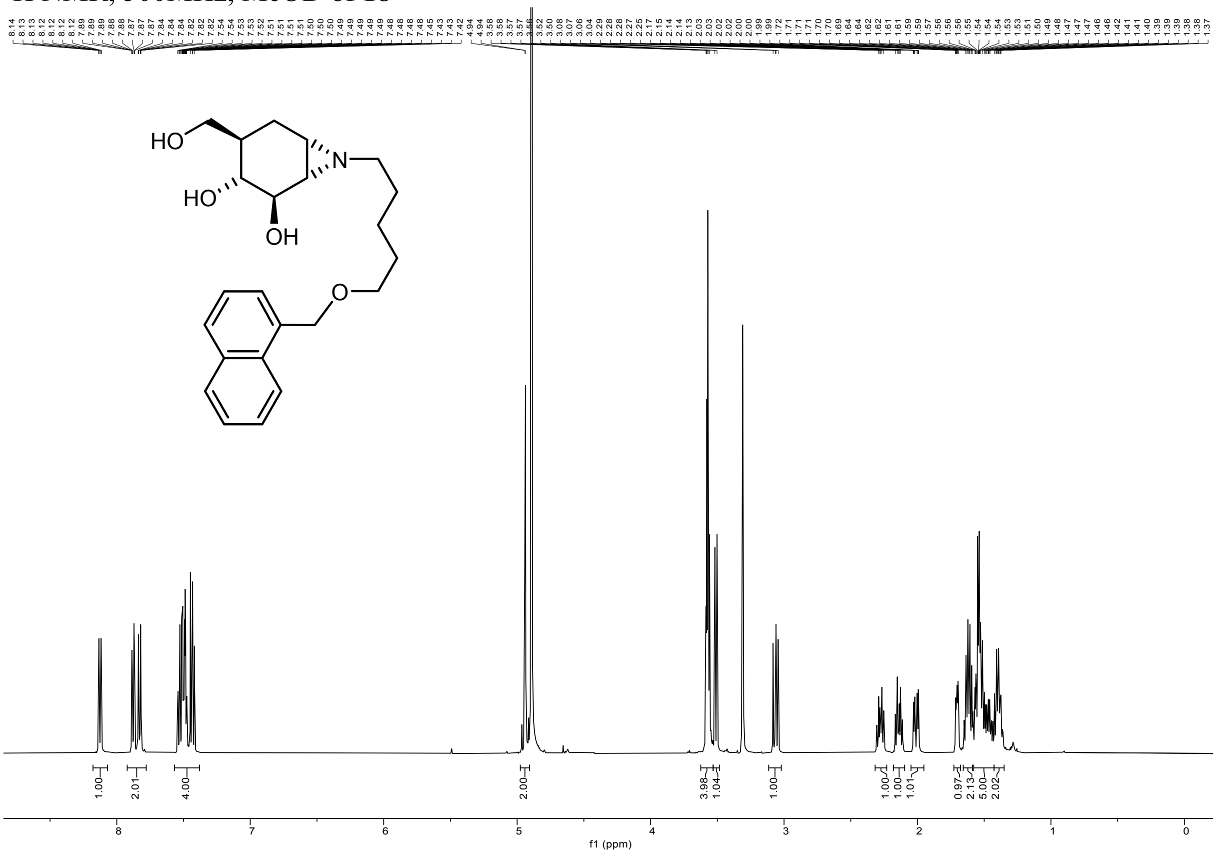

^{13}C NMR, 126MHz, MeOD of **18**

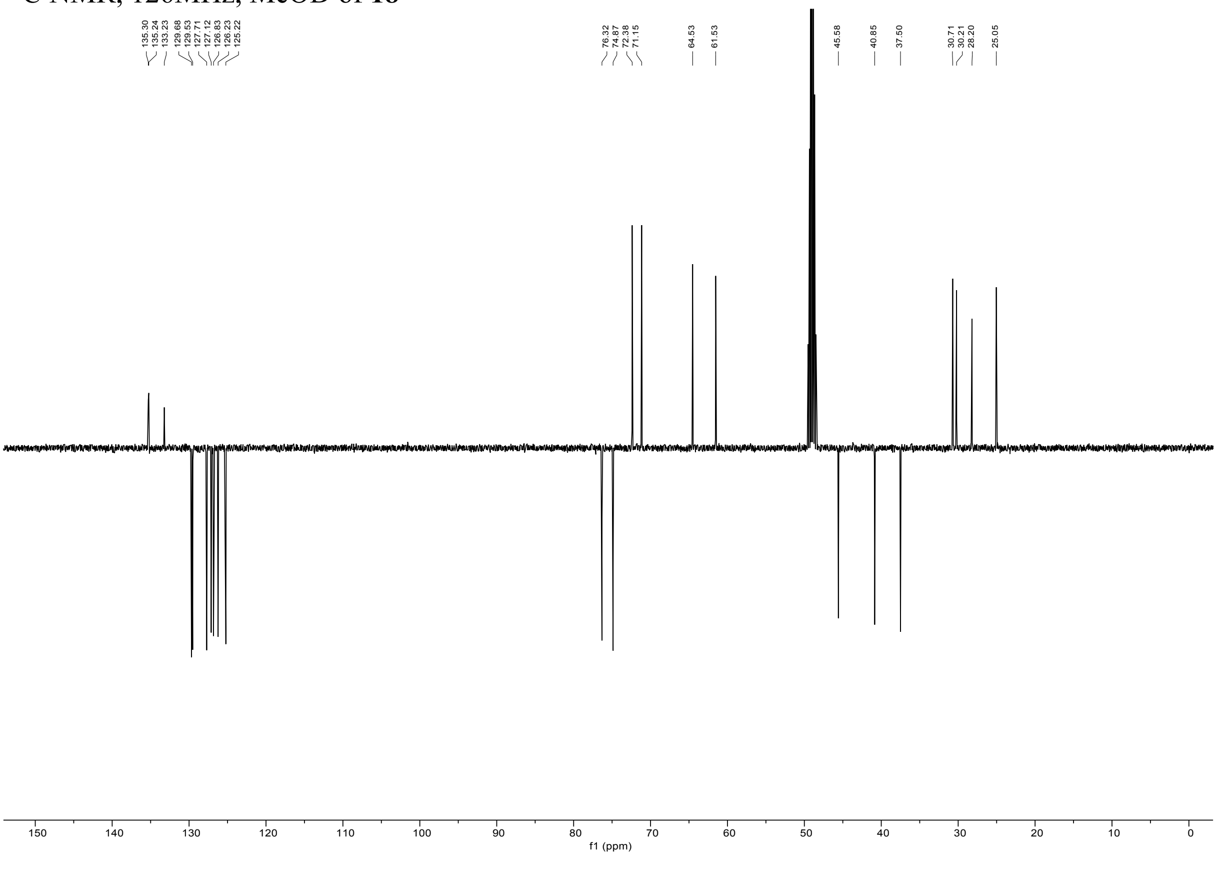

HH-COSY NMR, MeOD of **18**

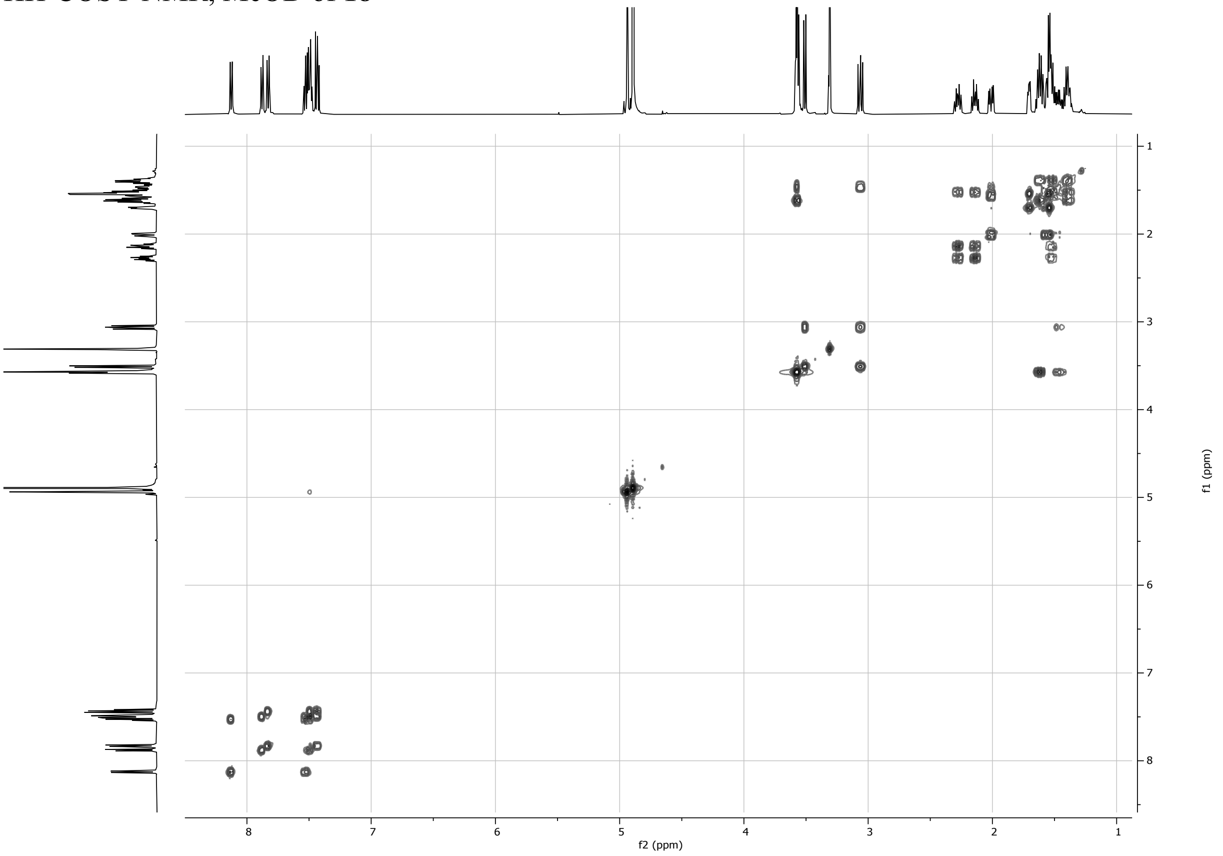

HSQC NMR, MeOD of **18**

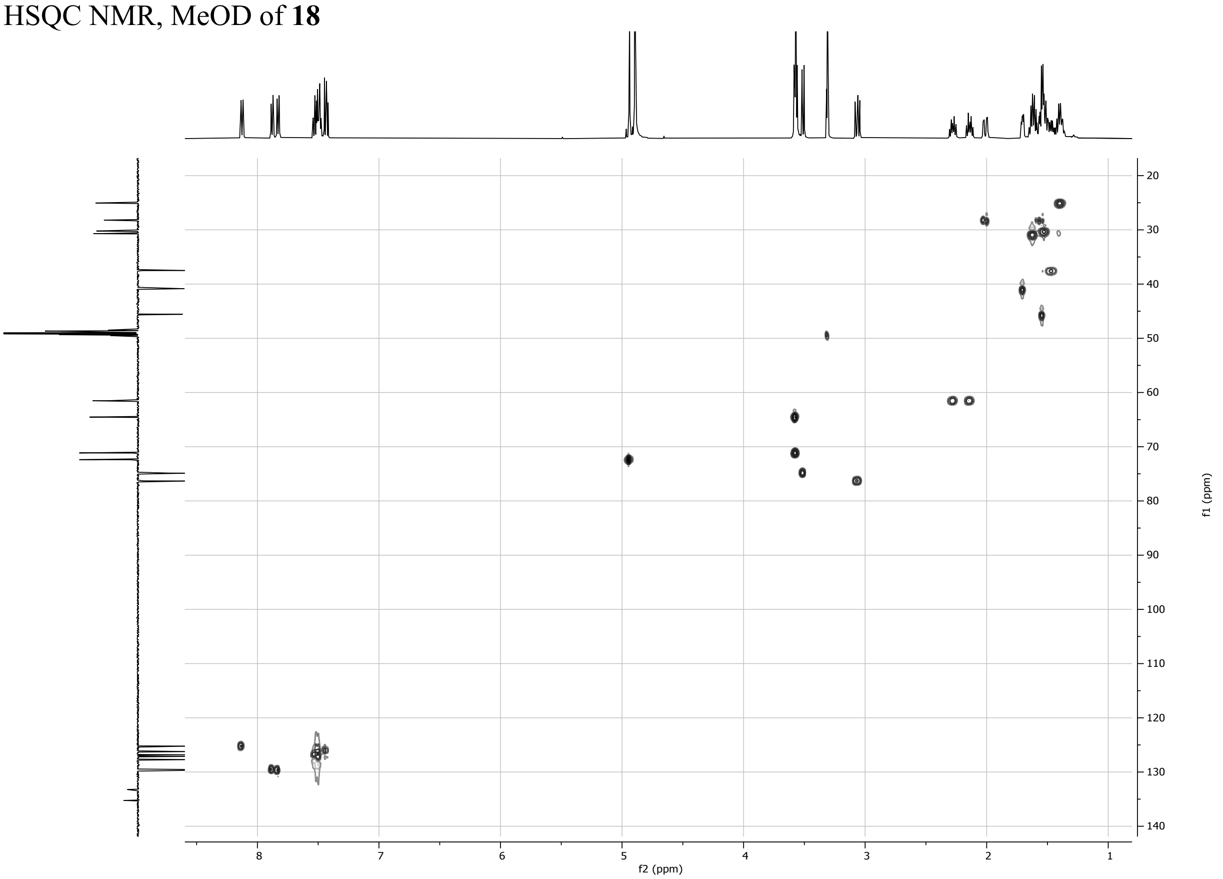

References

[1] Wennekes, T.; Meijer, A. J.; Groen, A. K.; Boot, R. G.; Groener, J. E.; Van Eijk, M.; Ottenhoff, R.; Bijl, N.; Ghauharali, K.; Song, H.; O'Shea, T. J.; Liu, H.; Yew, N.; Copeland, D.; Van Den Berg, R. J.; Van Der Marel, G. A.; Overkleeft, H. S.; Aerts, J. M. Dual-Action Lipophilic Iminosugar Improves Glycemic Control in Obese Rodents by Reduction of Visceral Glycosphingolipids and Buffering of Carbohydrate Assimilation. J Med Chem 2010, 53, 689–698.

[2] Lahav, D.; Liu, B.; Van Den Berg, R. J. B. H. N.; Van Den Nieuwendijk, A. M. C. H.; Wennekes, T.; Ghisaidoobe, A. T.; Breen, I.; Ferraz, M. J.; Kuo, C. L.; Wu, L.; Geurink, P. P.; Ovaa, H.; Van Der Marel, G. A.; Van Der Stelt, M.; Boot, R. G.; Davies, G. J.; Aerts, J. M. F. G.; Overkleeft, H. S. A Fluorescence Polarization Activity-Based Protein Profiling Assay in the Discovery of Potent, Selective Inhibitors for Human Nonlysosomal Glucosylceramidase. J Am Chem Soc 2017, 139, 14192–14197.

[3] Ghisaidoobe, A. T.; Van Den Berg, R. J. B. H. N.; Butt, S. S.; Strijland, A.; Donker-Koopman, W. E.; Scheij, S.; Van Den Nieuwendijk, A. M. C. H.; Koomen, G. J.; Van Loevezijn, A.; Leemhuis, M.; Wennekes, T.; Van Der Stelt, M.; Van Der Marel, G. A.; Van Boeckel, C. A. A.; Aerts, J. M. F. G.; Overkleeft, H. S. Identification and Development of Biphenyl Substituted Iminosugars as Improved Dual Glucosylceramide Synthase/Neutral Glucosylceramidase Inhibitors. J Med Chem 2014, 57, 9096–9104.

[4] Ghisaidoobe, A.; Bikker, P.; De Bruijn, A. C. J.; Godschalk, F. D.; Rogaar, E.; Guijt, M. C.; Hagens, P.; Halma, J. M.; Van't Hart, S. M.; Luitjens, S. B.; Van Rixel, V. H. S.; Wijzenbroek, M.; Zweegers, T.; Donker-Koopman, W. E.; Strijland, A.; Boot, R.; Van Der Marel, G.; Overkleeft, H. S.; Aerts, J. M. F. G.; Van Den Berg, R. J. B. H. N. Identification of Potent and Selective Glucosylceramide Synthase Inhibitors from a Library of N-Alkylated Iminosugars. ACS Med Chem Lett 2011, 2, 119–123.

[5] https://doi.org/10.1073/pnas.1604463113

[6] Sastry, G. M.; Adzhigirey, M.; Day, T.; Annabhimoju, R.; Sherman, W., "Protein and ligand preparation: Parameters, protocols, and influence on virtual screening enrichments", J. Comput. Aid. Mol. Des., 2013, 27(3), 221-234

[7] Yang, Y; Yao, K; Repasky, M. P.; Leswing, K; Abel, R; Shoichet, B. K.; Jerome, S. V., "Efficient exploration of chemical space with docking and deep learning", J. Chem. Theory Comput. 2021, 17(11), 7106–7119

[8] Friesner, R. A.; Murphy, R. B.; Repasky, M. P.; Frye, L. L.; Greenwood, J. R.; Halgren, T. A.; Sanschagrin, P. C.; Mainz, D. T., "Extra precision Glide: Docking and scoring incorporating a model of hydrophobic enclosure for protein-ligand complexes", J. Med. Chem., 2006, 49, 6177–6196

[9] Halgren, T. A.; Murphy, R. B.; Friesner, R. A.; Beard, H. S.; Frye, L. L.; Pollard, W. T.; Banks, J. L., "Glide: A new approach for rapid, accurate docking and scoring. 2. Enrichment factors in database screening", J. Med. Chem., 2004, 47, 1750–1759

[10] Friesner, R. A.; Banks, J. L.; Murphy, R. B.; Halgren, T. A.; Klicic, J. J.; Mainz, D. T.; Repasky, M. P.; Knoll, E. H.; Shaw, D. E.; Shelley, M.; Perry, J. K.; Francis, P.; Shenkin, P. S., "Glide: A new approach for rapid, accurate docking and scoring. 1. Method and assessment of docking accuracy", J. Med. Chem., 2004, 47, 1739–1749

[11] Lu, C.; Wu, C.; Ghoreishi, D.; Chen, W.; Wang, L.; Damm, W.; Ross, G. A.; Dahlgren, M. K.; Russell, E.; Von Bargen, C. D.; Abel, R.; Friesner, R. A.; Harder, E. D., "OPLS4: Improving force field accuracy on challenging regimes of chemical space", J. Chem. Theory Comput., 2021, 17(7), 4291–4300

[12] Johnston, R. C.; Yao, K.; Kaplan, Z.; Chelliah, M.; Leswing, K.; Seekins, S.; Watts, S.; Calkins, D.; Chief Elk, J.; Jerome, S. V.; Repasky, M. P;. Shelley, J. C., "Epik: pKa and protonation state prediction through machine learning", J. Chem. Theory Comput. 2023, 19, 2380–2388

[13] Zhu, K.; Borrelli, K. W.; Greenwood, J. R.; Day, T.; Abel, R.; Farid, R. S.; Harder, E., "Docking covalent inhibitors: A parameter free approach to pose prediction and scoring", J. Chem. Inf. Model., 2014, 54, 1932−1940

[14] UCSF ChimeraX: Tools for structure building and analysis. Meng EC, Goddard TD, Pettersen EF, Couch GS, Pearson ZJ, Morris JH, Ferrin TE. Protein Sci. 2023 Nov;32(11):e4792.

[15] Salgado-Benvindo C, Leijs AA, Thaler M, Tas A, Arbiser JL, Snijder EJ, van Hemert MJ. 2023. Honokiol Inhibits SARS-CoV-2 Replication in Cell Culture at a Post-Entry Step. Microbiol Spectr 11:e0327322.
